# Comparative efficacy and certainty of evidence of exercise interventions for cognitive outcomes in older adults with mild cognitive impairment: a systematic review and network meta-analysis of randomized controlled trials

**DOI:** 10.3389/fnagi.2026.1866613

**Published:** 2026-07-07

**Authors:** Bu-Yu Liu, Chang-Kuan Zhuang, Hui Ruan

**Affiliations:** School of Physical Education, Hainan Normal University, Haikou, China

**Keywords:** cognitive function, exercise intervention, mild cognitive impairment, network meta-analysis, older adults

## Abstract

**Background:**

Mild cognitive impairment (MCI) is common in older adults and is linked to dementia, functional decline, and poorer quality of life. Exercise is recommended as a non-pharmacological strategy, but comparative effects of exercise modalities, alone or combined with cognitive training, remain uncertain. This systematic review and network meta-analysis compared exercise-based interventions for cognitive and psychological outcomes in older adults with MCI.

**Methods:**

Following a registered PROSPERO protocol (CRD420261372312), we searched PubMed, Embase, Cochrane Library, and Web of Science from inception to March 1, 2026. Eligible studies were randomized controlled trials in older adults with MCI. Intervention nodes included aerobic exercise, resistance training, mind–body exercise, multicomponent exercise, digital or virtual reality–based exercise, cognitive training, and combined exercise–cognitive training. Outcomes included global cognition, executive function, attention/working memory, depression-related assessments (DRA), and memory function assessments (MFA). Risk of bias, rankings, and certainty were assessed using RoB 2, SUCRA, and GRADE.

**Results:**

Seventy-four RCTs involving 5,578 participants and 12 intervention nodes were included. Compared with wait-list control, mind–body exercise was associated with higher MoCA (MD = 3.94, 95% CI 2.28–5.59) and MMSE scores (MD = 4.52, 95% CI 2.34–6.70; moderate certainty). Resistance training was associated with lower ADAS-Cog scores (MD = −5.02, 95% CI −7.30 to −2.74; high certainty). Digital or virtual reality–based exercise was associated with faster TMT-B performance (MD = −48.40, 95% CI −77.22 to −19.58; moderate certainty). Multicomponent exercise was associated with lower DRA scores (SMD = −0.56, 95% CI −0.96 to −0.16; moderate certainty) and better DST performance (SMD = 1.70, 95% CI 0.71–2.69; high certainty). SCWT and MFA showed favorable but statistically uncertain trends.

**Conclusion:**

Exercise-based interventions may be associated with favorable cognitive and psychological outcomes in older adults with MCI, with effects varying by domain. Favorable estimates were observed for mind–body exercise in global cognition, resistance training in ADAS-Cog, digital or virtual reality–based exercise in executive function, and multicomponent exercise in DST and DRA. Findings should be interpreted cautiously given evidence certainty, network sparsity, and estimate precision. Further head-to-head trials with standardized protocols are warranted.

**Systematic review registration:**

https://www.crd.york.ac.uk/PROSPERO/view/CRD420261372312, identifier CRD420261372312.

## 1 Background

Mild cognitive impairment (MCI) is widely regarded as an intermediate clinical state between normal ageing and dementia (Albert et al., 2011; Petersen et al., 1999). MCI is typically defined by the coexistence of subjective cognitive concerns and objectively measurable cognitive impairment, with basic activities of daily living generally remaining intact; therefore, it does not meet established diagnostic criteria for dementia (Albert et al., 2011). MCI is common in later life, with pooled prevalence estimates from previous systematic reviews and meta-analyses ranging from 19.7 to 23.7% (Salari et al., 2025; Song et al., 2023). Rather than a benign manifestation of ageing, MCI is increasingly recognized as a clinically important risk state for subsequent dementia (Salemme et al., 2025). Its consequences also extend beyond cognitive decline, affecting quality of life, functional independence, and fall risk (Campbell et al., 2025; Davis et al., 2015; Gopalakrishnan et al., 2024; Hopkins et al., 2023; Saykin and Wishart, 2003). In addition, MCI imposes considerable economic and caregiving burdens through increased healthcare use, higher medical costs, and greater demands on families and society (Frech et al., 2025; Gläser et al., 2025; Ritchie et al., 2025; Takechi and Yoshino, 2023). At the population level, the lifetime economic burden attributable to cognitive impairment is substantial, and a meaningful proportion of this burden may begin during the MCI stage (Gracner et al., 2025). These findings highlight the need for effective, scalable, and clinically relevant interventions that can delay cognitive deterioration and mitigate the wider health and societal burden associated with MCI.

For mild cognitive impairment (MCI) as a broad clinical syndrome, no pharmacological treatment has been established as routine standard care. The American Academy of Neurology guideline found no high-quality evidence that drug therapy provides a clear benefit for MCI-related outcomes (Petersen et al., 2018). Pharmacological trials, particularly those evaluating cholinesterase inhibitors, have not shown consistent or clinically meaningful effects. Prior systematic reviews and meta-analyses have likewise suggested that any potential benefit remains uncertain, while concerns about adverse events continue to weigh against routine use in this population (Fitzpatrick-Lewis et al., 2015; Matsunaga et al., 2019). Against this background, non-pharmacological interventions have become an increasingly important focus in both MCI management and research.

Non-pharmacological interventions have therefore become central to both clinical management and research in MCI. The American Academy of Neurology (AAN) guideline states that exercise training delivered over approximately 6 months is likely to improve cognitive outcomes in individuals with MCI, and that cognitive training may also be beneficial. The guideline also recommends regular exercise as part of MCI management, including exercise at least twice weekly. Similarly, the 2019 World Health Organization guideline on risk reduction of cognitive decline and dementia states that physical activity may be recommended for adults with MCI to reduce the risk of further cognitive decline (Petersen et al., 2018; World Health Organization [WHO], 2019).

However, existing guidelines and reviews have mainly addressed whether exercise should be recommended, rather than which exercise modality is most effective, whether exercise should be combined with cognitive training, or how different intervention strategies compare in relative efficacy. Recent evidence syntheses suggest that, although exercise-based interventions are generally supported, uncertainty persists regarding the superiority of specific exercise modalities. Evidence for combined exercise–cognitive interventions also remains insufficiently consistent and definitive (Petersen et al., 2018; Veronese et al., 2023; World Health Organization [WHO], 2019). Consequently, clinicians still lack clear, practical, and evidence-based guidance for prescribing exercise interventions to people with MCI.

In recent years, network meta-analysis (NMA) has been increasingly used to compare the relative effects of competing intervention strategies. Many previous NMAs, however, have placed substantial emphasis on treatment rankings, often identifying the “best” intervention on the basis of SUCRA values or P-scores (Seok et al., 2024; Yu et al., 2024). Methodological work has shown that these rankings are driven largely by relative effect estimates and can be misleading when interpreted without sufficient attention to precision, heterogeneity, inconsistency, and certainty of evidence (Brignardello-Petersen and Guyatt, 2025; Phillips et al., 2022). Rankings may therefore exaggerate the apparent superiority of a given intervention and, in turn, lead to inappropriate clinical inferences. For a condition such as MCI, where patient characteristics are heterogeneous and clinically relevant outcomes span multiple domains, treatment rankings alone provide an insufficient basis for robust clinical recommendations.

Accordingly, we conducted a systematic review and network meta-analysis to compare the relative effects of different exercise modalities, delivered alone or in combination with cognitive training, on multidimensional cognitive outcomes in older adults with MCI. In addition to estimating comparative effects within the treatment network, we interpreted the findings using the GRADE or CINeMA framework and prespecified thresholds for clinical importance. This approach allowed us to assess the evidence from complementary perspectives of certainty and clinical relevance, rather than relying on treatment rankings alone. By doing so, we aimed to provide more clinically meaningful evidence to inform exercise prescription and future guideline development for older adults with MCI (Brignardello-Petersen et al., 2020; Izcovich et al., 2023; Johnston et al., 2015; Nikolakopoulou et al., 2020).

## 2 Materials and methods

The conduct and reporting of this systematic review and network meta-analysis followed the PRISMA 2020 statement and the PRISMA extension for network meta-analyses (PRISMA-NMA) (Hutton et al., 2015; Supplementary Table 1). Prior to study conduct, the protocol was registered in the International Prospective Register of Systematic Reviews (PROSPERO; CRD420261372312). All subsequent methodological procedures adhered to the prespecified protocol.

### 2.1 Data sources and search strategy

We searched PubMed, Embase, the Cochrane Library, and Web of Science from database inception to March 1, 2026. Search strategies combined controlled vocabulary with free-text terms for mild cognitive impairment, older adults, exercise-based interventions, cognitive training, and randomized controlled trials. No language restrictions were applied. The full search strategy for each database is provided in Supplementary Table 2.

Search terms covered concepts related to cognitive impairment (e.g., “mild cognitive impairment,” “cognitive dysfunction,” and “cognitive decline”), older age (e.g., “aged,” “older adults,” and “elderly”), exercise-based interventions (e.g., “exercise,” “physical activity,” “aerobic exercise,” “resistance training,” “mind–body exercise,” “tai chi,” “yoga,” “dance,” “exergaming,” and “virtual reality”), cognitive training, and randomized study design (e.g., “randomized controlled trial,” “randomized,” and “RCT”). Search terms and syntax were adapted to the indexing system and search requirements of each database.

To mitigate the risk of incomplete retrieval, we supplemented the database search by screening the reference lists of eligible studies and relevant review articles.

### 2.2 Selection criteria

The eligibility criteria were established in advance using the PICOS framework.

#### 2.2.1 Inclusion criteria

(1) Participants: Studies were eligible if they enrolled older adults aged 60 years or older, consistent with the World Health Organization definition adopted in this review (PAHO, 2026), and included participants diagnosed with mild cognitive impairment (MCI). MCI diagnosis had to be based on established diagnostic criteria or on diagnostic standards explicitly reported by the original investigators (Nasreddine et al., 2005). For studies that reported only cognitive screening scores without a clear diagnostic basis for MCI, eligibility was determined through full-text assessment.

(2) Interventions: Eligible randomized controlled trials evaluated one of the following intervention nodes: aerobic exercise, such as jogging, cycling, or swimming; resistance training, including exercise performed against external loads or elastic resistance; mind–body exercise, such as tai chi, Baduanjin, qigong, or yoga; multicomponent exercise, defined as interventions combining at least two exercise components, such as aerobic exercise and resistance training, resistance and balance training, or aerobic, resistance, and flexibility training; digital or virtual reality–based exercise, including exergaming, motion-sensing training, and immersive or non-immersive virtual reality exercise; cognitive training interventions targeting specific cognitive domains, including memory, attention, and executive function; and combined interventions pairing cognitive training with aerobic exercise, resistance training, mind–body exercise, or multicomponent exercise.

The intervention nodes were classified according to shared theoretical, clinical, and mechanistic characteristics rather than intervention labels alone (Larkey et al., 2009; National Center for Complementary and Integrative Health, 2026a; National Center for Complementary and Integrative Health, 2026b; Zhang et al., 2018). Mind–body exercise was defined within the framework of mind–body practices and meditative movement, which integrates physical movement or postural control with attentional focus, breath regulation, relaxation, body awareness, and cognitive–motor sequencing (Larkey et al., 2009; National Center for Complementary and Integrative Health, 2026a).

Tai chi, Baduanjin, qigong, and yoga were therefore classified as mind–body exercise because they share core features of coordinated movement, regulated breathing, attentional engagement, and meditative or relaxation components (National Center for Complementary and Integrative Health, 2026b; Zhang et al., 2018). Dance-based interventions were included in this node only when they were delivered as structured programs emphasizing rhythmic coordination, movement sequence learning, attentional engagement, emotional expression, and mind–body integration, rather than primarily as conventional aerobic exercise (Huang et al., 2023; Wu et al., 2019).

Multicomponent exercise was retained as a separate intervention node because this category is consistent with established exercise-prescription frameworks for older adults, in which cardiorespiratory, resistance, flexibility, balance, coordination, neuromotor, or functional training components are integrated within a structured program (Bull et al., 2020; Garber et al., 2011).

In the present review, multicomponent exercise was defined as an intervention combining at least two exercise components, such as aerobic, resistance, balance, flexibility, coordination, or functional training. This classification was also supported by previous studies and reviews in older adults with MCI or cognitive frailty, in which multicomponent exercise has been examined as a distinct intervention strategy targeting multidimensional physical, functional, and cognitive outcomes (Luo et al., 2024; Suzuki et al., 2013; Wang et al., 2024).

Digital or virtual reality–based exercise was classified as a separate node because these interventions share a technology-mediated motor–cognitive training framework. Although the specific platforms differed, including exergaming, motion-sensing systems, and immersive or non-immersive virtual reality, these interventions commonly combined physical movement with interactive task performance, visuospatial processing, real-time feedback, attention, inhibition, task switching, and/or dual-task demands (Chan et al., 2024; Liao et al., 2019; Zhao et al., 2020).

Nevertheless, differences in platform type, immersion level, task complexity, feedback mode, exercise intensity, and training dose were considered potential sources of within-node heterogeneity and were therefore taken into account when interpreting pooled estimates (Manser et al., 2024).

(3) Comparators: Comparator conditions included wait-list or usual care controls (WLC), such as usual care, continuation of habitual daily activities, or no structured intervention, and active controls (AC), such as health education, community-based health guidance, or other non-exercise control conditions that were not expected to produce exercise-equivalent effects.

(4) Outcomes: Eligible studies were required to report at least one prespecified outcome and provide sufficient quantitative data for effect-size calculation. Outcomes of interest included the Montreal Cognitive Assessment (MoCA), Mini-Mental State Examination (MMSE), Alzheimer’s Disease Assessment Scale–Cognitive Subscale (ADAS-Cog), Trail Making Test Part B (TMT-B), Digit Span Test (DST), Stroop Color and Word Test (SCWT), depression-related assessments (DRA), and memory function assessments (MFA).

#### 2.2.2 Exclusion criteria

(1) Enrolled mixed populations from which data for participants with MCI could not be identified or extracted separately.

(2) Provided incomplete or insufficiently clear outcome data for quantitative synthesis.

(3) Reviews, case reports, study protocols, editorials, conference abstracts without extractable data, or other non-original articles.

After removal of duplicates, two reviewers independently screened titles and abstracts, and then assessed the full texts of potentially eligible records. Disagreements were resolved by consensus, with consultation of a third reviewer when necessary. When multiple reports described the same trial, the report with the most complete dataset and the most up-to-date results was retained as the primary study. Additional reports were used, where appropriate, to supplement missing data or clarify study details.

### 2.3 Data extraction and quality assessment

Data extraction from the included randomized controlled trials was performed independently by two reviewers. Discrepancies were settled by consensus discussion and, when consensus could not be reached, adjudicated by a third reviewer. The extracted data comprised the first author, year of publication, sample size, participant age, sex distribution, intervention frequency, session duration, intervention period, characteristics of the experimental and control interventions, and outcome data required for the prespecified analyses. To further clarify clinical and methodological heterogeneity, we also extracted exercise intensity, supervision status, adherence or completion rate, level of social interaction, level of cognitive engagement, and intervention dose for variables that were reported or could be calculated.

For continuous outcomes, post-intervention change-from-baseline means and their corresponding standard deviations were preferentially extracted. When the standard deviation of the change score was unavailable, it was derived, where feasible, using the procedures recommended in the Cochrane Handbook. The formulas and data conversion methods applied are detailed in Supplementary Table 3.

The methodological quality of the included studies was appraised using the Cochrane Risk of Bias 2 (RoB 2) tool (Sterne et al., 2019). This instrument assesses risk of bias in randomized controlled trials across five prespecified domains: bias arising from the randomization process, deviations from intended interventions, missing outcome data, outcome measurement, and selective reporting of results. Judgments for each domain, as well as the overall risk-of-bias judgment, were categorized as low risk, some concerns, or high risk. Two reviewers conducted the assessments independently, with disagreements resolved through discussion or, when required, consultation with a third reviewer.

### 2.4 Statistical analysis

Network meta-analyses were implemented in Stata/MP 18.0. For continuous outcomes reported on a common scale and in the same units, treatment effects were expressed as mean differences (MDs) with 95% confidence intervals (CIs). When outcome measures differed across studies, standardized mean differences (SMDs) with 95% CIs were estimated instead. For multi-arm trials, the correlation arising from shared comparator groups was incorporated into the analysis to prevent underestimation of standard errors.

The primary analysis used a random-effects consistency model, with the between-study variance (τ^2^) estimated by restricted maximum likelihood (REML). When closed loops were available, inconsistency was evaluated at both the global and local levels. Local inconsistency was assessed using node-splitting methods, with *P* < 0.05 considered suggestive of potential inconsistency. Loop-specific inconsistency factors were also examined; a 95% confidence interval crossing zero was taken to indicate the absence of statistical evidence for disagreement between direct and indirect estimates. Within-node heterogeneity analyses were additionally conducted for mind–body exercise, multicomponent exercise, and digital or virtual reality–based exercise across outcomes where sufficient data were available.

Network geometry was displayed graphically, with node size weighted by the total sample size for each intervention and edge thickness weighted by the number of direct comparisons. Intervention rankings were characterized using the surface under the cumulative ranking curve (SUCRA), the probability that each intervention ranked first, and the corresponding mean rank. These ranking metrics were interpreted as supplementary indicators and were considered together with effect estimates, 95% confidence intervals, prespecified MID thresholds, and GRADE/CINeMA certainty.

For outcomes informed by at least 10 studies, potential small-study effects and publication bias were evaluated using comparison-adjusted funnel plots. Egger’s regression test was further performed as a supplementary assessment of funnel plot asymmetry when the number of included studies was sufficient. Both funnel plots and Egger’s test were interpreted as exploratory tools rather than definitive methods for excluding reporting bias, particularly in networks with heterogeneous interventions or limited direct comparisons. Leave-one-out analyses and additional sensitivity analyses excluding studies at high risk of bias were conducted to examine the robustness of pooled estimates.

Univariable network meta-regression analyses were conducted to examine whether study-level covariates were associated with estimated treatment effects. These covariates included mean age, intervention frequency, intervention duration, completion rate, supervision status, level of social interaction, level of cognitive engagement, exercise intensity, and intervention dose. Regression coefficients, corresponding 95% confidence intervals, and Wald test *P*-values were reported. Because these analyses used aggregate study-level covariates, the meta-regression findings were interpreted as exploratory.

### 2.5 Certainty of evidence assessment

Evidence certainty for the network meta-analysis was evaluated using the GRADE framework as operationalized by the Confidence in Network Meta-Analysis (CINeMA) approach. Randomized trial evidence was initially rated as high certainty and then judged across six domains: within-study bias, reporting bias, indirectness, imprecision, heterogeneity, and incoherence. Within-study risk of bias was assessed for each trial using RoB 2 and was then summarized at the comparison level according to the relative contribution of each study to the network estimate, as quantified by the CINeMA contribution matrix. Indirectness was assessed by examining whether the assumptions of transitivity and exchangeability were sufficiently met.

Baseline severity and key intervention-related characteristics were considered as potential effect modifiers, including mean age, intervention frequency, intervention duration, completion rate, supervision status, level of social interaction, level of cognitive engagement, exercise intensity, and intervention dose. Direct and indirect bodies of evidence were then compared with respect to participant profiles, intervention and comparator characteristics, and outcome assessment methods.

Imprecision was assessed against prespecified minimal important difference (MID) thresholds. Whenever available, outcome-specific MID/MCID estimates derived from MCI, prodromal cognitive impairment, or closely related older adult populations were prioritized. For outcomes without validated MCI-specific MID estimates, particularly those synthesized as standardized mean differences (SMDs), pragmatic distribution-based thresholds were used to support imprecision judgments rather than to define definitive clinical response thresholds.

This approach is consistent with MID methodology recommending the use of anchor-based estimates when available and distribution-based approaches when instrument-specific MIDs are lacking. Prior methodological studies also suggest that SD-based thresholds can help interpret effect magnitude, although clinically important effects expressed in SMD units are context-dependent and should not be assumed to be universally applicable (Norman et al., 2003; Revicki et al., 2008; Tsujimoto et al., 2022).

For MoCA, an MD of 2 points was used as the threshold for clinical importance. This threshold represents a conservative value within the range reported in previous studies; Lindvall et al. (2024) suggested that the MCID for MoCA may range from 1 to 2 points, depending on the estimation method (Lindvall et al., 2024). For MMSE, an MD of 1.7 points was prespecified as the threshold for a clinically meaningful benefit, based on evidence that meaningful change in MCI generally falls within the range of 1–2 points. Borland et al. reported a triangulated MCID of 1.7 points in prodromal/MCI populations (Borland et al., 2022; Muir et al., 2024). For ADAS-Cog, an MD of −2 points was defined a priori as a small but clinically important benefit, consistent with MCID/MID studies in early Alzheimer’s disease and MCI suggesting that a clinically meaningful change is approximately 2–3 points, with 2 points representing a conservative lower bound (Muir et al., 2024).

For TMT-B, an MD of −20.1 s was prespecified as the threshold for clinical importance, based primarily on the clinically important change reported by Borland et al. in individuals with MCI and its subsequent use in exercise trials involving older adults with MCI (Borland et al., 2022). For DST, an SMD of 0.40 was used as a pragmatic threshold because no outcome-specific MCID has been established for older adults with MCI. For SCWT, a change of 9.3 s was considered clinically meaningful; in the direction of benefit, this was defined as an MD of −9.3 s, based on the triangulated MCID reported by Borland et al. and its later application in exercise trials of older adults with MCI (Borland et al., 2022).

For depression-related assessments, an SMD of −0.30 was prespecified as the threshold for a small but potentially clinically important benefit because depressive symptoms were measured using different instruments. This threshold was informed by MID/MCID methodology studies and further supported by depression-specific methodological work suggesting that clinically relevant SMD thresholds are approximate and may be lower than 0.50, while still requiring cautious interpretation (Cuijpers et al., 2014; Howard et al., 2011; Mouelhi et al., 2020; Tsujimoto et al., 2022; Watt et al., 2021).

For memory function assessments, an SMD of 0.30 was prespecified as a pragmatic threshold of clinical importance, based on distribution-based MID methodology and previous older-adult cognitive intervention research using a 0.3 SD between-group difference as a clinically meaningful target (Mouelhi et al., 2020; Tsujimoto et al., 2022; Vellas et al., 2014; Watt et al., 2021). These SMD-based thresholds were therefore interpreted cautiously as decision thresholds for imprecision assessment rather than as validated MCI-specific MCIDs. Judgments regarding imprecision were based on whether the 95% confidence interval extended across both the line of no effect and the relevant MID threshold. Details are provided in Supplementary Table 4.

Between-study heterogeneity was primarily appraised using the random-effects estimate of τ^2^, alongside consideration of whether the prediction interval crossed the prespecified MID threshold. For networks with closed loops, incoherence was examined within the CINeMA framework by contrasting direct and indirect evidence through node-splitting/SIDE procedures or the design-by-treatment interaction model.

## 3 Results

### 3.1 Study selection and characteristics

**FIGURE 1:**
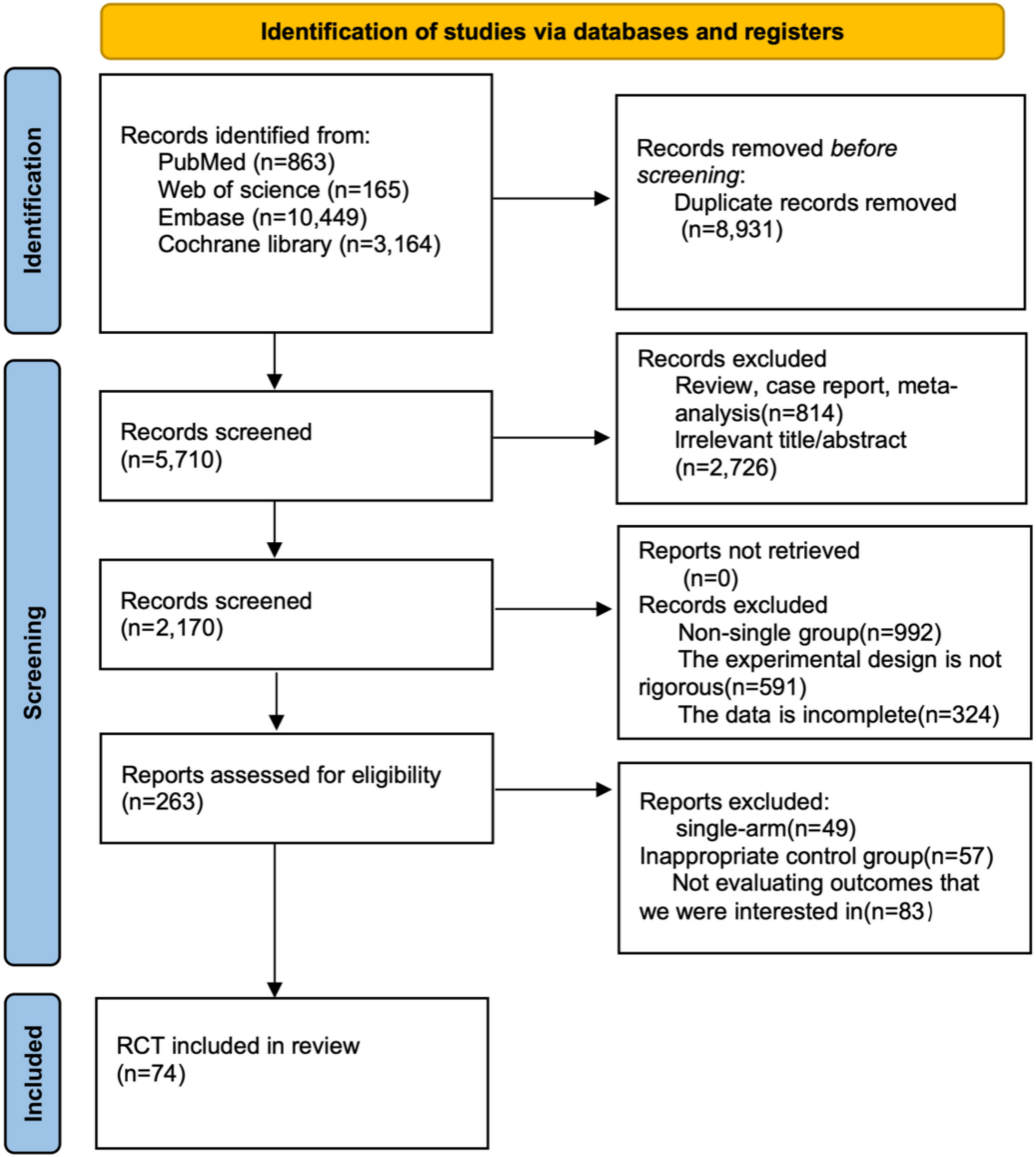
PRISMA flow diagram of study selection.

The literature search identified 14,641 records. After duplicate removal and title/abstract screening, 263 articles underwent full-text assessment. A total of 74 studies satisfied the prespecified eligibility criteria and were included in the final review (Figure 1).

Across the 74 included studies, 5,578 participants were represented, contributing evidence across 12 intervention nodes: aerobic exercise (AE), resistance training (RT), mind–body exercise (MBE), multicomponent exercise (ME), digital or virtual reality–based exercise (D/VRE), cognitive training (CT), aerobic exercise plus cognitive training (AE-CT), resistance training plus cognitive training (RT-CT), mind–body exercise plus cognitive training (MBE-CT), multicomponent exercise plus cognitive training (ME-CT), wait-list or usual care control (WLC), and active control (AC).

Intervention duration varied across intervention nodes, ranging from 4 to 48 weeks. Multicomponent exercise was generally delivered over 24–48 weeks, dance-based interventions over 12–40 weeks, resistance training over 8–24 weeks, yoga over 12–16 weeks, aerobic exercise over 6–48 weeks, tai chi over 12–24 weeks, cognitive training over 4–48 weeks, and digital or virtual reality–based exercise interventions over 8–24 weeks. Participants were predominantly older adults, with mean ages generally ranging from 65 to 78 years, and women accounted for most of the sample.

At baseline, mean scores were 21.26 ± 2.57 for MoCA, 23.69 ± 2.26 for MMSE, 16.53 ± 3.56 for ADAS-Cog, 181.44 ± 56.41 for TMT-B, 8.44 ± 1.63 for DST, 30.42 ± 11.06 for SCWT, 6.69 ± 3.73 for depression-related assessments, and 25.80 ± 8.21 for memory function assessments.

Across the included studies, the mean completion rate was 88.2%. In terms of supervision, 56 studies used fully supervised interventions, 13 used partial supervision, and one was unsupervised; four studies did not report supervision status. Most studies involved moderate-to-high social interaction and high cognitive engagement. After excluding studies with insufficient information on intervention frequency or active exercise duration, as well as non-exercise interventions, moderate intensity was the most frequently prescribed exercise intensity. Among eligible exercise studies, the mean intervention dose was 433.95 MET⋅min/week. Detailed characteristics of all included studies are presented in Supplementary Tables 5–7.

### 3.2 Risk of bias assessment

The RoB 2 tool was applied to evaluate risk of bias across the 74 included randomized controlled trials. Across the included studies, overall RoB 2 judgments indicated low risk of bias in 25 studies, some concerns in 34 studies, and high risk of bias in 15 studies. Overall, these judgments indicate that a substantial proportion of the included studies had at least some risk-of-bias concerns, which may reduce confidence in the robustness of some pooled estimates, particularly for comparisons informed by a small number of trials.

Domain-specific assessments indicated that 15 studies were at high risk of bias arising from the randomization process, and 12 were at high risk because of missing outcome data. Among the 34 studies classified as having some concerns, the main reason for downgrading in 22 studies was bias due to deviations from the intended interventions. Detailed domain-level judgments are presented in Supplementary Figure 1.

### 3.3 Consistency and heterogeneity assessment

For MoCA, MMSE, ADAS-Cog, TMT-B, DST, SCWT, and depression-related assessments, the treatment networks contained closed loops and were therefore assessed for inconsistency. Global tests showed no statistical evidence of inconsistency for any outcome, with all *P* > 0.05 (Supplementary Table 8). Local inconsistency was evaluated through node-splitting procedures and likewise showed no statistically significant inconsistency for any comparison (*P* > 0.05 for all; Supplementary Tables 9–15). Loop-specific analyses further showed that the 95% confidence intervals for all inconsistency factors included zero, indicating the absence of statistically detectable discordance between direct and indirect evidence (Supplementary Tables 16–22). Accordingly, random-effects consistency models were used for the primary analyses of these outcomes.

Because the network for memory function assessments (MFA) contained no closed loops, global inconsistency tests, local node-splitting analyses, and loop-specific inconsistency assessments could not be performed. MFA was therefore analyzed using a random-effects consistency model, with interpretation relying primarily on network geometry and the plausibility of the transitivity assumption. As shown in Supplementary Tables 5–7, the included studies were broadly comparable in key baseline and design characteristics, including age, intervention frequency, and intervention duration. Nevertheless, because no closed loops were available, disagreement between direct and indirect evidence could not be formally tested. The certainty of evidence for this outcome was therefore interpreted with caution, and downgrading was applied where appropriate.

The primary measure used to quantify between-study heterogeneity was the between-study variance (τ^2^) estimated under the random-effects model. Appreciable heterogeneity was observed for MoCA (τ^2^ = 1.4089562) and MMSE (τ^2^ = 1.0651807). TMT-B also showed substantial heterogeneity (τ^2^ = 41.982295); however, this estimate should be interpreted in relation to the measurement scale, because TMT-B is a time-based outcome and its τ^2^ is not directly comparable in absolute terms with estimates from other continuous measures. In contrast, heterogeneity for memory function assessments was low to moderate (τ^2^ = 0.061795), whereas DST and depression-related assessments showed minimal to low between-study heterogeneity, with τ^2^ values close to zero for DRA and low for DST. Further details are provided in Supplementary Table 23.

Additional within-node heterogeneity analyses are summarized in Supplementary Table 24. For D/VRE, within-node heterogeneity could not be estimated reliably because sufficient outcome-specific direct comparisons were not available; therefore, the within-node heterogeneity results primarily involved MBE and ME. Substantial heterogeneity was mainly observed for mind–body exercise in MoCA (*I*^2^ = 84.51%) and multicomponent exercise in MMSE (*I*^2^ = 93.23%), while mind–body exercise in TMT-B also showed relatively high heterogeneity (*I*^2^ = 66.59%).

In contrast, several other comparisons showed low or no heterogeneity, such as multicomponent exercise in MoCA and ADAS-Cog, mind–body exercise in MMSE and depression-related assessments, and multicomponent exercise in TMT-B. These findings suggest that within-node variability was outcome-dependent rather than uniformly high across the broad intervention nodes, and therefore the corresponding pooled estimates should be interpreted with consideration of the observed heterogeneity level.

### 3.4 Global cognition outcomes

#### 3.4.1 Montreal Cognitive Assessment (MoCA)

**FIGURE 2:**
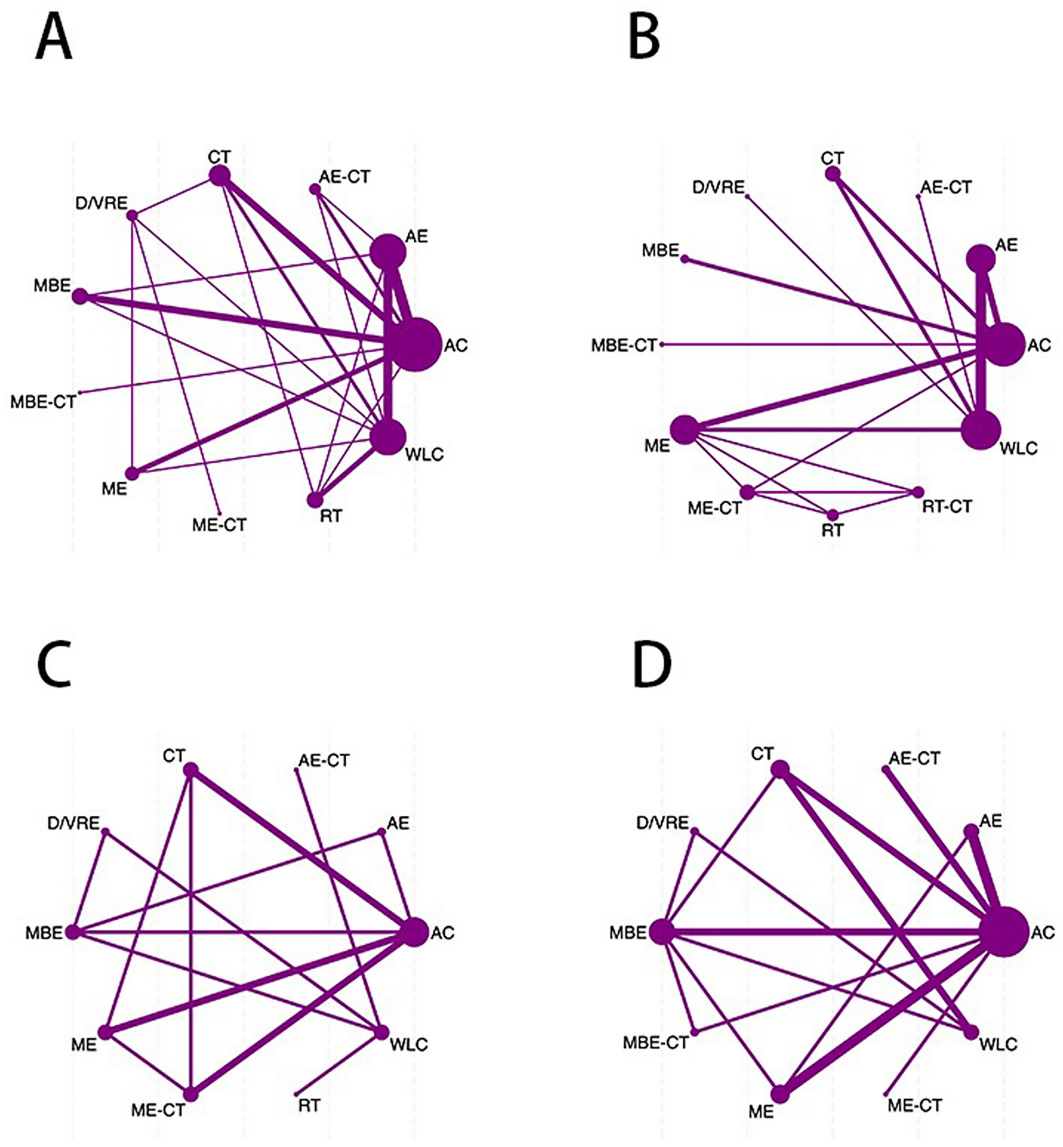
Network plots of exercise-related intervention comparisons for four cognitive outcomes in older adults with mild cognitive impairment: **(A)** MoCA; **(B)** MMSE; **(C)** ADAS-Cog; **(D)** TMT-B.

**FIGURE 3:**
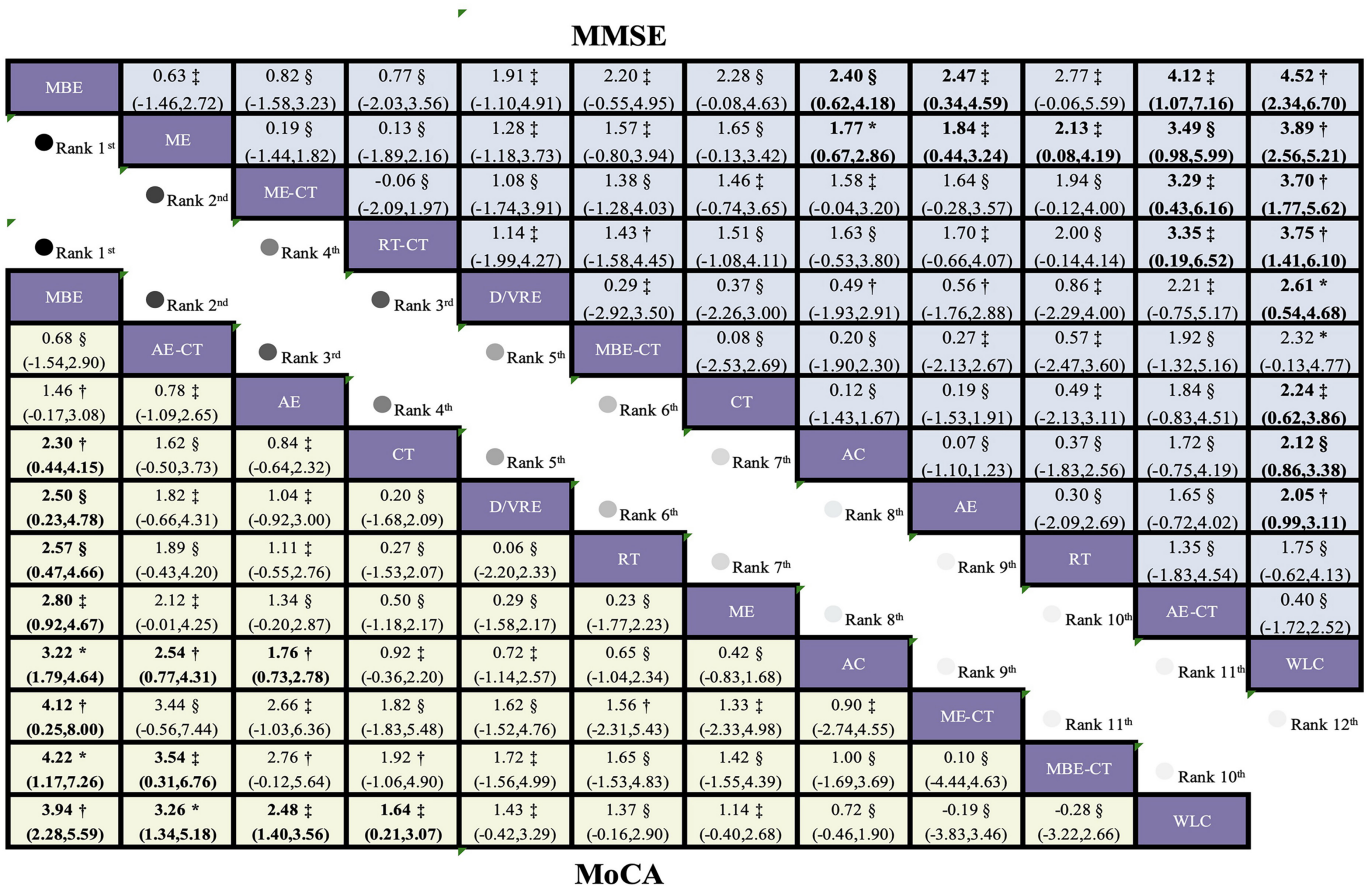
League table from the network meta-analysis comparing intervention nodes in older adults with mild cognitive impairment. Mean differences (MDs) with 95% confidence intervals (CIs) are presented for MoCA in the lower-left triangle and MMSE in the upper-right triangle. Symbols denote certainty of evidence according to the GRADE approach: * high; † moderate; ‡ low; § very low. AE, aerobic exercise; RT, resistance training; MBE, mind–body exercise; ME, multicomponent exercise; D/VRE, digital or virtual reality–based exercise; CT, cognitive training; AE-CT, aerobic exercise plus cognitive training; RT-CT, resistance training plus cognitive training; MBE-CT, mind–body exercise plus cognitive training; ME-CT, multicomponent exercise plus cognitive training.

For the MoCA outcome, the network included 34 studies, 1,947 participants, and 11 intervention nodes (Figure 2). Compared with wait-list/usual care control, mind–body exercise (MBE) was associated with higher MoCA scores (MD = 3.94, 95% CI 2.28–5.59; moderate-certainty evidence), as was aerobic exercise plus cognitive training (AE-CT) (MD = 3.26, 95% CI 1.34–5.18; high-certainty evidence) (Figure 3).

In the descriptive ranking analysis, MBE had the largest SUCRA value (96.3%), followed by AE-CT (87.9%) and aerobic exercise (AE; 77.0%). MBE also had the highest probability of being ranked first (69.3%) and the lowest mean rank (1.4), followed by AE-CT (mean rank, 2.2) and AE (mean rank, 3.3) (Supplementary Figure 6 and Supplementary Table 25).

#### 3.4.2 Mini-Mental State Examination (MMSE)

For MMSE, the treatment network included 24 studies, 1,879 participants, and 12 intervention nodes (Figure 2). Compared with wait-list/usual care control, moderate-certainty evidence showed that mind–body exercise (MBE) was associated with higher MMSE scores (MD = 4.52, 95% CI 2.34–6.70), as was multicomponent exercise (ME) (MD = 3.89, 95% CI 2.56–5.21) (Figure 3).

In the descriptive ranking analysis, MBE had the largest SUCRA value (90.7%), followed by ME (82.7%) and multicomponent exercise plus cognitive training (ME-CT; 77.7%). MBE also had the highest probability of being ranked first (53.9%) and the lowest mean rank (2.0), followed by ME (mean rank, 2.9) and ME-CT (mean rank, 3.5) (Supplementary Figure 7 and Supplementary Table 26).

#### 3.4.3 Alzheimer’s Disease Assessment Scale–Cognitive Subscale (ADAS-Cog)

**FIGURE 4:**
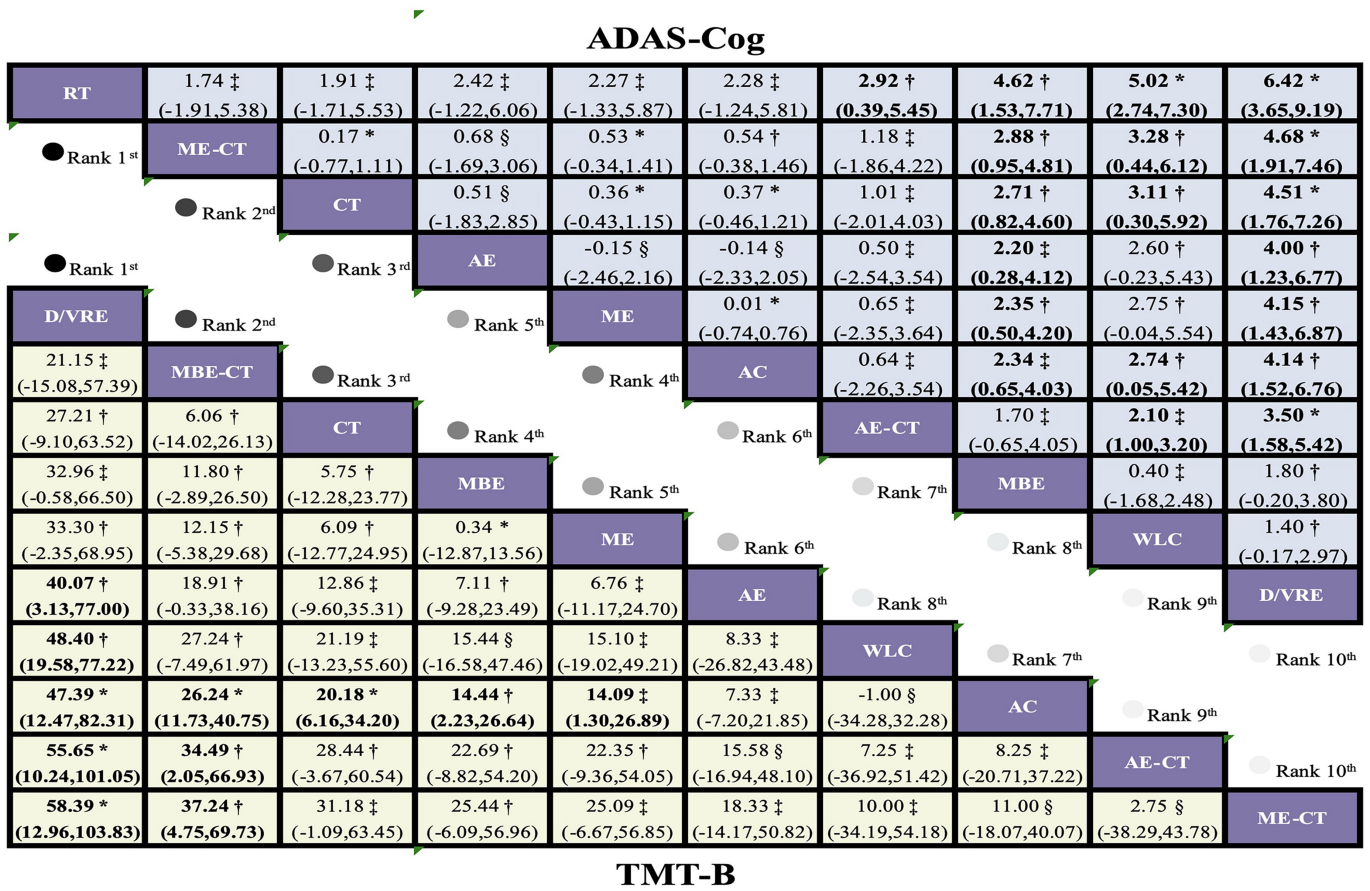
League table from the network meta-analysis comparing intervention nodes in older adults with mild cognitive impairment. Mean differences (MDs) with 95% confidence intervals (CIs) are presented for TMT-B in the lower-left triangle and ADAS-Cog in the upper-right triangle. Symbols denote certainty of evidence according to the GRADE approach: * high; † moderate; ‡ low; § very low. AE, aerobic exercise; RT, resistance training; MBE, mind–body exercise; ME, multicomponent exercise; D/VRE, digital or virtual reality–based exercise; CT, cognitive training; AE-CT, aerobic exercise plus cognitive training; RT-CT, resistance training plus cognitive training; MBE-CT, mind–body exercise plus cognitive training; ME-CT, multicomponent exercise plus cognitive training.

For ADAS-Cog, the treatment network included 8 studies, 1,003 participants, and 10 intervention nodes (Figure 2). Compared with wait-list/usual care control, resistance training (RT) was associated with lower ADAS-Cog scores (MD = −5.02, 95% CI −7.30 to −2.74; high-certainty evidence), as was multicomponent exercise plus cognitive training (ME-CT) (MD = −3.28, 95% CI −6.12 to −0.44; moderate-certainty evidence) (Figure 4).

In the descriptive ranking analysis, RT had the largest SUCRA value (92.7%), followed by ME-CT (78.3%) and cognitive training (CT; 72.7%). RT also had the highest probability of being ranked first (77.9%) and the lowest mean rank (1.7), followed by ME-CT (mean rank, 3.0) and CT (mean rank, 3.5) (Supplementary Figure 8 and Supplementary Table 27).

### 3.5 Executive function and attention/working memory outcomes

#### 3.5.1 Trail Making Test Part B (TMT-B)

For TMT-B, the network included 17 studies, 918 participants, and 10 intervention nodes (Figure 2). Compared with wait-list/usual care control, digital or virtual reality–based exercise (D/VRE) was associated with faster TMT-B performance, as reflected by lower completion time (MD = −48.40, 95% CI −77.22 to −19.58; moderate-certainty evidence). Mind–body exercise plus cognitive training (MBE-CT) was also associated with a numerically lower TMT-B completion time, but the confidence interval included the null (MD = −27.24, 95% CI −61.97 to 7.49) (Figure 4).

In the descriptive ranking analysis, D/VRE had the largest SUCRA value (96.7%), followed by MBE-CT (84.1%) and cognitive training (CT; 72.6%). D/VRE also had the highest probability of being ranked first (85.3%) and the lowest mean rank (1.3), followed by MBE-CT (mean rank, 2.4) and CT (mean rank, 3.5) (Supplementary Figure 9 and Supplementary Table 28).

#### 3.5.2 Digit Span Test (DST)

**FIGURE 5:**
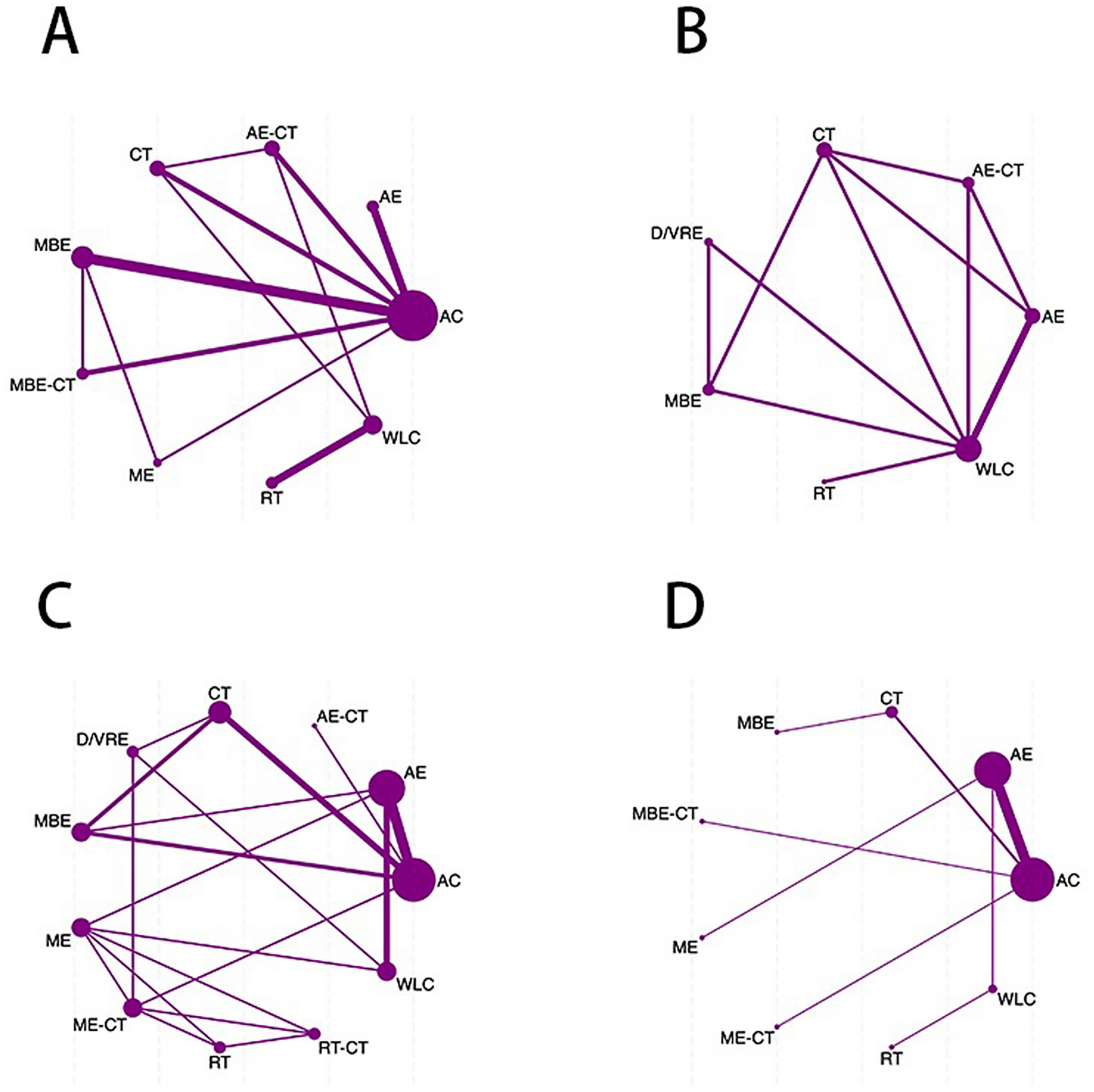
Network plots of exercise-related intervention comparisons for four cognitive outcomes in older adults with mild cognitive impairment: **(A)** DST; **(B)** SCWT; **(C)** DRA; **(D)** MFA.

**FIGURE 6:**
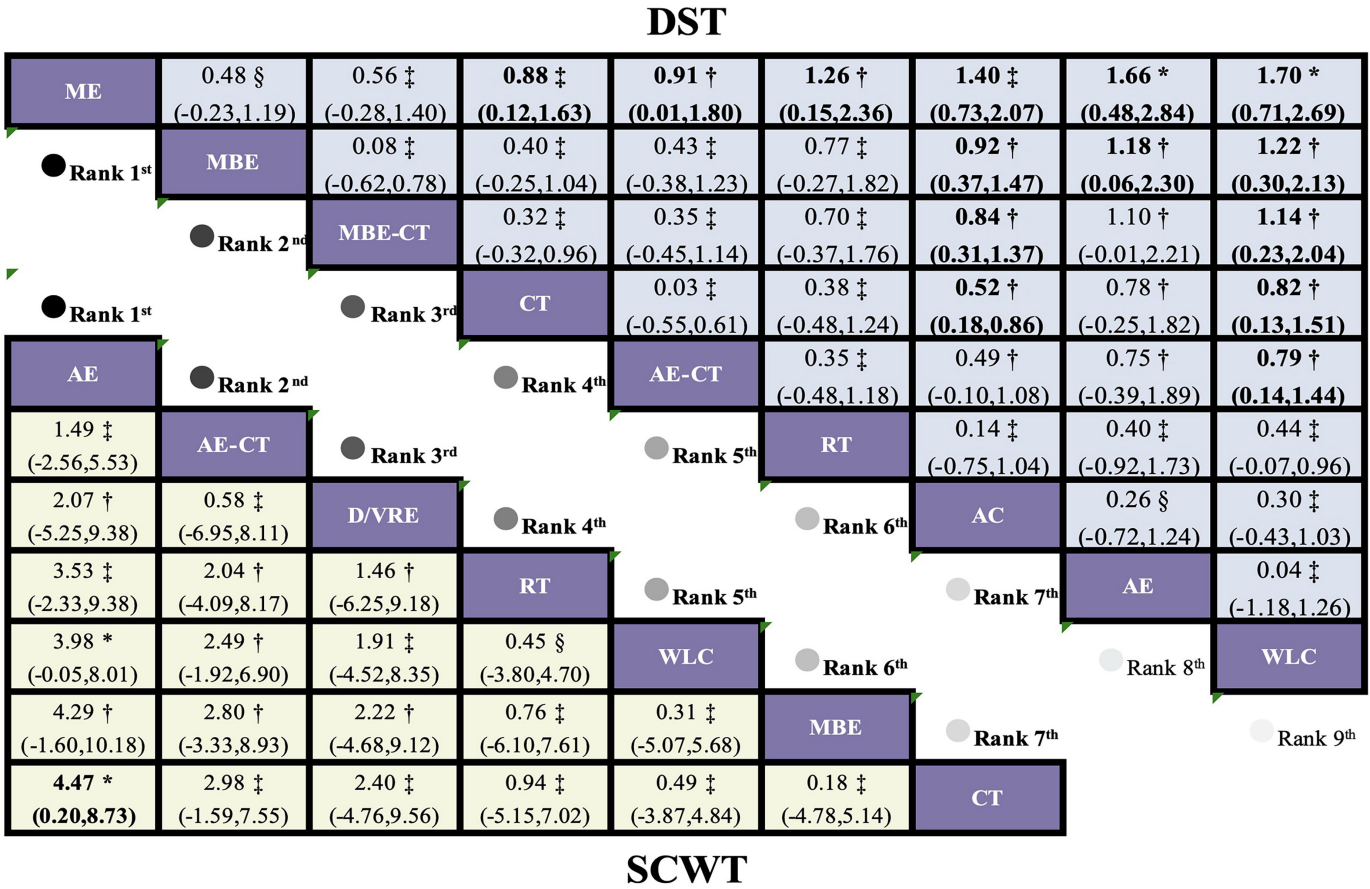
League table from the network meta-analysis comparing intervention nodes in older adults with mild cognitive impairment. Mean differences (MDs) with 95% confidence intervals (CIs) are presented for SCWT in the lower-left triangle, and standardized mean differences (SMDs) with 95% CIs are presented for DST in the upper-right triangle. Symbols denote certainty of evidence according to the GRADE approach: * high; † moderate; ‡ low; § very low. AE, aerobic exercise; RT, resistance training; MBE, mind–body exercise; ME, multicomponent exercise; D/VRE, digital or virtual reality–based exercise; CT, cognitive training; AE-CT, aerobic exercise plus cognitive training; RT-CT, resistance training plus cognitive training; MBE-CT, mind–body exercise plus cognitive training; ME-CT, multicomponent exercise plus cognitive training.

For DST, the treatment network included 16 studies, 818 participants, and 9 intervention nodes (Figure 5). Compared with wait-list/usual care control, multicomponent exercise (ME) was associated with better DST performance (SMD = 1.70, 95% CI 0.71–2.69; high-certainty evidence), as was mind–body exercise (MBE) (SMD = 1.22, 95% CI 0.30–2.13; moderate-certainty evidence) (Figure 6).

In the descriptive ranking analysis, ME had the largest SUCRA value (97.1%), followed by MBE (78.9%) and mind–body exercise plus cognitive training (MBE-CT; 75.1%). ME also had the highest probability of being ranked first (83.3%) and the lowest mean rank (1.2), followed by MBE (mean rank, 2.7) and MBE-CT (mean rank, 3.0) (Supplementary Figure 10 and Supplementary Table 29).

#### 3.5.3 Stroop Color and Word Test (SCWT)

For SCWT, the treatment network included 5 studies, 266 participants, and 7 intervention nodes (Figure 5). Compared with wait-list/usual care control, aerobic exercise (AE) was associated with a numerically favorable effect on SCWT performance (MD = 3.98, 95% CI −0.05 to 8.01; high-certainty evidence), as was aerobic exercise plus cognitive training (AE-CT) (MD = 2.49, 95% CI −1.92 to 6.90; moderate-certainty evidence). However, the 95% CIs for both comparisons crossed the null, indicating no statistically significant difference (Figure 6).

In the descriptive ranking analysis, AE had the largest SUCRA value (87.2%), followed by AE-CT (67.9%). AE also had the highest probability of being ranked first (51.0%) and the lowest mean rank (1.8), followed by AE-CT (mean rank, 2.9) (Supplementary Figure 11 and Supplementary Table 30).

#### 3.6 Depression-Related Assessments (DRA)

**FIGURE 7:**
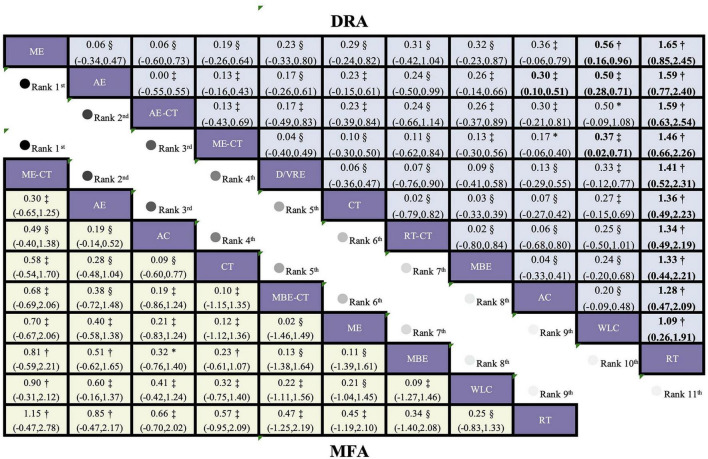
League table from the network meta-analysis comparing intervention nodes in older adults with mild cognitive impairment. Standardized mean differences (SMDs) with 95% confidence intervals (CIs) are presented for MFA in the lower-left triangle and for DRA in the upper-right triangle. Symbols denote certainty of evidence according to the GRADE approach: * high; † moderate; ‡ low; § very low. AE, aerobic exercise; RT, resistance training; MBE, mind–body exercise; ME, multicomponent exercise; D/VRE, digital or virtual reality–based exercise; CT, cognitive training; AE-CT, aerobic exercise plus cognitive training; RT-CT, resistance training plus cognitive training; MBE-CT, mind–body exercise plus cognitive training; ME-CT, multicomponent exercise plus cognitive training.

For depression-related assessments, the network included 22 studies, 1,389 participants, and 11 intervention nodes (Figure 5). Compared with wait-list/usual care control, multicomponent exercise (ME) was associated with lower depression-related scores (SMD = −0.56, 95% CI −0.96 to −0.16; moderate-certainty evidence), as was aerobic exercise (AE) (SMD = −0.50, 95% CI −0.71 to −0.28; low-certainty evidence) (Figure 7).

In the descriptive ranking analysis, ME had the largest SUCRA value (82.7%), followed by AE (79.8%) and aerobic exercise plus cognitive training (AE-CT; 73.6%). ME also had the highest probability of being ranked first (34.5%), followed by AE-CT (29.4%). Mean ranks showed a similar pattern, with ME having the lowest mean rank (2.7), followed by AE (3.0) and AE-CT (3.6) (Supplementary Figure 12 and Supplementary Table 31).

### 3.7 Memory Function Assessments (MFA)

For memory function assessments, the network included 14 studies, 661 participants, and 9 intervention nodes (Figure 5). Compared with wait-list/usual care control, multicomponent exercise plus cognitive training (ME-CT) showed a numerically favorable but statistically uncertain effect on memory function (SMD = 0.90, 95% CI −0.31 to 2.12; moderate-certainty evidence). Aerobic exercise (AE) showed a similar pattern, with a smaller and statistically uncertain effect estimate (SMD = 0.60, 95% CI −0.16 to 1.37; low-certainty evidence) (Figure 7).

In the descriptive ranking analysis, ME-CT had the largest SUCRA value (85.5%), followed by AE (76.2%). ME-CT also had the highest probability of being ranked first (57.2%) and the lowest mean rank (2.2), followed by AE (mean rank, 2.9) (Supplementary Figure 13 and Supplementary Table 32).

### 3.8 Meta-regression, sensitivity analyses, and publication bias

Key baseline characteristics were broadly comparable across treatment groups, with no evident major imbalances. Univariable network meta-regression analyses showed no consistent evidence that the examined study-level covariates, including mean age, intervention frequency, intervention duration, completion rate, supervision status, social interaction, cognitive engagement, exercise intensity, and intervention dose, materially modified treatment effects across outcomes.

These findings provided no clear indication that the examined covariates violated the transitivity assumption; however, the results should be interpreted cautiously because the analyses were exploratory and some intervention-related variables were incompletely reported across trials (Supplementary Tables 33–86).

Leave-one-out sensitivity analyses were performed for MoCA, MMSE, TMT-B, DST, depression-related assessments, and memory function assessments, to assess the influence of each individual study on the corresponding network estimates.

Across outcomes, sequential omission of single studies did not materially change the direction of the estimated effects for exercise interventions or cognitive training versus wait-list/usual care control. Effect-size changes were modest, confidence intervals largely overlapped, and conclusions regarding statistical significance remained essentially unchanged. These findings provided some support for the robustness of the primary analyses to the omission of individual studies (Supplementary Tables 87–92).

Further sensitivity analyses were undertaken after removal of studies classified as high risk of bias according to the RoB 2 assessment. Across outcomes, intervention rankings, effect directions, and the statistical significance of pairwise comparisons remained largely unchanged, suggesting that the findings were not materially influenced by studies at high risk of bias.

However, because a considerable number of studies were still judged as having some concerns, residual within-study bias cannot be fully excluded and should be considered when interpreting the pooled estimates (Supplementary Tables 93–100 and Supplementary Figures 2–5).

For outcomes with more than 10 studies—MoCA, MMSE, TMT-B, DST, depression-related assessments, and memory function assessments—comparison-adjusted funnel plots were generated to assess whether the evidence showed indications of small-study effects or publication bias. Visual inspection of the comparison-adjusted funnel plots showed approximate symmetry, with no pronounced systematic asymmetry or prominent outliers (Supplementary Figures 14–19).

Egger’s regression tests were further conducted for outcomes with a sufficient number of studies, including MoCA, MMSE, TMT-B, DST, depression-related assessments, and memory function assessments. The results did not indicate statistically significant funnel plot asymmetry for these outcomes (all *P* > 0.05; Supplementary Table 101).

Nevertheless, given the heterogeneous nature of the exercise networks and the limited amount of direct evidence for several comparisons, reporting bias or small-study effects could not be definitively excluded.

### 3.9 Certainty of evidence

Certainty of evidence for the network estimates was assessed within the GRADE framework using CINeMA (Supplementary Tables 102–109). Across the eight outcome networks, 29 estimates were rated as high certainty, 90 as moderate certainty, 135 as low certainty, and 105 as very low certainty.

Higher-certainty estimates were generally supported by multiple direct comparisons and had confidence intervals that crossed neither the null effect nor the prespecified MID threshold. Estimates rated as low or very low certainty typically came from sparse networks or relied largely on indirect evidence, often with wide confidence intervals and possible small-study effects.

Therefore, high SUCRA rankings were not interpreted as definitive evidence of intervention superiority when the corresponding estimates were imprecise, derived from sparse networks, or supported by moderate, low, or very low certainty of evidence.

## 4 Discussion

### 4.1 Main findings

In this network meta-analysis of 74 randomized controlled trials including 5,578 participants, several exercise-based interventions showed favorable estimates for specific cognitive and psychological outcomes in older adults with mild cognitive impairment.

Mind–body exercise (MBE) and resistance training (RT) were associated with favorable estimates for global cognitive outcomes, whereas digital or virtual reality–based exercise (D/VRE) and multicomponent exercise (ME) showed favorable estimates for executive function and attention/working memory. Multicomponent exercise (ME) was also associated with a favorable estimate for depression-related outcomes. For memory function, multicomponent exercise combined with cognitive training (ME-CT) showed a favorable but less certain signal. Overall, these findings should be interpreted as domain-specific comparative signals rather than definitive evidence that any single intervention is superior across outcomes.

### 4.2 Interpretation of findings

Our findings suggest that MBE and RT were associated with more favorable estimates for global cognitive outcomes in older adults with MCI. These associations may reflect partly distinct mechanisms, although the underlying pathways remain to be clarified.

MBE combines motor control, postural regulation, attentional engagement, sequential memory, and breath regulation, thereby engaging cognition through sustained cognitive–motor coupling. It may also support cognitive function through neurotrophic signaling and changes in functional brain network organization (Jasim et al., 2023; Sungkarat et al., 2018). By contrast, the apparent benefit of RT may be related to improvements in muscle strength, functional reserve, and neuroplasticity. Previous studies indicate that RT can enhance functional brain plasticity in people with MCI and may help preserve brain regions closely involved in cognitive performance, including the hippocampus and precuneus (Nagamatsu et al., 2012; Ribeiro et al., 2025).

Digital or virtual reality–based exercise (D/VRE), multicomponent exercise (ME), and aerobic exercise (AE) showed more favorable profiles for executive function and attention/working memory. Although these interventions may operate through different mechanisms, each may strengthen neural and behavioral processes involved in prefrontal cognitive control. D/VRE is not simply exercise or cognitive training in isolation; rather, it combines whole-body movement, spatial navigation, target selection, response inhibition, task switching, and real-time feedback within a single training environment.

By creating an immersive, multisensory, and feedback-rich context, D/VRE may be hypothesized to engage prefrontal networks and improve training efficiency, although this mechanism was not directly examined in the present analysis. Some studies have reported improved cognitive performance after VR-based training despite reduced prefrontal activation, a pattern that may indicate greater neural efficiency (Liao et al., 2019; Liao et al., 2020).

ME combines aerobic, resistance, balance, and coordination components, and may support executive function through broader sensorimotor and cognitive stimulation (Bae et al., 2019; Suzuki et al., 2013). The relative benefit of AE may be more closely linked to neurovascular and metabolic pathways, including improved cerebral perfusion, arousal regulation, and processing speed, which may in turn support sustained attention and working memory (Chen et al., 2024; Shu et al., 2025).

Multicomponent exercise (ME) appeared to be among the more favorable interventions for depression-related outcomes in older adults with MCI, whereas multicomponent exercise combined with cognitive training (ME-CT) showed a more favorable signal for memory function. These findings may reflect different mechanistic pathways. For depression-related outcomes, ME integrates aerobic, resistance, balance, and functional training, and may reduce depressive symptoms indirectly by improving physical fitness, attenuating frailty, increasing daily activity, and promoting social engagement. Its effects may therefore extend beyond mood to broader gains in everyday functioning and quality of life (Liu et al., 2023; Luo et al., 2024).

For memory outcomes, ME-CT may provide wide-ranging sensorimotor and neural stimulation through multicomponent exercise, while the cognitive training component offers targeted practice in encoding, retrieval, and memory strategies. This combination may generate additive or synergistic effects, but this interpretation remains hypothesis-generating (Castellote-Caballero et al., 2024; Muñoz-Perete et al., 2025).

In this interpretation, ME may act primarily through a functional–emotional pathway, whereas ME-CT may influence memory through a dual pathway that combines exercise-induced neurocognitive stimulation with structured cognitive practice. These explanations remain inferential and were not directly tested in the present study. Future trials should examine these mechanisms more explicitly using neuroimaging, biomarker-based measures, and adequately powered head-to-head randomized designs.

### 4.3 Implications for clinical practice and research

These findings reinforce the potential role of exercise in the management of mild cognitive impairment and suggest that it may be considered an important non-pharmacological component of care, rather than merely a supportive adjunct. Although current clinical guidelines provide limited direction on the choice of specific exercise modalities, our comparative findings may help inform the tailoring of exercise recommendations to distinct cognitive and affective outcome domains, while taking into account evidence certainty, patient preferences, functional status, accessibility, and safety considerations.

From a practical prescription perspective, these findings should not be interpreted as supporting a single fixed exercise protocol for all older adults with MCI. Rather, exercise modality should be selected alongside key prescription variables, including intensity, progression, supervision, adherence, and individual tolerance. For most older adults with MCI, programs may need to begin at a tolerable low-to-moderate intensity and progress gradually in duration, frequency, resistance load, movement complexity, or cognitive–motor task demands according to functional capacity and safety. Supervision may be particularly important during resistance training, multicomponent exercise, and digital or virtual reality–based exercise, where technique, balance challenge, equipment use, and dual-task demands may affect both safety and adherence. Attendance, completion rate, and patient preference should also be considered when translating these findings into clinical or community-based exercise recommendations.

For global cognitive outcomes, mind–body exercise modalities such as tai chi, Baduanjin, and yoga, as well as low- to moderate-intensity resistance training using elastic bands or machines, may be reasonable options for some older adults with MCI, particularly when programs are introduced progressively and matched to individual functional capacity, preferences, and safety considerations. These interventions are generally accessible, low cost, and well tolerated, and they can often be delivered in group-based settings, making them practical options for routine clinical and community implementation.

For executive function and attention/working memory, exergaming, immersive or non-immersive virtual reality–based exercise, and multicomponent programs combining aerobic, resistance, and balance training appear promising, but their use should be considered within individualized care plans and in light of accessibility, supervision, and safety requirements. For depression-related outcomes and memory function, multicomponent exercise, with or without cognitive training, may have clinical relevance, although the strength of evidence differs across outcomes. Programs that integrate resistance-based exercise, aerobic activity, balance, coordination, and functional training may offer broader therapeutic benefits, whereas the addition of cognitive training may be especially valuable when memory improvement is a primary treatment goal.

### 4.4 Comparison with previous studies

A key difference between this study and earlier reviews is the study population. We restricted inclusion to older adults with mild cognitive impairment and did not pool participants with dementia or other stages of cognitive impairment. Compared with previous network meta-analyses that combined MCI and dementia populations, our sample was more clinically homogeneous and better suited to estimating intervention effects specifically in MCI (Ali et al., 2022; Huang et al., 2022; Tao et al., 2023). Differences between our findings and those of earlier studies regarding the most favorable intervention types may therefore reflect, at least in part, differences in the populations included.

Methodologically, much of the earlier literature has relied on conventional pairwise meta-analysis, usually estimating the overall effect of exercise or cognitive training on a single outcome. In contrast, we used network meta-analysis to integrate direct and indirect evidence and compare multiple interventions within a single analytic framework. We also interpreted treatment rankings alongside GRADE-based certainty assessments, emphasizing clinical interpretation based on both relative effects and certainty of evidence rather than rankings alone (Ahn and Kim, 2023; Meng et al., 2022).

Another important distinction lies in the way interventions were classified. In this study, active interventions were separated into 10 nodes: aerobic exercise, resistance training, multicomponent exercise, mind–body exercise, cognitive training, aerobic exercise plus cognitive training, resistance training plus cognitive training, multicomponent exercise plus cognitive training, mind–body exercise plus cognitive training, and digital or virtual reality–based exercise. In contrast, many previous reviews have treated “exercise” as a single broad category or have used coarser classifications, such as aerobic exercise, combined exercise, traditional mind–body exercise, and combined interventions. This difference in classification granularity may partly explain why our conclusions do not fully align with previous reports regarding the ranking of specific intervention types. In particular, separating combined interventions into more clinically distinct categories—such as aerobic exercise plus cognitive training, multicomponent exercise plus cognitive training, and mind–body exercise plus cognitive training—made it possible to identify outcome-specific patterns of benefit that may have been obscured in broader classifications (Ahn and Kim, 2023; Jeong et al., 2025; Meng et al., 2022; Wang et al., 2024).

The range of outcomes assessed also distinguishes this review from prior work. We examined eight outcomes—MoCA, MMSE, ADAS-Cog, TMT-B, DST, SCWT, depression-related outcomes, and memory function—covering global cognition, executive function and attention/working memory, mood, and memory. By contrast, several earlier reviews focused mainly on global cognition or on a single cognitive domain. Some addressed broader cognitive and psychological outcomes, others focused specifically on executive function, and others examined combined physical–cognitive interventions primarily in relation to memory and executive performance. This broader outcome framework may explain why our analysis was able to show that no single intervention was consistently superior across all domains (Ahn and Kim, 2023; Chen et al., 2024; Jeong et al., 2025; Meng et al., 2022).

### 4.5 Strengths and limitations

Several strengths of this study should be highlighted. First, it was conducted within a rigorous methodological framework, including RoB 2 for risk-of-bias assessment and GRADE-based evaluation of the certainty of evidence. Leave-one-out sensitivity analyses and additional analyses excluding studies at high risk of bias were also performed, providing a more cautious assessment of whether the primary findings were influenced by individual studies or by trials with greater methodological limitations. Second, key outcomes were interpreted against prespecified MID/MCID thresholds within an explicit evidence-grading framework, enhancing the clinical interpretability of the results and the transparency of evidence appraisal. Third, exercise interventions were classified into 10 active nodes, and eight outcomes were assessed across four clinically relevant domains: global cognition, executive function and attention/working memory, mood, and memory. This more granular analytic structure enabled more precise comparisons across intervention types and improved the clinical relevance of the findings.

Despite these strengths, several limitations should be acknowledged. Blinding participants and intervention personnel is inherently challenging in exercise trials, and this likely contributed to the number of studies judged as having some concerns or a high risk of bias. The relatively high proportion of studies with non-low risk-of-bias judgments may have affected the robustness of some findings by increasing the possibility of performance bias, attrition bias, or bias related to deviations from intended interventions. Although sensitivity analyses excluding studies at high risk of bias showed broadly consistent effect directions and rankings, these analyses cannot fully rule out the influence of studies with some concerns. Therefore, findings based on sparse networks, indirect evidence, or moderate-to-low certainty should be interpreted cautiously. In addition, exercise prescription variables, including intensity, progression, supervision, adherence, and intervention dose, were not consistently reported across trials, which limited our ability to derive more precise modality-specific prescription recommendations.

The MFA network also contained no closed loops; therefore, disagreement between direct and indirect evidence could not be formally evaluated. We accounted for this limitation by downgrading for indirectness where appropriate in the GRADE assessment, but the corresponding findings should still be interpreted cautiously. Finally, publication bias and small-study effects remain possible. Because fewer than 10 studies contributed to the ADAS-Cog and SCWT networks, these biases could not be assessed reliably for those outcomes. Rankings for ADAS-Cog and SCWT should therefore be interpreted alongside relative effect estimates and certainty of evidence, rather than as stand-alone indicators of comparative benefit.

## 5 Conclusion

This systematic review and network meta-analysis suggests that exercise interventions may be associated with favorable cognitive and psychological outcomes in older adults with mild cognitive impairment, although effects varied by intervention modality, outcome domain, and certainty of evidence. Mind–body exercise showed favorable estimates for global cognition, resistance training showed favorable results for ADAS-Cog performance, and digital or virtual reality–based exercise showed a favorable signal for executive function. Multicomponent exercise was associated with encouraging results for working memory and depression-related outcomes, whereas multicomponent exercise combined with cognitive training showed a favorable but less certain signal for memory function.

These findings support exercise as an important non-pharmacological component of MCI management. However, the certainty of evidence remains limited for several comparisons. Large, well-designed head-to-head trials, together with greater standardization of intervention protocols and outcome measures, are needed to strengthen the evidence base and inform future clinical recommendations and guideline updates.

## Data Availability

The original contributions presented in this study are included in the article/Supplementary material, further inquiries can be directed to the corresponding author.
