# Supplementary material for: Comparative efficacy and certainty of evidence of exercise interventions for cognitive outcomes in older adults with mild cognitive impairment: a systematic review and network meta-analysis of randomized controlled trials
Source: Front Aging Neurosci. 2026 Jul 7;18:1866613. doi: 10.3389/fnagi.2026.1866613 (PMC13385057; doi:10.3389/fnagi.2026.1866613)

Supplementary Materials for

Comparative Efficacy and Certainty of Evidence of Exercise Interventions for Cognitive Outcomes in Older Adults With Mild Cognitive Impairment: A Systematic Review and Network Meta-Analysis of Randomized Controlled Trials

Table of Contents

Title	Content	page
Table 1	PRISMA NMA Checklist of Items to Include When Reporting a Systematic Review Involving a Network Meta-analysis	9-10
Table 2	Literature Search Strategy	13-18
Table 3	Pre-transformation data, calculation formulas, and data formats	19-20
Table 4	Prespecified Minimal Important Differences (MIDs) and Their Rationale for Imprecision Judgement Across Outcomes	21
Table 5	Characteristics of the included studies	22-31
Table 6	Intervention-related and methodological characteristics of the included studies.	32-37
	REFERENCES	38-43
Table 7	Baseline characteristics of the included outcome measures	44-47
Table 8	Global inconsistency table for MoCA、MMSE、ADAS-Cog、TMT-B、DST、SCWT and DRA outcomes in older adults with mild cognitive impairment	48
Table 9	Node-splitting analysis for the MoCA outcome in older adults with mild cognitive impairment	48-49
Table 10	Node-splitting analysis for the MMSE outcome in older adults with mild cognitive impairment	49-50
Table 11	Node-splitting analysis for the ADAS-Cog outcome in older adults with mild cognitive impairment	50
Table 12	Node-splitting analysis for the TMT-B outcome in older adults with mild cognitive impairment	50-51
Table 13	Node-splitting analysis for the DST outcome in older adults with mild cognitive impairment	51-52
Table 14	Node-splitting analysis for the SCWT outcome in older adults with mild cognitive impairment	52
Table 15	Node-splitting analysis for the DRA outcome in older adults with mild cognitive impairment	52-53
Table 16	Loop-specific inconsistency analysis for the MoCA outcome in older adults with mild cognitive impairment	53-54
Table 17	Loop-specific inconsistency analysis for the MMSE outcome in older adults with mild cognitive impairment	54

Table 18	Loop-specific inconsistency analysis for the ADAS-Cog outcome in older adults with mild cognitive impairment	54
Table 19	Loop-specific inconsistency analysis for the TMT-B outcome in older adults with mild cognitive impairment	54-55
Table 20	Loop-specific inconsistency analysis for the DST outcome in older adults with mild cognitive impairment	55
Table 21	Loop-specific inconsistency analysis for the SCWT outcome in older adults with mild cognitive impairment	55
Table 22	Loop-specific inconsistency analysis for the DRA outcome in older adults with mild cognitive impairment	55
Table 23	Heterogeneity estimates (τ^2) for outcomes with estimable between-study variance.	56
Table 24	Within-node heterogeneity tests for MBE and ME stratified by outcome.	56
Table 25	SUCRA ranking table for the MoCA outcome in older adults with mild cognitive impairment	57
Table 26	SUCRA ranking table for the MMSE outcome in older adults with mild cognitive impairment	57-58
Table 27	SUCRA ranking table for the ADAS-Cog outcome in older adults with mild cognitive impairment	58
Table 28	SUCRA ranking table for the TMT-B outcome in older adults with mild cognitive impairment	58-59
Table 29	SUCRA ranking table for the DST outcome in older adults with mild cognitive impairment	59
Table 30	SUCRA ranking table for the SCWT outcome in older adults with mild cognitive impairment	59-60
Table 31	SUCRA ranking table for the DRA outcome in older adults with mild cognitive impairment	60
Table 32	SUCRA ranking table for the MFA outcome in older adults with mild cognitive impairment	60-61
Table 33	Meta-regression analysis of the MoCA outcome with mean age as a moderator in older adults with mild cognitive impairment	61
Table 34	Meta-regression analysis of the MMSE outcome with mean age as a moderator in older adults with mild cognitive impairment	61-62
Table 35	Meta-regression analysis of the TMT-B outcome with mean age as a moderator in older adults with mild cognitive impairment	62
Table 36	Meta-regression analysis of the DST outcome with mean age as a moderator in older adults with mild cognitive impairment	62
Table 37	Meta-regression analysis of the DRA outcome with mean age as a moderator in older adults with	63

	mild cognitive impairment	
Table 38	Meta-regression analysis of the MFA outcome with mean age as a moderator in older adults with mild cognitive impairment	63
Table 39	Meta-regression analysis of the MoCA outcome with intervention frequency as a moderator in older adults with mild cognitive impairment	64
Table 40	Meta-regression analysis of the MMSE outcome with intervention frequency as a moderator in older adults with mild cognitive impairment	64-65
Table 41	Meta-regression analysis of the TMT-B outcome with intervention frequency as a moderator in older adults with mild cognitive impairment	65
Table 42	Meta-regression analysis of the DST outcome with intervention frequency as a moderator in older adults with mild cognitive impairment	66
Table 43	Meta-regression analysis of the DRA outcome with intervention frequency as a moderator in older adults with mild cognitive impairment	66-67
Table 44	Meta-regression analysis of the MFA outcome with intervention frequency as a moderator in older adults with mild cognitive impairment	67
Table 45	Meta-regression analysis of the MoCA outcome with intervention duration as a moderator in older adults with mild cognitive impairment	67-68
Table 46	Meta-regression analysis of the MMSE outcome with intervention duration as a moderator in older adults with mild cognitive impairment	68
Table 47	Meta-regression analysis of the TMT-B outcome with intervention duration as a moderator in older adults with mild cognitive impairment	68
Table 48	Meta-regression analysis of the DST outcome with intervention duration as a moderator in older adults with mild cognitive impairment	69-70
Table 49	Meta-regression analysis of the DRA outcome with intervention duration as a moderator in older adults with mild cognitive impairment	70
Table 50	Meta-regression analysis of the MFA outcome with intervention duration as a moderator in older adults with mild cognitive impairment	70-71
Table 51	Meta-regression analysis of the MoCA outcome with completion rate as a moderator in older adults with mild cognitive impairment.	71
Table 52	Meta-regression analysis of the MMSE outcome with completion rate as a moderator in older adults with mild cognitive impairment.	72

Table 53	Meta-regression analysis of the TMT-B outcome with completion rate as a moderator in older adults with mild cognitive impairment.	72-73
Table 54	Meta-regression analysis of the DST outcome with completion rate as a moderator in older adults with mild cognitive impairment.	73
Table 55	Meta-regression analysis of the DRA outcome with completion rate as a moderator in older adults with mild cognitive impairment.	74
Table 56	Meta-regression analysis of the MFA outcome with completion rate as a moderator in older adults with mild cognitive impairment.	74-75
Table 57	Meta-regression analysis of the MoCA outcome with supervision status as a moderator in older adults with mild cognitive impairment.	75
Table 58	Meta-regression analysis of the MMSE outcome with supervision status as a moderator in older adults with mild cognitive impairment.	75-76
Table 59	Meta-regression analysis of the TMT-B outcome with supervision status as a moderator in older adults with mild cognitive impairment.	76
Table 60	Meta-regression analysis of the DST outcome with supervision status as a moderator in older adults with mild cognitive impairment.	76
Table 61	Meta-regression analysis of the DRA outcome with supervision status as a moderator in older adults with mild cognitive impairment.	77
Table 62	Meta-regression analysis of the MFA outcome with supervision status as a moderator in older adults with mild cognitive impairment.	77
Table 63	Meta-regression analysis of the MoCA outcome with level of social interaction as a moderator in older adults with mild cognitive impairment.	78
Table 64	Meta-regression analysis of the MMSE outcome with level of social interaction as a moderator in older adults with mild cognitive impairment.	79
Table 65	Meta-regression analysis of the TMT-B outcome with level of social interaction as a moderator in older adults with mild cognitive impairment.	79-80
Table 66	Meta-regression analysis of the DST outcome with level of social interaction as a moderator in older adults with mild cognitive impairment.	80-81
Table 67	Meta-regression analysis of the DRA outcome with level of social interaction as a moderator in older adults with mild cognitive impairment.	81-82
Table 68	Meta-regression analysis of the MFA outcome with level of social interaction as a moderator in older	82

	adults with mild cognitive impairment.	
Table 69	Meta-regression analysis of the MoCA outcome with level of cognitive engagement as a moderator in older adults with mild cognitive impairment.	82-83
Table 70	Meta-regression analysis of the MMSE outcome with level of cognitive engagement as a moderator in older adults with mild cognitive impairment.	83
Table 71	Meta-regression analysis of the TMT-B outcome with level of cognitive engagement as a moderator in older adults with mild cognitive impairment.	84
Table 72	Meta-regression analysis of the DST outcome with level of cognitive engagement as a moderator in older adults with mild cognitive impairment.	84
Table 73	Meta-regression analysis of the DRA outcome with level of cognitive engagement as a moderator in older adults with mild cognitive impairment.	85
Table 74	Meta-regression analysis of the MFA outcome with level of cognitive engagement as a moderator in older adults with mild cognitive impairment.	85-86
Table 75	Meta-regression analysis of the MoCA outcome with exercise intensity as a moderator in older adults with mild cognitive impairment.	86
Table 76	Meta-regression analysis of the MMSE outcome with exercise intensity as a moderator in older adults with mild cognitive impairment.	87
Table 77	Meta-regression analysis of the TMT-B outcome with exercise intensity as a moderator in older adults with mild cognitive impairment.	87
Table 78	Meta-regression analysis of the DST outcome with exercise intensity as a moderator in older adults with mild cognitive impairment.	88
Table 79	Meta-regression analysis of the DRA outcome with exercise intensity as a moderator in older adults with mild cognitive impairment.	88-89
Table 80	Meta-regression analysis of the MFA outcome with exercise intensity as a moderator in older adults with mild cognitive impairment.	89
Table 81	Meta-regression analysis of the MoCA outcome with intervention dose as a moderator in older adults with mild cognitive impairment.	89-90
Table 82	Meta-regression analysis of the MMSE outcome with intervention dose as a moderator in older adults with mild cognitive impairment.	90
Table 83	Meta-regression analysis of the TMT-B outcome with intervention dose as a moderator in older adults with mild cognitive impairment.	91

Table 84	Meta-regression analysis of the DST outcome with intervention dose as a moderator in older adults with mild cognitive impairment.	91-92
Table 85	Meta-regression analysis of the DRA outcome with intervention dose as a moderator in older adults with mild cognitive impairment.	92
Table 86	Meta-regression analysis of the MFA outcome with intervention dose as a moderator in older adults with mild cognitive impairment.	93
Table 87	Sensitivity analysis for the MoCA outcome in older adults with mild cognitive impairment.	93-102
Table 88	Sensitivity analysis for the MMSE outcome in older adults with mild cognitive impairment.	102-108
Table 89	Sensitivity analysis for the TMT-B outcome in older adults with mild cognitive impairment.	109-112
Table 90	Sensitivity analysis for the DST outcome in older adults with mild cognitive impairment.	113-116
Table 91	Sensitivity analysis for the DRA outcome in older adults with mild cognitive impairment.	116-121
Table 92	Sensitivity analysis for the MFA outcome in older adults with mild cognitive impairment.	122-124
Table 93	Sensitivity analysis after excluding studies at high risk of bias: SUCRA ranking table for the MoCA outcome in older adults with mild cognitive impairment.	124-125
Table 94	Sensitivity analysis after excluding studies at high risk of bias: SUCRA ranking table for the MMSE outcome in older adults with mild cognitive impairment.	125
Table 95	Sensitivity analysis after excluding studies at high risk of bias: SUCRA ranking table for the ADAS-Cog outcome in older adults with mild cognitive impairment.	126
Table 96	Sensitivity analysis after excluding studies at high risk of bias: SUCRA ranking table for the TMT-B outcome in older adults with mild cognitive impairment.	126-127
Table 97	Sensitivity analysis after excluding studies at high risk of bias: SUCRA ranking table for the DST outcome in older adults with mild cognitive impairment.	127
Table 98	Sensitivity analysis after excluding studies at high risk of bias: SUCRA ranking table for the SCWT outcome in older adults with mild cognitive impairment.	127
Table 99	Sensitivity analysis after excluding studies at high risk of bias: SUCRA ranking table for the DRA outcome in older adults with mild cognitive impairment.	128
Table 100	Sensitivity analysis after excluding studies at high risk of bias: SUCRA ranking table for the MFA outcome in older adults with mild cognitive impairment.	128
Table 101	Egger’s regression tests for funnel plot asymmetry and small-study effects across MoCA, MMSE, TMT-B, DST, DRA, and MFA networks.	129

Table 102	GRADE certainty of evidence for the MoCA outcome in older adults with mild cognitive impairment	130-132
Table 103	GRADE certainty of evidence for the MMSE outcome in older adults with mild cognitive impairment	132-136
Table 104	GRADE certainty of evidence for the ADAS-Cog outcome in older adults with mild cognitive impairment	136-138
Table 105	GRADE certainty of evidence for the TMT-B outcome in older adults with mild cognitive impairment	138-140
Table 106	GRADE certainty of evidence for the DST outcome in older adults with mild cognitive impairment	140-142
Table 107	GRADE certainty of evidence for the SCWT outcome in older adults with mild cognitive impairment	142-144
Table 108	GRADE certainty of evidence for the DRA outcome in older adults with mild cognitive impairment	144-146
Table 109	GRADE certainty of evidence for the MFA outcome in older adults with mild cognitive impairment	147-148
Figure 1	RoB 2.0 quality assessment of studies	149
Figure 2	League table from the network meta-analysis comparing intervention nodes for older adults with mild cognitive impairment. MDs and 95% CIs are presented for MoCA (lower-left triangle) and MMSE (upper-right triangle).	150
Figure 3	League table from the network meta-analysis comparing intervention nodes for older adults with mild cognitive impairment. MDs and 95% CIs are presented for TMT-B (lower-left triangle) and ADAS-Cog (upper-right triangle).	151
Figure 4	League table from the network meta-analysis comparing intervention nodes for older adults with mild cognitive impairment. MDs and 95% CIs are presented for SCWT (lower-left triangle) and SMDs and 95% CIs are presented for DST (upper-right triangle).	152
Figure 5	League table from the network meta-analysis comparing intervention nodes for older adults with mild cognitive impairment. SMDs and 95% CIs are presented for MFA (lower-left triangle) and DRA (upper-right triangle).	153
Figure 6	Cumulative ranking probability curves of different interventions for MoCA in older adults with mild cognitive impairment.	154
Figure 7	Cumulative ranking probability curves of different interventions for MMSE in older adults with mild cognitive impairment.	155
Figure 8	Cumulative ranking probability curves of different interventions for ADAS-Cog in older adults with	156

mild cognitive impairment.

Figure 9 Cumulative ranking probability curves of different interventions for TMT-B in older adults with mild cognitive impairment. 157

Figure 10 Cumulative ranking probability curves of different interventions for DST in older adults with mild cognitive impairment. 158

Figure 11 Cumulative ranking probability curves of different interventions for SCWT in older adults with mild cognitive impairment. 159

Figure 12 Cumulative ranking probability curves of different interventions for DRA in older adults with mild cognitive impairment. 160

Figure 13 Cumulative ranking probability curves of different interventions for MFA in older adults with mild cognitive impairment. 161

Figure 14 Comparison-adjusted funnel plot for the MoCA outcome in older adults with mild cognitive impairment. 162

Figure 15 Comparison-adjusted funnel plot for the MMSE outcome in older adults with mild cognitive impairment. 162

Figure 16 Comparison-adjusted funnel plot for the TMT-B outcome in older adults with mild cognitive impairment. 163

Figure 17 Comparison-adjusted funnel plot for the DST outcome in older adults with mild cognitive impairment. 163

Figure 18 Comparison-adjusted funnel plot for the DRA outcome in older adults with mild cognitive impairment. 164

Figure 19 Comparison-adjusted funnel plot for the MFA outcome in older adults with mild cognitive impairment. 164

Supplementary Table 1. PRISMA NMA Checklist of Items to Include When Reporting a Systematic Review Involving a Network Meta-analysis

Section/Topic	Item	Checklist Item	Reported on Page
**Title**	**1**	Comparative efficacy and certainty of evidence of exercise interventions for cognitive outcomes in older adults with mild cognitive impairment: a systematic review and network meta-analysis of randomized controlled trials	**1**
**ABSTRACT**			
**Structured summary**	**2**	**Structured abstract reporting background, methods, results, conclusions, and PROSPERO registration. Discussion/Conclusions: limitations; conclusions and implications of findings.**	**1-2**
**INTRODUCTION**			
**Rationale**	**3**	**MCI is common in older adults and is associated with dementia progression, functional decline, and impaired quality of life, underscoring the need for effective and clinically meaningful interventions.**	**2-3**
**Objectives**	**4**	**To compare the efficacy and certainty of evidence of prespecified intervention nodes for cognitive outcomes in adults aged ⩾60 years with MCI.**	**3**
**METHODS**			
**Protocol and registration**	**5**	**The review protocol was prospectively registered in PROSPERO (CRD420261372312).**	**3**
**Eligibility criteria**	**6**	**We included RCTs in adults aged ⩾60 years with MCI that evaluated prespecified interventions and reported sufficient data for at least one eligible outcome.**	**4-5**
**Information sources**	**7**	**PubMed, Embase, Cochrane Library, and Web of Science were searched from inception to March 1, 2026.**	**3**
**Search**	**8**	**The search combined controlled vocabulary and free-text terms; the full search strategy is provided in Supplementary Table 2.**	**3-4;** Supplementary **Table 2**
**Study selection**	**9**	**Two reviewers independently screened titles/abstracts and full texts, with disagreements resolved by discussion or a third reviewer.**	**5**
**Data collection process**	**10**	**Two reviewers independently extracted data, with disagreements resolved by discussion or a third reviewer.**	**5-6**

**Data items**	**11**	**Extracted data included study characteristics, participant characteristics, intervention/comparator details, follow-up, and outcome data.**	**6; Supplementary Tables 5–7**
**Geometry of the network**	**12**	**Interventions were grouped into prespecified nodes, and outcome-specific network plots were constructed.**	**4-7; Figures 2 and 5**
**Risk of bias within individual studies**	**13**	**Risk of bias in individual studies was assessed using the Cochrane Risk of Bias 2 (RoB 2) tool.**	**6 and 9–10; Supplementary Figure 1**
**Summary measures**	**14**	**Effect sizes were expressed as MDs or SMDs with 95% CIs; treatment ranking was summarized using SUCRA, probability of being best, and mean rank.**	**6–7; Figures 3, 4, 6 and 7; Supplementary Tables 25–32**
**Planned methods of analysis**	**15**	**Random-effects consistency models were used; τ^2 was estimated with REML, and multi-arm trials were handled by preserving within-study correlations.**	**6-7**
**Assessment of Inconsistency**	**16**	**Global inconsistency, node-splitting, and loop-specific inconsistency factors were used to assess inconsistency.**	**6 and 10; Supplementary Tables 8–22**
**Risk of bias across studies**	**17**	**Small-study effects/publication bias were assessed using comparison-adjusted funnel plots when at least 10 studies were available.**	**7 and 14; Supplementary Figures 14–19; Supplementary Table 101**
**Additional analyses**	**18**	**Additional analyses included meta-regression and sensitivity analyses.**	**7 and 13–14; Supplementary Tables 33–100; Supplementary Figures 2–5**
**RESULTS†**			
**Study selection**	**19**	**A total of 14,641 records were identified. After deduplication and title/abstract screening, 263 full-text articles were assessed, and 74 studies were included (Figure 1).**	**9; Figure 1**
**Presentation of**	**20**	**Network plots for all outcomes are shown in Figures 2 and 5.**	**11–13; Figures 2 and**

network structure			5
Summary of network geometry	21	Seven outcome networks contained closed loops, whereas the MFA network did not. Network geometry is shown in Figures 2 and 5.	10; Figures 2 and 5
Study characteristics	22	Study characteristics are summarized in Supplementary Tables 5 and 6.	9; Supplementary Tables 5–7
Risk of bias within studies	23	RoB 2 assessments showed 25 studies at low risk, 34 with some concerns, and 15 at high risk (Supplementary Figure 1).	9–10; Supplementary Figure 1
Results of individual studies	24	Study-level outcome data and effect estimates are presented in Figures 3, 4, 6, and 7 and Supplementary Tables 23 – 30.	11–13; Figures 3, 4, 6 and 7; Supplementary Tables 25–32
Synthesis of results	25	Network meta-analysis results, including relative effects and treatment rankings, are presented in Figures 3, 4, 6, and 7 and Supplementary Tables 23 – 30.	11–13; Figures 3, 4, 6 and 7; Supplementary Tables 25–32
Exploration for inconsistency	26	No significant global or local inconsistency was detected in the closed-loop networks; inconsistency was not assessable for MFA.	10; Supplementary Tables 8–22
Risk of bias across studies	27	Funnel plots and Egger's tests were used as exploratory assessments of small-study effects/publication bias.	14; Supplementary Figures 14–19; Supplementary Table 101
Results of additional analyses	28	Meta-regression and sensitivity analyses were exploratory and generally showed broadly consistent findings.	13–14; Supplementary Tables 33–100; Supplementary Figures 2–5
DISCUSSION			
Summary of evidence	29	Moderate- to high-certainty evidence supported differential benefits across outcomes, with MBE, RT, D/VRE, ME, and ME-CT showing relative advantages in specific domains.	14-17
Limitations	30	Limitations included risk of bias in some trials, sparse networks for some outcomes, the open-loop MFA network, and limited assessment of publication bias in small networks.	18

**Conclusions**	**31**	**Exercise interventions may benefit older adults with MCI, but certainty varied across comparisons; further high-quality head-to-head RCTs are needed.**	**18-19**
**FUNDING**			
**Funding**	**32**	**This research was funded by the Sports Culture Research Base of the National Sports Administration Bureau, located at Hainan Normal University; and this research was the outcome of Hainan Province Philosophy and Social Science Planning Project(HNSK(ZC) 25-224) “Construction of Personalized Health Care Service Model for Chronic Disease Exercise-Climate Synergistic Intervention under the Background of Free Trade Port”**	**19**

Supplementary Table 2. Literature Search Strategy.

PubMed	#1 "Cognitive Dysfunction"[MeSH Terms] 54,276
	#2 "cognitive dysfunctions"[Title/Abstract] OR "dysfunction cognitive"[Title/Abstract] OR "dysfunctions cognitive"[Title/Abstract] OR "cognitive disorder"[Title/Abstract] OR "cognitive disorders"[Title/Abstract] OR "disorder cognitive"[Title/Abstract] OR "disorders cognitive"[Title/Abstract] OR "cognitive impairments"[Title/Abstract] OR "cognitive impairment"[Title/Abstract] OR "impairment cognitive"[Title/Abstract] OR "impairments cognitive"[Title/Abstract] OR "mild cognitive impairment"[Title/Abstract] OR "cognitive impairment mild"[Title/Abstract] OR "cognitive impairments mild"[Title/Abstract] OR "impairment mild cognitive"[Title/Abstract] OR "impairments mild cognitive"[Title/Abstract] OR "mild cognitive impairments"[Title/Abstract] OR "cognitive decline"[Title/Abstract] OR "cognitive declines"[Title/Abstract] OR "decline cognitive"[Title/Abstract] OR "declines cognitive"[Title/Abstract] OR "mental deterioration"[Title/Abstract] OR "deterioration mental"[Title/Abstract] OR (("deteriorate"[All Fields] OR "deteriorated"[All Fields] OR "deteriorates"[All Fields] OR "deteriorating"[All Fields] OR "Deterioration"[All Fields] OR "Deteriorations"[All Fields] OR "deteriorative"[All Fields]) AND "Mental"[Title/Abstract]) OR "mental deteriorations"[Title/Abstract] 170,070
	#3 #1 OR #2 180,032
	#4 "Aged"[MeSH Terms] 3,861,005
	#5 "Elderly"[Title/Abstract] 337,309
	#6 #4 OR #5 3,961,099
	#7 #3 AND #6 69,393
	#8 "Exercise"[MeSH Terms] 285,389
	#9 "Exercises"[Title/Abstract] OR "exercise physical"[Title/Abstract] OR "exercises physical"[Title/Abstract] OR "physical exercise"[Title/Abstract] OR "physical exercises"[Title/Abstract] OR "exercise isometric"[Title/Abstract] OR "exercises isometric"[Title/Abstract] OR "isometric exercises"[Title/Abstract] OR "isometric exercise"[Title/Abstract] OR "exercise aerobic"[Title/Abstract] OR "aerobic exercise"[Title/Abstract] OR "aerobic exercises"[Title/Abstract] OR "exercises aerobic"[Title/Abstract] OR "exercise training"[Title/Abstract] OR "exercise trainings"[Title/Abstract] OR "training exercise"[Title/Abstract] OR (("education"[MeSH Subheading] OR "education"[All Fields] OR "Training"[All Fields] OR "education"[MeSH Terms] OR "train"[All Fields] OR "train s"[All Fields] OR "trained"[All Fields] OR "training s"[All Fields] OR "Trainings"[All Fields] OR "trains"[All Fields]) AND "Exercise"[Title/Abstract]) OR "physical activity"[Title/Abstract] OR "activities physical"[Title/Abstract] OR "activity physical"[Title/Abstract] OR "physical activities"[Title/Abstract] OR "active breaks"[Title/Abstract] OR "activity breaks"[Title/Abstract] OR "acute exercise"[Title/Abstract] OR "acute exercises"[Title/Abstract] OR "exercise acute"[Title/Abstract] OR "exercises acute"[Title/Abstract] 366,758

#10 #8 OR #9 510,967

#11 "Cognitive Training"[MeSH Terms] 648

#12 "training cognitive"[Title/Abstract] OR "cognitive rehabilitation"[Title/Abstract] OR "rehabilitation cognitive"[Title/Abstract] OR "brain training"[Title/Abstract] OR "training brain"[Title/Abstract] OR "memory training"[Title/Abstract] OR "training memory"[Title/Abstract] 5,111

#13 #11 OR #12 5,474

#14 "Music"[MeSH Terms] 18,769

#15 "Songs"[Title/Abstract] OR "Song"[Title/Abstract] OR "vocal melody"[Title/Abstract] OR "melodies vocal"[Title/Abstract] OR "melody vocal"[Title/Abstract] OR "vocal melodies"[Title/Abstract] OR "classical music"[Title/Abstract] OR "music classical"[Title/Abstract] OR "jazz music"[Title/Abstract] OR "music jazz"[Title/Abstract] OR "rap music"[Title/Abstract] OR "music rap"[Title/Abstract] OR "hip hop music"[Title/Abstract] OR ("Hop"[All Fields] AND "music hip"[Title/Abstract]) OR "music hip hop"[Title/Abstract] OR ("Rock"[Title/Abstract] AND "Roll"[Title/Abstract]) OR "Music"[Title/Abstract] 37,037

#16 #14 OR #15 44,368

#17 "Dancing"[MeSH Terms] 4,032

#18 "Dance"[Title/Abstract] OR "Ballet"[Title/Abstract] OR "jazz dance"[Title/Abstract] OR "dance jazz"[Title/Abstract] OR "tap dance"[Title/Abstract] OR (("danced"[All Fields] OR "Dancing"[MeSH Terms] OR "Dancing"[All Fields] OR "Dance"[All Fields] OR "dances"[All Fields]) AND "Tap"[Title/Abstract]) OR "modern dance"[Title/Abstract] OR "dance modern"[Title/Abstract] OR "hip hop dance"[Title/Abstract] OR (("danced"[All Fields] OR "Dancing"[MeSH Terms] OR "Dancing"[All Fields] OR "Dance"[All Fields] OR "dances"[All Fields]) AND "Hip-Hop"[Title/Abstract]) OR "hip hop dance"[Title/Abstract] OR "line dancing"[Title/Abstract] OR (("danced"[All Fields] OR "Dancing"[MeSH Terms] OR "Dancing"[All Fields] OR "Dance"[All Fields] OR "dances"[All Fields]) AND "Line"[Title/Abstract]) OR "salsa dancing"[Title/Abstract] OR "dancing salsa"[Title/Abstract] OR "square dance"[Title/Abstract] OR (("danced"[All Fields] OR "Dancing"[MeSH Terms] OR "Dancing"[All Fields] OR "Dance"[All Fields] OR "dances"[All Fields]) AND "Square"[Title/Abstract]) 8,463

#19 #17 OR #18 9,462

#20 "Video Games"[MeSH Terms] 9,110

#21 "games video"[Title/Abstract] OR "game video"[Title/Abstract] OR "video game"[Title/Abstract] OR "Videogame"[Title/Abstract] OR "Videogames"[Title/Abstract] OR "computer games"[Title/Abstract] OR "computer game"[Title/Abstract] OR "game computer"[Title/Abstract] OR "games computer"[Title/Abstract] 6,112

#22 #20 OR #21 12,323

	#23 #10 OR #13 OR #16 OR #19 OR #22 574,254<br><br>#24 #7 AND #23 4,692<br><br>#25 #7 AND #23 Filters: Randomized Controlled Trial 863
Web of Science	#1 TS=(“Cognitive Dysfunction” or “Cognitive Dysfunctions” or “Dysfunction, Cognitive” or “Dysfunctions, Cognitive” or “Cognitive Disorder” or “Cognitive Disorders” or “Disorder, Cognitive” or “Disorders, Cognitive” or “Cognitive Impairments” or “Cognitive Impairment” or “Impairment, Cognitive” or “Impairments, Cognitive” or “Mild Cognitive Impairment” or “Cognitive Impairment, Mild” or “Cognitive Impairments, Mild” or “Impairment, Mild Cognitive” or “Impairments, Mild Cognitive” or “Mild Cognitive Impairments” or “Cognitive Decline” or “Cognitive Declines” or “Decline, Cognitive” or “Declines, Cognitive” or “Mental Deterioration” or “Deterioration, Mental” or “Deteriorations, Mental” or “Mental Deteriorations”) 71,317<br><br>#2: TS=(“Aged” or “Elderly”) 293,669<br><br>#3 #1 AND #2 12,214<br><br>#4 TS=(“Exercise” or “Exercises” or “Exercise, Physical” or “Exercises, Physical” or “Physical Exercise” or “Physical Exercises” or “Exercise, Isometric” or “Exercises, Isometric” or “Isometric Exercises” or “Isometric Exercise” or “Exercise, Aerobic” or “Aerobic Exercise” or “Aerobic Exercises” or “Exercises, Aerobic” or “Exercise Training” or “Exercise Trainings” or “Training, Exercise” or “Trainings, Exercise” or “Physical Activity” or “Activities, Physical” or “Activity, Physical” or “Physical Activities” or “Active Breaks” or “Activity Breaks” or “Acute Exercise” or “Exercise, Acute” or “Acute Exercises”) 170,649<br><br>#5 TS=(“Training, Cognitive” or “Cognitive Rehabilitation” or “Rehabilitation, Cognitive” or “Brain Training” or “Training, Brain” or “Memory Training” or “Training, Memory” or “Cognitive Training”) 3,694<br><br>#6 TS=(“Music” or “Songs” or “Song” or “Vocal Melody” or “Melodies, Vocal” or “Melody, Vocal” or “Vocal Melodies” or “Classical Music” or “Music, Classical” or “Jazz Music” or “Music, Jazz” or “Rap Music” or “Music, Rap” or “Hip Hop Music” or “Hop Music, Hip” or “Music, Hip Hop” or “Rock and Roll Music”) 22,087<br><br>#7 TS=(“Dancing” or “Dance” or “Ballet” or “Jazz Dance” or “Dance, Jazz” or “Tap Dance” or “Dance, Tap” or “Modern Dance” or “Dance, Modern” or “Hip-Hop Dance” or “Dance, Hip-Hop” or “Hip Hop Dance” or “Line Dancing” or “Dancing, Line” or “Salsa Dancing” or “Dancing, Salsa” or “Square Dance” or “Dance, Square”) 4,814<br><br>#8 TS=(“Video Games” or “Games, Video” or “Game, Video” or “Video Game” or “Videogame” or “Videogames” or “Computer Games” or “Computer Game” or “Game, Computer” or “Games,

	Computer") 5,192<br>#9 #4 OR #5 OR #6 OR #7 OR #8 202,370<br>#10 TS=("randomized controlled trial") 59,949<br>#11 #3 AND #9 AND #10 165
Cochrane	ID SearchHits<br>#1 MeSH descriptor: [Cognitive Dysfunction] explode all trees 5,283<br>#2 ('Cognitive Dysfunctions' OR 'Impairments, Cognitive' OR 'Cognitive Impairment' OR 'Impairment, Cognitive' OR 'Cognitive Disorder' OR 'Cognitive Impairments' OR 'Disorders, Cognitive' OR 'Disorder, Cognitive' OR 'Cognitive Disorders' OR 'Dysfunction, Cognitive' OR 'Dysfunctions, Cognitive' OR 'Mild Cognitive Impairment' OR 'Mild Cognitive Impairments' OR 'Impairments, Mild Cognitive' OR 'Cognitive Impairment, Mild' OR 'Impairment, Mild Cognitive' OR 'Cognitive Impairments, Mild' OR 'Declines, Cognitive' OR 'Mental Deteriorations' OR 'Decline, Cognitive' OR 'Cognitive Decline' OR 'Mental Deterioration' OR 'Deteriorations, Mental' OR 'Deterioration, Mental' OR 'Cognitive Declines'):ti,ab,kw 67,403<br>#3 #1 OR #2 67,420<br>#4 MeSH descriptor: [Aged] explode all trees 291,574<br>#5 (Elderly):ti,ab,kw 66,619<br>#6 #4 OR #5 337,695<br>#7 #3 AND #6 13,316<br>#8 MeSH descriptor: [Exercise] explode all trees 42,846<br>#9 ('Exercises' OR 'Exercise, Physical' OR 'Physical Exercises' OR 'Physical Exercise' OR 'Exercises, Physical' OR 'Physical Activity' OR 'Physical Activities' OR 'Activity, Physica' OR 'Activities, Physical' OR 'Trainings, Exercise' OR 'Training, Exercise' OR 'Exercise Training' OR 'Exercise Trainings' OR 'Exercises, Isometric' OR 'Exercise, Isometric' OR 'Isometric Exercises' OR 'Isometric Exercise' OR 'Aerobic Exercises' OR 'Aerobic Exercise' OR 'Exercise, Aerobic' OR 'Exercises, Aerobic' OR 'Acute Exercises' OR 'Acute Exercise' OR 'Exercises, Acute' OR 'Exercise, Acute' OR 'Activity Breaks' OR 'Active Breaks'):ti,ab,kw 168,140<br>#10 #8 OR #9 178,770<br>#11 MeSH descriptor: [Cognitive Training] explode all trees 364<br>#12 ('Training, Memory' OR 'Memory Training' OR 'Brain Training' OR 'Training, Cognitive' OR 'Cognitive Rehabilitation' OR 'Training, Brain' OR 'Rehabilitation, Cognitive'):ti,ab,kw 36,772

	#13 #11 OR #12 36,772
	#14 MeSH descriptor: [Music] explode all trees 1,327
	#15 (′Songs′ OR ′Song′ OR ′Rock and Roll Music′ OR ′Music, Hip Hop′ OR ′Hip Hop Music′ OR ′Hop Music, Hip′ OR ′Rap Music′ OR ′Music, Rap′ OR ′Music, Classical′ OR ′Classical Music′ OR ′Vocal Melody′ OR ′Melody, Vocal′ OR ′Melodies, Vocal′ OR ′Vocal Melodies′ OR ′Music, Jazz′ OR ′Jazz Music′):ti,ab,kw 1,237
	#16 #14 OR #15 2,402
	#17 MeSH descriptor: [Dancing] explode all trees 337
	#18 (′Salsa Dancing′ OR ′Dancing, Salsa′ OR ′Line Dancing′ OR ′Dancing, Line′ OR ′Hip-Hop Dance′ OR ′Dance, Hip-Hop′ OR ′Hip Hop Dance′ OR ′Ballet′ OR ′Dance′ OR ′Square Dance′ OR ′Dance, Square′ OR ′Dance, Modern′ OR ′Modern Dance′ OR ′Jazz Dance′ OR ′Dance, Jazz′ OR ′Dance, Tap′ OR ′Tap Dance′):ti,ab,kw 1,679
	#19 #17 OR #18 1,721
	#20 MeSH descriptor: [Video Games] explode all trees 1,482
	#21 (′Games, Video′ OR ′Video Game′ OR ′Videogames′ OR ′Videogame′ OR ′Game, Video′ OR ′Games, Computer′ OR ′Computer Games′ OR ′Game, Computer′ OR ′Computer Game′):ti,ab,kw 4,644
	#22 #20 OR #21 4,708
	#23 #10 OR #13 OR #16 OR #19 OR #22 208,005
	#24 (′randomized controlled trial′ OR ′randomized′ OR ′placebo′):ti,ab,kw 1,428,375
	#25 #7 AND #23 AND #24 3,164
Embase	#1 'cognition'/exp 3,836,199
	#2 'cognitive accessibility' OR 'cognitive balance' OR 'cognitive dissonance' OR 'cognitive function' OR 'cognitive functioning' OR 'cognitive structure' OR 'cognitive symptoms' OR 'cognitive task' OR 'cognitive thinking' OR 'neurobehavioural manifestations' OR 'volition' OR 'cognition' 638,703
	#3 #1 OR #2 3,927,549
	#4 'aged'/exp 4,717,069
	#5 'aged patient' OR 'aged people' OR 'aged person' OR 'aged subject' OR 'elderly' OR 'elderly patient' OR 'elderly people' OR 'elderly person' OR 'elderly subject' OR 'senior citizen' OR 'senium' OR 'aged' 7,327,027

#6 #4 OR #5 7,327,027

#7 #3 AND #6 727,102

#8 'exercise'/exp 575,990

#9 'biometric exercise' OR 'effort' OR 'exercise capacity' OR 'exercise performance' OR 'exercise training' OR 'exertion' OR 'fitness training' OR 'fitness workout' OR 'physical conditioning, human' OR 'physical effort' OR 'physical exercise' OR 'physical exertion' OR 'physical work-out' OR 'physical workout' OR 'exercise' 1,088,780

#10 #8 OR #9 1,111,248

#11 'cognitive rehabilitation'/exp 8,938

#12 'brain training' OR 'cognitive training' OR 'cognitive rehabilitation' 16,291

#13 #11 OR #12 16,291

#14 'music'/exp 29,843

#15 'music preference' OR 'musical ability' OR 'musical test' OR 'orchestra' OR 'rock and roll music' OR 'music' 53,334

#16 #14 OR #15 53,819

#17 'dancing'/exp 8,893

#18 'dance' OR 'dancer' OR 'dancing' 19,300

#19 #17 OR #18 19,300

#20 'video game'/exp 10,119

#21 'computer game' OR 'computer game' OR 'television game' OR 'tv games' OR 'video games' OR 'videogame' OR 'videogames' OR 'video game' 14,084

#22 #20 OR #21 14,890

#23 #10 OR #13 OR #16 OR #19 OR #22 1,195,827

#24 #7 AND #23 53,012

#25 #24 AND 'randomized controlled trial'/de 10,449

Supplementary Table 3. Pre-transformation data, calculation formulas, and data formats.

First author	Year	outcome	Experimental group pre-conversion pre-intervention data	Experimental group pre-conversion post-intervention data	Control group pre-conversion pre-intervention data	Control group pre-conversion post-intervention data	Formulae	data format
Langoni	2018	DRA	4 (1.8﹣6.0)	2.5 (1.0﹣4.0)	3.5 (2.0﹣7.3)	4.0 (2.0﹣5.3)	Mean ≈ Median<br>SD ≈ (Q3 − Q1) / 1.35	median (IQR)
Takao Suzuki	2012	MMSE		0.32（−0.96, 1.60）		−1.37（−2.66, −0.07）	SE(change mean) = (95%CI 上限 − 95%CI 下限) / [2 × t(0.975, n−1)]<br>SD(change) = SE(change mean) × √n	Mean difference (95% CI)
		MFA		3.83（1.40, 6.25）		0.60（−1.87, 3.06）		
Montero-Odasso	2023	ADAS-Cog		−2.16 (0.65)		−0.07 (0.08)	change SD = SE × √n	mean (SE)
Miaoran Lin	2024	MoCA		3.54 (2.75 to 4.33)		1.4 (0.72 to 2.07)	SE(change mean) = (95%CI 上限 − 95%CI 下限) / [2 × t(0.975, n−1)]<br>SD(change) = SE(change mean) × √n	Mean difference (95% CI)
Goumopoulos	2023	MoCA	21.0 (4.5)	21.5 (6.0)	21.5 (2.5)	21.5 (3.3)	Mean ≈ Median<br>SD ≈ (Q3 − Q1) / 1.35	median (IQR)
		TMT-B	207.5 (55.0)	194.5 (50.0)	202.5 (32.5)	198.0 (38.0)		
		DST	5.5 (1.8)	6.0 (0.8)	6.0 (1.0)	5.5 (1.0)		
		MFA	10.5 (2.8)	11.0 (2.0)	12.0 (2.0)	11.5 (1.8)		
		DRA	3.5 (3.0)	3.0 (2.8)	3.5 (2.8)	3.5 (2.8)		
Givon Schaham	2024	MoCA	23.0（21.0﹣24.0）	23.0（21.7﹣25.0）	23.0（20.0﹣24.0）	22.0（20.0﹣24.0）	Mean ≈ Median	median (IQR)

Hong Yu	2025	MoCA	3.50 (1.53, 5.47)	1.68 (−0.07, 3.43)	SD ≈ (Q3 − Q1) / 1.35<br>SE(change mean) = (95%CI 上限 − 95%CI 下限) / [2 × t(0.975, n−1)]<br>SD(change) = SE(change mean) × √n	Mean difference (95% CI)
		TMT-B	−32.54 (−62.51, −2.56)	−13.18 (−49.41, 23.05)		
		DST	1.39 (0.77, 2.02)	0.89 (0.21, 1.57)		

Supplementary Table 4. Prespecified Minimal Important Differences (MIDs) and Their Rationale for Imprecision Judgement Across Outcomes.

Outcome	Effect Size Metric	Prespecified MID
MoCA	MD	2 points
MMSE	MD	1.7 points
ADAS-Cog	MD	-2 points
TMT-B	MD	−20.1 seconds
DST	SMD	0.4
SCWT	MD	-9.3 seconds
DRA	SMD	-0.3
MFA	SMD	0.3

Supplementary Table 5. Characteristics of the included studies.

First author	Year	Mean age (Years)	Sex (M/F)	Total (n)	Intervention frequency	Duration of intervention	Intervention period	Experimental intervention	Control intervention
Lan Li[1]	2021	70.3	NR	90	5 times a week	30 minutes	24 weeks	Multicomponent exercise: aerobic, strength, balance, coordination, sensitivity exercise	Regular community health instruction
Chang J.[2]	2021	76.29	0/136	136	3 times a week	30 minutes	18 weeks	Chinese square dance: handclapping, high-fiving, chest expansion, arm extension, leg kicking	Not scheduled for specialized physical activity
Lina Wang[3]	2020	68.31	NR	116	3 times a week	60 minutes	24 weeks	Structured limbs-exercise program: limbering-up, upper and lower limbs, relaxation exercise	Health promotion classes: instruction regarding cognitive disorders, healthy eating, living habits
Tomoto T.[4]	2021	65	24/28	52	3 times a week(Weeks 1-10);<br>3-4 times a week(Weeks 11-25);<br>4-5 times a week(Weeks 26-48)	25-30 minutes(Weeks 1-10);<br>30-35 minutes(Weeks 11-25);<br>30-40 minutes(Weeks 26-48)	48 weeks	The moderate-to-vigorous AET program	The SAT program: the upper and lower limb stretching exercises
Binu P. Thomas[5]	2020	66.25	16/14	30	3 times a week(Weeks 1-10);<br>3-4 times a week(Weeks 11-25);<br>4-5 times a week(Weeks 26-48)	25-30 minutes(Weeks 1-10);<br>30-35 minutes(Weeks 11-25);<br>30-40 minutes(Weeks 26-48)	48 weeks	The moderate to vigorous AE	Stretch training: stretch and balance routine
Kerime Bademli[6]	2019	71.46	25/35	60	4 times a week	80 minutes	20 weeks	Physical Activity Program: rhythmic exercises, free walking	No application: daily routine activities
Imran Amjad[7]	2018	58.89	NR	40	3 times a week	20-40 minutes	6 weeks	The aerobics exercise: a treadmill and a stationary bicycle	The no-aerobic exercise: gentle movements, general body stretching

Dan Song.[8]	2019	75.78	30/90	120	3 times a week	60 minutes	16 weeks	Aerobic stepping exercise	Health education program: no information relating to brain health and physical exercise
Soon-Gook Hong[9]	2017	75.34	6/16	22	2 times a week	60 minutes	12 weeks	Resistance exercise program: 15RM (65% of 1RM)	Non-training group: maintain their current lifestyle
Yi Zhu[10]	2018	79.6	NR	60	3 times a week	35 minutes	12 weeks	A specially designed aerobic dance routine	Usual care only: promoting a healthy lifestyle
Lazarou[11]	2017	66.86	28/101	129	2 times a week	60 minutes	40 weeks	International Ballroom Dancing: Tango, Waltz, Viennese Waltz, Fox trot, Rumba, Chachacha, Swing, Salsa, Merengue, Disco – Hustle, Greek traditional ballroom dancing	Control group: maintain a regular lifestyle routine
Lü[12]	2016	69.71	13/32	45	3 times a week	60 minutes	12 weeks	Momentum-based dumbbell training: dumbbell-spinning exercises	Control group: maintain a regular lifestyle routine
Tarumi[13]	2019	64.9	27/43	70	3 times a week(Weeks 1-10); 3-4 times a week(Weeks 11-25); 4-5 times a week(Weeks 26-48)	25-30 minutes(Weeks 1-10); 30-35 minutes(Weeks 11-25); 30-40 minutes(Weeks 26-48)	48 weeks	The moderate-to-high intensity AET program	Stretching and toning: the upper and lower body stretching exercises
Yoon[14]	2017	76	0/58	58	2 times a week	60 minutes	12 weeks	HSPT：“elastic band-based high-speed power training”; LSST：“traditional low-speed strength training	Balance and tone group: continue their routine daily activities, carried out static and dynamic stretching
Thaiyanto[15]	2021	67.9	0/40	40	3 times a week	60 minutes	12 weeks	Multicomponent exercise program: aerobic, resistance, balance exercise	Control group: educational material
Nascimento[16]	2014	68.5	13/32	45	3 times a week	60 minutes	16 weeks	A multimodal physical exercise program:	Control group:

								muscular resistance, aerobic fitness, motor coordination, balance exercise	did not attend a regular physical exercise program
Marta Bisbe[17]	2020	74.89	16/15	31	2 times a week	60 minutes	12 weeks	Choreography group: learning the steps, performing choreography, music support	Physical Therapy group: strength, endurance, flexibility, balance, coordination and gait exercises
Takehiko Doi[18]	2017	76	97/104	201	1 time a week	60 minutes	40 weeks	The dance program: salsa, rumba, waltz, cha-cha, blues, jitterbug, tango; The music program: playing percussion instruments, such as the conga	Health education control group
Wang Shuo[19]	2019	81.08	19/47	66	3 times a week	40 minutes	12 weeks	Chinese square dancing: Jiamusi happy dance aerobics	Usual lifestyle: maintained their usual lifestyle, without square dancing
Rojasavastera[20]	2020	67.28	NR	39	2-3 times a week	65 minutes	5 weeks	Action observation with gait training (AOGT); Gait training (GT)	CT group: no training program
Parial[21]	2023	63.8	14/46	60	3 times a week	60 minutes	12 weeks	Dual-task Zumba Gold (DTZ): executive function, perceptual-motor ability, memory, complex attention	Health education with self-reported PA
Ming Qi[22]	2019	69.9	NR	38	3 times a week	35 minutes	12 weeks	Specially designed moderate-intensity aerobic dance (SDMIAD): knee bending, heel lifts, boxing, shoulder movements, kicking, square stepping, sculling and jumping	The CG : only received usual care
Harris A. Eyre[23]	2017	67.8	27/52	79	1 time a week	60 minutes	12 weeks	Kundalini yoga (KY)	Memory enhancement training (MET):

									education about memory, instruction in specific memory strategies
Lam[24]	2015	75.4	121/434	555	3 times a week	60 minutes	48 weeks	Physical exercise: one stretching & toning , one mind body exercise (e.g. Tai Chi), one aerobic exercise session (e.g. static bicycle riding); Cognitive activity: cognitively demanding activities selected from the reference list (e.g. reading and discussing newspapers, playing board games); Cognitive-Physical activity: one cognitive and two types of mind body exercise	Social activity: social activities from the reference list (e.g. tea gathering, film watching)
Langoni[25]	2018	72.25	12/40	52	2 times a week	60 minutes	24 weeks	Strength and aerobic exercises: ankle weights, elastic bands, dumbbells; walking	Usual routine control: maintained its usual routine
Geoffrey Tremont[26]	2022	71.6	NR	46	2 times a week	60 minutes	12 weeks	Yoga intervention (YI): Pranayama, Seated Meditation, Half Sun Salutations, Standing Postures, Seated Postures, Inversions, Shavasana	Healthy living education (HLE)
Hongyu Yang[27]	2016	67	13/12	25	1 time a week	60 minutes	12 weeks	Yogic meditation(KY + KK intervention)	Memory enhancement training (MET): teaching verbal and visual association strategies
Danny J. Yu[28]	2022	63	NR	50	1 time a week/3 times a week(moderate-intensity); 1 time a week/3 times a week(vigorous-intensity)	150 minutes/50 minutes(moderate-intensity); 75 minutes/75 minutes(vigorous-intensity)	12 weeks	Moderate-intensity or vigorous-intensity Walking exercise	Stretching exercise control group

Langoni[29]	2018	72.25	NR	60	2 times a week	60 minutes	24 weeks	Strength and aerobic training:<br>walked and exercised	Control group:<br>not to initiate any kind of physical or cognitive activity
Tsubasa Tomoto[30]	2021	64.7	20/17	37	3 times a week(Weeks 1-10);<br>3-4 times a week(Weeks 11-25);<br>4-5 times a week(Weeks 26-48)	25-30 minutes(Weeks 1-10);<br>30-35 minutes(Weeks 11-25);<br>30-40 minutes(Weeks 26-48)	48 weeks	The moderate-to-vigorous intensity AET program	Stretching and toning (SAT):<br>the upper and lower limb stretching exercises
Donnezan[31]	2018	76.29	NR	69	2 times a week	60 minutes	12 weeks	Cognitive and physical training:<br>a combination of CT and PT within the same intervention;<br>Aerobic training:<br>on bikes;<br>Cognitive Training:<br>working memory, mental flexibility, inhibition, reasoning and updating	Control group:<br>maintaining their usual lifestyle
Tao J. [32]	2019	65.82	NR	69	3 times a week	60 minutes	24 weeks	Baduanjin:<br>mind-body exercise;<br>Brisk walking	Non-exercise health education control:<br>maintain their original physical activity levels
Somporn Sungkarat[33]	2018	67.9	9/57	66	3 times a week	50 minutes	24 weeks	Tai Chi (TC):<br>the 10-form TC	Educational control group:<br>information related to cognitive impairment and fall prevention
Kate E. Devenney[34]	2019	70.5	30/34	64	1 time a week	30 minutes	1 day	High-intensity aerobic exercise:<br>cycling exercise	Resting control condition:<br>lying in a semi recumbent position
Kohanpour[35]	2017	67.85	40/0	40	3 times a week	21 minutes	12 weeks	Aerobic exercise:<br>running;	Placebo:<br>a concentration of 1/10 000

								Lavender:<br>the essence was administrated at daily dose of 2 drops twice a day;<br>Aerobic exercise + Lavender	such that it did not possess any therapeutic effect
Fuzhong Li[36]	2022	74.6	30/39	69	2 times a week	60 minutes	16 weeks	Cognitive-enhanced Tai Ji Quan:<br>integrated with cognitive exercises	Standard Tai Ji Quan:<br>the same 8-form routine
Angus P. Yu[37]	2022	70.7	40/0	40	3 times a week	60 minutes	24 weeks	Tai Chi;<br>Conventional exercise training	Control group:<br>no intervention and maintained their usual lifestyle
Chien-Liang Liu[38]	2022	73.88	NR	54	3 times a week	50 minutes	12 weeks	Exergaming-based Tai Chi:<br>imitated a virtually-presented TC coach;<br>Traditional Tai Chi:<br>simplified 24 form Yang Style TC	Control group:<br>maintain their usual daily physical activities
Somporn Sungkarat[39]	2016	68.3	9/57	66	3 times a week	50 minutes	15 weeks	Tai Chi classes:<br>Tai Chi principles, the 10-form Tai Chi	Control group:<br>maintain their routine lifestyle
Mei-yi Siu[40]	2018	60	42/118	160	2 times a week	60 minutes	16 weeks	Yang-style simple form of Tai Chi:<br>Tai Chi 24 form	Usual care:<br>continue their daily activities
Phoemsapthawee J.[41]	2016	67.85	0/24	24	5 times a week	30 minutes	12 weeks	Arm Swing Exercise(ASE)	Control period
Jurakic[42]	2017	70.4	0/28	28	3 times a week	30 minutes	8 weeks	HUBER training:<br>combined balance and core resistance training	Pilates training:<br>supine, side-lying, sitting, quadruped, lower- and upper-limb exercises
Scherder[43]	2005	86	5/38	43	3 times a week	30 minutes	6 weeks	Walking group:<br>self-paced slow walking with an ai;	Social visits:<br>continued their normal social

								Hand and face exercises: bending and stretching the fingers, and sliding a wooden club through the hand by moving the fingers	activities
Silvia Varela[44]	2011	78.3	NR	68	3 times a week	30 minutes	12 weeks	Aerobic exercise(40% of heart rate reserve): cycling in a recumbent bike; Aerobic exercise(60% of heart rate reserve): cycling in a recumbent bike	Recreational activities: did not exercise
Takao Suzuki[45]	2012	75.6	27/23	50	2 times a week	90 minutes	48 weeks	Multicomponent exercise: muscle strength ,aerobic exercise, postural balance retraining, dual-task training	Health education control group
Xiu-hong Wei[46]	2014	66	40/20	60	5 times a week	30 minutes	24 weeks	Handball training program: tos, hit, bounce, pass, grab, field going, roll, pinch training	Maintained the original life entertainment
Montero-Odasso[47]	2023	73.1	89/86	175	3 times a week	90 minutes	20 weeks	Exercise + Cognitive training + Vitamin D; Exercise + Cognitive training + Placebo vitamin D; Exercise + Sham cognitive training + Vitamin D; Exercise + Sham cognitive training + Placebo vitamin D	Balance-toning+Sham cognitive training + Placebo vitamin D
Jorge Buele[48]	2024	76.38	11/23	34	2 times a week	40 minutes	6 weeks	Motor training + VR-based cognitive training: social interaction, balance, resistance, whole-body aerobic exercises	Motor training + Traditional cognitive training: social activities, balance, resistance, low intensity aerobic exercises
Carvalho[49]	2025	74.5	NR	66	2 times a week	60 minutes	5 weeks	Digital Cognitive Training Group (CT) : divided attention	Computer games control group (CG)
Miaoran Lin[50]	2024	67.88	NR	100	5 times a week	60 minutes	12 weeks	Tai Chi exercise: TC consisted of 24 postures	Health education: health education about the protection of cognitive

									impairment, maintain routine lifestyle
So Young Moon[51]	2025	72.5	NR	300	1time/1-2 weeks(facility-based);<br>1-2 times a week(home-based);<br>3 times a week(physical exercise)	50 minutes(facility-based);<br>30-40 minutes(home-based);<br>50 minutes(physical exercise)	24 weeks	Multidomain intervention (MI):<br>metabolic and vascular risk factor management + cognitive training + physical exercise + nutritional education + motivational enhancement	Usual care:<br>educational booklets corresponding to their risk factors
Chunhui Zhou[52]	2025	65.51	16/50	66	NR	45-50 minutes	12 weeks	Tai Chi Combined with Music Therapy Group (TCMG):<br>four sets of eight-style Tai Chi music;<br>Tai Chi Group (TCG):<br>four sets of eight-style Tai Chi	Control Group (CG):<br>did not participate in the exercise intervention, maintain their original daily activities
Ha Yeong Choi[53]	2025	73.2	22/58	80	3 times a week	NR	52 weeks	Mobile multidomain intervention:<br>diet + physical activity + metabolic + vascular risk management	Control group:<br>general dietary and physical activity recommendations
Cheung[54]	2024	66.48	NR	28	1 time a week	90 minutes	10 weeks	Chanwuyi Lifestyle Medicine Program(CLMP):<br>nei Gong, the Chan medical principle	strategic memory training (SMT):<br>teach basic neuroscience knowledge
Alessandro Sale[55]	2023	74.5	58/55	113	3 times a week	180 minutes	28 weeks	Train the Brain:<br>combined motor and cognitive intervention	Control group:<br>no-training, normal activity
Jingsong Wu[56]	2023	66.75	NR	60	3 times a week	60 minutes	8 weeks	Computerized Cognitive Training Program (CCT):<br>the CCT consists of 11 modules	Health Education Program as Control
Elmar Graessel[57]	2024	73.35	52/37	89	3 times a week	30 minutes	24 weeks	Individualized computerized cognitive training (iCCT),MAKSCog(iCCT (MAKSCog):<br>adaptive ML-based(10 tasks)	Basic CCT (bCCT):<br>no ML
Goumopoulos[58]	2023	73	10/11	21	2 times a week	60 minutes	12 weeks	COGNIPLAT game platform	Normal daily activities and

									usual care
İsmail Uysal[59]	2022	73.75	NR	58	3 times a week	30 minutes	12 weeks	AG: aerobic exercise training + ower limb strengthening group;<br>DG: dual-task training + lower limb strengthening group;<br>ADG: aerobic exercise training + dual-task training and lower limb strengthening group	CG:<br>only lower limb strengthening exercises
Qiang Zhang[60]	2023	66.7	4/38	42	3 times a week	60 minutes	12 weeks	Traditional Chinese exercise + Rhythm training group(TCE + RTG);<br>Walking group (WG)	control group: health science knowledge dissemination activities
Kevser Gursan[61]	2026	77.49	17/30	47	3 times a week	30 minutes	8 weeks	Hand exercise group (HEG):<br>putty, hand springs, hand exercise balls, water bottle transfers, bead stringing;<br>Cognitive exercise group (CEG):<br>story repetition, puzzles, serial subtraction, shape completion, visual memory exercises	Control group (CG):<br>No intervention, routine daily living activities
Mosayeb Mozafari[62]	2025	71.43	27/23	60	3 times a week	45 minutes	12 weeks	Selected smartphone video games:<br>classic sudoku, golf, archery	Control group:<br>did not do any intervention
Petri[63]	2025	81	2/18	20	2 times a week	50-60 minutes	6 weeks	The RehaCom cognitive rehabilitation software:<br>Figural Memory, Attention & Concentration, Topological Memory, Memory Strategy Training, Alertness	Control group:<br>did not receive any kind of intervention
Priya G.[64]	2025	69.18	11/11	22	1 time a week	60 minutes	2 weeks	Auditory-based cognitive training (AbCT):<br>15 cognitive training sessions	Control group:<br>did not receive any intervention
Sunyoung Kang[65]	2026	72	NR	100	2 times a week	15 minutes	12 weeks	Mobile app-based MMT program(ET-101)	Control group:<br>sham device
Jin-Hyuck Park[66]	2024	69.68	39/51	90	2 times a week	15 minutes	4 weeks	AG: virtual shopping training,VR-based cognitive training;	Wait-list control group(CG)

								SG: virtual shopping training, sham neurofeedback	
Hiroshi Hayashi[67]	2026	76.1	16/18	34	1 time a week	60 minutes	8 weeks	eSports:<br>a physical rhythm game, puzzle game, driving simulator	Control group:<br>maintain their usual daily routines
Kang[68]	2024	74.72	NR	29	2 times a week	30 minutes	8 weeks	Computerized cognitive training (CCT):<br>multidomain CCT training	Educational book reading
Rivas-Campo Y.[69]	2023	77	NR	145	3 times a week	45 minutes	12 weeks	High-intensity intervallic functional training (HIFT) program:<br>general physical activity recommendations, practiced manual activities	Control group:<br>general physical activity recommendations
Eun Hee Lim[70]	2023	74.38	7/17	34	3 times a week	30 minutes	4 weeks	Brain Talk™ serious game training:<br>home-based cognitive training program	Control group:<br>same daily activities as before
Kitsana Krootnark[71]	2024	69	19/71	90	5 times a week	15-40 minutes	12 weeks	Aerobic exercise group:<br>home-based aerobic exercise program;<br>Resistance exercise group:<br>home-based resistance training program	Control group:<br>continue their usual daily life activities
Givon Schaham[72]	2024	75.35	33/28	61	3-5 times a week	30-60 minutes	5 weeks	TECH :tablet enhancement of cognition and health	Standard care:<br>single consultation
Meixiang Fan[73]	2024	66.8	NR	120	1 time a week(the in-person training course);<br>3 times a week(computerized interaction training);<br>every day(homework)	90 minutes(the in-person training course);<br>30 minutes(computerized interaction training);<br>30 minutes(homework)	12 weeks	Multimodal intervention:<br>cognitive training, physical, healthy lifestyle education, computerized interaction training	Regular health education
Hong Yu[74]	2025	81.31	38/34	72	4-6 times a week	20-30 minutes	24 weeks	Multi-domain cognitive-motor training program:<br>TWT + repetition + categorization/enumeration + auditory clapping + calculation	Usual care control group

Supplementary Table 6. Intervention-related and methodological characteristics of the included studies.

First author	Year	Completion rate	Adherence	Supervision status	Level of social interaction	Level of cognitive engagement	Exercise intensity	Intervention dose (MET-min/week)
Lan Li[1]	2021	93.30%	NR	Yes	High	Moderate to high	Moderate	4.3 MET × 25 min × 5/week = 537.5 MET • min/week
Chang J.[2]	2021	80.10%	NR	Yes	High	Moderate to high	Moderate	5.5 MET × 30 min × 3/week = 495 MET • min/week
Lina Wang[3]	2020	95.70%	93.51%	Partial	Moderate	High	Moderate	4.3 MET × 40 min × 3/week = 516 MET • min/week
Tomoto T.[4]	2021	68.60%	69%	Partial	Low to moderate	Low to moderate	High	6.0 MET × 15-20 min × 3/week = 270-360 MET • min/week(Weeks 1-10); 6.0 MET × 20-25 min × 3-4/week = 360-600 MET • min/week(Weeks 11-25); 6.0 MET × 20-30 min × 4-5/week = 480-900 MET • min/week(Weeks 26-48)
Binu P. Thomas[5]	2020	68.60%	69%	Partial	Low	Low to moderate	High	6.0 MET × 15-20 min × 3/week = 270-360 MET • min/week (Weeks 1-10); 6.0 MET × 20-25 min × 3-4/week = 360-600 MET • min/week (Weeks 11-25); 6.0 MET × 20-30 min × 4-5/week = 480-900 MET • min/week (Weeks 26-48)
Kerime Bademli[6]	2019	100.00%	NR	Yes	High	Moderate	Moderate to high	(3.0 – 6.0 MET × 20 min × 4/week) + (3.0 MET × 40 min × 3/week) = 600 – 840 MET • min/week
Imran Amjad[7]	2018	82.50%	NR	Yes	Low	Low to moderate	High	6.0 MET × 20 – 40 min × 3/week = 360 – 720 MET • min/week
Dan Song.[8]	2019	79.20%	73.1%	Yes	High	Moderate to high	Moderate	5.5 MET × 40 min × 3/week = 660 MET • min/week
Soon-Gook Hong[9]	2017	83.90%	NR	NR	Low to moderate	Low to moderate	Moderate	3.5 MET × 40 min × 2/week = 280 MET • min/week
Yi Zhu[10]	2018	90.00%	NR	Yes	High	High	Moderate	4.8 MET × 25 min × 3/week = 360 MET • min/week
Lazarou[11]	2017	83.80%	NR	Yes	High	High	Moderate to high	3.0 – 6.0 MET × 45 min × 2/week = 270 – 540 MET • min/week
Lü[12]	2016	93.30%	NR	Yes	Moderate to high	High	Moderate	3.5 MET×50 min×3/week=525 MET • min/week
Tarumi[13]	2019	68.60%	69%	Partial	Low	Low to moderate	High	6.0 MET × 15-20 min × 3/week = 270-360 MET • min/week (Weeks 1-10);

								6.0 MET × 20-25 min × 3-4/week = 360-600 MET • min/week (Weeks 11-25);<br>6.0 MET × 20-30 min × 4-5/week = 480-900 MET • min/week (Weeks 26-48)
Yoon[14]	2017	50.00%	NR	Yes	Low to moderate<br>Low to moderate	Moderate to high<br>Low to moderate	Moderate<br>High	3.5 MET × 40 min × 2/week = 280 MET • min/week (HSPT);<br>6.0 MET×40 min×2/week=480 MET • min/week (LSST)
Thaiyanto[15]	2021	100.00%	92.08%	Yes	High	Moderate to high	Moderate	(3.5 MET × 15 min + 5.5 MET × 15 min + 3.0 MET × 15 min) × 3/week = 540 MET • min/week
Nascimento[16]	2014	100.00%	NR	Yes	High	Moderate to high	Moderate to high	3.0 – 6.0 MET × 45 min × 3/week = 405 – 810 MET • min/week
Marta Bisbe[17]	2020	86.10%	NR	Yes	High	High	Moderate	3.0 – 4.8 MET × 50 min × 2/week = 300 – 480 MET • min/week
Takehiko Doi[18]	2017	85.60%	86.0%	Yes	High<br>High	High<br>High	Moderate to high<br>Non-exercise	3.0 – 6.0 MET × 60 min × 1/week = 180 – 360 MET • min/week (Dance group);<br>NR (Music group)
Wang Shuo[19]	2019	92.40%	NR	Yes	High	Moderate to high	Moderate	5.5 MET × 30 min × 3/week = 495 MET • min/week
Rojasavastera[20]	2020	84.60%	NR	Partial	Low to moderate<br>Low to moderate	Moderate to high<br>Moderate to high	Moderate<br>Moderate	3.0 – 3.8 MET × 40 min × 2 – 3/week = 240 – 456 MET • min/week (AOGT);<br>3.0 – 3.8 MET × 40 min × 2 – 3/week = 240 – 456 MET • min/week (GT)
Parial[21]	2023	85.00%	NR	Yes	High	High	Moderate	5.5 MET × 40 min × 3/week = 660 MET • min/week
Ming Qi[22]	2019	97.00%	NR	Yes	High	High	Moderate	4.8 MET × 25 min × 3/week = 360 MET • min/week
Harris A. Eyre[23]	2017	73.90%	59.4%	Partially	Moderate	Moderate to high	Low	2.3 MET × [(39 min × 1/week) + (12 min × 7/week)] = 282.9 MET • min/week
Lam[24]	2015	76.10%	73.3%	Partially	Moderate to high<br>Moderate to high	Low to moderate<br>High	Low to high<br>Moderate	(2.3+3.3+6.8) MET×60 min×1/week=744 MET·min/week (Physical exercise);<br>3.3 MET × 60 min × 2/week = 396 MET • min/week (Cognitive-Physical activity)
Langoni[25]	2018	86.70%	NR	Yes	High	Low to moderate	Moderate	3.0 – 5.9 MET × 20 – 30 min × 2/week = 120 – 354 MET • min/week
Geoffrey Tremont[26]	2022	80.40%	NR	Yes	Moderate	Moderate to high	Low	2.3 MET × 55 min × 2/week = 253 MET • min/week

Hongyu Yang[27]	2016	NR	NR	Partially	Moderate	Moderate to high	Low	2.3 MET × (41 min × 1/week + 12 min × 7/week) = 287.5 MET • min/week
Danny J. Yu[28]	2022	74.00%	NR	Yes	Low<br>Low<br>Low<br>Low	Low to moderate<br>Low to moderate<br>Low to moderate<br>Low to moderate	Moderate<br>Moderate<br>High<br>High	3.5 MET × 150 min × 1/week = 525 MET • min/week(M1);<br>3.5 MET × 50 min × 3/week = 525 MET • min/week(M3);<br>7.0 MET × 75 min × 1/week = 525 MET • min/week(V1);<br>7.0 MET × 25 min × 3/week = 525 MET • min/week(V3)
Langoni[29]	2018	86.70%	NR	Yes	High	Low to moderate	Moderate	[(3.5 MET × 30 min) + (3.0 – 5.9 MET × 20 – 30 min)] × 2/week = 330 – 564 MET • min/week
Tsubasa Tomoto[30]	2021	52.90%	69%	Partial	Low	Low to moderate	High	6.0 MET × 15-20 min × 3/week = 270-360 MET • min/week (Weeks 1-10);<br>6.0 MET × 20-25 min × 3-4/week = 360-600 MET • min/week (Weeks 11-25);<br>6.0 MET × 20-30 min × 4-5/week = 480-900 MET • min/week (Weeks 26-48)
Donnezan[31]	2018	100.00%	NR	Yes	Moderate<br>Moderate to high<br>Moderate to high	Low to moderate<br>High<br>High	Moderate<br>Moderate<br>Non-exercise	4.5 MET × 60 min × 2/week = 540 MET • min/week (Aerobic training);<br>4.5 MET × 60 min × 2/week = 540 MET • min/week (Cognitive-Physical training);<br>NR (Cognitive Training)
Tao J. [32]	2019	82.60%	NR	Yes	High<br>Moderate to high	High<br>Low to moderate	Moderate<br>Moderate	3.3 MET × 40 min × 3/week = 396 MET • min/week (Baduanjin);<br>3.0 – 6.0 MET × 40 min × 3/week = 360 – 720 MET • min/week (Brisk walking)
Somporn Sungkarat[33]	2018	84.80%	83.6%	Partial	Low to moderate	High	Moderate	3.3 MET × 30 min × 3/week = 297 MET • min/week
Kate E. Devenney[34]	2019	100.00%	NR	Yes	Low	Low	High	6.0 MET × 9.18 – 10.42 min × 1 session = 55.1 – 62.5 MET • min/session
Kohanpour[35]	2017	100.00%	NR	NR	Low to moderate<br>Low to moderate<br>Low	Low<br>Low<br>Low	High<br>High<br>Non-exercise	6.0 MET × 8 – 26 min × 3/week = 144 – 468 MET • min/week (Aerobic exercise);<br>6.0 MET × 8 – 26 min × 3/week = 144 – 468 MET • min/week (Aerobic exercise + Lavender);

								NR(Lavender)
Fuzhong Li[36]	2022	94.20%	NR	Yes	High	High	Moderate	3.24 MET × 45–55 min × 2/week = 291.6–356.4 MET • min/week
Angus P. Yu[37]	2022	91.90%	NR	Yes	High<br>High	High<br>Low to moderate	Moderate<br>Moderate	3.24 MET × 40 min × 3/week = 388.8 MET • min/week (Tai Chi);<br>(4.3+5.5) MET × 20 min × 3/week = 588 MET·min/week (Conventional exercise group)
Chien-Liang Liu[38]	2022	92.60%	NR	Yes	Moderate to high<br>Moderate to high	High<br>High	Moderate<br>Moderate	3.24 MET × 35 min × 3/week = 340.2 MET •min/week (Exergaming-based Tai Chi);<br>3.24 MET × 35 min × 3/week = 340.2 MET • min/week (Traditional Tai Chi)
Somporn Sungkarat[39]	2016	89.40%	87.5%	Partial	Low to moderate	High	Moderate	3.24 MET × 30 min × 3/week = 291.6 MET • min/week
Mei-yi Siu[40]	2018	90.60%	NR	Yes	High	High	Moderate	3.24 MET × <60 min × 2/week = <388.8 MET • min/week
Phoemsapthawee J.[41]	2016	100.00%	NR	Yes	Moderate to high	Moderate	Low	2.0–2.9 MET × 10–30 min × 5/week = 100–435 MET • min/week
Jurakic[42]	2017	100.00%	NR	NR	Low to moderate	High	Moderate	3.5 MET × 30 min × 3/week = 315 MET • min/week
Scherder[43]	2005	100.00%	NR	Yes	Low<br>Low	Low to moderate<br>Moderate	Low to moderate<br>Low	2.1–3.5 MET × 30 min × 3/week = 189–315 MET • min/week (Walking group);<br>2.3–2.8 MET × 30 min × 3/week = 207–252 MET • min/week (Hand and face exercises)
Silvia Varela[44]	2011	70.60%	NR	Yes	Low<br>Low	Low to moderate<br>Low to moderate	Moderate<br>High	3.0 MET × 20 min × 3/week = 180 MET • min/week (40% of heart rate reserve);<br>6.0 MET × 20 min × 3/week = 360 MET •min/week (60% of heart rate reserve)
Takao Suzuki[45]	2012	94.00%	79.2%	Yes	High	High	Moderate	[(3.5 MET × 20 min) + (3.0–5.9 MET × 60 min)] × 2/week = 500–848 MET • min/week
Xiu-hong Wei[46]	2014	100.00%	NR	Yes	High	High	Moderate	3.0 MET × 30 min × 5/week = 450 MET • min/week
Montero-Odasso[47]	2023	82.30%	NR	Yes	Moderate<br>Moderate<br>Moderate<br>Moderate	High<br>High<br>Low to moderate<br>Low to moderate	Moderate<br>Moderate<br>Moderate<br>Moderate	3.5–5.9 MET × 60 min × 3/week = 630–1062 MET • min/week (Arm 1);<br>3.5–5.9 MET × 60 min × 3/week = 630–1062 MET • min/week (Arm 2);<br>3.5–5.9 MET × 60 min × 3/week = 630–1062 MET • min/week (Arm 3);<br>3.5–5.9 MET × 60 min × 3/week = 630–1062 MET • min/week (Arm 4)

Jorge Buele[48]	2024	76.50%	NR	Yes	Moderate to high	High	Low to moderate	2.0 – 3.5 MET × <40 min × 2/week = <160 – 280 MET • min/week
Carvalho[49]	2025	83.30%	NR	Yes	Low	High	Non-exercise	NR
Miaoran Lin[50]	2024	96.00%	NR	Yes	High	High	Low to moderate	3.3 MET × 60 min × 5/week = 990 MET • min/week
So Young Moon[51]	2025	84.30%	84.7%	Yes	High	High	Moderate	3.0 – 5.9 MET × 50 min × 3/week = 450 – 885 MET • min/week
Chunhui Zhou[52]	2025	100.00%	NR	NR	Low to moderate	Moderate to high	Moderate to high	3.0 – 6.0 MET × 45 – 50 min × f/week = 135f – 300f MET • min/week. f = NR(TCMG)
					Moderate	High	Moderate to high	3.0 – 6.0 MET × 45 – 50 min × f/week = 135f – 300f MET • min/week. f = NR(TCG)
Ha Yeong Choi[53]	2025	88.80%	NR	Yes	Low to moderate	Low to moderate	NR	NR
Cheung[54]	2024	85.70%	NR	Yes	Moderate to high	Moderate to high	Low	NR
Alessandro Sale[55]	2023	91.20%	NR	Yes	High	High	Moderate	3.0 – 5.9 MET × 60 min × 3/week = 540 – 1062 MET • min/week
Jingsong Wu[56]	2023	95.00%	NR	Yes	Low	High	Non-exercise	NR
Elmar Graessel[57]	2024	89.90%	NR	No	Low	High	Non-exercise	NR
Goumopoulos[58]	2023	95.20%	NR	Yes	Low to moderate	High	Non-exercise	NR
İsmail Uysal[59]	2022	100.00%	NR	Yes	Low to moderate	Low to moderate	Moderate	2.3 – 3.5 MET × 30 min × 3/week = 207 – 315 MET • min/week (aerobic exercise training) +3.5 MET × t min × 3/week (lower limb strengthening). t = NR(AG);
					Moderate	High	Low to moderate	2.3 – 3.5 MET × 30 min × 3/week = 207 – 315 MET • min/week (dual-task training) +3.5 MET × t min × 3/week (lower limb strengthening). t = NR(DG);
					Moderate	High	Moderate	(2.0 – 5.9 MET × 20 min × 3/week) + (2.3 – 3.5 MET × 30 min × 3/week) = 327 – 669 MET • min/week (aerobic exercise training+dual-task training) +3.5 MET × t min × 3/week (lower limb strengthening). t = NR(ADG)
Qiang Zhang[60]	2023	100.00%	NR	Yes	Moderate to high	High	Low to moderate	(3.0 MET × 30 min + 2.0 MET × 10 min) × 3/week = 330 MET • min/week
					Moderate to high	Low to moderate	Low	(TCE + RTG);

Kevser Gursan[61]	2026	92.20%	NR	Yes	Low<br>Low to moderate	Moderate<br>High	Low to moderate<br>Non-exercise	2.8 MET × 60 min × 3/week = 504 MET • min/week (WG)<br>2.3 – 3.0 MET × 30 min × 3/week = 207 – 270 MET • min/week (HEG);<br>NR(CEG)
Mosayeb Mozafari[62]	2025	100.00%	NR	Yes	Low	High	Non-exercise	NR
Petri[63]	2025	100.00%	NR	Yes	Low to moderate	High	Non-exercise	NR
Priya G.[64]	2025	100.00%	NR	Yes	Low to moderate	High	Non-exercise	NR
Sunyoung Kang[65]	2026	89.00%	83.2%	Yes	Low	High	Non-exercise	NR
Jin-Hyuck Park[66]	2024	100.00%	NR	Yes	Low to moderate<br>Low to moderate	High<br>High	Non-exercise<br>Non-exercise	NR(AG)<br>NR(SG)
Hiroshi Hayashi[67]	2026	100.00%	NR	Yes	High	Moderate to high	Non-exercise	NR
Kang[68]	2024	93.10%	NR	Partial	Low	High	Non-exercise	NR
Rivas-Campo Y.[69]	2023	91.00%	NR	Yes	Moderate to high	Moderate	Low to high	{[2.0 – 2.9 MET × 25 min/session × 3/week × 2 weeks] + [[(6.0 MET × 16 min/session) + (2.0 – 2.9 MET × 9 min/session)] × 3/week × 1 week] + [[(6.0 MET × 16 min/session) + (2.0 – 5.9 MET × 9 min/session)] × 3/week × 9 weeks]} ÷ 12 weeks = 310 – 402.25 MET • min/week
Eun Hee Lim[70]	2023	100.00%	NR	Yes	Low	High	Non-exercise	NR
Kitsana Krootnark[71]	2024	100.00%	94.83%	Yes	Low<br>Low	Low to moderate<br>Low to moderate	Low to moderate<br>Low to moderate	2.0 – 3.0 MET × 15 – 40 min × 5/week = 150 – 600 MET • min/week (Aerobic exercise group);<br>2.3 – 3.5 MET × 15 – 40 min × 5/week = 172.5 – 700 MET • min/week (Resistance exercise group)
Givon Schaham[72]	2024	82.00%	96.67%	Partial	Moderate	High	Non-exercise	NR
Meixiang Fan[73]	2024	96.70%	NR	Yes	Moderate to high	High	Low to moderate	{[2.0 – 3.5 MET × ((15 min/session × 1/week) + (30 min/session × 5/week)) × 6 weeks] + [2.0 – 3.5 MET × 30 min/session × 5/week × 6 weeks]} ÷ 12 weeks = 315 – 551.25 MET • min/week
Hong Yu[74]	2025	65.30%	NR	Yes	Moderate	High	Low to moderate	2.3 – 3.5 MET × 20 – 30 min × 6 week = 276 – 630 MET • min/week

REFERENCES

[1] Li L, Liu M, Zeng H, et al. Multi-component exercise training improves the physical and cognitive function of the elderly with mild cognitive impairment: a six-month randomized controlled trial[J]. Ann Palliat Med, 2021, 10(8): 8919-8929. DOI:10.21037/apm-21-1809.

[2] Chang J, Zhu W, Zhang J, et al. The Effect of Chinese Square Dance Exercise on Cognitive Function in Older Women With Mild Cognitive Impairment: The Mediating Effect of Mood Status and Quality of Life[J]. Front Psychiatry, 2021, 12: 711079. DOI:10.3389/fpsyt.2021.711079.

[3] Wang L, Wu B, Tao H, et al. Effects and mediating mechanisms of a structured limbs-exercise program on general cognitive function in older adults with mild cognitive impairment: A randomized controlled trial[J]. Int J Nurs Stud, 2020, 110: 103706. DOI:10.1016/j.ijnurstu.2020.103706.

[4] Tomoto T, Liu J, Tseng B Y, et al. One-Year Aerobic Exercise Reduced Carotid Arterial Stiffness and Increased Cerebral Blood Flow in Amnestic Mild Cognitive Impairment[J]. J Alzheimers Dis, 2021, 80(2): 841-853. DOI:10.3233/JAD-201456.

[5] Thomas B P, Tarumi T, Sheng M, et al. Brain Perfusion Change in Patients with Mild Cognitive Impairment After 12 Months of Aerobic Exercise Training[J]. J Alzheimers Dis, 2020, 75(2): 617-631. DOI:10.3233/JAD-190977.

[6] Bademli K, Lok N, Canbaz M, et al. Effects of Physical Activity Program on cognitive function and sleep quality in elderly with mild cognitive impairment: A randomized controlled trial[J]. Perspect Psychiatr Care, 2019, 55(3): 401-408. DOI:10.1111/ppc.12324.

[7] Amjad I, Toor H, Niazi I K, et al. Therapeutic effects of aerobic exercise on EEG parameters and higher cognitive functions in mild cognitive impairment patients[J]. Int J Neurosci, 2019, 129(6): 551-562. DOI:10.1080/00207454.2018.1551894.

[8] Song D, Yu D S F. Effects of a moderate-intensity aerobic exercise programme on the cognitive function and quality of life of community-dwelling elderly people with mild cognitive impairment: A randomised controlled trial[J]. Int J Nurs Stud, 2019, 93: 97-105. DOI:10.1016/j.ijnurstu.2019.02.019.

[9] Hong S G, Kim J H, Jun T W. Effects of 12-Week Resistance Exercise on Electroencephalogram Patterns and Cognitive Function in the Elderly With Mild Cognitive Impairment: A Randomized Controlled Trial[J]. Clin J Sport Med, 2018, 28(6): 500-508. DOI:10.1097/JSM.0000000000000476.

[10] Zhu Y, Wu H, Qi M, et al. Effects of a specially designed aerobic dance routine on mild cognitive impairment[J]. Clin Interv Aging, 2018, Volume 13: 1691-1700. DOI:10.2147/CIA.S163067.

[11] Lazarou I, Parastatidis T, Tsolaki A, et al. International Ballroom Dancing Against Neurodegeneration: A Randomized Controlled Trial in Greek Community-Dwelling Elders With Mild Cognitive impairment[J]. Am J Alzheimers Dis Dementiasr, 2017, 32(8): 489-499. DOI:10.1177/1533317517725813.

[12] Lü J, Sun M, Liang L, et al. Effects of momentum-based dumbbell training on cognitive function in older adults with mild cognitive impairment: a pilot randomized controlled trial[J]. Clin Interv Aging, 2015: 9. DOI:10.2147/CIA.S96042.

[13] Tarumi T, Rossetti H, Thomas B P, et al. Exercise Training in Amnestic Mild Cognitive

Impairment: A One-Year Randomized Controlled Trial[J]. J Alzheimers Dis, 2019, 71(2): 421-433. DOI:10.3233/JAD-181175.

[14] Yoon D H, Kang D, Kim H, et al. Effect of elastic band-based high-speed power training on cognitive function, physical performance and muscle strength in older women with mild cognitive impairment[J]. Geriatr Gerontol Int, 2017, 17(5): 765-772. DOI:10.1111/ggi.12784.

[15] Thaiyanto J, Sittichoke C, Phirom K, et al. Effects of Multicomponent Exercise on Cognitive Performance and Fall Risk in Older Women with Mild Cognitive Impairment[J]. J Nutr Health Aging, 2021, 25(2): 160-164. DOI:10.1007/s12603-020-1458-5.

[16] Nascimento C M C, Pereira J R, Pires De Andrade L, et al. Physical Exercise Improves Peripheral BDNF Levels and Cognitive Functions in Mild Cognitive Impairment Elderly with Different BDNF Val66Met Genotypes[J]. J Alzheimers Dis, 2014, 43(1): 81-91. DOI:10.3233/JAD-140576.

[17] Bisbe M, Fuente-Vidal A, López E, et al. Comparative Cognitive Effects of Choreographed Exercise and Multimodal Physical Therapy in Older Adults with Amnestic Mild Cognitive Impairment: Randomized Clinical Trial[J]. J Alzheimer's Dis, 2020, 73(2): 769-783. DOI:10.3233/JAD-190552.

[18] Doi T, Verghese J, Makizako H, et al. Effects of Cognitive Leisure Activity on Cognition in Mild Cognitive Impairment: Results of a Randomized Controlled Trial[J]. J Am Med Dir Assoc, 2017, 18(8): 686-691. DOI:10.1016/j.jamda.2017.02.013.

[19] Wang S, Yin H, Meng X, et al. Effects of Chinese square dancing on older adults with mild cognitive impairment[J]. Geriatr Nur (Lond), 2020, 41(3): 290-296. DOI:10.1016/j.gerinurse.2019.10.009.

[20] Rojasavastera R, Bovonsunthonchai S, Hiengkaew V, et al. Action observation combined with gait training to improve gait and cognition in elderly with mild cognitive impairment A randomized controlled trial[J]. Dement Neuropsychol, 2020, 14(2): 118-127. DOI:10.1590/1980-57642020dn14-020004.

[21] Parial L L, Kor P P K, Sumile E F, et al. Dual-Task Zumba Gold for Improving the Cognition of People With Mild Cognitive Impairment: A Pilot Randomized Controlled Trial[J]. The Gerontologist, 2023, 63(7): 1248-1261. DOI:10.1093/geront/gnac081.

[22] Qi M, Zhu Y, Zhang L, et al. The effect of aerobic dance intervention on brain spontaneous activity in older adults with mild cognitive impairment: A resting-state functional MRI study[J]. Exp Ther Med, 2018. DOI:10.3892/etm.2018.7006.

[23] Eyre H A, Siddarth P, Acevedo B, et al. A randomized controlled trial of Kundalini yoga in mild cognitive impairment[J]. Int Psychogeriatr, 2017, 29(4): 557-567. DOI:10.1017/S1041610216002155.

[24] Lam L C wa, Chan W C, Leung T, et al. Would Older Adults with Mild Cognitive Impairment Adhere to and Benefit from a Structured Lifestyle Activity Intervention to Enhance Cognition?: A Cluster Randomized Controlled Trial[J]. PLOS ONE, 2015, 10(3): e0118173. DOI:10.1371/journal.pone.0118173.

[25] Langoni C D S, Resende T D L, Barcellos A B, et al. The effect of group exercises on balance, mobility, and depressive symptoms in older adults with mild cognitive impairment: a randomized controlled trial[J]. Clin Rehabil, 2019, 33(3): 439-449. DOI:10.1177/0269215518815218.

[26] Tremont G, Davis J, Ott B R, et al. Feasibility of a Yoga Intervention for Individuals with

Mild Cognitive Impairment: A Randomized Controlled Trial[J]. J Integr Complement Med, 2022, 28(3): 250-260. DOI:10.1089/jicm.2021.0204.
[27] Yang H, Leaver A M, Siddarth P, et al. Neurochemical and Neuroanatomical Plasticity Following Memory Training and Yoga Interventions in Older Adults with Mild Cognitive Impairment[J]. Front Aging Neurosci, 2016, 8. DOI:10.3389/fnagi.2016.00277.
[28] Yu D J, Yu A P, Bernal J D K, et al. Effects of exercise intensity and frequency on improving cognitive performance in middle-aged and older adults with mild cognitive impairment: A pilot randomized controlled trial on the minimum physical activity recommendation from WHO[J]. Front Physiol, 2022, 13: 1021428. DOI:10.3389/fphys.2022.1021428.
[29] Langoni C D S, Resende T D L, Barcellos A B, et al. Effect of Exercise on Cognition, Conditioning, Muscle Endurance, and Balance in Older Adults With Mild Cognitive Impairment: A Randomized Controlled Trial[J]. J Geriatr Phys Ther, 2019, 42(2): E15-E22. DOI:10.1519/JPT.0000000000000191.
[30] Tomoto T, Tarumi T, Chen J N, et al. One-year aerobic exercise altered cerebral vasomotor reactivity in mild cognitive impairment[J]. J Appl Physiol, 2021, 131(1): 119-130. DOI:10.1152/japplphysiol.00158.2021.
[31] Combourieu Donnezan L, Perrot A, Belleville S, et al. Effects of simultaneous aerobic and cognitive training on executive functions, cardiovascular fitness and functional abilities in older adults with mild cognitive impairment[J]. Ment Health Phys Act, 2018, 15: 78-87. DOI:10.1016/j.mhpa.2018.06.001.
[32] Tao J, Liu J, Chen X, et al. Mind-body exercise improves cognitive function and modulates the function and structure of the hippocampus and anterior cingulate cortex in patients with mild cognitive impairment[J]. NeuroImage Clin, 2019, 23: 101834. DOI:10.1016/j.nicl.2019.101834.
[33] Sungkarat S, Boripuntakul S, Kumfu S, et al. Tai Chi Improves Cognition and Plasma BDNF in Older Adults With Mild Cognitive Impairment: A Randomized Controlled Trial[J]. Neurorehabil Neural Repair, 2018, 32(2): 142-149. DOI:10.1177/1545968317753682.
[34] Devenney K E, Guinan E M, Kelly Á M, et al. Acute high-intensity aerobic exercise affects brain-derived neurotrophic factor in mild cognitive impairment: a randomised controlled study[J]. BMJ Open Sport Exerc Med, 2019, 5(1): e000499. DOI:10.1136/bmjsem-2018-000499.
[35] Kohanpour M A, Peeri M, Azarbayjani M A. The effects of aerobic exercise with lavender essence use on cognitive state and serum brain-derived neurotrophic factor levels in elderly with mild cognitive impairment[J]. J HerbMed Pharmacol, 2017, 6(2): 80-84.
[36] Li F, Harmer P, Fitzgerald K, et al. A cognitively enhanced online Tai Ji Quan training intervention for community-dwelling older adults with mild cognitive impairment: A feasibility trial[J]. BMC Geriatr, 2022, 22(1): 76. DOI:10.1186/s12877-021-02747-0.
[37] Yu A P, Chin E C, Yu D J, et al. Tai Chi versus conventional exercise for improving cognitive function in older adults: a pilot randomized controlled trial[J]. Sci Rep, 2022, 12(1): 8868. DOI:10.1038/s41598-022-12526-5.
[38] Liu C L, Cheng F Y, Wei M J, et al. Effects of Exergaming-Based Tai Chi on Cognitive Function and Dual-Task Gait Performance in Older Adults With Mild Cognitive Impairment: A Randomized Control Trial[J]. Front Aging Neurosci, 2022, 14: 761053. DOI:10.3389/fnagi.2022.761053.

[39] Sungkarat S, Boripuntakul S, Chattipakorn N, et al. Effects of Tai Chi on Cognition and Fall Risk in Older Adults with Mild Cognitive Impairment: A Randomized Controlled Trial[J]. J Am Geriatr Soc, 2017, 65(4): 721-727. DOI:10.1111/jgs.14594.

[40] Siu M yi, Lee D T F. Effects of tai chi on cognition and instrumental activities of daily living in community dwelling older people with mild cognitive impairment[J]. BMC Geriatr, 2018, 18(1): 37. DOI:10.1186/s12877-018-0720-8.

[41] Phoemsapthawee J, Ammawat W, Leelayuwat N. The Benefit of Arm Swing Exercise on Cognitive Performance in Older Women with Mild Cognitive Impairment[J].

[42] Greblo Jurakic Z, Krizanic V, Sarabon N, et al. Effects of feedback-based balance and core resistance training vs. Pilates training on cognitive functions in older women with mild cognitive impairment: a pilot randomized controlled trial[J]. Aging Clin Exp Res, 2017, 29(6): 1295-1298. DOI:10.1007/s40520-017-0740-9.

[43] Scherder E J A, Van Paasschen J, Deijen J B, et al. Physical activity and executive functions in the elderly with mild cognitive impairment[J]. Aging Ment Health, 2005, 9(3): 272-280. DOI:10.1080/13607860500089930.

[44] Varela S, Ayán C, Cancela J M, et al. Effects of two different intensities of aerobic exercise on elderly people with mild cognitive impairment: a randomized pilot study[J]. Clin Rehabil, 2012, 26(5): 442-450. DOI:10.1177/0269215511425835.

[45] Suzuki T, Shimada H, Makizako H, et al. Effects of multicomponent exercise on cognitive function in older adults with amnestic mild cognitive impairment: a randomized controlled trial[J]. BMC Neurol, 2012, 12(1): 128. DOI:10.1186/1471-2377-12-128.

[46] Wei X hong, Ji L li. Effect of handball training on cognitive ability in elderly with mild cognitive impairment[J]. Neurosci Lett, 2014, 566: 98-101. DOI:10.1016/j.neulet.2014.02.035.

[47] Montero-Odasso M, Zou G, Speechley M, et al. Effects of Exercise Alone or Combined With Cognitive Training and Vitamin D Supplementation to Improve Cognition in Adults With Mild Cognitive Impairment: A Randomized Clinical Trial[J]. JAMA Netw Open, 2023, 6(7): e2324465. DOI:10.1001/jamanetworkopen.2023.24465.

[48] Buele J, Avilés-Castillo F, Del-Valle-Soto C, et al. Effects of a dual intervention (motor and virtual reality-based cognitive) on cognition in patients with mild cognitive impairment: a single-blind, randomized controlled trial[J]. J NeuroEngineering Rehabil, 2024, 21(1): 130. DOI:10.1186/s12984-024-01422-w.

[49] Carvalho C M, Poltronieri B C, Reuwsaat K, et al. Digital cognitive training for functionality in mild cognitive impairment: a randomized controlled clinical trial[J]. GeroScience, 2025, 47(3): 5111-5121. DOI:10.1007/s11357-024-01464-x.

[50] Lin M, Liu W, Ma C, et al. Tai Chi-Induced Exosomal LRP1 is Associated With Memory Function and Hippocampus Plasticity in aMCI Patients[J]. Am J Geriatr Psychiatry, 2024, 32(10): 1215-1230. DOI:10.1016/j.jagp.2024.04.012.

[51] Moon S Y, Park Y K, Jeong J H, et al. South Korean study to prevent cognitive impairment and protect brain health through multidomain interventions via face-to-face and video communication platforms in mild cognitive impairment (SUPERBRAIN-MEET): A randomized controlled trial[J]. Alzheimers Dement, 2025, 21(2): e14517. DOI:10.1002/alz.14517.

[52] Zhou C. Effect of Tai Chi combined with music therapy on the cognitive function in older

adult individuals with mild cognitive impairment[J]. Front Public Health, 2025, 13: 1475863. DOI:10.3389/fpubh.2025.1475863.

[53] Choi H Y, Kim Y J, Son D S, et al. Effectiveness of a 52-week multidomain intervention to maintain cognitive function in patients with mild cognitive impairment: a randomized controlled trial[J]. Sci Rep, 2025, 15(1): 41141. DOI:10.1038/s41598-025-24865-0.

[54] Cheung M C, Sze S L, Chan A S. *Chanwuyi* Lifestyle Medicine Program Improves Memory and Executive Functions of Older Adults With Mild Cognitive Impairment[J]. Am J Alzheimers Dis Dementias®, 2024, 39: 15333175241255744. DOI:10.1177/15333175241255744.

[55] Sale A, Noale M, Cintoli S, et al. Long-term beneficial impact of the randomised trial 'Train the Brain', a motor/cognitive intervention in mild cognitive impairment people: effects at the 14-month follow-up[J]. Age Ageing, 2023, 52(5): afad067. DOI:10.1093/ageing/afad067.

[56] Wu J, He Y, Liang S, et al. Computerized Cognitive Training Enhances Episodic Memory by Down-Modulating Posterior Cingulate-Precuneus Connectivity in Older Persons With Mild Cognitive Impairment: A Randomized Controlled Trial[J]. Am J Geriatr Psychiatry, 2023, 31(10): 820-832. DOI:10.1016/j.jagp.2023.04.008.

[57] Graessel E, Jank M, Scheerbaum P, et al. Individualised computerised cognitive training (iCCT) for community-dwelling people with mild cognitive impairment (MCI): results on cognition in the 6-month intervention period of a randomised controlled trial (MCI-CCT study)[J]. BMC Med, 2024, 22(1): 472. DOI:10.1186/s12916-024-03647-x.

[58] Goumopoulos C, Skikos G, Frounta M. Feasibility and Effects of Cognitive Training with the COGNIPLAT Game Platform in Elderly with Mild Cognitive Impairment: Pilot Randomized Controlled Trial[J]. Games Health J, 2023, 12(5): 414-425. DOI:10.1089/g4h.2023.0029.

[59] Uysal İ, Başar S, Aysel S, et al. Aerobic exercise and dual-task training combination is the best combination for improving cognitive status, mobility and physical performance in older adults with mild cognitive impairment[J]. Aging Clin Exp Res, 2022, 35(2): 271-281. DOI:10.1007/s40520-022-02321-7.

[60] Zhang Q, Zhu M, Huang L, et al. A Study on the Effect of Traditional Chinese Exercise Combined With Rhythm Training on the Intervention of Older Adults With Mild Cognitive Impairment[J]. Am J Alzheimers Dis Dementias®, 2023, 38: 15333175231190626. DOI:10.1177/15333175231190626.

[61] Gursan K, Bayar K. Investigation of the effects of different rehabilitation approaches in elderly individuals with mild cognitive impairment[J]. BMC Geriatr, 2026, 26(1): 185. DOI:10.1186/s12877-025-06946-x.

[62] Mozafari M, Otaghi M, Pakseresht M, et al. Effect of Video Games on Cognitive Performance and Problem-Solving Ability in the Aged with Cognitive Dysfunction: A Randomized Clinical Trial[J]. Iran J Med Sci, 2025, 50(2). DOI:10.30476/ijms.2024.101861.3452.

[63] Petri M C, Messinis L, Patrikelis P, et al. Feasibility and Clinical Effectiveness of Computer-Based Cognitive Rehabilitation in Illiterate and Low-Educated Individuals with Mild Cognitive Impairment: Preliminary Data[J]. Arch Clin Neuropsychol, 2025, 40(3): 382-393. DOI:10.1093/arclin/acae078.

[64] G P, MM K, R V, et al. Influence of auditory-based cognitive training on auditory resolution, executive function, and working memory skills in individuals with mild cognitive impairment

– a pilot randomized controlled study[J]. F1000Research, 2025, 13: 1022. DOI:10.12688/f1000research.152775.2.

[65] Kang S, Lim J I, Stenzel L, et al. Efficacy and Safety of Mobile App–Based Metamemory Cognitive Training for Mild Cognitive Impairment: Multicenter Randomized Clinical Trial[J]. JMIR MHealth UHealth, 2026, 14: e73464. DOI:10.2196/73464.

[66] Park J H. Is virtual reality-based cognitive training in parallel with functional near-infrared spectroscopy-derived neurofeedback beneficial to improve cognitive function in older adults with mild cognitive impairment?[J]. Disabil Rehabil, 2025, 47(7): 1717-1724. DOI:10.1080/09638288.2024.2380483.

[67] Hayashi H, Sone T, Iokawa K, et al. Effects of Electronic Sports on Community-Dwelling Older Adults With Mild Cognitive Impairment: A Randomized Controlled Study[J]. Geriatr Gerontol Int, 2026, 26(1): e70319. DOI:10.1111/ggi.70319.

[68] Kang J M, Kim N, Yun S K, et al. Exploring transfer effects on memory and its neural mechanisms through a computerized cognitive training in mild cognitive impairment: randomized controlled trial[J]. Psychogeriatrics, 2024, 24(5): 1075-1086. DOI:10.1111/psyg.13161.

[69] Rivas-Campo Y, Aibar-Almazán A, Rodríguez-López C, et al. Enhancing Cognition in Older Adults with Mild Cognitive Impairment through High-Intensity Functional Training: A Single-Blind Randomized Controlled Trial[J]. J Clin Med, 2023, 12(12): 4049. DOI:10.3390/jcm12124049.

[70] Lim E H, Kim D S, Won Y H, et al. Effects of Home Based Serious Game Training (Brain Talk^{TM}) in the Elderly With Mild Cognitive Impairment: Randomized, a Single-Blind, Controlled Trial[J]. Brain Neurorehabilitation, 2023, 16(1): e4. DOI:10.12786/bn.2023.16.e4.

[71] Krootnark K, Chaikeeree N, Saengsirisuwan V, et al. Effects of low-intensity home-based exercise on cognition in older persons with mild cognitive impairment: a direct comparison of aerobic versus resistance exercises using a randomized controlled trial design[J]. Front Med, 2024, 11: 1392429. DOI:10.3389/fmed.2024.1392429.

[72] Givon Schaham N, Buckman Z, Rand D. TECH preserves global cognition of older adults with MCI compared with a control group: a randomized controlled trial[J]. Aging Clin Exp Res, 2024, 36(1): 1. DOI:10.1007/s40520-023-02659-6.

[73] Fan M, Li Q, Yang T, et al. Effect of Multimodal Intervention in Individuals with Mild Cognitive Impairment: A Randomized Clinical Trial in Shanghai[J]. J Alzheimer's Dis, 2024, 101(1): 235-248. DOI:10.3233/JAD-231370.

[74] Yu H, Ma B X, Feng Y C, et al. Effects of multi-domain cognitive-motor training in older adults with amnestic mild cognitive impairment: A randomized controlled trial[J]. Geriatr Nur (Lond), 2025, 61: 64-72. DOI:10.1016/j.gerinurse.2024.10.072.

Supplementary Table 7. Baseline characteristics of the included outcome measures.

First author	Year	MoCA	MMSE	ADAS-Cog	TMT-B	DST	SCWT	DRA	MFA	Group
Lan Li	2021	26.50±1.33	21.52±2.05	NR	NR	NR	NR	NR	NR	
Chang J.	2021	21.61 ± 2.11	NR	NR	NR	NR	NR	NR	4.97 ± 1.41	
Lina Wang	2020	21.65 ± 2.22	NR	NR	NR	NR	NR	NR	5.59 ± 3.04	
Tomoto T.	2021	NR	28.5 ± 1.50	NR	NR	NR	NR	45.7 ± 11.33	NR	
Binu P. Thomas	2020	NR	NR	NR	NR	NR	NR	46.6 ± 9.48	NR	
Kerime Bademli	2019	NR	23.27 ± 2.17	NR	NR	NR	NR	NR	NR	
Imran Amjad	2018	NR	NR	NR	NR	NR	NR	NR	NR	
Dan Song.	2019	22.03 ± 1.81	NR	NR	NR	NR	NR	NR	5.33 ± 3.48	
Soon-Gook Hong	2017	20.70 ± 3.46	NR	NR	NR	12.70 ± 4.71	3.90 ± 0.99	8.10 ± 2.88	NR	
Yi Zhu	2018	23.2±1.9	NR	NR	200±73	NR	16.8±2.7	14.3±5.4	12.3±7.2	
Lazarou	2017	24.70 ± 2.25	27.60 ± 2.19	NR	NR	NR	NR	40.45 ± 12.29	2.73 ± 2.69	
Lü	2016	NR	NR	10.23 ± 3.13	NR	NR	7.27 ± 1.38	NR	NR	
Tarumi	2019	NR	NR	NR	NR	NR	NR	44.3 ± 11.18	NR	
Yoon	2017	16.44 ± 4.22	NR	NR	NR	NR	NR	NR	NR	
Thaiyanto	2021	NR	NR	8.15 ± 3.38	329.86 ± 112.79	NR	NR	NR	NR	
Nascimento	2014	21.8 ± 3.8	NR	NR	NR	NR	NR	NR	NR	
Marta Bisbe	2020	NR	NR	NR	239.12 ± 118.15	NR	NR	17.18 ± 3.00	6.59 ± 3.00	
Takehiko Doi	2017	NR	26.0 ± 2.6	NR	41.1 ± 14.2	NR	NR	NR	NR	
Wang Shuo	2019	19.39 ± 3.00	25.03 ± 2.01	NR	NR	NR	NR	NR	4.88 ± 2.85	
Rojasavastera	2020	22.18 ± 1.47	NR	NR	NR	NR	NR	NR	NR	AOGT
		22.64 ± 1.36								GT
Parial	2023	20.67 ± 1.88	NR	NR	226.28 ± 56.54	NR	5.57 ± 1.76	NR	3.63 ± 2.09	

Ming Qi	2019	22.6 ± 2.1	NR	NR	190.6 ± 59.2	NR	16.4 ± 2.9	14.1 ± 5.9(	NR	
Harris A. Eyre	2017	NR	NR	NR	112.26 ± 60.09	37.41 ± 11.62	NR	17.19 ± 9.53	7.70 ± 6.57	
Lam	2015	NR	25.8±2.3	11.7±3.3	NR	NR	NR	NR	NR	Physical exercise
				11.3±3.2						Cognitive activity
				11.6±3.4						Cognitive-Physical activity
Langoni	2018	NR	NR	NR	NR	NR	NR	NR	3.93 ± 3.11	
Geoffrey Tremont	2022	NR	NR	NR	NR	NR	NR	NR	10.61 ± 9.82	
Hongyu Yang	2016	NR	NR	NR	NR	NR	NR	NR	7.5 ± 5.1	
Danny J. Yu	2022	18.9 ± 1.9	NR	NR	NR	NR	NR	NR	10.9 ± 1.9	3 times moderate-intensity
Langoni	2018	NR	21.9 ± 4.8	NR	NR	NR	NR	NR	NR	
Tsubasa Tomoto	2021	NR	NR	NR	NR	NR	NR	45.2 ± 9.3	NR	
Donnezan	2018	NR	NR	NR	NR	26.52 ± 6.80	5.48 ± 0.88	NR	NR	Cognitive and Physical training
						28.89 ± 6.45	NR			physical training
						27.19 ± 8.82	5.18 ± 0.91			Cognitive training
Tao J.	2019	NR	NR	22.45 ± 2.16	NR	NR	NR	NR	NR	Baduanjin
				21.47 ± 2.27						Brisk walking
Somporn Sungkarat	2018	NR	NR	NR	NR	NR	12.4 ± 2.8	NR	NR	
Kate E. Devenney	2019	NR	NR	NR	NR	66.42 ± 28.51	NR	NR	NR	
Kohanpour	2017	NR	22.70 ± 1.63	NR	NR	NR	NR	NR	NR	Aerobic exercise
Fuzhong Li	2022	NR	NR	NR	95.36 ± 8.08	NR	10.57 ± 1.41	NR	NR	Cognitive-enhanced Tai Ji Quan
		25.09 ± 2.43			95.38 ± 11.68		10.64 ± 1.05			Standard Tai Ji Quan

Angus P. Yu	2022	19.7 ± 1.5	NR	NR	60.0 ± 30.1	NR	7.7 ± 0.7	NR	NR	Tai Chi
					57.1 ± 38.3		7.5 ± 1.0			Aerobic exercise +Tai Chi
Chien-Liang Liu	2022	NR	NR	22.6 ± 2.5	197.3 ± 54.9	21.6 ± 10.0	NR	NR	NR	EXER-TC
				21.8 ± 3.6	204.5 ± 78.3	22.6 ± 11.6				TC
Somporn Sungkarat	2016	NR	NR	NR	NR	NR	12.4 ± 2.8	NR	NR	
Mei-yi Siu	2018	NR	25.46 ± 1.89	NR	NR	NR	NR	NR	NR	
Phoemsapthawee J.	2016	NR	18.8 ± 4.0	NR	NR	NR	NR	NR	NR	
Jurakic	2017	23.43 ± 1.70	NR	NR	NR	NR	NR	NR	NR	
Scherder	2005	NR	NR	NR	NR	NR	10.47 ± 3.04	NR	NR	Walking
Silvia Varela	2011	NR	20.81 ± 4.69	NR	NR	NR	NR	NR	NR	60% of heart rate reserve
Takao Suzuki	2012	NR	26.8 ± 1.8	NR	NR	NR	NR	12.5 ± 5.9	NR	
Xiu-hong Wei	2014	NR	24.33 ± 1.65	NR	NR	NR	NR	NR	NR	
Montero-Odasso	2023	NR	NR	14.5 ± 6.1	NR	NR	NR	NR	NR	Exercise + Cognitive training + Placebo vitamin D
Jorge Buele	2024	22.36 ± 2.59	NR	NR	NR	NR	NR	NR	3.93 ± 2.81	
Carvalho	2025	20.85 ± 1.87	NR	NR	NR	NR	NR	NR	3.72 ± 2.23	
Miaoran Lin	2024	21.33 ± 2.22	NR	NR	NR	NR	NR	NR	NR	
So Young Moon	2025	NR	26.5 ± 2.0	NR	NR	NR	NR	NR	4.4 ± 3.7	
Chunhui Zhou	2025	22.90 ± 2.63	NR	NR	NR	NR	NR	NR	NR	TCG
Ha Yeong Choi	2025	NR	NR	NR	279.9 ± 51.0	NR	NR	NR	NR	
Cheung	2024	NR	NR	NR	NR	NR	NR	32.33 ± 19.31	NR	
Alessandro Sale	2023	NR	NR	13.4 ± 2.84	NR	NR	NR	NR	NR	
Jingsong Wu	2023	NR	NR	NR	NR	NR	NR	12.78 ± 7.36	NR	

Elmar Graessel	2024	22.0 ± 1.8	NR	NR	NR	NR	NR	NR	NR	
Goumopoulos	2023	21.00 ± 3.33	NR	NR	207.50 ± 40.74	NR	5.50 ± 1.33	10.50 ± 2.07	3.50 ± 2.22	
İsmail Uysal	2022	NR	20.92 ± 0.90	NR	NR	NR	NR	NR	6.83 ± 3.19	AG
			21.25 ± 1.22						6.83 ± 3.04	DG
			21.17 ± 1.11						7.17 ± 4.34	ADG
Qiang Zhang	2023	18.33 ± 4.18	22.44 ± 2.33	NR	NR	NR	NR	NR	12.59 ± 4.43	TCE + RTG
		19.06 ± 5.20	NR						12.17 ± 3.36	WG
Kevser Gursan	2026	17.47±1.55	NR	NR	NR	NR	NR	NR	NR	HEG
		18.00±1.73								CEG
Mosayeb Mozafari	2025	NR	20.94 ± 0.87	NR	NR	NR	NR	NR	NR	
Petri	2025	NR	20.60±2.83	NR	NR	NR	NR	NR	2.20±1.68	
Priya G.	2025	NR	NR	NR	289.73 ± 120.36	NR	NR	NR	NR	
Sunyoung Kang	2026	NR	23.9 ± 2.79	29.2 ± 6.88	NR	NR	NR	NR	NR	
Jin-Hyuck Park	2024	NR	NR	NR	237.73±21.86	NR	2.97±0.85	NR	NR	AG
Hiroshi Hayashi	2026	22.9±2.1	NR	NR	NR	NR	NR	NR	4.44±3.0	
Kang	2024	17.73 ± 6.14	23.07 ± 3.69	NR	NR	NR	NR	NR	12.71 ± 7.00	
Rivas-Campo Y.	2023	21.63 ± 1.53	NR	NR	199.33 ± 29.09	NR	NR	NR	NR	
Eun Hee Lim	2023	20.33 ± 4.70	24.42 ± 1.98	NR	194.77 ± 83.62	NR	NR	NR	NR	
Kitsana Krootnark	2024	20.17 ± 2.09	NR	NR	NR	NR	NR	NR	NR	Aerobic exercise
		19.60 ± 1.83					5.93 ± 1.11			Resistance exercise
Givon Schaham	2024	22.67 ± 2.22	NR	NR	NR	NR	NR	NR	NR	
Meixiang Fan	2024	24.8 ± 3.9	28.4 ± 1.6	NR	NR	NR	6.9 ± 2.3	NR	NR	
Hong Yu	2025	20.36 ± 2.90	NR	NR	171.00 ± 66.26	NR	6.83 ± 1.11	NR	NR	

Supplementary Table 8. Global inconsistency table for MoCA, MMSE, ADAS-Cog, TMT-B, DST, SCWT and DRA outcomes in older adults with mild cognitive impairment.

Outcome	Global inconsistency P value
MoCA	0.94
MMSE	0.318
ADAS-Cog	0.9014
TMT-B	0.3904
DST	0.6972
SCWT	0.1859
DRA	0.8517

Supplementary Table 9. Node-splitting analysis for the MoCA outcome in older adults with mild cognitive impairment

Side	Coef.	Std. Err.	Coef.	Std. Err.	Coef.	Std. Err.	P>z	tau
AC VS AE	1.666775	0.6359732	1.920893	0.9299351	-0.2541185	1.126292	0.821	1.218488
AC VS AE-CT	2.548463	1.150686	2.505845	1.476204	0.0426181	1.87137	0.982	1.219425
AC VS CT	0.5451609	0.826558	1.429312	1.088225	-0.8841507	1.367965	0.518	1.218203
AC VS MBE	2.936088	0.8294913	3.980665	1.562914	-1.044577	1.779301	0.557	1.194839
AC VS ME	0.9833013	0.6667199	-1.588284	1.28319	2.571585	1.446061	0.075	1.109371
AC VS RT	0.19	2.41175	0.6906341	0.9177833	-0.5006341	2.580477	0.846	1.20294
AE VS AE-CT	1.65962	1.64968	0.3138637	1.196384	1.345756	2.069624	0.516	1.207969
AE VS MBE	2.403141	2.123554	1.236698	0.9008417	1.166443	2.317944	0.615	1.194891
AE VS RT	-0.9110566	1.411841	-1.237709	1.062757	0.3266523	1.764201	0.853	1.224053
AE VS WLC	-2.780748	0.6430176	-1.763006	1.014059	-1.017742	1.201918	0.397	1.18619
AE-CT VS WLC	-2.395182	1.612323	-3.808449	1.254421	1.413267	2.064811	0.494	1.210107

CT VS E	-0.4199997	1.462174	0.0028544	1.284382	-0.4228541	1.946174	0.828	1.220344
CT VS RT	-0.8805377	1.345987	0.3072993	1.247786	-1.187837	1.836061	0.518	1.206011
CT VS WLC	-1.685359	1.177521	-1.560333	0.9431499	-0.1250262	1.511681	0.934	1.225352
E VS ME	-1.499995	1.334409	0.8684433	1.288949	-2.368439	1.855277	0.202	1.154735
E VS ME-CT	-1.62	1.582005	-1.51138	167.1842	-0.1086199	167.1907	0.999	1.18701
E VS WLC	-0.1999974	1.50162	-2.2013	1.180668	2.001302	1.910196	0.295	1.175592
MBE VS WLC	-4.450014	1.531132	-3.676926	1.015507	-0.773088	1.837285	0.674	1.204077
ME VS WLC	0.1999962	1.71967	-1.505033	0.8669312	1.70503	1.925834	0.376	1.188917
RT VS WLC	-1.654589	0.8734882	-0.2786943	1.698627	-1.375895	1.905243	0.47	1.201027

Supplementary Table 10. Node-splitting analysis for the MMSE outcome in older adults with mild cognitive impairment.

Side	Coef.	Std. Err.	Coef.	Std. Err.	Coef.	Std. Err.	P>z	tau
AE-CT VS AE	0.5880835	0.682694	-1.600149	1.033429	2.188232	1.239313	0.077	0.9654167
AE-CT VS CT	-0.5545663	0.9778561	1.387074	1.342936	-1.941641	1.661543	0.243	1.032283
AE-CT VS ME	1.70764	0.718138	1.891313	0.9916461	-0.1836726	1.224024	0.881	1.082055
AE-CT VS ME-CT	1	1.10979	2.361001	1.293829	-1.361001	1.70459	0.425	1.052448
AE VS WLC	-1.550742	0.5870206	-3.741533	1.093716	2.190791	1.239791	0.077	0.965458
AE-CT VS WLC	-0.4	1.082761	-4.241303	81.93	3.841303	81.94219	0.963	1.029742
CT VS WLC	-3.145419	1.134063	-1.20383	1.212953	-1.941588	1.661671	0.243	1.032286
D/VRE VS WLC	-2.61	1.055252	-4.241319	53.17245	1.631319	53.18229	0.976	1.029814
ME VS ME-CT	0.4200044	1.141928	-0.9431136	1.265949	1.363118	1.704882	0.424	1.052469
ME VS RT	-1.83	1.13336	-4.556421	3.210336	2.726421	3.407545	0.424	1.052396
ME VS RT-CT	0.17	1.119455	-2.556421	3.205453	2.726421	3.407545	0.424	1.052396
ME VS WLC	-4.397135	0.9122837	-3.252085	1.02107	-1.14505	1.368491	0.403	1.036084
ME-CT VS RT	-2.25	1.13739	0.4720028	3.207829	-2.722003	3.40918	0.425	1.052448

ME-CT VS RT-CT	-0.25	1.123535	2.472003	3.20	-2.722003	3.40918	0.425	1.052448
RT VS RT-CT	2.00002	2.033553	2.707943	14722.58	-0.7079227	14722.58	1	1.999999

Supplementary Table 11. Node-splitting analysis for the ADAS-Cog outcome in older adults with mild cognitive impairment.

Side	Coef.	Std.Err.	Coef.	Std.Err.	Coef.	Std. Err.	P>z	tau
AC VS AE	0.14	1.116811	-3.736597	110.9487	3.876597	110.9524	0.972	1.81e-07
AC VS CT	-0.3345303	0.4373708	-1.070563	1.940787	0.7360322	1.992825	0.712	1.79e-06
AC VS MBE	2.34	0.864523	0.4017069	55.46903	1.938293	55.47577	0.972	8.36e-11
AC VS ME	-0.0154539	0.3902462	0.1469344	2.484067	-0.1623883	2.527646	0.949	7.22e-09
AC VS ME-CT	-0.5673992	0.4815523	-0.1441044	1.838146	-0.4232949	1.884544	0.822	3.32e-10
AE VS MBE	2.2	0.9786864	-1.676586	110.9622	3.876586	110.962	0.972	1.97e-10
C VS WLC	2.1	0.5587773	5.41754	179.6125	-3.31754	179.6132	0.985	4.83e-10
CT VS ME	0.3941831	0.4118771	-0.275995	1.740621	0.6701781	1.783236	0.707	3.81e-08
CT VS ME-CT	-0.1015667	0.4895429	-1.933004	2.406406	1.831437	2.45237	0.455	4.08e-10
D/VRE VS MBE	-1.8	1.018812	2.076586	110.9447	-3.876586	110.9538	0.972	1.40e-09
D/VRE VS WLC	-1.4	0.8020424	-3.740898	144.1582	2.340898	144.1611	0.987	5.69e-10
MBE VS WLC	0.3999449	1.063013	-1.495627	66.57136	1.895572	66.58072	0.977	3.94e-07
ME VS ME-CT	-0.5162635	0.4558728	-0.8604683	1.879177	0.3442049	1.915912	0.857	2.87e-07
RT VS WLC	5.02	1.165377	5.589561	241.7615	-0.5695613	241.767	0.998	2.71e-10

Supplementary Table 12. Node-splitting analysis for the TMT-B outcome in older adults with mild cognitive impairment.

Side	Coef.	Std. Err.	Coef.	Std. Err.	Coef.	Std. Err.	P>z	tau
AC VS AE	-6.992127	7.40682	-20.99255	44.80102	14.00042	45.26201	0.757	6.574814
AC VS CT	-23.03757	8.837682	-12.0021	12.80527	-11.03548	15.33943	0.472	6.637166

AC VS MBE	-13.50384	9.053253	-17.87019	12.95439	4.366352	16.92541	0.796	8.067513
AC VS MBE-CT	-23.58	8.61758	-47.63415	23.80651	24.05415	25.20111	0.34	7.607357
AC VS ME	-16.54783	8.544213	-1.967169	21.95397	-14.58066	25.20377	0.563	8.183704
AE VS ME	7.029688	44.30837	-6.972278	9.249381	14.00197	45.26348	0.757	6.574797
CT VS MBE	6.389864	13.56441	1.399316	15.13996	4.990548	20.32761	0.806	9.425058
CT VS WLC	-15.73163	26.14354	47.90547	21.1231	-63.63711	33.6107	0.058	4.157378
D/VRE VS MBE	18.9	18.03599	146.1158	61.93164	-127.2158	67.21332	0.058	4.15803
D/VRE VS WLC	56.2	14.39096	-71.06877	64.73895	127.2688	67.22092	0.058	4.157506
MBE VS MBE-CT	-14.86	8.849102	9.19415	23.5503	-24.05415	25.2011	0.34	7.607349
MBE VS ME	0.3732934	10.88768	2.345014	14.00497	-1.971721	17.73531	0.911	9.530133
MBE VS WLC	37.30097	19.6342	-26.32798	27.27903	63.62894	33.61049	0.058	4.157616

Supplementary Table 13. Node-splitting analysis for the DST outcome in older adults with mild cognitive impairment.

Side	Coef.	Std. Err.	Coef.	Std. Err.	Coef.	Std. Err.	P>z	tau
AC VS AE-CT	0.4967936	0.436267	0.4892567	0.4679162	0.0075368	0.6398283	0.991	0.2039783
AC VS CT	0.5192807	0.2165406	0.5267441	0.6018633	-0.0074634	0.639715	0.991	0.2039785
AC VS MBE	0.9807897	0.2956521	0.019923	1.150691	0.9608667	1.196143	0.422	0.1513444
AC VS MBE-CT	0.8339434	0.2965973	1.036243	1.195904	-0.2023001	1.246546	0.871	0.1732196
AC VS ME	1.6	0.3607715	0.1048233	1.046267	1.495177	1.144478	0.191	0.0773913
AE-CT VS CT	0.0300021	0.4148805	0.0224983	0.4871532	0.0075038	0.639882	0.991	0.2039981
AE-CT VS WLC	-0.7900004	0.3814032	-0.8049545	1.217865	0.0149541	1.278616	0.991	0.2038717
CT VS WLC	-0.8200021	0.4110589	-0.8050328	1.188059	-0.0149693	1.278095	0.991	0.203874
MBE VS MBE-CT	0.1639186	0.540929	-0.2535072	0.4805995	0.4174258	0.723626	0.564	0.1573736
MBE VS ME	0.2	0.4096614	1.695177	0.9907939	-1.495177	1.144477	0.191	0.0773895

RT VS WLC	-0.4426493	0.2627513	-0.5050001	44.66161	0.0623508	44.66272	0.999	0.1330744

Supplementary Table 14. Node-splitting analysis for the SCWT outcome in older adults with mild cognitive impairment.

Side	Coef.	Std. Err.	Coef.	Std. Err.	Coef.	Std. Err.	P>z	tau
AE VS AE-CT	-1.89	2.077472	22.91495	15.02672	-24.80495	15.12955	0.101	1.11e-06
AE VS CT	-5.557197	2.273067	8.752092	8.229411	-14.30929	8.590501	0.096	7.94e-06
AE VS WLC	-0.6146007	6.190757	-13.30599	23.25288	12.69138	24.00289	0.597	6.330882
AE-CT VS C	-3.55	6.937681	9.55816	23.86506	-13.10816	24.88818	0.598	6.506432
AE-CT VS WLC	-2.63	7.25135	6.01384	18.37164	-8.64384	19.78048	0.662	6.861092
CT VS MBE	-1.400002	7.184383	5.156497	10.17082	-6.5565	12.45234	0.599	6.50979
CT VS WLC	0.9097225	8.250442	1.61332	11.07415	-0.703598	13.80417	0.959	7.854162
D/VRE VS E	-0.7	7.575719	-13.80921	23.60256	13.10921	24.90019	0.599	6.508954
D/VRE VS WLC	-2.9	7.362694	10.19839	23.78125	-13.09839	24.88044	0.599	6.505255
MBE VS WLC	-2.199971	7.552509	4.354557	9.899576	-6.554528	12.45155	0.599	6.509663
RT VS WLC	-0.45	2.867508	-7.083021	323.9134	6.633021	323.9269	0.984	1.876137

Supplementary Table 15. Node-splitting analysis for the DRA outcome in older adults with mild cognitive impairment.

Side	Coef.	Std. Err.	Coef.	Std. Err.	Coef.	Std. Err.	P>z	tau
AC VS AE	-0.2772592	0.1168531	-0.4832481	0.3244632	0.205989	0.3456197	0.551	0.0360978
AC VS CT	-0.0995521	0.2463943	-0.0462478	0.2522636	-0.0533043	0.3526288	0.88	0.0001765
AC VS MBE	-0.0990544	0.2581807	0.0279326	0.2848249	-0.126987	0.389546	0.744	0.0120218
AC VS ME-CT	-0.1984828	0.1335685	0.0032004	0.3474636	-0.2016833	0.3722518	0.588	0.0440355
AE VS MBE	0.1061432	0.3853202	0.3190325	0.2403467	-0.2128893	0.455088	0.64	3.67e-07
AE VS ME	-0.1798859	0.362315	-0.0694062	0.2544444	-0.1104797	0.4427343	0.803	0.0200988

AE VS WLC	0.554284	0.1260127	0.1993349	0.306029	0.3549491	0.3338868	0.288	0.0396363
CT VS D/VRE	-0.1477201	0.2724929	0.0957921	0.3446835	-0.2435122	0.4393847	0.579	0.0330298
CT VS MBE	0.0749654	0.224735	-0.0523476	0.3145698	0.1273131	0.3866005	0.742	0.0009435
D/VRE VS ME-CT	0.3180655	0.3964103	-0.2255925	0.2770573	0.543658	0.4836344	0.261	1.38e-06
D/VRE VS WLC	-0.0935622	0.3438097	0.6535187	0.2984159	-0.747081	0.4552553	0.101	2.61e-07
ME VS ME-CT	0.0931619	0.409364	0.2984856	0.2854508	-0.2053237	0.4990601	0.681	0.0284088
ME VS RT	1.597867	0.4454395	2.008458	0.9232785	-0.4105919	0.9980942	0.681	0.0283946

Supplementary Table 16. Loop-specific inconsistency analysis for the MoCA outcome in older adults with mild cognitive impairment.

Loop	IF	seIF	z_value	p_value	CI_95	Loop_Heterog_tau2
AE-AE-CT-WLC	2.74	3.927	0.698	0.485	(0.00,10.44)	5.713
AC-ME-RT-WLC	2.608	2.906	0.897	0.37	(0.00,8.30)	0.695
AC-MBE-ME-WLC	2.595	3.142	0.826	0.409	(0.00,8.75)	1.631
CT-RT-WLC	2.55	1.05	2.43	0.015	(0.00,4.61)	0
AC-AE-ME-WLC	2.274	2.294	0.991	0.322	(0.00,6.77)	1.987
AC-CT-D/VRE-ME	2.125	1.148	1.852	0.064	(0.00,4.37)	0
AC-CT-ME-WLC	1.763	1.433	1.23	0.219	(0.00,4.57)	0
AC-AE-CT-RT-WLC	1.75	3.368	0.52	0.603	(0.00,8.35)	1.741
AC-AE-CT-MBE-WLC	1.442	3.83	0.376	0.707	(0.00,8.95)	4.002
D/VRE-ME-WLC	1.1	1.692	0.65	0.516	(0.00,4.42)	0
AC-AE-CT-ME-WLC	1.045	1.754	0.596	0.551	(0.00,4.48)	0
AC-AE-MBE	1.025	2.745	0.373	0.709	(0.00,6.41)	1.783
AC-AE-AE-CT	1.016	1.48	0.687	0.492	(0.00,3.92)	0.235
CT-D/VRE-WLC	0.737	1.345	0.548	0.584	(0.00,3.37)	0
AC-AE-CT-CT-WLC	0.718	1.41	0.509	0.611	(0.00,3.48)	0

AE-MBE-WLC	0.666	4.031	0.165	0.869	(0.00,8.57)	5.469
AC-AE-RT	0.577	2.357	0.245	0.806	(0.00,5.20)	0.272
AC-MBE-RT-WLC	0.378	4.103	0.092	0.927	(0.00,8.42)	4.098
AC-CT-RT	0.293	2.218	0.132	0.895	(0.00,4.64)	0
AE-RT-WLC	0.199	2.969	0.067	0.947	(0.00,6.02)	4.905
AC-CT-MBE-WLC	0.191	2.522	0.076	0.939	(0.00,5.13)	1.912

Supplementary Table 17. Loop-specific inconsistency analysis for the MMSE outcome in older adults with mild cognitive impairment.

Loop	IF	seIF	z_value	p_value	CI_95	Loop_Heterog_tau2
AC-AE-CT-RT-CT	2.661	1.547	1.721	0.085	(0.00,5.69)	0.629
AC-AE-ME-RT-CT	1.748	1.577	1.109	0.268	(0.00,4.84)	1.265
AC-ME-ME-CT	1.15	2.618	0.439	0.661	(0.00,6.28)	2.737
AC-CT-ME-RT-CT	1.047	2.109	0.496	0.62	(0.00,5.18)	1.524

Supplementary Table 18. Loop-specific inconsistency analysis for the ADAS-Cog outcome in older adults with mild cognitive impairment.

Loop	IF	seIF	z_value	p_value	CI_95	Loop_Heterog_tau2
AC-ME-ME-CT	1.075	2.028	0.53	0.596	(0.00,5.05)	0
AC-CT-ME-CT	0.867	1.676	0.518	0.605	(0.00,4.15)	0
AC-CT-ME	0.175	1.202	0.146	0.884	(0.00,2.53)	0

Supplementary Table 19. Loop-specific inconsistency analysis for the TMT-B outcome in older adults with mild cognitive impairment.

Loop	IF	seIF	z_value	p_value	CI_95	Loop_Heterog_tau2
CT-MBE-WLC	59.422	33.737	1.761	0.078	(0.00,125.55)	0
AC-MBE-MBE-CT	27.88	15.383	1.812	0.07	(0.00,58.03)	0

AC-AE-ME	16.027	45.971	0.349	0.727	(0.00,106.13)	71.161
AC-CT-MBE	5.949	25.716	0.231	0.817	(0.00,56.35)	237.168
AC-MBE-ME	1.65	8.608	0.192	0.848	(0.00,18.52)	0

Supplementary Table 20. Loop-specific inconsistency analysis for the DST outcome in older adults with mild cognitive impairment.

Loop	IF	seIF	z_value	p_value	CI_95	Loop_Heterog_tau2
AC-MBE-ME	0.674	0.646	1.043	0.297	(0.00,1.94)	0
AC-MBE-MBE-CT	0.303	0.67	0.451	0.652	(0.00,1.62)	0.004
AC-AE-CT-CT	0.001	0.567	0.002	0.999	(0.00,1.11)	0

Supplementary Table 21. Loop-specific inconsistency analysis for the SCWT outcome in older adults with mild cognitive impairment.

Loop	IF	seIF	z_value	p_value	CI_95	Loop_Heterog_tau2
AE-AE-CT-WLC	11.97	7.912	1.513	0.13	(0.00,27.48)	0
AE-CT-WLC	11.97	8.022	1.492	0.136	(0.00,27.69)	0
CT-MBE-WLC	4.52	5.503	0.821	0.411	(0.00,15.31)	0

Supplementary Table 22. Loop-specific inconsistency analysis for the DRA outcome in older adults with mild cognitive impairment.

Loop	IF	seIF	z_value	p_value	CI_95	Loop_Heterog_tau2
ME-ME-CT-RT-CT	0.855	0.723	1.183	0.237	(0.00,2.27)	0
CT-D/VRE-ME-CT	0.269	0.553	0.487	0.626	(0.00,1.35)	0
AE-ME-RT-CT	0.244	0.679	0.36	0.719	(0.00,1.57)	0.084
AC-AE-ME-ME-CT	0.169	0.677	0.249	0.803	(0.00,1.50)	0
AC-CT-MBE	0.073	0.422	0.173	0.863	(0.00,0.90)	0
AC-AE-MBE	0.053	0.593	0.089	0.929	(0.00,1.21)	0

Supplementary Table 23. Heterogeneity estimates (τ^2) for outcomes with estimable between-study variance.

Outcome	Heterogeneity (τ^2)
MoCA	1.4089562
MMSE	1.0651807
TMT-B	41.9822952
DST	0.020431
DRA	3.655744×10^{-18}
MFA	0.061795

Supplementary Table 24. Within-node heterogeneity tests for MBE and ME stratified by outcome.

Outcome	Comparison	df	Q	P value for Q	t^2	I^2	H^2
MoCA	MBE VS AC	3	13.07	0.004	5.371	84.51%	6.46
	ME VS AC	2	0.29	0.867	0	0	1
MMSE	MBE VS AC	1	0.71	0.4	0	0	1
	ME VS AC	2	45.37	<0.001	2.745	93.23%	14.76
ADAS-Cog	ME VS AC	1	0.01	0.921	0	0	1
TMT-B	MBE VS AC	1	2.99	0.084	258.808	66.59%	2.99
	ME VS AC	2	3	0.223	79.177	25.25%	1.34
DST	MBE VS AC	3	6.07	0.108	0.078	45.03%	1.82
DRA	MBE VS AC	1	0.81	0.367	0	0	1

Supplementary Table 25. SUCRA ranking table for the MoCA outcome in older adults with mild cognitive impairment.

Treatm~t	SUCRA	PrBest	MeanRank
MBE	96.3	69.3	1.4
AE-CT	87.9	26.9	2.2
AE	77	1.2	3.3
CT	57.8	0.1	5.2
D/VRE	52.3	0.5	5.8
RT	49.5	0.4	6.1
ME	43.8	0	6.6
AC	31.4	0	7.9
ME-CT	22.4	1.3	8.8
MBE-CT	17.6	0.2	9.2
WLC	14	0	9.6

Supplementary Table 26. SUCRA ranking table for the MMSE outcome in older adults with mild cognitive impairment.

Treatm~t	SUCRA	PrBest	MeanRank
MBE	90.7	53.9	2
ME	82.7	10.4	2.9
ME-CT	77.7	10.7	3.5
RT-CT	77.6	18.5	3.5
D/VRE	53.1	4.2	6.2
MBE-CT	45.9	1.8	7
CT	43.8	0.2	7.2
AC	40.1	0	7.6

AE	38.6	0	7.8
RT	33.1	0.2	8.4
AE-CT	12.5	0	10.6
WLC	4.3	0	11.5

Supplementary Table 27. SUCRA ranking table for the ADAS-Cog outcome in older adults with mild cognitive impairment.

Treatm~t	SUCRA	PrBest	MeanRank
RT	92.7	77.9	1.7
ME-CT	78.3	11.1	3
CT	72.7	5.4	3.5
AE	57.5	4.4	4.8
ME	56.8	0.4	4.9
AC	56.4	0.3	4.9
AE-CT	50	0.4	5.5
MBE	18.9	0	8.3
WLC	15.7	0	8.6
D/VRE	1	0	9.9

Supplementary Table 28. SUCRA ranking table for the TMT-B outcome in older adults with mild cognitive impairment.

Treatm~t	SUCRA	PrBest	MeanRank
D/VRE	96.7	85.3	1.3
MBE-CT	84.1	10.3	2.4
CT	72.6	3.4	3.5
MBE	59.7	0.1	4.6

ME	58.6	0.4	4.7
AE	42.3	0.2	6.2
WLC	29	0	7.4
AC	24.8	0	7.8
AE-CT	17.6	0.2	8.4
ME-CT	14.8	0.1	8.7

Supplementary Table 29. SUCRA ranking table for the DST outcome in older adults with mild cognitive impairment.

Treatm~t	SUCRA	PrBest	MeanRank
ME	97.1	83.3	1.2
MBE	78.9	6.9	2.7
MBE-CT	75.1	7.4	3
CT	56.9	0.1	4.5
AE-CT	55.8	1.2	4.5
RT	36.6	0.9	6.1
AC	24	0	7.1
AE	16	0.1	7.7
WLC	9.7	0	8.2

Supplementary Table 30. SUCRA ranking table for the SCWT outcome in older adults with mild cognitive impairment.

Treatm~t	SUCRA	PrBest	MeanRank
AE	87.2	51	1.8
AE-CT	67.9	14.8	2.9
D/VRE	59.8	24.5	3.4
RT	42.3	6.5	4.5

WLC	33	0.1	5
MBE	32.2	2.7	5.1
CT	27.5	0.3	5.3

Supplementary Table 31. SUCRA ranking table for the DRA outcome in older adults with mild cognitive impairment.

Treatm~t	SUCRA	PrBest	MeanRank
ME	82.7	34.5	2.7
AE	79.8	11.7	3
AE-CT	73.6	29.4	3.6
ME-CT	62.7	3	4.7
D/VRE	55.9	5.9	5.4
CT	48.7	1.9	6.1
RT-CT	48.5	11.5	6.2
MBE	44.6	2.1	6.5
AC	36.1	0	7.4
WLC	17.3	0	9.3
RT	0.1	0	11

Supplementary Table 32. SUCRA ranking table for the MFA outcome in older adults with mild cognitive impairment.

Treatm~t	SUCRA	PrBest	MeanRank
ME-CT	85.5	57.2	2.2
AE	76.2	10.9	2.9
AC	56.8	0.4	4.5
CT	51.3	4.2	4.9

MBE-CT	45.7	9	5.3
ME	43.8	7.8	5.5
MBE	37	5.6	6
WLC	30.7	1.1	6.5
RT	23	3.7	7.2

Supplementary Table 33. Meta-regression analysis of the MoCA outcome with mean age as a moderator in older adults with mild cognitive impairment.

Intervention	Covariate	Coefficient	Standard Error	Z-statistic	P>z	lower confidence interval	upper confidence interval
WLC VS AC	Mean age	-0.1126024	0.1184208	-0.95	0.342	-0.3447029	0.1194981
WLC VS AE	Mean age	-0.1750008	0.102493	-1.71	0.088	-0.3758835	0.0258819
WLC VS AE-CT	Mean age	-0.0664263	0.178272	-0.37	0.709	-0.415833	0.2829804
WLC VS CT	Mean age	-0.0028563	0.3872757	-0.01	0.994	-0.7619027	0.7561901
WLC VS D/VRE	Mean age	-0.509287	0.356456	-1.43	0.153	-1.207928	0.1893539
WLC VS MBE	Mean age	-0.2717819	0.2066825	-1.31	0.189	-0.6768722	0.1333084
WLC VS ME	Mean age	-0.05036	0.202127	-0.25	0.803	-0.4465215	0.3458016
WLC VS RT	Mean age	-0.283934	0.1912848	-1.48	0.138	-0.6588453	0.0909773

Supplementary Table 34. Meta-regression analysis of the MMSE outcome with mean age as a moderator in older adults with mild cognitive impairment.

Intervention	Covariate	Coefficient	Standard Error	Z-statistic	P>z	lower confidence interval	upper confidence interval
WLC VS AC	Mean age	0.1561934	0.1114021	1.4	0.161	-0.0621506	0.3745375
WLC VS AE	Mean age	0.0540539	0.0819578	0.66	0.51	-0.1065804	0.2146882
WLC VS CT	Mean age	0.3606037	0.2190438	1.65	0.1	-0.0687143	0.7899217

WLC VS MBE	Mean age	0.3098452	0.2620227	1.18	0.237	-0.2037098	0.8234002
WLC VS ME	Mean age	-0.3786419	0.2328966	-1.63	0.104	-0.8351108	0.077827
WLC VS ME-CT	Mean age	0.8818578	1.017398	0.87	0.386	-1.112206	2.875921

Supplementary Table 35. Meta-regression analysis of the TMT-B outcome with mean age as a moderator in older adults with mild cognitive impairment.

Intervention	Covariate	Coefficient	Standard Error	Z-statistic	P>z	lower confidence interval	upper confidenc e interval
WLC VS AC	Mean age	-13.44749	8.736203	-1.54	0.124	-30.57014	3.675149
WLC VS AE	Mean age	-13.34957	9.093181	-1.47	0.142	-31.17188	4.47274
WLC VS AE-CT	Mean age	-15.28234	8.984406	-1.7	0.089	-32.89145	2.326775
WLC VS CT	Mean age	-4.27869	8.763087	-0.49	0.625	-21.45402	12.89664
WLC VS MBE	Mean age	-9.138189	8.531296	-1.07	0.284	-25.85922	7.582844
WLC VS ME	Mean age	-11.84251	8.848438	-1.34	0.181	-29.18512	5.500114

Supplementary Table 36. Meta-regression analysis of the DST outcome with mean age as a moderator in older adults with mild cognitive impairment.

Intervention	Covariate	Coefficient	Standard Error	Z-statistic	P>z	lower confidence interval	upper confidenc e interval
WLC VS AC	Mean age	0.0760464	1.917875	0.04	0.968	-3.682919	3.835012
WLC VS AE	Mean age	0.1158892	1.919911	0.06	0.952	-3.647068	3.878846
WLC VS AE-CT	Mean age	0.0993476	1.917718	0.05	0.959	-3.659311	3.858007
WLC VS CT	Mean age	0.0881429	1.917839	0.05	0.963	-3.670752	3.847038
WLC VS MBE	Mean age	0.1667651	1.921893	0.09	0.931	-3.600076	3.933606
WLC VS MBE-CT	Mean age	0.1606971	1.920158	0.08	0.933	-3.602743	3.924137
WLC VS RT	Mean age	-0.1049564	0.1056939	-0.99	0.321	-0.3121126	0.1021998

Supplementary Table 37. Meta-regression analysis of the DRA outcome with mean age as a moderator in older adults with mild cognitive impairment.

Intervention	Covariate	Coefficient	Standard Error	Z-statistic	P>z	lower confidence interval	upper confidence interval
WLC VS AC	Mean age	0.0249268	0.1495443	0.17	0.868	-0.2681746	0.3180282
WLC VS AE	Mean age	-0.0724206	0.0923203	-0.78	0.433	-0.2533651	0.1085238
WLC VS CT	Mean age	0.0065841	0.2103561	0.03	0.975	-0.4057063	0.4188746
WLC VS D/VRE	Mean age	0.6635464	1.255434	0.53	0.597	-1.797058	3.124151
WLC VS MBE	Mean age	0.1469169	0.7275776	0.2	0.84	-1.279109	1.572943
WLC VS ME	Mean age	-0.0870538	0.7132723	-0.12	0.903	-1.485042	1.310934
WLC VS ME-CT	Mean age	0.6125859	0.6135325	1	0.318	-0.5899156	1.815087

Supplementary Table 38. Meta-regression analysis of the MFA outcome with mean age as a moderator in older adults with mild cognitive impairment.

Intervention	Covariate	Coefficient	Standard Error	Z-statistic	P>z	lower confidence interval	upper confidence interval
WLC VS AC	Mean age	-0.801869	4.990383	-0.16	0.872	-10.58284	8.979102
WLC VS AE	Mean age	-0.6959069	4.988968	-0.14	0.889	-10.4741	9.08229
WLC VS CT	Mean age	-0.9681777	5.004316	-0.19	0.847	-10.77646	8.840101

Supplementary Table 39. Meta-regression analysis of the MoCA outcome with intervention frequency as a moderator in older adults with mild cognitive impairment.

Intervention	Covariate	Coefficient	Standard Error	Z-statistic	P>z	lower confidence interval	upper confidence interval
WLC VS AC	Intervention frequency	-0.1493092	1.718737	-0.09	0.931	-3.517972	3.219354
WLC VS AE	Intervention frequency	0.0395412	0.6831461	0.06	0.954	-1.299401	1.378483
WLC VS AE-CT	Intervention frequency	0.1823996	1.821879	0.1	0.92	-3.388418	3.753217
WLC VS CT	Intervention frequency	0.0889828	1.674006	0.05	0.958	-3.192009	3.369974
WLC VS D/VRE	Intervention frequency	1.341095	1.251071	1.07	0.284	-1.110959	3.79315
WLC VS MBE	Intervention frequency	-0.0823047	1.868802	-0.04	0.965	-3.74509	3.58048
WLC VS ME	Intervention frequency	0.1930384	1.98457	0.1	0.923	-3.696648	4.082725
WLC VS RT	Intervention frequency	0.6027334	0.7536329	0.8	0.424	-0.87436	2.079827

Supplementary Table 40. Meta-regression analysis of the MMSE outcome with intervention frequency as a moderator in older adults with mild cognitive impairment.

Intervention	Covariate	Coefficient	Standard Error	Z-statistic	P>z	lower confidence interval	upper confidence interval
WLC VS AC	Intervention frequency	-0.587554	0.6730689	-0.87	0.383	-1.906745	0.7316367
WLC VS AE	Intervention frequency	-0.4800883	0.6125948	-0.78	0.433	-1.680752	0.7205756

WLC VS CT	Intervention frequency	-0.475435	1.910144	-0.25	0.803	-4.219249	3.268379
WLC VS MBE	Intervention frequency	0.4417496	2.101403	0.21	0.833	-3.676925	4.560424
WLC VS ME	Intervention frequency	0.1381657	0.7343184	0.19	0.851	-1.301072	1.577403

Supplementary Table 41. Meta-regression analysis of the TMT-B outcome with intervention frequency as a moderator in older adults with mild cognitive impairment.

Intervention	Covariate	Coefficient	Standard Error	Z-statistic	P>z	lower confidence interval	upper confidence interval
WLC VS AC	Intervention frequency	-20.19652	29.72405	-0.68	0.497	-78.45459	38.06154
WLC VS AE	Intervention frequency	-28.69398	31.02547	-0.92	0.355	-89.50278	32.11482
WLC VS AE-CT	Intervention frequency	-36.26487	35.56836	-1.02	0.308	-105.9776	33.44784
WLC VS CT	Intervention frequency	-16.66272	24.96981	-0.67	0.505	-65.60264	32.27721
WLC VS MBE	Intervention frequency	-26.87603	22.90644	-1.17	0.241	-71.77184	18.01978
WLC VS ME	Intervention frequency	-33.39053	54.41211	-0.61	0.539	-140.0363	73.25525

Supplementary Table 42. Meta-regression analysis of the DST outcome with intervention frequency as a moderator in older adults with mild cognitive impairment.

Intervention	Covariate	Coefficient	Standard Error	Z-statistic	P>z	lower confidence interval	upper confidenc e interval
WLC VS AC	Intervention frequency	-0.5810503	15.29737	-0.04	0.97	-30.56335	29.40125
WLC VS AE-CT	Intervention frequency	-0.4987646	15.29718	-0.03	0.974	-30.48068	29.48315
WLC VS MBE	Intervention frequency	-0.707063	15.31015	-0.05	0.963	-30.7144	29.30027
WLC VS MBE-CT	Intervention frequency	-1.110996	15.30984	-0.07	0.942	-31.11772	28.89573
WLC VS RT	Intervention frequency	-0.3917146	15.17091	2.29	0.922	0.0567513	29.72667

Supplementary Table 43. Meta-regression analysis of the DRA outcome with intervention frequency as a moderator in older adults with mild cognitive impairment.

Intervention	Covariate	Coefficient	Standard Error	Z-statistic	P>z	lower confidence interval	upper confidenc e interval
WLC VS AC	Intervention frequency	-0.1973909	21.19033	-0.01	0.993	-41.72967	41.33489
WLC VS AE	Intervention frequency	-0.343989	0.9849248	-0.35	0.727	-2.274406	1.586428
WLC VS CT	Intervention frequency	-2.722549	21.81443	-0.12	0.901	-45.47805	40.03296
WLC VS D/VRE	Intervention frequency	-1.321178	21.22486	-0.06	0.95	-42.92114	40.27878
WLC VS MBE	Intervention frequency	-1.902992	21.29842	-0.09	0.929	-43.64712	39.84114

WLC VS ME	Intervention frequency	-0.5464312	2.151749	-0.25	0.8	-4.763781	3.670919
WLC VS ME-CT	Intervention frequency	-1.161514	21.24482	-0.05	0.956	-42.8006	40.47757

Supplementary Table 44. Meta-regression analysis of the MFA outcome with intervention frequency as a moderator in older adults with mild cognitive impairment.

Intervention	Covariate	Coefficient	Standard Error	Z-statistic	P>z	lower confidence interval	upper confidenc e interval
WLC VS AC	Intervention frequency	3.090579	19.09181	0.16	0.871	-34.32869	40.50985
WLC VS AE	Intervention frequency	2.276431	19.08412	0.12	0.905	-35.12775	39.68061
WLC VS CT	Intervention frequency	4.129719	19.22761	0.21	0.83	-33.5557	41.81514

Supplementary Table 45. Meta-regression analysis of the MoCA outcome with intervention duration as a moderator in older adults with mild cognitive impairment.

Intervention	Covariate	Coefficient	Standard Error	Z-statistic	P>z	lower confidence interval	upper confidenc e interval
WLC VS AC	Intervention duration	0.006267	0.1375349	0.05	0.964	-0.2632965	0.2758304
WLC VS AE	Intervention duration	0.126545	0.0499118	2.54	0.011	0.0287197	0.2243702
WLC VS AE-CT	Intervention duration	0.0583414	0.1583356	0.37	0.713	-0.2519908	0.3686735
WLC VS CT	Intervention duration	-0.0165383	0.148481	-0.11	0.911	-0.3075558	0.2744791

WLC VS D/VRE	Intervention duration	-0.0085462	0.6825433	-0.01	0.99	-1.346307	1.329214
WLC VS MBE	Intervention duration	0.2686798	0.2322906	1.16	0.247	-0.1866015	0.723961
WLC VS ME	Intervention duration	0.0977947	0.1645101	0.59	0.552	-0.2246391	0.4202285
WLC VS RT	Intervention duration	0.3429574	0.3735291	0.92	0.359	-0.3891462	1.075061

Supplementary Table 46. Meta-regression analysis of the MMSE outcome with intervention duration as a moderator in older adults with mild cognitive impairment.

Intervention	Covariate	Coefficient	Standard Error	Z-statistic	P>z	lower confidence interval	upper confidence interval
WLC VS AC	Intervention duration	0.0664217	0.0451891	1.47	0.142	-0.0221473	0.1549908
WLC VS AE	Intervention duration	0.0332134	0.0356221	0.93	0.351	-0.0366046	0.1030314
WLC VS CT	Intervention duration	-0.3927502	0.2059864	-1.91	0.057	-0.7964761	0.0109756
WLC VS MBE	Intervention duration	-0.1909915	0.3801353	-0.5	0.615	-0.936043	0.5540601
WLC VS ME	Intervention duration	-0.0474669	0.0519593	-0.91	0.361	-0.1493053	0.0543715
WLC VS ME-CT	Intervention duration	-0.2676857	0.1188233	-2.25	0.074	-0.5005752	0.0347962

Supplementary Table 47. Meta-regression analysis of the TMT-B outcome with intervention duration as a moderator in older adults with mild cognitive impairment.

Intervention	Covariate	Coefficient	Standard Error	Z-statistic	P>z	lower confidence interval	upper confidence interval
WLC VS AC	Intervention duration	8.725044	3.545316	2.46	0.14	-15.67374	1.776352
WLC VS AE	Intervention duration	8.120389	3.563978	2.28	0.273	-15.10566	1.13512
WLC VS AE-CT	Intervention duration	11.40279	4.613335	2.47	0.413	-20.44476	2.360825
WLC VS CT	Intervention duration	5.537453	3.483712	1.59	0.112	-12.3654	1.290497
WLC VS MBE	Intervention duration	11.94443	4.322749	2.76	0.516	-20.41686	3.471999
WLC VS ME	Intervention duration	10.61008	3.965544	2.68	0.437	-18.3824	2.837759

Supplementary Table 48. Meta-regression analysis of the DST outcome with intervention duration as a moderator in older adults with mild cognitive impairment.

Intervention	Covariate	Coefficient	Standard Error	Z-statistic	P>z	lower confidence interval	upper confidence interval
WLC VS AC	Intervention duration	-0.0281472	1.65003	-0.02	0.986	-3.262146	3.205852
WLC VS AE	Intervention duration	-0.0582428	1.663584	-0.04	0.972	-3.318807	3.202322
WLC VS AE-CT	Intervention duration	0.003585	1.651553	0	0.998	-3.233399	3.240569
WLC VS CT	Intervention duration	-0.0364715	1.651342	-0.02	0.982	-3.273042	3.200099

WLC VS MBE	Intervention duration	-0.0135544	1.651816	-0.01	0.993	-3.251054	3.223946

Supplementary Table 49. Meta-regression analysis of the DRA outcome with intervention duration as a moderator in older adults with mild cognitive impairment.

Intervention	Covariate	Coefficient	Standard Error	Z-statistic	P>z	lower confidence interval	upper confidence interval
WLC VS AC	Intervention duration	0.1444318	0.1227084	1.18	0.239	-0.0960723	0.3849359
WLC VS AE	Intervention duration	0.0265216	0.0430188	0.62	0.538	-0.0577937	0.110837
WLC VS CT	Intervention duration	0.165312	0.2778446	0.59	0.552	-0.3792533	0.7098774
WLC VS D/VRE	Intervention duration	1.200157	0.9035478	1.33	0.184	-0.5707643	2.971078
WLC VS ME	Intervention duration	0.0391725	0.1407358	0.28	0.781	-0.2366645	0.3150096
WLC VS ME-CT	Intervention duration	0.0400826	0.1389578	0.29	0.773	-0.2322698	0.3124349

Supplementary Table 50. Meta-regression analysis of the MFA outcome with intervention duration as a moderator in older adults with mild cognitive impairment.

Intervention	Covariate	Coefficient	Standard Error	Z-statistic	P>z	lower confidence interval	upper confidence interval
WLC VS AC	Intervention duration	0.1464036	1.012813	0.14	0.885	-1.838674	2.131481
WLC VS AE	Intervention duration	0.1350039	1.012736	0.13	0.894	-1.849922	2.11993

WLC VS CT	Intervention duration	-0.1135859	1.171943	-0.1	0.923	-2.410553	2.183381

Supplementary Table 51. Meta-regression analysis of the MoCA outcome with completion rate as a moderator in older adults with mild cognitive impairment.

Intervention	Covariate	Coefficient	Standard Error	Z-statistic	P>z	lower confidence interval	upper confidence interval
WLC VS AC	Completion rate	0.1333768	0.0878666	1.52	0.129	-0.0388384	0.3055921
WLC VS AE	Completion rate	0.0471578	0.0784936	0.6	0.548	-0.1066868	0.2010024
WLC VS AE-CT	Completion rate	0.1035566	0.1553108	0.67	0.505	-0.2008469	0.4079601
WLC VS CT	Completion rate	0.0834858	0.1499503	0.56	0.578	-0.2104114	0.377383
WLC VS D/VRE	Completion rate	0.0187479	0.1485835	0.13	0.9	-0.2724704	0.3099663
WLC VS MBE	Completion rate	-0.0894699	0.2688941	-0.33	0.739	-0.6164926	0.4375528
WLC VS ME	Completion rate	-0.3231603	0.2209362	-1.46	0.144	-0.7561873	0.1098667
WLC VS RT	Completion rate	0.127729	0.0921639	1.39	0.166	-0.0529088	0.3083669

Supplementary Table 52. Meta-regression analysis of the MMSE outcome with completion rate as a moderator in older adults with mild cognitive impairment.

Intervention	Covariate	Coefficient	Standard Error	Z-statistic	P>z	lower confidence interval	upper confidence interval
WLC VS AC	Completion rate	-0.1122346	0.0607354	-1.85	0.065	-0.2312737	0.0068046
WLC VS AE	Completion rate	-0.0973352	0.0532885	-1.83	0.068	-0.2017787	0.0071084
WLC VS CT	Completion rate	0.196483	0.1148431	1.71	0.087	-0.0286054	0.4215714
WLC VS MBE	Completion rate	-0.0026919	0.1525797	-0.02	0.986	-0.3017427	0.2963589
WLC VS ME	Completion rate	0.0567488	0.04752	1.19	0.232	-0.0363887	0.1498864
WLC VS ME-CT	Completion rate	0.1188996	0.0664126	1.79	0.073	-0.0112667	0.2490658

Supplementary Table 53. Meta-regression analysis of the TMT-B outcome with completion rate as a moderator in older adults with mild cognitive impairment.

Intervention	Covariate	Coefficient	Standard Error	Z-statistic	P>z	lower confidence interval	upper confidence interval
WLC VS AC	Completion rate	9.12588	4.712427	1.94	0.053	-0.1103069	18.36207
WLC VS AE	Completion rate	7.239103	4.915912	1.47	0.141	-2.395907	16.87411
WLC VS AE-CT	Completion rate	10.75674	5.060074	2.13	0.231	0.8391772	20.6743
WLC VS CT	Completion rate	7.322472	5.261083	1.39	0.164	-2.989061	17.63401

WLC VS MBE	Completion rate	7.198671	5.957489	1.21	0.227	-4.477793	18.87514
WLC VS ME	Completion rate	11.51223	6.211247	1.85	0.064	-0.6615929	23.68605

Supplementary Table 54. Meta-regression analysis of the DST outcome with completion rate as a moderator in older adults with mild cognitive impairment.

Intervention	Covariate	Coefficient	Standard Error	Z-statistic	P>z	lower confidence interval	upper confidence interval
WLC VS AC	Completion rate	0.0223983	0.70198	0.03	0.975	-1.353457	1.398254
WLC VS AE	Completion rate	0.015252	0.7045418	0.02	0.983	-1.365625	1.396129
WLC VS AE-CT	Completion rate	0.0291375	0.7017283	0.04	0.967	-1.346225	1.4045
WLC VS CT	Completion rate	0.0075333	0.7140784	0.01	0.992	-1.392035	1.407101
WLC VS MBE	Completion rate	0.0894167	0.7046883	0.13	0.899	-1.291747	1.47058
WLC VS MBE-CT	Completion rate	-0.1016341	0.7368976	-0.14	0.89	-1.545927	1.342659
WLC VS RT	Completion rate	0.0489369	0.0596758	0.82	0.412	-0.0680256	0.1658993

Supplementary Table 55. Meta-regression analysis of the DRA outcome with completion rate as a moderator in older adults with mild cognitive impairment.

Intervention	Covariate	Coefficient	Standard Error	Z-statistic	P>z	lower confidence interval	upper confidence interval
WLC VS AC	Completion rate	-0.0390539	0.0296225	-1.32	0.187	-0.097113	0.0190051
WLC VS AE	Completion rate	-0.0598454	0.0263884	-2.27	0.423	-0.1115657	-0.008125
WLC VS CT	Completion rate	-0.061472	0.0357317	-1.72	0.085	-0.1315047	0.0085608
WLC VS D/VRE	Completion rate	0.0248787	0.0337154	0.74	0.461	-0.0412022	0.0909596
WLC VS MBE	Completion rate	-0.065943	0.0358655	-1.84	0.066	-0.136238	0.004352
WLC VS ME	Completion rate	-0.0461583	0.1138828	-0.41	0.685	-0.2693644	0.1770479
WLC VS ME-CT	Completion rate	-0.0454595	0.092764	-0.49	0.624	-0.2272736	0.1363545

Supplementary Table 56. Meta-regression analysis of the MFA outcome with completion rate as a moderator in older adults with mild cognitive impairment.

Intervention	Covariate	Coefficient	Standard Error	Z-statistic	P>z	lower confidence interval	upper confidence interval
WLC VS AC	Completion rate	0.0399783	0.6666061	0.06	0.952	-1.266546	1.346502
WLC VS AE	Completion rate	0.0559079	0.6666538	0.08	0.933	-1.250709	1.362525
WLC VS CT	Completion rate	-0.4102223	1.431351	-0.29	0.774	-3.215618	2.395173

Supplementary Table 57. Meta-regression analysis of the MoCA outcome with supervision status as a moderator in older adults with mild cognitive impairment.

Intervention	Covariate	Coefficient	Standard Error	Z-statistic	P>z	lower confidence interval	upper confidence interval
WLC VS AC	Supervision status	-0.8930943	14.72859	-0.06	0.952	-29.76061	27.97442
WLC VS AE	Supervision status	-1.159361	0.9867944	-1.17	0.24	-3.093442	0.7747208
WLC VS AE-CT	Supervision status	-0.6418364	1.123057	-0.57	0.568	-2.842988	1.559315
WLC VS CT	Supervision status	-0.9090552	14.74317	-0.06	0.951	-29.80514	27.98703
WLC VS D/VRE	Supervision status	0.0821269	14.72561	0.01	0.996	-28.77955	28.9438
WLC VS MBE	Supervision status	0.1161638	0.6665088	0.17	0.862	-1.190169	1.422497
WLC VS ME	Supervision status	-0.6782625	14.72296	-0.05	0.963	-29.53474	28.17822
WLC VS RT	Supervision status	-0.0996512	0.7930686	-0.13	0.9	-1.654037	1.454735

Supplementary Table 58. Meta-regression analysis of the MMSE outcome with supervision status as a moderator in older adults with mild cognitive impairment.

Intervention	Covariate	Coefficient	Standard Error	Z-statistic	P>z	lower confidence interval	upper confidence interval
WLC VS AC	Supervision status	-0.5396963	0.5959523	-0.91	0.365	-1.707741	0.6283488

WLC VS AE	Supervision status	-0.749516	0.3379802	-2.22	0.187	-1.411945	-0.0870869
WLC VS CT	Supervision status	-0.9902415	0.993682	-1	0.319	-2.937822	0.9573394
WLC VS ME	Supervision status	-1.649896	0.7142379	-2.31	0.221	-3.049776	-0.2500154

Supplementary Table 59. Meta-regression analysis of the TMT-B outcome with supervision status as a moderator in older adults with mild cognitive impairment.

Intervention	Covariate	Coefficient	Standard Error	Z-statistic	P>z	lower confidence interval	upper confidence interval
WLC VS CT	Supervision status	7.242693	593.5957	0.01	0.99	-1156.183	1170.669
WLC VS MBE	Supervision status	9.730398	593.5796	0.02	0.987	-1153.664	1173.125

Supplementary Table 60. Meta-regression analysis of the DST outcome with supervision status as a moderator in older adults with mild cognitive impairment.

Intervention	Covariate	Coefficient	Standard Error	Z-statistic	P>z	lower confidence interval	upper confidence interval
WLC VS AC	Supervision status	-0.2063511	11.18208	-0.02	0.985	-22.12283	21.71012
WLC VS MBE	Supervision status	-0.478983	11.18283	-0.04	0.966	-22.39692	21.43895
WLC VS RT	Supervision status	-0.1261401	0.1663421	-0.76	0.448	-0.4521646	0.1998845

Supplementary Table 61. Meta-regression analysis of the DRA outcome with supervision status as a moderator in older adults with mild cognitive impairment.

Intervention	Covariate	Coefficient	Standard Error	Z-statistic	P>z	lower confidence interval	upper confidence interval
WLC VS AC	Supervision status	0.0867483	7.90572	0.01	0.991	-15.40818	15.58168
WLC VS AE	Supervision status	-0.0395658	7.905933	-0.01	0.996	-15.53491	15.45578
WLC VS CT	Supervision status	0.251272	7.905863	0.03	0.975	-15.24393	15.74648
WLC VS MBE	Supervision status	0.2645644	7.906271	0.03	0.973	-15.23144	15.76057

Supplementary Table 62. Meta-regression analysis of the MFA outcome with supervision status as a moderator in older adults with mild cognitive impairment.

Intervention	Covariate	Coefficient	Standard Error	Z-statistic	P>z	lower confidence interval	upper confidence interval
WLC VS AC	Supervision status	0.0371854	7.908983	0	0.996	-15.46414	15.53851
WLC VS AE	Supervision status	-0.2717376	7.90824	-0.03	0.973	-15.7716	15.22813
WLC VS CT	Supervision status	0.1455497	15.812	0.01	0.993	-30.8454	31.1365

Supplementary Table 63. Meta-regression analysis of the MoCA outcome with level of social interaction as a moderator in older adults with mild cognitive impairment.

Intervention	Covariate	Coefficient	Standard Error	Z-statistic	P>z	lower confidence interval	upper confidence interval
WLC VS AC	Level of social interaction	0.0853532	0.4391482	0.19	0.846	-0.7753614	0.9460679
WLC VS AE	Level of social interaction	0.2516584	0.3669287	0.69	0.493	-0.4675086	0.9708255
WLC VS AE-CT	Level of social interaction	0.2450444	0.7943959	0.31	0.758	-1.311943	1.802032
WLC VS CT	Level of social interaction	0.21765	1.000658	0.22	0.828	-1.743604	2.178904
WLC VS D/VRE	Level of social interaction	0.377152	0.6542565	0.58	0.564	-0.9051672	1.659471
WLC VS MBE	Level of social interaction	0.5883402	1.084722	0.54	0.588	-1.537676	2.714356
WLC VS ME	Level of social interaction	-0.1732979	0.7603208	-0.23	0.82	-1.663499	1.316903
WLC VS RT	Level of social interaction	2.112981	1.780779	1.19	0.235	-1.377282	5.603244

Supplementary Table 64. Meta-regression analysis of the MMSE outcome with level of social interaction as a moderator in older adults with mild cognitive impairment.

Intervention	Covariate	Coefficient	Standard Error	Z-statistic	P>z	lower confidence interval	upper confidence interval
WLC VS AC	Level of social interaction	-0.2883799	0.3097097	-0.93	0.352	-0.8953998	0.3186401
WLC VS AE	Level of social interaction	-0.2470858	0.279712	-0.88	0.377	-0.7953112	0.3011395
WLC VS CT	Level of social interaction	-3.845602	1.863288	-2.06	0.239	-7.49758	-0.1936243
WLC VS MBE	Level of social interaction	0.7410764	1.53342	0.48	0.629	-2.264372	3.746525
WLC VS ME	Level of social interaction	-2.988731	0.8730493	-3.42	0.486	-4.699876	-1.277586
WLC VS ME-CT	Level of social interaction	-1.877459	0.9230332	-2.03	0.542	-3.686571	-0.0683475

Supplementary Table 65. Meta-regression analysis of the TMT-B outcome with level of social interaction as a moderator in older adults with mild cognitive impairment.

Intervention	Covariate	Coefficient	Standard Error	Z-statistic	P>z	lower confidence interval	upper confidence interval
WLC VS AC	Level of social interaction	35.58635	39.44963	0.9	0.367	-41.73351	112.9062

WLC VS AE-CT	Level of social interaction	19.51516	43.77706	0.45	0.656	-66.28631	105.3166
WLC VS CT	Level of social interaction	4.600547	51.29631	0.09	0.929	-95.93837	105.1395
WLC VS MBE	Level of social interaction	50.26653	81.67149	0.62	0.538	-109.8067	210.3397
WLC VS ME	Level of social interaction	37.07395	42.21694	0.88	0.38	-45.66974	119.8176

Supplementary Table 66. Meta-regression analysis of the DST outcome with level of social interaction as a moderator in older adults with mild cognitive impairment.

Intervention	Covariate	Coefficient	Standard Error	Z-statistic	P>z	lower confidence interval	upper confidence interval
WLC VS AC	Level of social interaction	-0.1429803	5.678108	-0.03	0.98	-11.27187	10.98591
WLC VS AE	Level of social interaction	-0.1263031	5.678738	-0.02	0.982	-11.25642	11.00382
WLC VS AE-CT	Level of social interaction	-0.0553576	5.681456	-0.01	0.992	-11.19081	11.08009
WLC VS CT	Level of social interaction	0.0955637	5.680572	0.02	0.987	-11.03815	11.22928

WLC VS MBE	Level of social interaction	-0.3464943	5.678818	-0.06	0.951	-11.47677	10.78378
WLC VS MBE-CT	Level of social interaction	-0.4669355	5.68816	-0.08	0.935	-11.61552	10.68165
WLC VS RT	Level of social interaction	0.3135969	0.1335098	2.35	0.946	0.0519225	0.5752712

Supplementary Table 67. Meta-regression analysis of the DRA outcome with level of social interaction as a moderator in older adults with mild cognitive impairment.

Intervention	Covariate	Coefficient	Standard Error	Z-statistic	P>z	lower confidence interval	upper confidenc e interval
WLC VS AC	Level of social interaction	0.0561371	3.372023	0.02	0.987	-6.552907	6.665181
WLC VS AE	Level of social interaction	0.1275895	3.373153	0.04	0.97	-6.483669	6.738848
WLC VS CT	Level of social interaction	0.1738763	3.385609	0.05	0.959	-6.461796	6.809549
WLC VS D/VRE	Level of social interaction	-0.0570434	3.374274	-0.02	0.987	-6.670499	6.556412
WLC VS MBE	Level of social interaction	0.2589944	3.40902	0.08	0.939	-6.422561	6.94055

WLC VS ME	Level of social interaction	0.8703933	3.423261	0.25	0.799	-5.839074	7.579861
WLC VS ME-CT	Level of social interaction	0.7988446	3.425066	0.23	0.816	-5.914161	7.511851

Supplementary Table 68. Meta-regression analysis of the MFA outcome with level of social interaction as a moderator in older adults with mild cognitive impairment.

Intervention	Covariate	Coefficient	Standard Error	Z-statistic	P>z	lower confidence interval	upper confidenc e interval
WLC VS AC	Level of social interaction	0.0359355	3.39149	0.01	0.992	-6.611263	6.683134
WLC VS AE	Level of social interaction	-0.1068252	3.391035	-0.03	0.975	-6.753132	6.539481
WLC VS CT	Level of social interaction	0.4394409	3.437314	0.13	0.898	-6.29757	7.176452

Supplementary Table 69. Meta-regression analysis of the MoCA outcome with level of cognitive engagement as a moderator in older adults with mild cognitive impairment.

Intervention	Covariate	Coefficient	Standard Error	Z-statistic	P>z	lower confidence interval	upper confidenc e interval
WLC VS AC	Level of cognitive engagement	-0.3647487	0.645041	-0.57	0.572	-1.629006	0.8995084

WLC VS AE	Level of cognitive engagement	0.0372665	0.5706318	0.07	0.948	-1.081151	1.155684
WLC VS AE-CT	Level of cognitive engagement	-0.4597453	2.041213	-0.23	0.822	-4.460449	3.540958
WLC VS D/VRE	Level of cognitive engagement	-2.014691	2.160342	-0.93	0.351	-6.248883	2.219501
WLC VS ME	Level of cognitive engagement	-0.0842913	1.080994	-0.08	0.938	-2.203001	2.034418
WLC VS RT	Level of cognitive engagement	0.4000627	0.5979585	0.67	0.503	-0.7719145	1.57204

Supplementary Table 70. Meta-regression analysis of the MMSE outcome with level of cognitive engagement as a moderator in older adults with mild cognitive impairment.

**Intervention**	**Covariate**	**Coefficient**	**Standard Error**	**Z-statistic**	**P>z**	**lower confidence interval**	**upper confidence interval**
WLC VS AC	Level of cognitive engagement	-0.620666	0.5062461	-1.23	0.22	-1.61289	0.371558
WLC VS AE	Level of cognitive engagement	-0.4751176	0.3490481	-1.36	0.173	-1.159239	0.2090041
WLC VS ME	Level of cognitive engagement	-0.8359641	0.6075108	-1.38	0.169	-2.026663	0.3547352

Supplementary Table 71. Meta-regression analysis of the TMT-B outcome with level of cognitive engagement as a moderator in older adults with mild cognitive impairment.

Intervention	Covariate	Coefficient	Standard Error	Z-statistic	P>z	lower confidence interval	upper confidence interval
WLC VS AC	Level of cognitive engagement	-2.555188	591.8062	0	0.997	-1162.474	1157.364
WLC VS CT	Level of cognitive engagement	13.44795	1187.436	0.01	0.991	-2313.884	2340.78
WLC VS MBE	Level of cognitive engagement	19.8134	1187.405	0.02	0.987	-2307.457	2347.084
WLC VS ME	Level of cognitive engagement	36.18238	593.105	0.06	0.951	-1126.282	1198.647

Supplementary Table 72. Meta-regression analysis of the DST outcome with level of cognitive engagement as a moderator in older adults with mild cognitive impairment.

Intervention	Covariate	Coefficient	Standard Error	Z-statistic	P>z	lower confidence interval	upper confidence interval
WLC VS AC	Level of cognitive engagement	-0.0998456	10.54306	-0.01	0.992	-20.76386	20.56417
WLC VS AE	Level of cognitive engagement	-0.0776127	10.54329	-0.01	0.994	-20.74208	20.58686
WLC VS RT	Level of cognitive engagement	0.2490399	0.1253045	1.99	0.983	0.0034476	0.4946323

Supplementary Table 73. Meta-regression analysis of the DRA outcome with level of cognitive engagement as a moderator in older adults with mild cognitive impairment.

Intervention	Covariate	Coefficient	Standard Error	Z-statistic	P>z	lower confidence interval	upper confidence interval
WLC VS AC	Level of cognitive engagement	-0.4207214	0.3573712	-1.18	0.239	-1.121156	0.2797133
WLC VS AE	Level of cognitive engagement	-0.112744	0.3032466	-0.37	0.71	-0.7070964	0.4816083
WLC VS CT	Level of cognitive engagement	-0.2610682	0.6146059	-0.42	0.671	-1.465674	0.9435371
WLC VS D/VRE	Level of cognitive engagement	0.5786844	0.5560297	1.04	0.298	-0.5111138	1.668483
WLC VS MBE	Level of cognitive engagement	-0.0011312	0.6172453	0	0.999	-1.21091	1.208647
WLC VS ME	Level of cognitive engagement	-0.0249285	0.1670907	-0.15	0.881	-0.3524203	0.3025633

Supplementary Table 74. Meta-regression analysis of the MFA outcome with level of cognitive engagement as a moderator in older adults with mild cognitive impairment.

Intervention	Covariate	Coefficient	Standard Error	Z-statistic	P>z	lower confidence interval	upper confidence interval
WLC VS AC	Level of cognitive engagement	0.0303609	5.200682	0.01	0.995	-10.16279	10.22351

WLC VS AE	Level of cognitive engagement	-0.1755895	5.200132	-0.03	0.973	-10.36766	10.01648
WLC VS CT	Level of cognitive engagement	0.2911098	31.63297	0.01	0.993	-61.70837	62.29059

Supplementary Table 75. Meta-regression analysis of the MoCA outcome with exercise intensity as a moderator in older adults with mild cognitive impairment.

Intervention	Covariate	Coefficient	Standard Error	Z-statistic	P>z	lower confidence interval	upper confidence interval
WLC VS AC	Exercise intensity	-0.0628536	0.5705848	-0.11	0.912	-1.181179	1.055472
WLC VS AE	Exercise intensity	-0.602699	0.7238827	-0.83	0.405	-2.021483	0.816085
WLC VS AE-CT	Exercise intensity	0.6853227	2.448662	0.28	0.78	-4.113967	5.484613
WLC VS CT	Exercise intensity	-0.1798496	0.9886205	-0.18	0.856	-2.11751	1.757811
WLC VS D/VRE	Exercise intensity	-0.8158843	0.7299735	-1.12	0.264	-2.246606	0.6148375
WLC VS MBE	Exercise intensity	-0.4150149	1.03876	-0.4	0.69	-2.450947	1.620917
WLC VS RT	Exercise intensity	0.0588871	0.9878888	0.06	0.952	-1.877339	1.995114

Supplementary Table 76. Meta-regression analysis of the MMSE outcome with exercise intensity as a moderator in older adults with mild cognitive impairment.

Intervention	Covariate	Coefficient	Standard Error	Z-statistic	P>z	lower confidence interval	upper confidence interval
WLC VS AC	Exercise intensity	0.86894	0.3552696	2.45	0.314	0.1726244	1.565256
WLC VS AE	Exercise intensity	0.301564	0.3834896	0.79	0.432	-0.4500618	1.05319
WLC VS MBE	Exercise intensity	1.898148	1.687425	1.12	0.261	-1.409145	5.205441
WLC VS ME	Exercise intensity	-0.9039699	0.6964926	-1.3	0.194	-2.26907	0.4611305

Supplementary Table 77. Meta-regression analysis of the TMT-B outcome with exercise intensity as a moderator in older adults with mild cognitive impairment.

Intervention	Covariate	Coefficient	Standard Error	Z-statistic	P>z	lower confidence interval	upper confidence interval
WLC VS AC	Exercise intensity	22.49801	10.93547	2.06	0.748	1.064893	43.93113
WLC VS AE	Exercise intensity	5.57291	14.97631	0.37	0.71	-23.78012	34.92594
WLC VS AE-CT	Exercise intensity	-9.641859	37.07487	-0.26	0.795	-82.30728	63.02356
WLC VS CT	Exercise intensity	18.61401	1229.754	0.02	0.988	-2391.66	2428.888
WLC VS MBE	Exercise intensity	20.18302	614.8004	0.03	0.974	-1184.804	1225.17

Supplementary Table 78. Meta-regression analysis of the DST outcome with exercise intensity as a moderator in older adults with mild cognitive impairment.

Intervention	Covariate	Coefficient	Standard Error	Z-statistic	P>z	lower confidence interval	upper confidence interval
WLC VS AC	Exercise intensity	-0.3836485	6.90057	-0.06	0.956	-13.90852	13.14122
WLC VS AE	Exercise intensity	-0.3169508	6.912921	-0.05	0.963	-13.86603	13.23212
WLC VS AE-CT	Exercise intensity	-0.2088801	6.907222	-0.03	0.976	-13.74679	13.32903
WLC VS CT	Exercise intensity	-0.1954232	6.899697	-0.03	0.977	-13.71858	13.32774
WLC VS MBE-CT	Exercise intensity	-0.5065332	6.908197	-0.07	0.942	-14.04635	13.03328
WLC VS RT	Exercise intensity	0.9319891	0.3643881	2.56	0.953	0.2178016	1.646177

Supplementary Table 79. Meta-regression analysis of the DRA outcome with exercise intensity as a moderator in older adults with mild cognitive impairment.

Intervention	Covariate	Coefficient	Standard Error	Z-statistic	P>z	lower confidence interval	upper confidence interval
WLC VS AC	Exercise intensity	0.2257494	0.1811115	1.25	0.213	-0.1292226	0.5807214
WLC VS AE	Exercise intensity	-0.0750719	0.2344909	-0.32	0.749	-0.5346657	0.3845219
WLC VS CT	Exercise intensity	0.1226421	0.4936689	0.25	0.804	-0.8449312	1.090215
WLC VS D/VRE	Exercise intensity	0.1162349	15.82889	0.01	0.994	-30.90782	31.14029

WLC VS MBE	Exercise intensity	0.7239921	0.5814192	1.25	0.213	-0.4155685	1.863553
WLC VS ME-CT	Exercise intensity	0.7258922	31.65704	0.02	0.982	-61.32077	62.77255

Supplementary Table 80. Meta-regression analysis of the MFA outcome with exercise intensity as a moderator in older adults with mild cognitive impairment.

Intervention	Covariate	Coefficient	Standard Error	Z-statistic	P>z	lower confidence interval	upper confidence interval
WLC VS AC	Exercise intensity	-0.0556691	4.518941	-0.01	0.99	-8.912631	8.801293
WLC VS AE	Exercise intensity	0.2532562	4.520849	0.06	0.955	-8.607445	9.113958
WLC VS CT	Exercise intensity	-0.204948	36.45409	-0.01	0.996	-71.65366	71.24376

Supplementary Table 81. Meta-regression analysis of the MoCA outcome with intervention dose as a moderator in older adults with mild cognitive impairment.

Intervention	Covariate	Coefficient	Standard Error	Z-statistic	P>z	lower confidence interval	upper confidence interval
WLC VS AC	Intervention dose	-0.0032951	0.0041676	-0.79	0.429	-0.0114635	0.0048733
WLC VS AE	Intervention dose	-0.0010429	0.0061085	-0.17	0.864	-0.0130153	0.0109296
WLC VS AE-CT	Intervention dose	-0.0032575	0.0081881	-0.4	0.691	-0.019306	0.0127909
WLC VS CT	Intervention dose	-0.0047806	0.0090551	-0.53	0.598	-0.0225283	0.0129671

WLC VS D/VRE	Intervention dose	0.0087266	0.0086769	1.01	0.315	-0.0082798	0.0257329
WLC VS MBE	Intervention dose	-0.0046618	0.0051399	-0.91	0.364	-0.0147358	0.0054122
WLC VS ME	Intervention dose	-0.0061039	0.0087985	-0.69	0.488	-0.0233486	0.0111407
WLC VS RT	Intervention dose	0.0067531	0.0110813	0.61	0.542	-0.0149658	0.028472

Supplementary Table 82. Meta-regression analysis of the MMSE outcome with intervention dose as a moderator in older adults with mild cognitive impairment.

Intervention	Covariate	Coefficient	Standard Error	Z-statistic	P>z	lower confidence interval	upper confidence interval
WLC VS AC	Intervention dose	0.0000583	0.0028585	0.02	0.984	-0.0055443	0.005661
WLC VS AE	Intervention dose	0.0059693	0.0049215	1.21	0.225	-0.0036767	0.0156153
WLC VS MBE	Intervention dose	-0.0174462	0.0327358	-0.53	0.594	-0.0816073	0.0467149
WLC VS ME	Intervention dose	-0.0041315	0.0036756	-1.12	0.261	-0.0113355	0.0030726
WLC VS ME-CT	Intervention dose	0.0232548	0.0428566	0.54	0.587	-0.0607426	0.1072523

Supplementary Table 83. Meta-regression analysis of the TMT-B outcome with intervention dose as a moderator in older adults with mild cognitive impairment.

Intervention	Covariate	Coefficient	Standard Error	Z-statistic	P>z	lower confidence interval	upper confidence interval
WLC VS AC	Intervention dose	-0.1836266	0.1098769	-1.67	0.095	-0.3989813	0.0317281
WLC VS AE	Intervention dose	-0.3690509	0.2045436	-1.8	0.071	-0.769949	0.0318471
WLC VS AE-CT	Intervention dose	-0.0283585	0.21486	-0.13	0.895	-0.4494763	0.3927593
WLC VS CT	Intervention dose	-0.1377252	0.127126	-1.08	0.279	-0.3868875	0.1114372
WLC VS MBE	Intervention dose	-0.3516585	0.3039427	-1.16	0.247	-0.9473753	0.2440583
WLC VS ME	Intervention dose	-0.0472479	0.2589811	-0.18	0.855	-0.5548416	0.4603458

Supplementary Table 84. Meta-regression analysis of the DST outcome with intervention dose as a moderator in older adults with mild cognitive impairment.

Intervention	Covariate	Coefficient	Standard Error	Z-statistic	P>z	lower confidence interval	upper confidence interval
WLC VS AC	Intervention dose	0.0021305	0.0323823	0.07	0.948	-0.0613376	0.0655987
WLC VS AE	Intervention dose	0.0015012	0.0326215	0.05	0.963	-0.0624357	0.0654381
WLC VS AE-CT	Intervention dose	0.0012839	0.0324485	0.04	0.968	-0.0623139	0.0648817
WLC VS CT	Intervention dose	0.0012727	0.0323771	0.04	0.969	-0.0621852	0.0647306

WLC VS MBE	Intervention dose	0.0151385	0.0328332	0.46	0.645	-0.0492135	0.0794904
WLC VS MBE-CT	Intervention dose	0.0002007	0.0325294	0.01	0.995	-0.0635559	0.0639572
WLC VS RT	Intervention dose	-0.0023984	0.0021595	-1.11	0.267	-0.0066311	0.0018342

Supplementary Table 85. Meta-regression analysis of the DRA outcome with intervention dose as a moderator in older adults with mild cognitive impairment.

Intervention	Covariate	Coefficient	Standard Error	Z-statistic	P>z	lower confidence interval	upper confidenc e interval
WLC VS AC	Intervention dose	-0.0014408	0.0012589	-1.14	0.252	-0.0039083	0.0010266
WLC VS AE	Intervention dose	-0.0013018	0.0016533	-0.79	0.431	-0.0045423	0.0019387
WLC VS CT	Intervention dose	-0.001602	0.0018005	-0.89	0.374	-0.0051309	0.001927
WLC VS D/VRE	Intervention dose	-0.0107943	0.0729062	-0.15	0.882	-0.1536879	0.1320993
WLC VS MBE	Intervention dose	-0.005802	0.00721	-0.8	0.421	-0.0199334	0.0083294
WLC VS ME	Intervention dose	-0.0003696	0.0032634	-0.11	0.91	-0.0067656	0.0060265
WLC VS ME-CT	Intervention dose	0.0025648	0.0354449	0.07	0.942	-0.066906	0.0720355

Supplementary Table 86. Meta-regression analysis of the MFA outcome with intervention dose as a moderator in older adults with mild cognitive impairment.

Intervention	Covariate	Coefficient	Standard Error	Z-statistic	P>z	lower confidence interval	upper confidence interval
WLC VS AC	Intervention dose	-0.0001404	0.0402526	0	0.997	-0.079034	0.0787531
WLC VS AE	Intervention dose	-0.0041012	0.0402902	-0.1	0.919	-0.0830685	0.0748661
WLC VS CT	Intervention dose	0.0001983	0.1257646	0	0.999	-0.2462958	0.2466924

Supplementary Table 87. Sensitivity analysis for the MoCA outcome in older adults with mild cognitive impairment.

dropped_id	comparison	eff	lci	uci	connected
Lan Li	WLC VS AC	0.7625485	-0.4503708	1.975468	1
Lan Li	WLC VS AE	2.502663	1.399234	3.606091	1
Lan Li	WLC VS AE-CT	3.2909527	1.333309	5.248597	1
Lan Li	WLC VS CT	1.6494039	0.1924986	3.106309	1
Lan Li	WLC VS D/VRE	1.3593583	-0.5463385	3.265055	1
Lan Li	WLC VS MBE	3.9862946	2.296633	5.675956	1
Lan Li	WLC VS MBE-CT	-0.23747178	-3.248083	2.773139	1
Lan Li	WLC VS ME	0.93419562	-0.7561519	2.624543	1
Lan Li	WLC VS ME-CT	-0.26075065	-3.975784	3.454283	1
Lan Li	WLC VS RT	1.3818449	-0.1836299	2.94732	1
Chang J.	WLC VS AC	0.99057029	-0.1857426	2.166883	1
Chang J.	WLC VS AE	2.8902579	1.746584	4.033932	1
Chang J.	WLC VS AE-CT	3.4912008	1.630281	5.35212	1
Chang J.	WLC VS CT	1.8237254	0.440571	3.20688	1
Chang J.	WLC VS D/VRE	1.5909753	-0.193732	3.375683	1
Chang J.	WLC VS MBE	4.1186205	2.512047	5.725194	1
Chang J.	WLC VS MBE-CT	-0.00946272	-2.820306	2.80138	1
Chang J.	WLC VS ME	1.374634	-0.1227518	2.87202	1
Chang J.	WLC VS ME-CT	-0.02915805	-3.533419	3.475103	1
Chang J.	WLC VS RT	1.5084131	0.0345308	2.982295	1

Lina Wang	WLC VS AC	0.74975092	-0.4663404	1.965842	1
Lina Wang	WLC VS AE	2.4959885	1.389101	3.602876	1
Lina Wang	WLC VS AE-CT	3.2825704	1.31929	5.245851	1
Lina Wang	WLC VS CT	1.6449997	0.1835979	3.106401	1
Lina Wang	WLC VS D/VRE	1.3760281	-0.5352481	3.287304	1
Lina Wang	WLC VS MBE	3.979405	2.28503	5.67378	1
Lina Wang	WLC VS MBE-CT	-0.25026848	-3.271399	2.770862	1
Lina Wang	WLC VS ME	0.98509669	-0.7044458	2.674639	1
Lina Wang	WLC VS ME-CT	-0.24408455	-3.969471	3.481302	1
Lina Wang	WLC VS RT	1.3785584	-0.1918692	2.948986	1
Imran Amjad	WLC VS AC	0.73103269	-0.4950601	1.957126	1
Imran Amjad	WLC VS AE	2.4680393	1.358002	3.578077	1
Imran Amjad	WLC VS AE-CT	3.2654302	1.309034	5.221826	1
Imran Amjad	WLC VS CT	1.6440565	0.181861	3.106252	1
Imran Amjad	WLC VS D/VRE	1.4335865	-0.4625683	3.329741	1
Imran Amjad	WLC VS MBE	3.9603269	2.268936	5.651718	1
Imran Amjad	WLC VS MBE-CT	-0.26898503	-3.282401	2.744431	1
Imran Amjad	WLC VS ME	1.1416138	-0.4415411	2.724769	1
Imran Amjad	WLC VS ME-CT	-0.18653553	-3.894633	3.521562	1
Imran Amjad	WLC VS RT	1.3712222	-0.1925911	2.935035	1
Dan Song.	WLC VS AC	0.77688583	-0.4608783	2.01465	1
Dan Song.	WLC VS AE	2.4386	1.322868	3.554332	1
Dan Song.	WLC VS AE-CT	3.2868414	1.327686	5.245996	1
Dan Song.	WLC VS CT	1.6719709	0.2050958	3.138846	1
Dan Song.	WLC VS D/VRE	1.4556989	-0.4441272	3.355525	1
Dan Song.	WLC VS MBE	3.9915825	2.297104	5.686061	1
Dan Song.	WLC VS MBE-CT	-0.22313558	-3.243653	2.797381	1
Dan Song.	WLC VS ME	1.1770691	-0.4135452	2.767683	1
Dan Song.	WLC VS ME-CT	-0.1644272	-3.876302	3.547447	1
Dan Song.	WLC VS RT	1.3756312	-0.1895227	2.940785	1
Soon-Gook Hong	WLC VS AC	0.72172885	-0.4779197	1.921377	1
Soon-Gook Hong	WLC VS AE	2.4819175	1.38639	3.577445	1
Soon-Gook Hong	WLC VS AE-CT	3.2614737	1.320133	5.202814	1
Soon-Gook Hong	WLC VS CT	1.6426178	0.1907396	3.094496	1
Soon-Gook Hong	WLC VS D/VRE	1.4329189	-0.4467976	3.312635	1
Soon-Gook Hong	WLC VS MBE	3.9478733	2.272651	5.623096	1
Soon-Gook Hong	WLC VS MBE-CT	-0.27828781	-3.258536	2.70196	1
Soon-Gook Hong	WLC VS ME	1.1375898	-0.424551	2.699731	1
Soon-Gook Hong	WLC VS ME-CT	-0.18720125	-3.868682	3.49428	1

Soon-Gook Hong	WLC VS RT	1.3968151	-0.273404	3.067034	1
Yi Zhu	WLC VS AC	0.64174061	-0.5900337	1.873515	1
Yi Zhu	WLC VS AE	2.5254467	1.413657	3.637236	1
Yi Zhu	WLC VS AE-CT	3.2237482	1.268887	5.178609	1
Yi Zhu	WLC VS CT	1.589699	0.1274266	3.051971	1
Yi Zhu	WLC VS D/VRE	1.3904812	-0.5035612	3.284523	1
Yi Zhu	WLC VS MBE	3.8995112	2.206324	5.592698	1
Yi Zhu	WLC VS MBE-CT	-0.35826996	-3.369681	2.653141	1
Yi Zhu	WLC VS ME	1.0725074	-0.5107347	2.65575	1
Yi Zhu	WLC VS ME-CT	-0.22963288	-3.933137	3.473872	1
Yi Zhu	WLC VS RT	1.3626751	-0.1988576	2.924208	1
Lazarou	WLC VS AC	0.28969055	-0.7118709	1.291252	1
Lazarou	WLC VS AE	1.8071569	0.8552763	2.759037	1
Lazarou	WLC VS AE-CT	2.840676	1.221751	4.459601	1
Lazarou	WLC VS CT	1.3723964	0.1880752	2.556718	1
Lazarou	WLC VS D/VRE	1.2854032	-0.2475076	2.818314	1
Lazarou	WLC VS MBE	3.385156	1.966214	4.804098	1
Lazarou	WLC VS MBE-CT	-0.71029891	-3.076094	1.655496	1
Lazarou	WLC VS ME	0.8764296	-0.3800974	2.132957	1
Lazarou	WLC VS ME-CT	-0.33466255	-3.420388	2.751063	1
Lazarou	WLC VS RT	1.1385166	-0.1230158	2.400049	1
Yoon	WLC VS AC	0.69414151	-0.5168973	1.90518	1
Yoon	WLC VS AE	2.4686913	1.377174	3.560209	1
Yoon	WLC VS AE-CT	3.2422644	1.301572	5.182957	1
Yoon	WLC VS CT	1.628807	0.1860209	3.071593	1
Yoon	WLC VS D/VRE	1.4211506	-0.4546873	3.296988	1
Yoon	WLC VS MBE	3.9252292	2.249108	5.60135	1
Yoon	WLC VS MBE-CT	-0.30587291	-3.28463	2.672884	1
Yoon	WLC VS ME	1.1170368	-0.4478111	2.681885	Yoon
Yoon	WLC VS ME-CT	-0.19896705	-3.87353	3.475596	Yoon
Yoon	WLC VS RT	1.4154986	-0.1932963	3.024293	Yoon
Nascimento	WLC VS AC	0.87651194	-0.3602827	2.113307	1
Nascimento	WLC VS AE	2.5635474	1.465714	3.661381	1
Nascimento	WLC VS AE-CT	3.367481	1.428518	5.306444	1
Nascimento	WLC VS CT	1.753638	0.2989556	3.20832	1
Nascimento	WLC VS D/VRE	1.5973188	-0.2993211	3.493959	1
Nascimento	WLC VS MBE	4.0566508	2.375087	5.738214	1
Nascimento	WLC VS MBE-CT	-0.12351626	-3.092297	2.845264	1
Nascimento	WLC VS ME	1.4807599	-0.245502	3.207022	1

Nascimento	WLC VS ME-CT	-0.02282768	-3.691412	3.645757	1
Nascimento	WLC VS RT	1.4241697	-0.1177887	2.966128	1
Wang Shuo	WLC VS AC	0.78274738	-0.4441949	2.00969	1
Wang Shuo	WLC VS AE	2.5800824	1.410219	3.749946	1
Wang Shuo	WLC VS AE-CT	3.3171998	1.357729	5.27667	1
Wang Shuo	WLC VS CT	1.6797581	0.2206523	3.138864	1
Wang Shuo	WLC VS D/VRE	1.4617777	-0.4266696	3.350225	1
Wang Shuo	WLC VS MBE	4.0017354	2.309184	5.694287	1
Wang Shuo	WLC VS MBE-CT	-0.21727422	-3.217352	2.782804	1
Wang Shuo	WLC VS ME	1.1840775	-0.3941893	2.762344	1
Wang Shuo	WLC VS ME-CT	-0.15834828	-3.851389	3.534693	1
Wang Shuo	WLC VS RT	1.4041516	-0.1582401	2.966543	1
Rojasavastera	WLC VS AC	0.86233073	-0.397565	2.122226	1
Rojasavastera	WLC VS AE	2.6958689	1.541019	3.850718	1
Rojasavastera	WLC VS AE-CT	3.4126046	0.8109721	6.014237	1
Rojasavastera	WLC VS CT	1.7325473	0.2626045	3.20249	1
Rojasavastera	WLC VS D/VRE	1.5021125	-0.3915005	3.395726	1
Rojasavastera	WLC VS MBE	4.0677271	2.35972	5.775734	1
Rojasavastera	WLC VS MBE-CT	-0.13769717	-3.151994	2.8766	1
Rojasavastera	WLC VS ME	1.2464283	-0.3468471	2.839704	1
Rojasavastera	WLC VS ME-CT	-0.11802057	-3.814189	3.578148	1
Rojasavastera	WLC VS RT	1.4439356	-0.1194574	3.007329	1
Parial	WLC VS AC	0.69215151	-0.5296825	1.913985	1
Parial	WLC VS AE	2.473119	1.372637	3.573601	1
Parial	WLC VS AE-CT	3.4112913	1.032276	5.790307	1
Parial	WLC VS CT	1.6198067	0.158407	3.081206	1
Parial	WLC VS D/VRE	1.4143821	-0.4813295	3.310094	1
Parial	WLC VS MBE	3.9330053	2.241483	5.624527	1
Parial	WLC VS MBE-CT	-0.3078631	-3.319911	2.704184	1
Parial	WLC VS ME	1.1112005	-0.4699165	2.692317	1
Parial	WLC VS ME-CT	-0.2057365	-3.913901	3.502428	1
Parial	WLC VS RT	1.3636591	-0.2011344	2.928453	1
Ming Qi	WLC VS AC	0.60643763	-0.6070822	1.819957	1
Ming Qi	WLC VS AE	2.5482011	1.451604	3.644798	1
Ming Qi	WLC VS AE-CT	3.205545	1.271893	5.139197	1
Ming Qi	WLC VS CT	1.5692849	0.1246459	3.013924	1
Ming Qi	WLC VS D/VRE	1.3769685	-0.4949251	3.248862	1
Ming Qi	WLC VS MBE	3.8657912	2.191575	5.540008	1
Ming Qi	WLC VS MBE-CT	-0.39356993	-3.364852	2.577712	1

Ming Qi	WLC VS ME	1.0491415	-0.5131078	2.611391	1
Ming Qi	WLC VS ME-CT	-0.24314083	-3.908813	3.422531	1
Ming Qi	WLC VS RT	1.3594056	-0.184385	2.903196	1
Danny J. Yu	WLC VS AC	0.78881411	-0.4017745	1.979403	1
Danny J. Yu	WLC VS AE	2.4316351	1.348347	3.514923	1
Danny J. Yu	WLC VS AE-CT	3.289739	1.367393	5.212085	1
Danny J. Yu	WLC VS CT	1.6810222	0.2490897	3.112955	1
Danny J. Yu	WLC VS D/VRE	1.468041	-0.3914813	3.327563	1
Danny J. Yu	WLC VS MBE	3.9831118	2.321458	5.644765	1
Danny J. Yu	WLC VS MBE-CT	-0.21120734	-3.158717	2.736302	1
Danny J. Yu	WLC VS ME	1.1938705	-0.3514808	2.739222	1
Danny J. Yu	WLC VS ME-CT	-0.15208301	-3.799745	3.495579	1
Danny J. Yu	WLC VS RT	1.3766675	-0.1591596	2.912494	1
Fuzhong Li	WLC VS AC	0.58377533	-0.5713074	1.738858	1
Fuzhong Li	WLC VS AE	2.4252983	1.379643	3.470954	1
Fuzhong Li	WLC VS AE-CT	3.1621931	1.294253	5.030133	1
Fuzhong Li	WLC VS CT	1.5556368	0.1671282	2.944145	1
Fuzhong Li	WLC VS D/VRE	1.3771688	-0.4243849	3.178723	1
Fuzhong Li	WLC VS MBE	4.5714777	2.810632	6.332324	1
Fuzhong Li	WLC VS MBE-CT	-0.41623001	-3.260282	2.427821	1
Fuzhong Li	WLC VS ME	1.0451819	-0.4494242	2.539788	1
Fuzhong Li	WLC VS ME-CT	-0.24293353	-3.789406	3.303539	1
Fuzhong Li	WLC VS RT	1.3305596	-0.1573778	2.818497	1
Angus P. Yu	WLC VS AC	0.82556791	-0.2869958	1.938132	1
Angus P. Yu	WLC VS AE	2.5147787	1.504457	3.5251	1
Angus P. Yu	WLC VS AE-CT	3.3179915	1.505832	5.13015	1
Angus P. Yu	WLC VS CT	1.7123002	0.3697039	3.054897	1
Angus P. Yu	WLC VS D/VRE	1.5112985	-0.232029	3.254626	1
Angus P. Yu	WLC VS MBE	3.3690531	1.765023	4.973083	1
Angus P. Yu	WLC VS MBE-CT	-0.17445297	-2.914834	2.565929	1
Angus P. Yu	WLC VS ME	1.2506558	-0.189112	2.690423	1
Angus P. Yu	WLC VS ME-CT	-0.10881879	-3.556816	3.339178	1
Angus P. Yu	WLC VS RT	1.3990131	-0.0411948	2.839221	1
Jurakic	WLC VS AC	0.84510664	-0.3357802	2.025993	1
Jurakic	WLC VS AE	2.5434434	1.477212	3.609675	1
Jurakic	WLC VS AE-CT	3.3423924	1.445484	5.239301	1
Jurakic	WLC VS CT	1.5856413	0.1765125	2.99477	1
Jurakic	WLC VS D/VRE	0.70780081	-1.457319	2.87292	1
Jurakic	WLC VS MBE	4.0171529	2.378927	5.655379	1

Jurakic	WLC VS MBE-CT	-0.15491813	-3.052215	2.742379	1
Jurakic	WLC VS ME	1.5330845	-0.1037801	3.169949	1
Jurakic	WLC VS ME-CT	-0.91219253	-4.689031	2.864646	1
Jurakic	WLC VS RT	1.3835421	-0.1272367	2.894321	1
Jorge Buele	WLC VS AC	0.71744661	-0.4633428	1.898236	1
Jorge Buele	WLC VS AE	2.4772292	1.399052	3.555406	1
Jorge Buele	WLC VS AE-CT	3.2563189	1.337043	5.175595	1
Jorge Buele	WLC VS CT	1.637602	0.2094315	3.065772	1
Jorge Buele	WLC VS D/VRE	1.4334765	-0.4234644	3.290417	1
Jorge Buele	WLC VS MBE	3.9345402	2.279129	5.589951	1
Jorge Buele	WLC VS MBE-CT	-0.28256934	-3.223384	2.658245	1
Jorge Buele	WLC VS ME	1.1383713	-0.4022742	2.679017	1
Jorge Buele	WLC VS ME-CT	1.3699146	-0.1643605	2.90419	1
Carvalho	WLC VS AC	0.70691401	-0.4998284	1.913656	1
Carvalho	WLC VS AE	2.4721494	1.371027	3.573271	1
Carvalho	WLC VS AE-CT	3.2520106	1.297418	5.206603	1
Carvalho	WLC VS CT	1.5857561	0.0448279	3.126684	1
Carvalho	WLC VS D/VRE	1.5399217	-0.6893244	3.769168	1
Carvalho	WLC VS MBE	3.943444	2.257379	5.629509	1
Carvalho	WLC VS MBE-CT	-0.29310178	-3.298895	2.712692	1
Carvalho	WLC VS ME	1.1451825	-0.4354094	2.725774	1
Carvalho	WLC VS ME-CT	-0.08021926	-3.969284	3.808846	1
Carvalho	WLC VS RT	1.3582414	-0.2100423	2.926525	1
Miaoran Lin	WLC VS AC	0.66317035	-0.5482179	1.874559	1
Miaoran Lin	WLC VS AE	2.4580904	1.35744	3.558741	1
Miaoran Lin	WLC VS AE-CT	3.223402	1.268205	5.178599	1
Miaoran Lin	WLC VS CT	1.6012893	0.1434807	3.059098	1
Miaoran Lin	WLC VS D/VRE	1.400017	-0.4937499	3.293784	1
Miaoran Lin	WLC VS MBE	4.2041231	2.383393	6.024854	1
Miaoran Lin	WLC VS MBE-CT	-0.33684196	-3.343887	2.670203	1
Miaoran Lin	WLC VS ME	1.0886347	-0.4876318	2.664901	1
Miaoran Lin	WLC VS ME-CT	-0.22009899	-3.926636	3.486439	1
Miaoran Lin	WLC VS RT	1.3543974	-0.2095764	2.918371	1
Chunhui Zhou	WLC VS AC	0.63269391	-0.6330672	1.898455	1
Chunhui Zhou	WLC VS AE	2.4274849	1.308364	3.546606	1
Chunhui Zhou	WLC VS AE-CT	3.1981572	1.236289	5.160026	1
Chunhui Zhou	WLC VS CT	1.5821994	0.1141597	3.050239	1
Chunhui Zhou	WLC VS D/VRE	1.3873443	-0.5004277	3.275116	1
Chunhui Zhou	WLC VS MBE	3.7300186	1.728491	5.731547	1

Chunhui Zhou	WLC VS MBE-CT	-0.36731578	-3.373419	2.638788	1
Chunhui Zhou	WLC VS ME	1.0677768	-0.5232686	2.658822	1
Chunhui Zhou	WLC VS ME-CT	-0.23276782	-3.917256	3.45172	1
Chunhui Zhou	WLC VS RT	1.341819	-0.2153648	2.899003	1
Elmar Graessel	WLC VS AC	0.69554071	-0.5301655	1.921247	1
Elmar Graessel	WLC VS AE	2.4674796	1.359613	3.575346	1
Elmar Graessel	WLC VS AE-CT	3.2447348	1.281149	5.20832	1
Elmar Graessel	WLC VS CT	1.6808919	0.1378217	3.223962	1
Elmar Graessel	WLC VS D/VRE	1.4364422	-0.4645614	3.337446	1
Elmar Graessel	WLC VS MBE	3.9369772	2.240399	5.633555	1
Elmar Graessel	WLC VS MBE-CT	-0.30447425	-3.324637	2.715688	1
Elmar Graessel	WLC VS ME	1.117008	-0.4640612	2.698077	1
Elmar Graessel	WLC VS ME-CT	-0.18368084	-3.899867	3.532505	1
Elmar Graessel	WLC VS RT	1.3749229	-0.1934741	2.94332	1
Goumopoulos	WLC VS AC	0.68314399	-0.5163065	1.882595	1
Goumopoulos	WLC VS AE	2.4605434	1.369942	3.551145	1
Goumopoulos	WLC VS AE-CT	3.2341906	1.296368	5.172014	1
Goumopoulos	WLC VS CT	1.7130744	0.2389881	3.187161	1
Goumopoulos	WLC VS D/VRE	1.4482175	-0.4269263	3.323361	1
Goumopoulos	WLC VS MBE	3.9168331	2.245025	5.588642	1
Goumopoulos	WLC VS MBE-CT	-0.3168696	-3.291256	2.657517	1
Goumopoulos	WLC VS ME	1.1145444	-0.4435522	2.672641	1
Goumopoulos	WLC VS ME-CT	-0.17190491	-3.846373	3.502563	1
Goumopoulos	WLC VS RT	1.3785457	-0.1702352	2.927326	1
Qiang Zhang	WLC VS AC	0.7033338	-0.5032913	1.909959	1
Qiang Zhang	WLC VS AE	2.5099273	1.406294	3.61356	1
Qiang Zhang	WLC VS AE-CT	3.2566157	1.309986	5.203246	1
Qiang Zhang	WLC VS CT	1.6281837	0.176646	3.079721	1
Qiang Zhang	WLC VS D/VRE	1.4217562	-0.4639645	3.307477	1
Qiang Zhang	WLC VS MBE	3.8078139	2.02335	5.592278	1
Qiang Zhang	WLC VS MBE-CT	-0.29668155	-3.289465	2.696102	1
Qiang Zhang	WLC VS ME	1.1217262	-0.4475922	2.691045	1
Qiang Zhang	WLC VS ME-CT	-0.19836273	-3.890785	3.49406	1
Qiang Zhang	WLC VS RT	1.3732071	-0.1837713	2.930186	1
Kevser Gursan	WLC VS AC	0.77972368	-0.4919803	2.051428	1
Kevser Gursan	WLC VS AE	2.550693	1.425787	3.675599	1
Kevser Gursan	WLC VS AE-CT	3.31025	1.337257	5.283243	1
Kevser Gursan	WLC VS CT	1.6569897	-0.108772	3.422751	1
Kevser Gursan	WLC VS D/VRE	1.4516207	-0.4970766	3.400318	1

Kevser Gursan	WLC VS MBE	4.0007678	2.289439	5.712097	1
Kevser Gursan	WLC VS MBE-CT	-0.22029788	-3.250154	2.809559	1
Kevser Gursan	WLC VS ME	1.1787844	-0.4370627	2.794631	1
Kevser Gursan	WLC VS ME-CT	-0.16850429	-3.901785	3.564776	1
Kevser Gursan	WLC VS RT	1.9758534	-0.0280016	3.979708	1
Hiroshi Hayashi	WLC VS AC	0.87339211	-0.3375908	2.084375	1
Hiroshi Hayashi	WLC VS AE	2.5627193	1.478707	3.646732	1
Hiroshi Hayashi	WLC VS AE-CT	3.3643554	1.444253	5.284458	1
Hiroshi Hayashi	WLC VS CT	1.8544153	0.3759551	3.332875	1
Hiroshi Hayashi	WLC VS D/VRE	2.1998414	-0.1481394	4.547822	1
Hiroshi Hayashi	WLC VS MBE	4.0476716	2.386362	5.708981	1
Hiroshi Hayashi	WLC VS MBE-CT	-0.12663536	-3.063595	2.810324	1
Hiroshi Hayashi	WLC VS ME	1.3926627	-0.2123671	2.997692	1
Hiroshi Hayashi	WLC VS ME-CT	0.57959436	-3.325392	4.484581	1
Hiroshi Hayashi	WLC VS RT	1.4433348	-0.0885234	2.975193	1
Kang	WLC VS AC	0.68215967	-0.5124606	1.87678	1
Kang	WLC VS AE	2.4598976	1.372727	3.547068	1
Kang	WLC VS AE-CT	3.233086	1.300462	5.16571	1
Kang	WLC VS CT	1.716111	0.2537176	3.178504	1
Kang	WLC VS D/VRE	1.4498122	-0.4200407	3.319665	1
Kang	WLC VS MBE	3.9137914	2.246958	5.580625	1
Kang	WLC VS MBE-CT	-0.31785376	-3.282919	2.647212	1
Kang	WLC VS ME	1.1149846	-0.4384564	2.668426	1
Kang	WLC VS ME-CT	-0.17030989	-3.836114	3.495494	1
Kang	WLC VS RT	1.378885	-0.1658104	2.92358	1
Rivas-Campo Y.	WLC VS AC	0.74963816	-0.4688345	1.968111	1
Rivas-Campo Y.	WLC VS AE	2.4960034	1.387429	3.604578	1
Rivas-Campo Y.	WLC VS AE-CT	3.2826922	1.316918	5.248466	1
Rivas-Campo Y.	WLC VS CT	1.6448618	0.181586	3.108138	1
Rivas-Campo Y.	WLC VS D/VRE	1.3758381	-0.5387402	3.290416	1
Rivas-Campo Y.	WLC VS MBE	3.9803985	2.283972	5.676825	1
Rivas-Campo Y.	WLC VS MBE-CT	-0.25038128	-3.276057	2.775294	1
Rivas-Campo Y.	WLC VS ME	0.98521211	-0.716328	2.686752	1
Rivas-Campo Y.	WLC VS ME-CT	-0.24427477	-3.974263	3.485713	1
Rivas-Campo Y.	WLC VS RT	1.3785496	-0.1938604	2.95096	1
Eun Hee Lim	WLC VS AC	0.64417413	-0.5620383	1.850387	1
Eun Hee Lim	WLC VS AE	2.4364368	1.345047	3.527826	1
Eun Hee Lim	WLC VS AE-CT	3.2060381	1.270267	5.141809	1
Eun Hee Lim	WLC VS CT	1.4704738	-0.0420511	2.982999	1

Eun Hee Lim	WLC VS D/VRE	1.3518264	-0.5317929	3.235446	1
Eun Hee Lim	WLC VS MBE	3.884713	2.215215	5.554211	1
Eun Hee Lim	WLC VS MBE-CT	-0.35583635	-3.325307	2.613634	1
Eun Hee Lim	WLC VS ME	1.0702855	-0.4935546	2.634126	1
Eun Hee Lim	WLC VS ME-CT	-0.26827867	-3.940892	3.404335	1
Eun Hee Lim	WLC VS RT	1.3225564	-0.2273711	2.872484	1
Kitsana Krootnark	WLC VS AC	0.49606012	-0.6878846	1.680005	1
Kitsana Krootnark	WLC VS AE	2.2225581	1.076479	3.368637	1
Kitsana Krootnark	WLC VS AE-CT	3.0710515	1.184838	4.957265	1
Kitsana Krootnark	WLC VS CT	1.407799	-0.0020556	2.817654	1
Kitsana Krootnark	WLC VS D/VRE	1.2997382	-0.5099505	3.109427	1
Kitsana Krootnark	WLC VS MBE	3.729934	2.095321	5.364547	1
Kitsana Krootnark	WLC VS MBE-CT	-0.50393898	-3.363917	2.356039	1
Kitsana Krootnark	WLC VS ME	0.96912615	-0.5410265	2.479279	1
Kitsana Krootnark	WLC VS ME-CT	-0.32035182	-3.874248	3.233544	1
Kitsana Krootnark	WLC VS RT	0.61822813	-1.251404	2.48786	1
Givon Schaham	WLC VS AC	0.74081654	-0.4801685	1.961802	1
Givon Schaham	WLC VS AE	2.49014	1.38504	3.59524	1
Givon Schaham	WLC VS AE-CT	3.2756362	1.316088	5.235185	1
Givon Schaham	WLC VS CT	1.5779763	0.0491542	3.106798	1
Givon Schaham	WLC VS D/VRE	1.4124376	-0.4846659	3.309541	1
Givon Schaham	WLC VS MBE	3.9695865	2.277383	5.66179	1
Givon Schaham	WLC VS MBE-CT	-0.25920198	-3.272413	2.754009	1
Givon Schaham	WLC VS ME	1.1441203	-0.433769	2.72201	1
Givon Schaham	WLC VS ME-CT	-0.20768083	-3.91778	3.502419	1
Givon Schaham	WLC VS RT	1.3633983	-0.2021294	2.928926	1
Meixiang Fan	WLC VS AC	0.71743524	-0.4633595	1.89823	1
Meixiang Fan	WLC VS AE	2.4772226	1.399047	3.555398	1
Meixiang Fan	WLC VS AE-CT	3.2563103	1.337038	5.175583	1
Meixiang Fan	WLC VS CT	1.6375817	0.2094228	3.065741	1
Meixiang Fan	WLC VS D/VRE	1.4333933	-0.4234267	3.290213	1
Meixiang Fan	WLC VS MBE	3.9345281	2.279119	5.589937	1
Meixiang Fan	WLC VS MBE-CT	1.138349	-0.4022837	2.678982	1
Meixiang Fan	WLC VS ME	-0.18672458	-3.830781	3.457332	1
Meixiang Fan	WLC VS ME-CT	1.3699082	-0.1643612	2.904178	1
Hong Yu	WLC VS AC	0.73419869	-0.464609	1.933006	1
Hong Yu	WLC VS AE	2.4808072	1.390452	3.571163	1
Hong Yu	WLC VS AE-CT	3.1450355	1.068419	5.221653	1
Hong Yu	WLC VS CT	1.6471555	0.2021977	3.092113	1

Hong Yu	WLC VS D/VRE	1.4380841	-0.4394965	3.315665	1
Hong Yu	WLC VS MBE	3.9562468	2.281994	5.6305	1
Hong Yu	WLC VS MBE-CT	-0.26581893	-3.244197	2.712559	1
Hong Yu	WLC VS ME	1.1473309	-0.4134214	2.708083	1
Hong Yu	WLC VS ME-CT	-0.18203682	-3.861189	3.497115	1
Hong Yu	WLC VS RT	1.3743395	-0.1766204	2.925299	1

Supplementary Table 88. Sensitivity analysis for the MMSE outcome in older adults with mild cognitive impairment.

dropped_id	comparison	eff	lci	uci	connected
Lan Li	WLC VS AC	2.3583031	1.184156	3.53245	1
Lan Li	WLC VS AE	2.1359026	1.171248	3.100557	1
Lan Li	WLC VS AE-CT	0.40000005	-1.453093	2.253093	1
Lan Li	WLC VS CT	2.3483478	0.8378596	3.858836	1
Lan Li	WLC VS D/VRE	2.6100001	0.8190677	4.400932	1
Lan Li	WLC VS MBE	4.7275612	2.742742	6.71238	1
Lan Li	WLC VS MBE-CT	2.557878	0.3846666	4.73109	1
Lan Li	WLC VS ME	3.552889	2.309816	4.795962	1
Lan Li	WLC VS ME-CT	3.6529759	1.939732	5.36622	1
Lan Li	WLC VS RT	1.563642	-0.5466518	3.673936	1
Lan Li	WLC VS RT-CT	3.563642	1.482056	5.645228	1
Tomoto T.	WLC VS AC	2.1370113	0.7401575	3.533865	1
Tomoto T.	WLC VS AE	2.0489539	0.9295227	3.168385	1
Tomoto T.	WLC VS AE-CT	0.4	-1.820054	2.620054	1
Tomoto T.	WLC VS CT	2.258962	0.5676553	3.950269	1
Tomoto T.	WLC VS D/VRE	2.61	0.441561	4.778439	1
Tomoto T.	WLC VS MBE	4.5437384	2.231929	6.855548	1
Tomoto T.	WLC VS MBE-CT	2.336454	-0.2691648	4.942073	1
Tomoto T.	WLC VS ME	3.9024577	2.488023	5.316893	1
Tomoto T.	WLC VS ME-CT	3.7123995	1.66346	5.761339	1
Tomoto T.	WLC VS RT	1.7682997	-0.730921	4.26752	1
Tomoto T.	WLC VS RT-CT	3.7682997	1.293271	6.243328	1
Kerime Bademli	WLC VS AC	1.9715893	0.5250553	3.418123	1
Kerime Bademli	WLC VS AE	1.9911848	0.8727697	3.1096	1
Kerime Bademli	WLC VS AE-CT	0.4	-1.787197	2.587197	1
Kerime Bademli	WLC VS CT	2.1613056	0.4635051	3.859106	1
Kerime Bademli	WLC VS D/VRE	2.61	0.4752123	4.744788	1

Kerime Bademli	WLC VS MBE	4.3753946	2.046684	6.704106	1
Kerime Bademli	WLC VS MBE-CT	2.1710885	-0.4338339	4.776011	1
Kerime Bademli	WLC VS ME	3.6461946	1.923412	5.368977	1
Kerime Bademli	WLC VS ME-CT	3.5024767	1.347709	5.657244	1
Kerime Bademli	WLC VS RT	1.5352169	-1.082992	4.153426	1
Kerime Bademli	WLC VS RT-CT	3.5352169	0.9400904	6.130343	1
Imran Amjad	WLC VS AC	2.4339883	1.175809	3.692168	1
Imran Amjad	WLC VS AE	1.8900811	0.8672453	2.912917	1
Imran Amjad	WLC VS AE-CT	0.4	-1.593825	2.393826	1
Imran Amjad	WLC VS CT	2.4070794	0.8256446	3.988514	1
Imran Amjad	WLC VS D/VRE	2.61	0.6738114	4.546189	1
Imran Amjad	WLC VS MBE	4.8188144	2.705034	6.932594	1
Imran Amjad	WLC VS MBE-CT	2.6334802	0.2950506	4.97191	1
Imran Amjad	WLC VS ME	4.0915443	2.803808	5.379281	1
Imran Amjad	WLC VS ME-CT	3.9537058	2.102499	5.804913	1
Imran Amjad	WLC VS RT	1.9838788	-0.2763972	4.244155	1
Imran Amjad	WLC VS RT-CT	3.9838788	1.750382	6.217376	1
Lazarou	WLC VS AC	2.0708332	0.7203204	3.421346	1
Lazarou	WLC VS AE	1.9732688	0.73823	3.208308	1
Lazarou	WLC VS AE-CT	0.4	-1.808558	2.608558	1
Lazarou	WLC VS CT	2.2201279	0.5435982	3.896657	1
Lazarou	WLC VS D/VRE	2.61	0.4533328	4.766667	1
Lazarou	WLC VS MBE	4.4765477	2.19725	6.755846	1
Lazarou	WLC VS MBE-CT	2.270298	-0.3009033	4.841499	1
Lazarou	WLC VS ME	3.8573985	2.466038	5.248759	1
Lazarou	WLC VS ME-CT	3.6563609	1.635756	5.676966	1
Lazarou	WLC VS RT	1.7178357	-0.7572136	4.192885	1
Lazarou	WLC VS RT-CT	3.7178357	1.267217	6.168454	1
Takehiko Doi	WLC VS AC	2.1659004	0.7760655	3.555735	1
Takehiko Doi	WLC VS AE	2.0345091	0.9186108	3.150408	1
Takehiko Doi	WLC VS AE-CT	0.4	-1.813511	2.613511	1
Takehiko Doi	WLC VS CT	2.2747561	0.5876207	3.961892	1
Takehiko Doi	WLC VS D/VRE	2.61	0.4482605	4.771739	1
Takehiko Doi	WLC VS MBE	4.5720416	2.26793	6.876153	1
Takehiko Doi	WLC VS MBE-CT	2.3653393	-0.2309474	4.961626	1
Takehiko Doi	WLC VS ME	3.9215626	2.512229	5.330896	1
Takehiko Doi	WLC VS ME-CT	3.736437	1.694734	5.77814	1
Takehiko Doi	WLC VS RT	1.7898646	-0.7014239	4.281153	1
Takehiko Doi	WLC VS RT-CT	3.7898646	1.322846	6.256883	1

Wang Shuo	WLC VS AC	2.1081978	0.7693741	3.447021	1
Wang Shuo	WLC VS AE	2.0347336	0.8320549	3.237412	1
Wang Shuo	WLC VS AE-CT	0.4	-1.805735	2.605735	1
Wang Shuo	WLC VS CT	2.2411203	0.5691603	3.91308	1
Wang Shuo	WLC VS D/VRE	2.61	0.4562231	4.763777	1
Wang Shuo	WLC VS MBE	4.5136538	2.243247	6.78406	1
Wang Shuo	WLC VS MBE-CT	2.3076548	-0.2550027	4.870312	1
Wang Shuo	WLC VS ME	3.8823834	2.497331	5.267436	1
Wang Shuo	WLC VS ME-CT	3.6876573	1.674441	5.700873	1
Wang Shuo	WLC VS RT	1.7459532	-0.7228302	4.214736	1
Wang Shuo	WLC VS RT-CT	3.7459532	1.301663	6.190243	1
Lam	WLC VS AC	1.8213728	0.6780466	2.964699	1
Lam	WLC VS AE	1.9000947	0.9580984	2.842091	1
Lam	WLC VS AE-CT	0.4	-1.375559	2.175559	1
Lam	WLC VS CT	2.021846	0.5348099	3.508882	1
Lam	WLC VS D/VRE	2.61	0.8994167	4.320583	1
Lam	WLC VS MBE	4.1814053	2.247622	6.115189	1
Lam	WLC VS MBE-CT	2.0210679	-0.0694078	4.111544	1
Lam	WLC VS ME	4.2218927	3.02204	5.421746	1
Lam	WLC VS ME-CT	3.6929145	2.039284	5.346545	1
Lam	WLC VS RT	1.9213504	-0.1105269	3.953228	1
Lam	WLC VS RT-CT	3.9213504	1.919305	5.923396	1
Langoni	WLC VS AC	2.0231185	0.6806462	3.365591	1
Langoni	WLC VS AE	2.0120164	0.9245299	3.099503	1
Langoni	WLC VS AE-CT	0.4	-1.767207	2.567207	1
Langoni	WLC VS CT	2.1885609	0.5292348	3.847887	1
Langoni	WLC VS D/VRE	2.61	0.4956982	4.724302	1
Langoni	WLC VS MBE	4.4250856	2.173866	6.676305	1
Langoni	WLC VS MBE-CT	2.2226146	-0.3089087	4.754138	1
Langoni	WLC VS ME	3.7302832	2.239679	5.220888	1
Langoni	WLC VS ME-CT	3.5695424	1.540391	5.598693	1
Langoni	WLC VS RT	1.6108786	-0.8737587	4.095516	1
Langoni	WLC VS RT-CT	3.6108786	1.150577	6.07118	1
Kohanpour	WLC VS AC	2.4518156	1.260491	3.64314	1
Kohanpour	WLC VS AE	2.586374	1.491975	3.680773	1
Kohanpour	WLC VS AE-CT	0.39999999	-1.457568	2.257568	1
Kohanpour	WLC VS CT	2.4037343	0.8877628	3.919706	1
Kohanpour	WLC VS D/VRE	2.61	0.8144377	4.405562	1
Kohanpour	WLC VS MBE	4.8215821	2.824326	6.818839	1

Kohanpour	WLC VS MBE-CT	2.6513721	0.4650371	4.837707	1
Kohanpour	WLC VS ME	4.096277	2.881568	5.310987	1
Kohanpour	WLC VS ME-CT	3.9627728	2.225153	5.700393	1
Kohanpour	WLC VS RT	1.9911922	-0.1287969	4.111181	1
Kohanpour	WLC VS RT-CT	3.9911922	1.899777	6.082607	1
Mei-yi Siu	WLC VS AC	2.1200871	0.8308106	3.409364	1
Mei-yi Siu	WLC VS AE	2.053602	0.9750521	3.132152	1
Mei-yi Siu	WLC VS AE-CT	0.4	-1.775902	2.575902	1
Mei-yi Siu	WLC VS CT	2.2448544	0.5959848	3.893724	1
Mei-yi Siu	WLC VS D/VRE	2.61	0.4867863	4.733214	1
Mei-yi Siu	WLC VS MBE	5.1890413	1.879654	8.498428	1
Mei-yi Siu	WLC VS MBE-CT	2.319556	-0.1917001	4.830812	1
Mei-yi Siu	WLC VS ME	3.8888883	2.534483	5.243293	1
Mei-yi Siu	WLC VS ME-CT	3.6965225	1.724244	5.668801	1
Mei-yi Siu	WLC VS RT	1.753709	-0.6737267	4.181145	1
Mei-yi Siu	WLC VS RT-CT	3.753709	1.351188	6.156229	1
Phoemsapthawee J.	WLC VS AC	2.1359864	0.8313831	3.44059	1
Phoemsapthawee J.	WLC VS AE	2.079879	0.9471009	3.212657	1
Phoemsapthawee J.	WLC VS AE-CT	0.4	-1.770995	2.570995	1
Phoemsapthawee J.	WLC VS CT	2.2534302	0.6020345	3.904826	1
Phoemsapthawee J.	WLC VS D/VRE	2.61	0.4918151	4.728185	1
Phoemsapthawee J.	WLC VS MBE	4.5382848	2.306242	6.770328	1
Phoemsapthawee J.	WLC VS MBE-CT	2.3354539	-0.1794646	4.850372	1
Phoemsapthawee J.	WLC VS ME	3.8993432	2.539569	5.259117	1
Phoemsapthawee J.	WLC VS ME-CT	3.7097045	1.733086	5.686323	1
Phoemsapthawee J.	WLC VS RT	1.7655276	-0.6624122	4.193468	1
Phoemsapthawee J.	WLC VS RT-CT	3.7655276	1.362497	6.168558	1
Silvia Varela	WLC VS AC	2.1396726	0.8496113	3.429734	1
Silvia Varela	WLC VS AE	2.0856114	0.9803595	3.190863	1
Silvia Varela	WLC VS AE-CT	0.4	-1.753652	2.553652	1
Silvia Varela	WLC VS CT	2.2537551	0.611886	3.895624	1
Silvia Varela	WLC VS D/VRE	2.61	0.5095941	4.710406	1
Silvia Varela	WLC VS MBE	4.540365	2.325912	6.754818	1
Silvia Varela	WLC VS MBE-CT	2.3391478	-0.1532664	4.831562	1
Silvia Varela	WLC VS ME	3.9009453	2.552642	5.249249	1
Silvia Varela	WLC VS ME-CT	3.7121351	1.752562	5.671709	1
Silvia Varela	WLC VS RT	1.7675885	-0.640777	4.175954	1
Silvia Varela	WLC VS RT-CT	3.7675885	1.384337	6.15084	1
Takao Suzuki	WLC VS AC	2.127943	0.8217625	3.434124	1

Takao Suzuki	WLC VS AE	2.0573148	0.9730399	3.14159	1
Takao Suzuki	WLC VS AE-CT	0.4	-1.783538	2.583538	1
Takao Suzuki	WLC VS CT	2.2501194	0.594382	3.905857	1
Takao Suzuki	WLC VS D/VRE	2.61	0.4789614	4.741039	1
Takao Suzuki	WLC VS MBE	4.5313907	2.292155	6.770627	1
Takao Suzuki	WLC VS MBE-CT	2.3274062	-0.1991596	4.853972	1
Takao Suzuki	WLC VS ME	3.8791961	2.499067	5.259326	1
Takao Suzuki	WLC VS ME-CT	3.6959568	1.717438	5.674475	1
Takao Suzuki	WLC VS RT	1.7485297	-0.6903366	4.187396	1
Takao Suzuki	WLC VS RT-CT	3.7485297	1.33446	6.162599	1
Xiu-hong Wei	WLC VS AC	2.1184895	0.8533896	3.383589	1
Xiu-hong Wei	WLC VS AE	2.0501485	0.9915336	3.108763	1
Xiu-hong Wei	WLC VS AE-CT	2.2383379	0.6138721	3.862804	1
Xiu-hong Wei	WLC VS CT	2.6099999	0.5418338	4.678166	1
Xiu-hong Wei	WLC VS D/VRE	4.5162214	2.333514	6.698929	1
Xiu-hong Wei	WLC VS MBE	2.3179847	-0.1343714	4.770341	1
Xiu-hong Wei	WLC VS MBE-CT	3.8850359	2.557319	5.212753	1
Xiu-hong Wei	WLC VS ME	3.6930616	1.763986	5.622138	1
Xiu-hong Wei	WLC VS ME-CT	1.7502025	-0.6229206	4.123326	1
Xiu-hong Wei	WLC VS RT	3.7502025	1.402571	6.097835	1
So Young Moon	WLC VS AC	2.0445065	0.7468132	3.3422	1
So Young Moon	WLC VS AE	2.0209658	0.943394	3.098537	1
So Young Moon	WLC VS AE-CT	0.4	-1.764754	2.564754	1
So Young Moon	WLC VS CT	2.2005181	0.5526278	3.848408	1
So Young Moon	WLC VS D/VRE	2.61	0.498213	4.721787	1
So Young Moon	WLC VS MBE	4.4462438	2.221521	6.670967	1
So Young Moon	WLC VS MBE-CT	2.2439987	-0.2619527	4.74995	1
So Young Moon	WLC VS ME	3.9863034	2.616083	5.356524	1
So Young Moon	WLC VS ME-CT	4.4038775	1.780306	7.027449	1
So Young Moon	WLC VS RT	2.1538775	-0.4554953	4.76325	1
So Young Moon	WLC VS RT-CT	4.1538775	1.567666	6.740088	1
İsmail Uysal	WLC VS AC	2.0454128	0.7475151	3.343311	1
İsmail Uysal	WLC VS AE	2.0213626	0.9436697	3.099056	1
İsmail Uysal	WLC VS AE-CT	0.4	-1.764991	2.564991	1
İsmail Uysal	WLC VS CT	2.2010613	0.5530182	3.849104	1
İsmail Uysal	WLC VS D/VRE	2.61	0.4979697	4.72203	1
İsmail Uysal	WLC VS MBE	4.4471719	2.222178	6.672166	1
İsmail Uysal	WLC VS MBE-CT	2.2449047	-0.2613573	4.751167	1
İsmail Uysal	WLC VS ME	3.9880574	2.617326	5.358789	1

İsmail Uysal	WLC VS ME-CT	3.0452104	0.5119728	5.578448	1
Qiang Zhang	WLC VS AC	2.1201754	0.830888	3.409463	1
Qiang Zhang	WLC VS AE	2.0536398	0.9750847	3.132195	1
Qiang Zhang	WLC VS AE-CT	0.4	-1.775908	2.575908	1
Qiang Zhang	WLC VS CT	2.2449054	0.596029	3.893782	1
Qiang Zhang	WLC VS D/VRE	2.61	0.4867801	4.73322	1
Qiang Zhang	WLC VS MBE	4.1598711	1.566601	6.753141	1
Qiang Zhang	WLC VS MBE-CT	2.3196443	-0.1916227	4.830911	1
Qiang Zhang	WLC VS ME	3.888948	2.534535	5.243361	1
Qiang Zhang	WLC VS ME-CT	3.696597	1.724309	5.668885	1
Qiang Zhang	WLC VS RT	1.753776	-0.673669	4.181221	1
Qiang Zhang	WLC VS RT-CT	3.753776	1.351246	6.156306	1
Mosayeb Mozafari	WLC VS AC	2.1184895	0.8533896	3.383589	1
Mosayeb Mozafari	WLC VS AE	2.0501485	0.9915336	3.108763	1
Mosayeb Mozafari	WLC VS AE-CT	0.39999992	-1.722222	2.522222	1
Mosayeb Mozafari	WLC VS CT	2.2383379	0.6138721	3.862804	1
Mosayeb Mozafari	WLC VS D/VRE	4.5162214	2.333514	6.698929	1
Mosayeb Mozafari	WLC VS MBE	2.3179847	-0.1343714	4.770341	1
Mosayeb Mozafari	WLC VS MBE-CT	3.8850359	2.557319	5.212753	1
Mosayeb Mozafari	WLC VS ME	3.6930616	1.763986	5.622138	1
Mosayeb Mozafari	WLC VS ME-CT	1.7502025	-0.6229206	4.123326	1
Mosayeb Mozafari	WLC VS RT	3.7502025	1.402571	6.097835	1
Petri	WLC VS AC	1.9073435	0.6296802	3.185007	1
Petri	WLC VS AE	1.9578411	0.9099757	3.005707	1
Petri	WLC VS AE-CT	0.4	-1.676797	2.476797	1
Petri	WLC VS CT	1.6297065	-0.1705531	3.429966	1
Petri	WLC VS D/VRE	2.61	0.5884725	4.631527	1
Petri	WLC VS MBE	4.300697	2.137135	6.464259	1
Petri	WLC VS MBE-CT	2.1069063	-0.3128804	4.526693	1
Petri	WLC VS ME	3.7400026	2.421777	5.058228	1
Petri	WLC VS ME-CT	3.5133072	1.606174	5.420441	1
Petri	WLC VS RT	1.5881206	-0.7485952	3.924836	1
Petri	WLC VS RT-CT	3.5881206	1.277298	5.898943	1
Sunyoung Kang	WLC VS AC	1.985518	0.6419774	3.329059	1
Sunyoung Kang	WLC VS AE	1.9961081	0.9079286	3.084288	1
Sunyoung Kang	WLC VS AE-CT	0.4	-1.768113	2.568113	1
Sunyoung Kang	WLC VS CT	2.5754706	0.6716045	4.479337	1
Sunyoung Kang	WLC VS D/VRE	2.61	0.4947696	4.72523	1
Sunyoung Kang	WLC VS MBE	4.3875745	2.134863	6.640286	1

Sunyoung Kang	WLC VS MBE-CT	2.1850226	-0.3478405	4.717886	1
Sunyoung Kang	WLC VS ME	3.7978911	2.422225	5.173557	1
Sunyoung Kang	WLC VS ME-CT	3.5830497	1.590021	5.576078	1
Sunyoung Kang	WLC VS RT	1.6516072	-0.7857534	4.088968	1
Sunyoung Kang	WLC VS RT-CT	3.6516072	1.239059	6.064156	1
Kang	WLC VS AC	2.0542576	0.7428777	3.365637	1
Kang	WLC VS AE	2.0256103	0.9433498	3.107871	1
Kang	WLC VS AE-CT	0.4	-1.774641	2.574641	1
Kang	WLC VS CT	2.4065645	0.6481851	4.164944	1
Kang	WLC VS D/VRE	2.61	0.4880787	4.731921	1
Kang	WLC VS MBE	4.4569036	2.220917	6.69289	1
Kang	WLC VS MBE-CT	2.2537428	-0.2678473	4.775333	1
Kang	WLC VS ME	3.8445122	2.481823	5.207201	1
Kang	WLC VS ME-CT	3.6411177	1.65992	5.622315	1
Kang	WLC VS RT	1.7038766	-0.728737	4.13649	1
Kang	WLC VS RT-CT	3.7038766	1.296124	6.111629	1
Eun Hee Lim	WLC VS AC	2.1196319	0.7639884	3.475276	1
Eun Hee Lim	WLC VS AE	2.0540439	0.9571412	3.150947	1
Eun Hee Lim	WLC VS AE-CT	0.4	-1.788675	2.588675	1
Eun Hee Lim	WLC VS CT	2.2444806	0.2325904	4.256371	1
Eun Hee Lim	WLC VS D/VRE	2.61	0.473698	4.746302	1
Eun Hee Lim	WLC VS MBE	4.523548	2.251647	6.795448	1
Eun Hee Lim	WLC VS MBE-CT	2.3190946	-0.2377716	4.875961	1
Eun Hee Lim	WLC VS ME	3.8892246	2.501547	5.276902	1
Eun Hee Lim	WLC VS ME-CT	3.6966266	1.685328	5.707925	1
Eun Hee Lim	WLC VS RT	1.7538949	-0.7054512	4.213241	1
Eun Hee Lim	WLC VS RT-CT	3.7538949	1.319137	6.188653	1
Meixiang Fan	WLC VS AC	2.1188532	0.8536139	3.384092	1
Meixiang Fan	WLC VS AE	2.0503068	0.9916378	3.108976	1
Meixiang Fan	WLC VS AE-CT	0.39999989	-1.722292	2.522291	1
Meixiang Fan	WLC VS CT	2.2385538	0.6140209	3.863087	1
Meixiang Fan	WLC VS D/VRE	2.6099999	0.5417621	4.678237	1
Meixiang Fan	WLC VS MBE	4.5165917	2.333756	6.699428	1
Meixiang Fan	WLC VS MBE-CT	3.8852848	2.55748	5.213089	1
Meixiang Fan	WLC VS ME	3.6933706	1.764184	5.622557	1
Meixiang Fan	WLC VS ME-CT	1.7504809	-0.622749	4.123711	1
Meixiang Fan	WLC VS RT	3.7504809	1.402741	6.098221	1

Supplementary Table 89. Sensitivity analysis for the TMT-B outcome in older adults with mild cognitive impairment.

dropped_id	comparison	eff	lci	uci	connected
Yi Zhu	WLC VS AC	-1.3864943	-34.12119	31.3482	1
Yi Zhu	WLC VS AE	-5.7275204	-39.90828	28.45324	1
Yi Zhu	WLC VS AE-CT	6.8297183	-36.56039	50.21983	1
Yi Zhu	WLC VS CT	-22.084016	-56.6292	12.46117	1
Yi Zhu	WLC VS D/VRE	-48.457655	-76.4814	-20.43391	1
Yi Zhu	WLC VS MBE	-15.326189	-46.85992	16.20754	1
Yi Zhu	WLC VS MBE-CT	-27.344918	-61.05529	6.365458	1
Yi Zhu	WLC VS ME	-15.174656	-48.45768	18.10836	1
Yi Zhu	WLC VS ME-CT	9.6133285	-33.60015	52.82681	1
Thaiyanto	WLC VS AC	-0.52304626	-34.00382	32.95773	1
Thaiyanto	WLC VS AE	-8.1335938	-43.46541	27.19823	1
Thaiyanto	WLC VS AE-CT	7.7443742	-36.7181	52.20684	1
Thaiyanto	WLC VS CT	-20.663587	-55.23177	13.90459	1
Thaiyanto	WLC VS D/VRE	-48.384758	-77.47818	-19.29133	1
Thaiyanto	WLC VS MBE	-15.501295	-47.59905	16.59646	1
Thaiyanto	WLC VS MBE-CT	-27.028974	-62.02827	7.970319	1
Thaiyanto	WLC VS ME	-15.795868	-50.0839	18.49216	1
Thaiyanto	WLC VS ME-CT	10.476728	-34.06419	55.01765	1
Marta Bisbe	WLC VS AC	-0.83722226	-34.11764	32.4432	1
Marta Bisbe	WLC VS AE	-7.9815677	-43.13705	27.17392	1
Marta Bisbe	WLC VS AE-CT	7.4202885	-36.78751	51.62809	1
Marta Bisbe	WLC VS CT	-21.009543	-55.46408	13.44499	1
Marta Bisbe	WLC VS D/VRE	-48.382099	-77.26832	-19.49588	1
Marta Bisbe	WLC VS MBE	-15.421871	-47.40583	16.56209	1
Marta Bisbe	WLC VS MBE-CT	-27.148463	-61.89109	7.594164	1
Marta Bisbe	WLC VS ME	-15.247953	-49.36452	18.86862	1
Marta Bisbe	WLC VS ME-CT	10.162568	-34.07824	54.40337	1
Takehiko Doi	WLC VS AC	-1.6197677	-33.98211	30.74258	1
Takehiko Doi	WLC VS AE	-21.052496	-59.37951	17.27451	1
Takehiko Doi	WLC VS AE-CT	6.572178	-36.28591	49.43027	1
Takehiko Doi	WLC VS CT	-22.720157	-56.82574	11.38543	1
Takehiko Doi	WLC VS D/VRE	-48.507492	-76.0274	-20.98758	1
Takehiko Doi	WLC VS MBE	-15.280376	-46.58376	16.02301	1
Takehiko Doi	WLC VS MBE-CT	-27.407092	-60.52923	5.715047	1
Takehiko Doi	WLC VS ME	-15.444378	-48.24262	17.35386	1

Takehiko Doi	WLC VS ME-CT	9.3800695	-33.19447	51.95461	1
Parial	WLC VS AC	-0.8140335	-34.2349	32.60683	1
Parial	WLC VS AE	-8.3950918	-43.76361	26.97343	1
Parial	WLC VS AE-CT	12.655739	-59.32862	84.6401	1
Parial	WLC VS CT	-20.835394	-55.42501	13.75422	1
Parial	WLC VS D/VRE	-48.350607	-77.43781	-19.26341	1
Parial	WLC VS MBE	-15.40278	-47.49392	16.68836	1
Parial	WLC VS MBE-CT	-27.137311	-62.12028	7.845656	1
Parial	WLC VS ME	-14.986234	-49.28989	19.31742	1
Parial	WLC VS ME-CT	10.185753	-34.30337	54.67487	1
Ming Qi	WLC VS AC	-1.0808077	-34.17558	32.01397	1
Ming Qi	WLC VS AE	-6.8695622	-41.70511	27.96599	1
Ming Qi	WLC VS AE-CT	7.1620615	-36.76379	51.08792	1
Ming Qi	WLC VS CT	-21.406639	-55.96921	13.15593	1
Ming Qi	WLC VS D/VRE	-48.404604	-76.98581	-19.8234	1
Ming Qi	WLC VS MBE	-15.377122	-47.19862	16.44437	1
Ming Qi	WLC VS MBE-CT	-27.240823	-61.61979	7.13814	1
Ming Qi	WLC VS ME	-15.076845	-48.89789	18.7442	1
Ming Qi	WLC VS ME-CT	9.918996	-33.96244	53.80044	1
Harris A. Eyre	WLC VS AC	0.32056205	-34.9406	35.58173	1
Harris A. Eyre	WLC VS AE	-8.9219364	-46.71054	28.86666	1
Harris A. Eyre	WLC VS AE-CT	8.6977133	-38.25987	55.6553	1
Harris A. Eyre	WLC VS CT	-17.24207	-56.13911	21.65496	1
Harris A. Eyre	WLC VS D/VRE	-48.384573	-79.87313	-16.89601	1
Harris A. Eyre	WLC VS MBE	-16.239695	-50.21486	17.73546	1
Harris A. Eyre	WLC VS MBE-CT	-27.022147	-64.84683	10.80254	1
Harris A. Eyre	WLC VS ME	-14.702278	-51.30554	21.90099	1
Harris A. Eyre	WLC VS ME-CT	11.320262	-36.24899	58.88951	1
Fuzhong Li	WLC VS AC	4.152574	-30.80596	39.11111	1
Fuzhong Li	WLC VS AE	-3.8795622	-40.96948	33.21036	1
Fuzhong Li	WLC VS AE-CT	12.441344	-33.3614	58.24409	1
Fuzhong Li	WLC VS CT	-17.72114	-52.82508	17.3828	1
Fuzhong Li	WLC VS D/VRE	-48.89229	-78.47485	-19.30974	1
Fuzhong Li	WLC VS MBE	-17.060115	-49.57528	15.45505	1
Fuzhong Li	WLC VS MBE-CT	-12.92254	-48.00619	22.16111	1
Fuzhong Li	WLC VS ME	15.152135	-30.82946	61.13373	1
Angus P. Yu	WLC VS AC	-2.0184055	-36.77931	32.7425	1
Angus P. Yu	WLC VS AE	-10.438805	-47.74241	26.8648	1
Angus P. Yu	WLC VS AE-CT	6.3059425	-39.6946	52.30648	1

Angus P. Yu	WLC VS CT	-20.586849	-55.979	14.8053	1
Angus P. Yu	WLC VS D/VRE	-47.985661	-78.31863	-17.65269	1
Angus P. Yu	WLC VS MBE	-14.803687	-47.64719	18.03982	1
Angus P. Yu	WLC VS MBE-CT	-27.529653	-64.03667	8.977358	1
Angus P. Yu	WLC VS ME	-11.957163	-52.12055	28.20622	1
Angus P. Yu	WLC VS ME-CT	8.9814233	-37.38707	55.34991	1
Chien-Liang Liu	WLC VS AC	38.058602	-14.22816	90.34536	1
Chien-Liang Liu	WLC VS AE	32.521503	-22.28282	87.32583	1
Chien-Liang Liu	WLC VS AE-CT	46.237049	-12.97232	105.4464	1
Chien-Liang Liu	WLC VS CT	15.291118	-35.64579	66.22803	1
Chien-Liang Liu	WLC VS D/VRE	25.563014	-27.63732	78.76334	1
Chien-Liang Liu	WLC VS MBE	12.811826	-40.71612	66.33977	1
Chien-Liang Liu	WLC VS MBE-CT	24.993697	-28.46154	78.44893	1
Chien-Liang Liu	WLC VS ME	49.056911	-9.969479	108.0833	1
Ha Yeong Choi	WLC VS AC	-0.95490145	-34.14607	32.23627	1
Ha Yeong Choi	WLC VS AE	-8.2783648	-43.34024	26.78351	1
Ha Yeong Choi	WLC VS AE-CT	7.2987377	-36.79928	51.39676	1
Ha Yeong Choi	WLC VS CT	-21.143305	-55.47993	13.19333	1
Ha Yeong Choi	WLC VS D/VRE	-48.3811	-77.18545	-19.57675	1
Ha Yeong Choi	WLC VS MBE	-15.390688	-47.33097	16.5496	1
Ha Yeong Choi	WLC VS MBE-CT	-27.192465	-61.83426	7.449327	1
Ha Yeong Choi	WLC VS ME	-15.046909	-49.07261	18.9788	1
Goumopoulos	WLC VS AC	0.17408284	-32.99903	33.3472	1
Goumopoulos	WLC VS AE	-6.380018	-40.53249	27.77246	1
Goumopoulos	WLC VS AE-CT	8.3921036	-35.35719	52.14139	1
Goumopoulos	WLC VS CT	-23.540503	-57.46424	10.38324	1
Goumopoulos	WLC VS D/VRE	-48.149394	-76.22412	-20.07467	1
Goumopoulos	WLC VS MBE	-14.466105	-46.03649	17.10428	1
Goumopoulos	WLC VS MBE-CT	-26.107104	-60.01601	7.801803	1
Goumopoulos	WLC VS ME	-13.973772	-47.45967	19.51213	1
Goumopoulos	WLC VS ME-CT	11.173842	-32.39999	54.74768	1
Priya G.	WLC VS AC	-10.766455	-46.96431	25.4314	1
Priya G.	WLC VS AE	-17.314523	-54.84578	20.21673	1
Priya G.	WLC VS AE-CT	-2.5479177	-48.61404	43.5182	1
Priya G.	WLC VS CT	-32.133062	-69.88972	5.623593	1
Priya G.	WLC VS D/VRE	-51.62143	-80.1609	-23.08196	1
Priya G.	WLC VS MBE	-24.314311	-59.1346	10.50598	1
Priya G.	WLC VS MBE-CT	-36.548542	-73.58752	0.4904394	1
Priya G.	WLC VS ME	-24.407064	-61.0318	12.21768	1

Priya G.	WLC VS ME-CT	0.23374723	-45.6861	46.15359	1
Jin-Hyuck Park	WLC VS AC	-8.6031337	-39.84013	22.63386	1
Jin-Hyuck Park	WLC VS AE	-11.760724	-43.27602	19.75457	1
Jin-Hyuck Park	WLC VS AE-CT	-0.48810163	-41.68401	40.70781	1
Jin-Hyuck Park	WLC VS CT	-17.926113	-49.75369	13.90146	1
Jin-Hyuck Park	WLC VS D/VRE	-49.934042	-75.90768	-23.9604	1
Jin-Hyuck Park	WLC VS MBE	-18.843913	-49.52253	11.83471	1
Jin-Hyuck Park	WLC VS MBE-CT	-32.73619	-64.34331	-1.129075	1
Jin-Hyuck Park	WLC VS ME	-20.270259	-51.90515	11.36463	1
Jin-Hyuck Park	WLC VS ME-CT	2.3969665	-38.2064	43.00034	1
Rivas-Campo Y.	WLC VS AC	0.58426099	-34.53923	35.70775	1
Rivas-Campo Y.	WLC VS AE	-8.5143832	-46.11875	29.08998	1
Rivas-Campo Y.	WLC VS AE-CT	8.9464794	-37.73508	55.62804	1
Rivas-Campo Y.	WLC VS CT	-18.637992	-54.33186	17.05587	1
Rivas-Campo Y.	WLC VS D/VRE	-48.161396	-79.29317	-17.02962	1
Rivas-Campo Y.	WLC VS MBE	-15.540942	-48.78257	17.70068	1
Rivas-Campo Y.	WLC VS MBE-CT	-26.541151	-63.99041	10.90811	1
Rivas-Campo Y.	WLC VS ME	-15.891686	-53.79333	22.00996	1
Rivas-Campo Y.	WLC VS ME-CT	11.583954	-35.64687	58.81478	1
Eun Hee Lim	WLC VS AC	-9.0657861	-44.87154	26.73997	1
Eun Hee Lim	WLC VS AE	-15.775885	-53.00895	21.45718	1
Eun Hee Lim	WLC VS AE-CT	-0.84039009	-46.66503	44.98425	1
Eun Hee Lim	WLC VS CT	-30.223062	-67.53877	7.092647	1
Eun Hee Lim	WLC VS D/VRE	-51.065839	-79.68046	-22.45122	1
Eun Hee Lim	WLC VS MBE	-22.773347	-57.20684	11.66015	1
Eun Hee Lim	WLC VS MBE-CT	-34.930771	-71.67194	1.810405	1
Eun Hee Lim	WLC VS ME	-22.791137	-59.08503	13.50275	1
Eun Hee Lim	WLC VS ME-CT	1.9343482	-43.77423	47.64293	1
Hong Yu	WLC VS AC	-0.81413535	-34.23396	32.60569	1
Hong Yu	WLC VS AE	-8.3951327	-43.76272	26.97245	1
Hong Yu	WLC VS AE-CT	6.0757598	-40.75043	52.90195	1
Hong Yu	WLC VS CT	-20.835528	-55.42423	13.75317	1
Hong Yu	WLC VS D/VRE	-48.350636	-77.43765	-19.26362	1
Hong Yu	WLC VS MBE	-15.402841	-47.49313	16.68745	1
Hong Yu	WLC VS MBE-CT	-27.137391	-62.11943	7.844647	1
Hong Yu	WLC VS ME	-14.986314	-49.28903	19.3164	1
Hong Yu	WLC VS ME-CT	10.185651	-34.30264	54.67394	1

Supplementary Table 90. Sensitivity analysis for the DST outcome in older adults with mild cognitive impairment.

dropped_id	comparison	eff	lci	uci	connected
Soon-Gook Hong	WLC VS AC	0.29755531	-0.3795194	0.97463	1
Soon-Gook Hong	WLC VS AE	0.03859069	-1.137939	1.21512	1
Soon-Gook Hong	WLC VS AE-CT	0.78950367	0.1854498	1.393558	1
Soon-Gook Hong	WLC VS CT	0.81975295	0.172919	1.466587	1
Soon-Gook Hong	WLC VS MBE	1.2184005	0.3576977	2.079103	1
Soon-Gook Hong	WLC VS MBE-CT	1.1256448	0.2925332	1.958756	1
Soon-Gook Hong	WLC VS ME	1.702079	0.7791737	2.624984	1
Soon-Gook Hong	WLC VS RT	0.60592647	0.116829	1.095024	1
Yi Zhu	WLC VS AC	0.29770136	-0.4473636	1.042766	1
Yi Zhu	WLC VS AE	-0.14085809	-1.745285	1.463569	1
Yi Zhu	WLC VS AE-CT	0.79033655	0.1246939	1.455979	1
Yi Zhu	WLC VS CT	0.81842621	0.1143003	1.522552	1
Yi Zhu	WLC VS MBE	1.2139936	0.2849761	2.143011	1
Yi Zhu	WLC VS MBE-CT	1.1405118	0.217775	2.063249	1
Yi Zhu	WLC VS ME	1.6958751	0.6841325	2.707618	1
Yi Zhu	WLC VS RT	0.4358953	-0.08439	0.9561806	1
Lü	WLC VS AC	0.29755531	-0.3795193	0.9746299	1
Lü	WLC VS AE	0.03859069	-1.137939	1.21512	1
Lü	WLC VS AE-CT	0.78950367	0.1854498	1.393558	1
Lü	WLC VS CT	0.81975295	0.172919	1.466587	1
Lü	WLC VS MBE	1.2184005	0.3576978	2.079103	1
Lü	WLC VS MBE-CT	1.1256448	0.2925333	1.958756	1
Lü	WLC VS ME	1.702079	0.7791739	2.624984	1
Lü	WLC VS RT	0.68608902	0.1941331	1.178045	1
Parial	WLC VS AC	0.24347672	-0.5825997	1.069553	1
Parial	WLC VS AE	-0.01850669	-1.307431	1.270417	1
Parial	WLC VS AE-CT	0.81862199	0.1086394	1.528605	1
Parial	WLC VS CT	0.78078994	0.0244574	1.537122	1
Parial	WLC VS MBE	1.1575911	0.1550926	2.160089	1
Parial	WLC VS MBE-CT	1.0924579	0.0941839	2.090732	1
Parial	WLC VS ME	1.6387669	0.5473667	2.730167	1
Parial	WLC VS RT	0.42211698	-0.1074238	0.9516578	1
Ming Qi	WLC VS AC	0.29771478	-0.4424169	1.037847	1
Ming Qi	WLC VS AE	0.18890526	-1.166377	1.544187	1
Ming Qi	WLC VS AE-CT	0.79027537	0.1291652	1.451385	1

Ming Qi	WLC VS CT	0.8185113	0.1186238	1.518399	1
Ming Qi	WLC VS MBE	1.2143441	0.2903072	2.138381	1
Ming Qi	WLC VS MBE-CT	1.1395258	0.2227777	2.056274	1
Ming Qi	WLC VS ME	1.696343	0.6910397	2.701646	1
Ming Qi	WLC VS RT	0.43815387	-0.0803678	0.9566755	1
Donnezan	disconnected				0
Somporn Sungkarat	WLC VS AC	0.29774358	-0.4343186	1.029806	1
Somporn Sungkarat	WLC VS AE	0.03714136	-1.183261	1.257544	1
Somporn Sungkarat	WLC VS AE-CT	0.79018325	0.1364711	1.443895	1
Somporn Sungkarat	WLC VS CT	0.81866471	0.1256846	1.511645	1
Somporn Sungkarat	WLC VS MBE	1.3455729	0.38942	2.301726	1
Somporn Sungkarat	WLC VS MBE-CT	1.1607937	0.2500763	2.071511	1
Somporn Sungkarat	WLC VS ME	1.751385	0.750613	2.752157	1
Somporn Sungkarat	WLC VS RT	0.44192009	-0.0748631	0.9587033	1
Fuzhong Li	WLC VS AC	0.29743548	-0.4904394	1.08531	1
Fuzhong Li	WLC VS AE	0.03510302	-1.2318	1.302006	1
Fuzhong Li	WLC VS AE-CT	0.79084677	0.0855877	1.496106	1
Fuzhong Li	WLC VS CT	0.81770741	0.0763761	1.559039	1
Fuzhong Li	WLC VS MBE	1.2399307	0.1912527	2.288609	1
Fuzhong Li	WLC VS MBE-CT	0.9974209	-0.0466291	2.041471	1
Fuzhong Li	WLC VS ME	1.7040466	0.6226959	2.785397	1
Fuzhong Li	WLC VS RT	0.41762228	-0.121327	0.9565715	1
Angus P. Yu	WLC VS AC	0.29786268	-0.397958	0.9936833	1
Angus P. Yu	WLC VS AE	0.0383514	-1.152904	1.229607	1
Angus P. Yu	WLC VS AE-CT	0.78983483	0.1690362	1.410633	1
Angus P. Yu	WLC VS CT	0.81950758	0.1571147	1.4819	1
Angus P. Yu	WLC VS MBE	0.9502511	-0.0162911	1.916793	1
Angus P. Yu	WLC VS MBE-CT	1.0859038	0.2224053	1.949402	1
Angus P. Yu	WLC VS ME	0.45996027	-0.0547814	0.9747019	1
Somporn Sungkarat	WLC VS AC	0.29769595	-0.4488447	1.044237	1
Somporn Sungkarat	WLC VS AE	0.03664976	-1.195634	1.268933	1
Somporn Sungkarat	WLC VS AE-CT	0.79035444	0.1233536	1.457355	1
Somporn Sungkarat	WLC VS CT	0.81840038	0.1130041	1.523797	1
Somporn Sungkarat	WLC VS MBE	1.2627302	0.3074512	2.218009	1
Somporn Sungkarat	WLC VS MBE-CT	1.1495391	0.223287	2.075791	1
Somporn Sungkarat	WLC VS ME	1.7161146	0.6987355	2.733494	1
Somporn Sungkarat	WLC VS RT	0.43522521	-0.0856556	0.956106	1
Scherder	WLC VS AC	0.29771004	-0.4446223	1.040042	1
Scherder	WLC VS AE	0.000308	-1.332972	1.333588	1

Scherder	WLC VS AE-CT	0.79030316	0.1271716	1.453435	1
Scherder	WLC VS CT	0.81847369	0.1166968	1.520251	1
Scherder	WLC VS MBE	1.214189	0.2879315	2.140446	1
Scherder	WLC VS MBE-CT	1.1399685	0.2205519	2.059385	1
Scherder	WLC VS ME	1.6961352	0.6879605	2.70431	1
Scherder	WLC VS RT	0.43714172	-0.0821316	0.9564151	1
Goumopoulos	WLC VS AC	0.28978207	-0.5808482	1.160412	1
Goumopoulos	WLC VS AE	0.02575473	-1.306244	1.357753	1
Goumopoulos	WLC VS AE-CT	0.78942028	0.0311578	1.547683	1
Goumopoulos	WLC VS CT	0.81972398	0.026384	1.613064	1
Goumopoulos	WLC VS MBE	1.1994483	0.1480503	2.250846	1
Goumopoulos	WLC VS MBE-CT	1.1499444	0.0877051	2.212184	1
Goumopoulos	WLC VS ME	1.6794258	0.5193245	2.839527	1
Goumopoulos	WLC VS RT	0.39780969	-0.1520273	0.9476467	1
Jin-Hyuck Park2024	WLC VS AC	0.3052924	-0.6117505	1.222335	1
Jin-Hyuck Park2024	WLC VS AE	0.04102345	-1.323537	1.405584	1
Jin-Hyuck Park2024	WLC VS AE-CT	0.79411987	0.0247018	1.563538	1
Jin-Hyuck Park2024	WLC VS CT	0.81367111	0.0073006	1.620042	1
Jin-Hyuck Park2024	WLC VS MBE	1.2144408	0.1226468	2.306235	1
Jin-Hyuck Park2024	WLC VS MBE-CT	1.1666491	0.0635466	2.269752	1
Jin-Hyuck Park2024	WLC VS ME	1.6942973	0.4946011	2.893993	1
Jin-Hyuck Park2024	WLC VS RT	0.39525965	-0.1576251	0.9481444	1
Kitsana Krootnark	WLC VS AC	0.29755531	-0.3795194	0.97463	1
Kitsana Krootnark	WLC VS AE	0.03859069	-1.137939	1.21512	1
Kitsana Krootnark	WLC VS AE-CT	0.78950367	0.1854498	1.393558	1
Kitsana Krootnark	WLC VS CT	0.81975295	0.172919	1.466587	1
Kitsana Krootnark	WLC VS MBE	1.2184005	0.3576978	2.079103	1
Kitsana Krootnark	WLC VS MBE-CT	1.1256448	0.2925332	1.958756	1
Kitsana Krootnark	WLC VS ME	1.702079	0.7791738	2.624984	1
Kitsana Krootnark	WLC VS RT	-0.1223559	-0.7590054	0.5142936	1
Meixiang Fan	WLC VS AC	0.29772584	-0.4474039	1.042856	1
Meixiang Fan	WLC VS AE	0.03672358	-1.194402	1.267849	1
Meixiang Fan	WLC VS AE-CT	0.79035144	0.124655	1.456048	1
Meixiang Fan	WLC VS CT	0.81844579	0.1142619	1.52263	1
Meixiang Fan	WLC VS MBE	1.2784572	0.334427	2.222487	1
Meixiang Fan	WLC VS MBE-CT	1.4781023	0.2447972	2.711407	1
Meixiang Fan	WLC VS ME	1.7227729	0.7083035	2.737242	1
Meixiang Fan	WLC VS RT	0.4358724	-0.0883485	0.9600933	1
Hong Yu	WLC VS AC	0.34392455	-0.443	1.130849	1

Hong Yu	WLC VS AE	0.08245063	-1.177274	1.342175	1
Hong Yu	WLC VS AE-CT	0.76651966	0.0788845	1.454155	1
Hong Yu	WLC VS CT	0.85042078	0.1196907	1.581151	1
Hong Yu	WLC VS MBE	1.2591697	0.2943937	2.223946	1
Hong Yu	WLC VS MBE-CT	1.1897493	0.2236402	2.155858	1
Hong Yu	WLC VS ME	1.7407001	0.6907598	2.79064	1
Hong Yu	WLC VS RT	0.42912479	-0.0952309	0.9534804	1

Supplementary Table 91. Sensitivity analysis for the DRA outcome in older adults with mild cognitive impairment.

dropped_id	comparison	eff	lci	uci	connected
Chang J.	WLC VS AC	-0.27107795	-0.586413	0.0442571	1
Chang J.	WLC VS AE	-0.58150989	-0.8422322	-0.3207876	1
Chang J.	WLC VS AE-CT	-0.5733304	-1.172253	0.0255919	1
Chang J.	WLC VS CT	-0.33625934	-0.7690276	0.0965089	1
Chang J.	WLC VS D/VRE	-0.37336934	-0.8230697	0.076331	1
Chang J.	WLC VS MBE	-0.30951567	-0.7674169	0.1483855	1
Chang J.	WLC VS ME	-0.60100124	-1.010435	-0.1915676	1
Chang J.	WLC VS ME-CT	-0.43865655	-0.8074114	-0.0699017	1
Chang J.	WLC VS RT	1.032189	0.2004214	1.863957	1
Chang J.	WLC VS RT-CT	-0.30940687	-1.072422	0.4536085	1
Lina Wang	WLC VS AC	-0.26480356	-0.5749446	0.0453375	1
Lina Wang	WLC VS AE	-0.49202169	-0.7076294	-0.276414	1
Lina Wang	WLC VS AE-CT	-0.56705622	-1.16326	0.0291479	1
Lina Wang	WLC VS CT	-0.32403339	-0.7511013	0.1030345	1
Lina Wang	WLC VS D/VRE	-0.36570244	-0.8128921	0.0814873	1
Lina Wang	WLC VS MBE	-0.28919333	-0.7381173	0.1597306	1
Lina Wang	WLC VS ME	-0.57300608	-0.9766722	-0.1693399	1
Lina Wang	WLC VS ME-CT	-0.43058773	-0.7942856	-0.0668899	1
Lina Wang	WLC VS RT	1.0500109	0.2215033	1.878518	1
Lina Wang	WLC VS RT-CT	-0.29140402	-1.050858	0.4680499	1
Dan Song.	WLC VS AC	-0.17330464	-0.5122193	0.16561	1
Dan Song.	WLC VS AE	-0.50281346	-0.7287521	-0.2768748	1
Dan Song.	WLC VS AE-CT	-0.47555977	-1.099007	0.1478874	1
Dan Song.	WLC VS CT	-0.25408526	-0.7040313	0.1958607	1
Dan Song.	WLC VS D/VRE	-0.31256853	-0.7723986	0.1472615	1
Dan Song.	WLC VS MBE	-0.22234755	-0.6911954	0.2465003	1

Dan Song.	WLC VS ME	-0.5562655	-0.9689192	-0.1436117	1
Dan Song.	WLC VS ME-CT	-0.34380773	-0.7293153	0.0416998	1
Dan Song.	WLC VS RT	1.1024763	0.2622805	1.942672	1
Dan Song.	WLC VS RT-CT	-0.23954999	-1.011675	0.5325752	1
Yi Zhu	WLC VS AC	-0.16651597	-0.4635272	0.1304953	1
Yi Zhu	WLC VS AE	-0.50174197	-0.7171687	-0.2863152	1
Yi Zhu	WLC VS AE-CT	-0.46877195	-1.058253	0.1207093	1
Yi Zhu	WLC VS CT	-0.2472609	-0.668596	0.1740742	1
Yi Zhu	WLC VS D/VRE	-0.30908389	-0.753306	0.1351383	1
Yi Zhu	WLC VS MBE	-0.21638298	-0.6604078	0.2276419	1
Yi Zhu	WLC VS ME	-0.55553029	-0.9588842	-0.1521764	1
Yi Zhu	WLC VS ME-CT	-0.34197481	-0.6966527	0.0127031	1
Yi Zhu	WLC VS RT	1.1037997	0.2767342	1.930865	1
Yi Zhu	WLC VS RT-CT	-0.23826065	-0.9961786	0.5196573	1
Lazarou	WLC VS AC	-0.19923174	-0.5515814	0.1531179	1
Lazarou	WLC VS AE	-0.49794108	-0.7912678	-0.2046144	1
Lazarou	WLC VS AE-CT	-0.50148528	-1.143872	0.1409017	1
Lazarou	WLC VS CT	-0.27550472	-0.7394058	0.1883964	1
Lazarou	WLC VS D/VRE	-0.32670199	-0.7982695	0.1448654	1
Lazarou	WLC VS MBE	-0.24174399	-0.7305954	0.2471074	1
Lazarou	WLC VS ME	-0.55915678	-0.9884719	-0.1298417	1
Lazarou	WLC VS ME-CT	-0.36226138	-0.7742194	0.0496966	1
Lazarou	WLC VS RT	1.0916212	0.2355526	1.94769	1
Lazarou	WLC VS RT-CT	-0.2502468	-1.039581	0.5390872	1
Marta Bisbe	WLC VS AC	-0.19999911	-0.493244	0.0932458	1
Marta Bisbe	WLC VS AE	-0.50724165	-0.7289441	-0.2855392	1
Marta Bisbe	WLC VS AE-CT	-0.50225392	-1.090393	0.085885	1
Marta Bisbe	WLC VS CT	-0.27380855	-0.6971861	0.1495689	1
Marta Bisbe	WLC VS D/VRE	-0.32762375	-0.7703862	0.1151387	1
Marta Bisbe	WLC VS MBE	-0.24254576	-0.6876689	0.2025774	1
Marta Bisbe	WLC VS ME	-0.51191024	-0.9818509	-0.0419695	1
Marta Bisbe	WLC VS ME-CT	-0.36801601	-0.7170081	-0.0190239	1
Marta Bisbe	WLC VS RT	1.1118565	0.2769253	1.946788	1
Marta Bisbe	WLC VS RT-CT	-0.22957086	-0.9964331	0.5372914	1
Wang Shuo	WLC VS AC	-0.08462928	-0.3835555	0.214297	1
Wang Shuo	WLC VS AE	-0.3782657	-0.614907	-0.1416244	1
Wang Shuo	WLC VS AE-CT	-0.38688802	-0.9773364	0.2035604	1
Wang Shuo	WLC VS CT	-0.17260362	-0.5963049	0.2510976	1
Wang Shuo	WLC VS D/VRE	-0.25599557	-0.7012326	0.1892415	1

Wang Shuo	WLC VS MBE	-0.13283506	-0.5807443	0.3150741	1
Wang Shuo	WLC VS ME	-0.50172156	-0.9076502	-0.0957929	1
Wang Shuo	WLC VS ME-CT	-0.26462559	-0.6212749	0.0920237	1
Wang Shuo	WLC VS RT	1.1696226	0.3411535	1.998092	1
Wang Shuo	WLC VS RT-CT	-0.17265096	-0.9321046	0.5868027	1
Parial	WLC VS AC	-0.19529487	-0.4803033	0.0897136	1
Parial	WLC VS AE	-0.49889603	-0.714164	-0.283628	1
Parial	WLC VS AE-CT	-0.26974024	-0.6859867	0.1465062	1
Parial	WLC VS CT	-0.32566194	-0.7672672	0.1159433	1
Parial	WLC VS D/VRE	-0.23770215	-0.677389	0.2019847	1
Parial	WLC VS MBE	-0.56064723	-0.9637273	-0.1575672	1
Parial	WLC VS ME	-0.36792061	-0.7145011	-0.02134	1
Parial	WLC VS ME-CT	1.0880503	0.2622507	1.91385	1
Parial	WLC VS RT	-0.25382101	-1.010391	0.5027484	1
Harris A. Eyre	WLC VS AC	-0.19540709	-0.4878464	0.0970322	1
Harris A. Eyre	WLC VS AE	-0.49822339	-0.7159582	-0.2804886	1
Harris A. Eyre	WLC VS AE-CT	-0.497662	-1.086571	0.0912467	1
Harris A. Eyre	WLC VS CT	-0.28602091	-0.7313207	0.1592789	1
Harris A. Eyre	WLC VS D/VRE	-0.33344204	-0.7819949	0.1151108	1
Harris A. Eyre	WLC VS MBE	-0.21676727	-0.7010716	0.267537	1
Harris A. Eyre	WLC VS ME	-0.56047553	-0.9648053	-0.1561457	1
Harris A. Eyre	WLC VS ME-CT	-0.36798586	-0.7181961	-0.0177756	1
Harris A. Eyre	WLC VS RT	1.0880978	0.260657	1.915539	1
Harris A. Eyre	WLC VS RT-CT	-0.25376857	-1.012123	0.504586	1
Langoni	WLC VS AC	-0.201328	-0.5061816	0.1035256	1
Langoni	WLC VS AE	-0.50313076	-0.7305046	-0.2757569	1
Langoni	WLC VS AE-CT	-0.50358262	-1.100319	0.0931541	1
Langoni	WLC VS CT	-0.27541691	-0.7062587	0.1554249	1
Langoni	WLC VS D/VRE	-0.32973541	-0.7783818	0.1189109	1
Langoni	WLC VS MBE	-0.24328206	-0.6956246	0.2090605	1
Langoni	WLC VS ME	-0.5881079	-1.178834	0.0026186	1
Langoni	WLC VS ME-CT	-0.37424597	-0.7422506	-0.0062414	1
Langoni	WLC VS RT	1.0713725	0.2014244	1.941321	1
Langoni	WLC VS RT-CT	-0.27068423	-1.076165	0.5347965	1
Geoffrey Tremont	WLC VS AC	-0.18963601	-0.4755865	0.0963145	1
Geoffrey Tremont	WLC VS AE	-0.49786651	-0.7131757	-0.2825574	1
Geoffrey Tremont	WLC VS AE-CT	-0.4918912	-1.075878	0.0920956	1
Geoffrey Tremont	WLC VS CT	-0.29347013	-0.7209708	0.1340306	1
Geoffrey Tremont	WLC VS D/VRE	-0.33615641	-0.7798595	0.1075467	1

Geoffrey Tremont	WLC VS MBE	-0.29500682	-0.7936786	0.2036649	1
Geoffrey Tremont	WLC VS ME	-0.55943333	-0.9625441	-0.1563226	1
Geoffrey Tremont	WLC VS ME-CT	-0.3639382	-0.7109038	-0.0169726	1
Geoffrey Tremont	WLC VS RT	1.0906775	0.2648075	1.916547	1
Geoffrey Tremont	WLC VS RT-CT	-0.25121902	-1.007864	0.5054258	1
Hongyu Yang	WLC VS AC	-0.19596071	-0.4809752	0.0890538	1
Hongyu Yang	WLC VS AE	-0.50030415	-0.7156128	-0.2849955	1
Hongyu Yang	WLC VS AE-CT	-0.49821569	-1.081745	0.0853134	1
Hongyu Yang	WLC VS CT	-0.24402306	-0.6672429	0.1791968	1
Hongyu Yang	WLC VS D/VRE	-0.31322768	-0.7563798	0.1299244	1
Hongyu Yang	WLC VS MBE	-0.272949	-0.724959	0.179061	1
Hongyu Yang	WLC VS ME	-0.56095845	-0.9640395	-0.1578774	1
Hongyu Yang	WLC VS ME-CT	-0.36748363	-0.7140662	-0.0209011	1
Hongyu Yang	WLC VS RT	1.0881211	0.2623215	1.913921	1
Hongyu Yang	WLC VS RT-CT	-0.25375707	-1.010327	0.5028123	1
Danny J. Yu	WLC VS AC	-0.15857677	-0.4457691	0.1286156	1
Danny J. Yu	WLC VS AE	-0.50252745	-0.717824	-0.2872309	1
Danny J. Yu	WLC VS AE-CT	-0.46083301	-1.045429	0.1237628	1
Danny J. Yu	WLC VS CT	-0.24105977	-0.658221	0.1761015	1
Danny J. Yu	WLC VS D/VRE	-0.30451047	-0.746585	0.137564	1
Danny J. Yu	WLC VS MBE	-0.21050183	-0.6509678	0.2299641	1
Danny J. Yu	WLC VS ME	-0.55411866	-0.9572477	-0.1509896	1
Danny J. Yu	WLC VS ME-CT	-0.3348166	-0.6828595	0.0132263	1
Danny J. Yu	WLC VS RT	1.1081447	0.2821185	1.934171	1
Danny J. Yu	WLC VS RT-CT	-0.23396773	-0.9907786	0.5228431	1
Jorge Buele	WLC VS AC	-0.21792308	-0.5057284	0.0698823	1
Jorge Buele	WLC VS AE	-0.51034956	-0.7265688	-0.2941303	1
Jorge Buele	WLC VS AE-CT	-0.52017732	-1.105075	0.0647199	1
Jorge Buele	WLC VS CT	-0.24208771	-0.6612002	0.1770247	1
Jorge Buele	WLC VS D/VRE	-0.20496994	-0.6954955	0.2855556	1
Jorge Buele	WLC VS MBE	-0.23247125	-0.6722555	0.207313	1
Jorge Buele	WLC VS ME	-0.57643875	-0.9804858	-0.1723917	1
Jorge Buele	WLC VS ME-CT	-0.42220401	-0.7818439	-0.0625641	1
Jorge Buele	WLC VS RT	1.0526104	0.2244339	1.880787	1
Jorge Buele	WLC VS RT-CT	-0.28891178	-1.048024	0.4702004	1
Carvalho	WLC VS AC	-0.2122695	-0.5158065	0.0912675	1
Carvalho	WLC VS AE	-0.5083708	-0.7310077	-0.2857339	1
Carvalho	WLC VS AE-CT	-0.51452374	-1.110736	0.0816883	1
Carvalho	WLC VS CT	-0.33482765	-0.8211463	0.151491	1

Carvalho	WLC VS D/VRE	-0.24406854	-0.7772994	0.2891623	1
Carvalho	WLC VS MBE	-0.27886808	-0.750232	0.1924959	1
Carvalho	WLC VS ME	-0.56484278	-0.9706985	-0.1589871	1
Carvalho	WLC VS ME-CT	-0.37401198	-0.7288509	-0.019173	1
Carvalho	WLC VS RT	1.0828808	0.2535382	1.912223	1
Carvalho	WLC VS RT-CT	-0.25896782	-1.019386	0.5014505	1
So Young Moon	WLC VS AC	-0.22247595	-0.5319965	0.0870446	1
So Young Moon	WLC VS AE	-0.51040454	-0.7336676	-0.2871415	1
So Young Moon	WLC VS AE-CT	-0.52472978	-1.124987	0.0755275	1
So Young Moon	WLC VS CT	-0.27445197	-0.7033916	0.1544877	1
So Young Moon	WLC VS D/VRE	-0.28876765	-0.7507117	0.1731764	1
So Young Moon	WLC VS MBE	-0.25101037	-0.7022264	0.2002056	1
So Young Moon	WLC VS ME	-0.52396913	-0.9467655	-0.1011727	1
So Young Moon	WLC VS ME-CT	-0.19396546	-0.8499045	0.4619736	1
So Young Moon	WLC VS RT	1.1947833	0.2982788	2.091288	1
So Young Moon	WLC VS RT-CT	-0.1483162	-0.9798695	0.6832371	1
Goumopoulos	WLC VS AC	-0.19855021	-0.4840578	0.0869574	1
Goumopoulos	WLC VS AE	-0.50013302	-0.7154965	-0.2847695	1
Goumopoulos	WLC VS AE-CT	-0.50080511	-1.084575	0.082965	1
Goumopoulos	WLC VS CT	-0.24454935	-0.6807901	0.1916914	1
Goumopoulos	WLC VS D/VRE	-0.31398832	-0.7597185	0.1317419	1
Goumopoulos	WLC VS MBE	-0.22576175	-0.6697818	0.2182583	1
Goumopoulos	WLC VS ME	-0.56143011	-0.9645306	-0.1583296	1
Goumopoulos	WLC VS ME-CT	-0.36975901	-0.7164707	-0.0230474	1
Goumopoulos	WLC VS RT	1.0867287	0.2609006	1.912557	1
Goumopoulos	WLC VS RT-CT	-0.2551331	-1.011733	0.5014669	1
İsmail Uysal	WLC VS AC	-0.18581329	-0.4858181	0.1141916	1
İsmail Uysal	WLC VS AE	-0.49530329	-0.7153606	-0.275246	1
İsmail Uysal	WLC VS AE-CT	-0.48806844	-1.082289	0.1061518	1
İsmail Uysal	WLC VS CT	-0.26144677	-0.6888301	0.1659366	1
İsmail Uysal	WLC VS D/VRE	-0.31725563	-0.7652698	0.1307585	1
İsmail Uysal	WLC VS MBE	-0.22958973	-0.6785779	0.2193985	1
İsmail Uysal	WLC VS ME	-0.58906138	-1.036066	-0.1420566	1
İsmail Uysal	WLC VS ME-CT	-0.34838034	-0.7275556	0.0307949	1
Qiang Zhang	WLC VS AC	-0.19917818	-0.4888647	0.0905083	1
Qiang Zhang	WLC VS AE	-0.50111316	-0.7165946	-0.2856318	1
Qiang Zhang	WLC VS AE-CT	-0.5014282	-1.087254	0.0843978	1
Qiang Zhang	WLC VS CT	-0.2263061	-0.6628787	0.2102665	1
Qiang Zhang	WLC VS D/VRE	-0.30520009	-0.7524778	0.1420776	1

Qiang Zhang	WLC VS MBE	-0.13908039	-0.6495398	0.371379	1
Qiang Zhang	WLC VS ME	-0.5616754	-0.9648545	-0.1584963	1
Qiang Zhang	WLC VS ME-CT	-0.36958873	-0.7195342	-0.0196432	1
Qiang Zhang	WLC VS RT	1.0866964	0.2603867	1.913006	1
Qiang Zhang	WLC VS RT-CT	-0.25516917	-1.012282	0.5019433	1
Petri	WLC VS AC	-0.20165442	-0.4871531	0.0838443	1
Petri	WLC VS AE	-0.5013129	-0.7166747	-0.2859511	1
Petri	WLC VS AE-CT	-0.50390921	-1.087675	0.0798565	1
Petri	WLC VS CT	-0.22054791	-0.6564384	0.2153426	1
Petri	WLC VS D/VRE	-0.30286639	-0.748523	0.1427902	1
Petri	WLC VS MBE	-0.2143862	-0.658329	0.2295566	1
Petri	WLC VS ME	-0.56217692	-0.9652771	-0.1590768	1
Petri	WLC VS ME-CT	-0.37151283	-0.7182221	-0.0248035	1
Petri	WLC VS RT	1.0854678	0.2596402	1.911295	1
Petri	WLC VS RT-CT	-0.25638479	-1.012984	0.5002147	1
Hiroshi Hayashi	WLC VS AC	-0.27469965	-0.5755659	0.0261666	1
Hiroshi Hayashi	WLC VS AE	-0.54220595	-0.7638008	-0.3206111	1
Hiroshi Hayashi	WLC VS AE-CT	-0.57695198	-1.168385	0.0144809	1
Hiroshi Hayashi	WLC VS CT	-0.42842388	-0.8870814	0.0302336	1
Hiroshi Hayashi	WLC VS D/VRE	-0.64129275	-1.225942	-0.0566436	1
Hiroshi Hayashi	WLC VS MBE	-0.3539754	-0.8157617	0.1078109	1
Hiroshi Hayashi	WLC VS ME	-0.59515516	-1.000406	-0.1899043	1
Hiroshi Hayashi	WLC VS ME-CT	-0.46324457	-0.8286319	-0.0978572	1
Hiroshi Hayashi	WLC VS RT	1.0224989	0.1928743	1.852123	1
Hiroshi Hayashi	WLC VS RT-CT	-0.31882077	-1.079494	0.4418521	1
Kang	WLC VS AC	-0.1868419	-0.4725607	0.0988769	1
Kang	WLC VS AE	-0.49568282	-0.7110868	-0.2802789	1
Kang	WLC VS AE-CT	-0.48909719	-1.072971	0.0947762	1
Kang	WLC VS CT	-0.33507794	-0.7795089	0.1093531	1
Kang	WLC VS D/VRE	-0.35593805	-0.8034023	0.0915262	1
Kang	WLC VS MBE	-0.26866811	-0.7145091	0.1771728	1
Kang	WLC VS ME	-0.55861337	-0.9617226	-0.1555042	1
Kang	WLC VS ME-CT	-0.36314413	-0.7099113	-0.016377	1
Kang	WLC VS RT	1.0914841	0.265644	1.917324	1
Kang	WLC VS RT-CT	-0.25041209	-1.007025	0.5062009	1

Supplementary Table 92. Sensitivity analysis for the MFA outcome in older adults with mild cognitive impairment.

dropped_id	comparison	eff	lci	uci	connected
Tomoto T.	WLC VS AC	0.16400261	-0.4673778	0.795383	1
Tomoto T.	WLC VS AE	0.60470004	0.0656499	1.14375	1
Tomoto T.	WLC VS CT	0.09739472	-0.7481522	0.9429416	1
Tomoto T.	WLC VS MBE	-0.13434512	-1.19982	0.9311296	1
Tomoto T.	WLC VS MBE-CT	-0.02505322	-1.124311	1.074204	1
Tomoto T.	WLC VS ME	0.20576284	-0.778381	1.189907	1
Tomoto T.	WLC VS ME-CT	0.6561677	-0.2939374	1.606273	1
Tomoto T.	WLC VS RT	-0.24904773	-1.185444	0.6873489	1
Binu P. Thomas	WLC VS AC	0.30124107	-0.4248119	1.027294	1
Binu P. Thomas	WLC VS AE	0.60450336	-0.0303263	1.239333	1
Binu P. Thomas	WLC VS CT	0.22481999	-0.7254031	1.175043	1
Binu P. Thomas	WLC VS MBE	-0.00692825	-1.205184	1.191327	1
Binu P. Thomas	WLC VS MBE-CT	0.11216775	-1.091776	1.316111	1
Binu P. Thomas	WLC VS ME	0.20555995	-0.8869552	1.298075	1
Binu P. Thomas	WLC VS ME-CT	0.79338897	-0.2761095	1.862887	1
Binu P. Thomas	WLC VS RT	-0.24904773	-1.243782	0.7456861	1
Soon-Gook Hong	WLC VS AC	0.26171126	-0.4178185	0.9412411	1
Soon-Gook Hong	WLC VS AE	0.60453439	0.00396	1.205109	1
Soon-Gook Hong	WLC VS CT	0.18864564	-0.7140221	1.091313	1
Soon-Gook Hong	WLC VS MBE	-0.04309978	-1.185618	1.099418	1
Soon-Gook Hong	WLC VS MBE-CT	0.07264361	-1.08569	1.230977	1
Soon-Gook Hong	WLC VS ME	0.20559332	-0.8474541	1.258641	1
Soon-Gook Hong	WLC VS ME-CT	0.75386516	-0.2640132	1.771744	1
Yi Zhu	WLC VS AC	0.3873249	-0.1547244	0.9293742	1
Yi Zhu	WLC VS AE	0.60468917	0.163819	1.045559	1
Yi Zhu	WLC VS CT	0.33194247	-0.422594	1.086479	1
Yi Zhu	WLC VS MBE	0.10019295	-0.8449579	1.045344	1
Yi Zhu	WLC VS MBE-CT	0.19824921	-0.805366	1.201864	1
Yi Zhu	WLC VS ME	0.20575729	-0.6751605	1.086675	1
Yi Zhu	WLC VS ME-CT	0.87948314	0.0418867	1.71708	1
Yi Zhu	WLC VS RT	-0.24904773	-1.132505	0.6344092	1
Lazarou	disconnected				0
Tarumi	WLC VS AC	0.16338231	-0.4937222	0.8204868	1
Tarumi	WLC VS AE	0.60468011	0.0449179	1.164442	1
Tarumi	WLC VS CT	0.09454869	-0.7753704	0.9644678	1

Tarumi	WLC VS MBE	-0.13719096	-1.23256	0.9581783	1
Tarumi	WLC VS MBE-CT	-0.02567365	-1.150083	1.098736	1
Tarumi	WLC VS ME	0.20574165	-0.8012779	1.212761	1
Tarumi	WLC VS ME-CT	0.65554622	-0.3235512	1.634644	1
Tarumi	WLC VS RT	-0.24904773	-1.197539	0.6994441	1
Marta Bisbe	WLC VS AC	0.26174964	-0.4178257	0.9413249	1
Marta Bisbe	WLC VS AE	0.60457408	0.0039486	1.205199	1
Marta Bisbe	WLC VS CT	0.18868172	-0.7140247	1.091388	1
Marta Bisbe	WLC VS MBE	-0.04306369	-1.185624	1.099497	1
Marta Bisbe	WLC VS MBE-CT	0.07268198	-1.08569	1.231054	1
Marta Bisbe	WLC VS ME	0.75390353	-0.2640188	1.771826	1
Marta Bisbe	WLC VS ME-CT	-0.24904773	-1.222262	0.7241662	1
Ming Qi	WLC VS AC	0.31251856	-0.3978566	1.022894	1
Ming Qi	WLC VS AE	0.6045148	-0.0149946	1.224024	1
Ming Qi	WLC VS CT	0.23757293	-0.6950957	1.170241	1
Ming Qi	WLC VS MBE	0.00582411	-1.170404	1.182052	1
Ming Qi	WLC VS MBE-CT	0.12344443	-1.063025	1.309914	1
Ming Qi	WLC VS ME	0.20557246	-0.8691927	1.280338	1
Ming Qi	WLC VS ME-CT	0.80466694	-0.2451209	1.854455	1
Ming Qi	WLC VS RT	-0.24904773	-1.234053	0.7359576	1
Harris A. Eyre	WLC VS AC	0.26171893	-0.4178828	0.9413207	1
Harris A. Eyre	WLC VS AE	0.60454174	0.0039088	1.205175	1
Harris A. Eyre	WLC VS CT	0.18865735	-0.714139	1.091454	1
Harris A. Eyre	WLC VS MBE	0.07265128	-1.08574	1.231043	1
Harris A. Eyre	WLC VS MBE-CT	0.20560066	-0.8474975	1.258699	1
Harris A. Eyre	WLC VS ME	0.75387284	-0.2640715	1.771817	1
Harris A. Eyre	WLC VS ME-CT	-0.24904773	-1.222266	0.7241709	1
Tsubasa Tomoto	WLC VS AC	0.25355007	-0.521841	1.028941	1
Tsubasa Tomoto	WLC VS AE	0.60448469	-0.0745633	1.283533	1
Tsubasa Tomoto	WLC VS CT	0.17306721	-0.8317458	1.17788	1
Tsubasa Tomoto	WLC VS MBE	-0.05867806	-1.323885	1.206529	1
Tsubasa Tomoto	WLC VS MBE-CT	0.06448127	-1.193202	1.322164	1
Tsubasa Tomoto	WLC VS ME	0.20553807	-0.9389725	1.350049	1
Tsubasa Tomoto	WLC VS ME-CT	0.74569797	-0.3839578	1.875354	1
Tsubasa Tomoto	WLC VS RT	-0.24904773	-1.272638	0.7745425	1
Takao Suzuki	WLC VS AC	0.26177111	-0.4178191	0.9413613	1
Takao Suzuki	WLC VS AE	0.60458215	0.0039565	1.205208	1
Takao Suzuki	WLC VS CT	0.18870316	-0.714013	1.091419	1
Takao Suzuki	WLC VS MBE	-0.04304223	-1.18561	1.099526	1

Takao Suzuki	WLC VS MBE-CT	0.07270345	-1.085678	1.231084	1
Takao Suzuki	WLC VS ME	0.20564107	-0.8474488	1.258731	1
Takao Suzuki	WLC VS ME-CT	-0.24904773	-1.222262	0.7241664	1
Cheung	WLC VS AC	0.26173003	-0.4178642	0.9413243	1
Cheung	WLC VS AE	0.60455017	0.0039234	1.205177	1
Cheung	WLC VS CT	0.18866198	-0.7140616	1.091386	1
Cheung	WLC VS MBE	-0.04308344	-1.185658	1.099491	1
Cheung	WLC VS MBE-CT	0.2056091	-0.847482	1.2587	1
Cheung	WLC VS ME	0.75388394	-0.2640518	1.77182	1
Cheung	WLC VS ME-CT	-0.24904773	-1.222263	0.724167	1
Jingsong Wu	WLC VS AC	0.25968699	-0.4595177	0.9788917	1
Jingsong Wu	WLC VS AE	0.60455025	-0.034211	1.243312	1
Jingsong Wu	WLC VS CT	-0.07965953	-1.337819	1.1785	1
Jingsong Wu	WLC VS MBE	-0.31138707	-1.767677	1.144902	1
Jingsong Wu	WLC VS MBE-CT	0.07061855	-1.131298	1.272535	1
Jingsong Wu	WLC VS ME	0.20560656	-0.8914868	1.3027	1
Jingsong Wu	WLC VS ME-CT	0.75183789	-0.315378	1.819054	1
Jingsong Wu	WLC VS RT	-0.24904773	-1.246301	0.7482058	1
Goumopoulos	WLC VS AC	0.25960535	-0.4596082	0.9788189	1
Goumopoulos	WLC VS AE	0.60448586	-0.0342784	1.24325	1
Goumopoulos	WLC VS CT	0.3241159	-0.7203142	1.368546	1
Goumopoulos	WLC VS MBE	0.09236074	-1.183853	1.368575	1
Goumopoulos	WLC VS MBE-CT	0.07053692	-1.131386	1.27246	1
Goumopoulos	WLC VS ME	0.20554218	-0.8915545	1.302639	1
Goumopoulos	WLC VS ME-CT	0.75175626	-0.3154673	1.81898	1
Goumopoulos	WLC VS RT	-0.24904773	-1.246303	0.7482076	1

Supplementary Table 93. Sensitivity analysis after excluding studies at high risk of bias: SUCRA ranking table for the MoCA outcome in older adults with mild cognitive impairment.

Treatm~t	SUCRA	PrBest	MeanRank
MBE	95.2	67.7	1.4
AE-CT	83.9	24.3	2.5
AE	81.5	7	2.7
CT	50.9	0.1	5.4

ME	47	0	5.8
D/VRE	46.3	0.6	5.8
RT	43	0.1	6.1
AC	29.7	0	7.3
MBE-CT	16.6	0.3	8.5
WLC	5.8	0	9.5

Supplementary Table 94. Sensitivity analysis after excluding studies at high risk of bias: SUCRA ranking table for the MMSE outcome in older adults with mild cognitive impairment.

Treatm~t	SUCRA	PrBest	MeanRank
MBE	82	41.3	2.8
ME	80.8	12.2	2.9
RT-CT	75.5	21	3.5
ME-CT	75.2	12.7	3.5
CT	50.2	3.6	6
D/VRE	49.4	6.2	6.1
MBE-CT	43.7	2.7	6.6
AC	37.2	0	7.3
RT	30.2	0.3	8
AE	24.1	0	8.6
WLC	1.8	0	10.8

Supplementary Table 95. Sensitivity analysis after excluding studies at high risk of bias: SUCRA ranking table for the ADAS-Cog outcome in older adults with mild cognitive impairment.

Treatm~t	SUCRA	PrBest	MeanRank
RT	92.2	79.1	1.6
ME-CT	78.9	10	2.7
CT	74.1	5.8	3.1
AE	56.1	4.2	4.5
ME	55.6	0.6	4.6
AC	53.8	0.3	4.7
MBE	20.4	0	7.4
WLC	17.8	0	7.6
D/VRE	1.1	0	8.9

Supplementary Table 96. Sensitivity analysis after excluding studies at high risk of bias: SUCRA ranking table for the TMT-B outcome in older adults with mild cognitive impairment.

Treatm~t	SUCRA	PrBest	MeanRank
D/VRE	96.7	85.1	1.3
MBE-CT	84.4	10.3	2.4
CT	73.1	3.7	3.4
MBE	59.2	0	4.7
ME	58.1	0.3	4.8
AE	39.1	0.1	6.5
WLC	28.9	0	7.4
AC	24.5	0	7.8
AE-CT	21.3	0.3	8.1

ME-CT	14.6	0.1	8.7

Supplementary Table 97. Sensitivity analysis after excluding studies at high risk of bias: SUCRA ranking table for the DST outcome in older adults with mild cognitive impairment.

Treatm~t	SUCRA	PrBest	MeanRank
ME	92	73.6	1.6
CT	78.2	14.3	2.7
MBE-CT	69.5	6.1	3.4
MBE	62.6	1.2	4
AE-CT	55.8	2.4	4.5
RT	32.4	2.2	6.4
AC	25	0	7
AE	24.7	0.2	7
WLC	10	0	8.2

Supplementary Table 98. Sensitivity analysis after excluding studies at high risk of bias: SUCRA ranking table for the SCWT outcome in older adults with mild cognitive impairment.

Treatm~t	SUCRA	PrBest	MeanRank
AE	90.2	60.5	1.6
AE-CT	70.3	14.3	2.8
D/VRE	57	18.2	3.6
RT	38	4.1	4.7
MBE	33.3	2.4	5
CT	32.1	0.5	5.1
WLC	29	0	5.3

Supplementary Table 99. Sensitivity analysis after excluding studies at high risk of bias: SUCRA ranking table for the DRA outcome in older adults with mild cognitive impairment.

Treatm~t	SUCRA	PrBest	MeanRank
ME	89.6	49.9	2
AE	72.5	6.7	3.8
AE-CT	72.3	22.5	3.8
ME-CT	65.5	2.9	4.4
RT-CT	56.4	12.4	5.4
D/VRE	46.1	3.5	6.4
CT	40.6	0.7	6.9
WLC	38.8	0.8	7.1
AC	35.9	0	7.4
MBE	31.9	0.6	7.8
RT	0.3	0	11

Supplementary Table 100. Sensitivity analysis after excluding studies at high risk of bias: SUCRA ranking table for the MFA outcome in older adults with mild cognitive impairment.

Treatm~t	SUCRA	PrBest	MeanRank
ME-CT	84.9	59.8	1.9
AE	73.1	15.4	2.6
AC	47.5	0.6	4.2
CT	42.7	3	4.4
ME	38.7	9	4.7
MBE-CT	36.4	8	4.8
MBE	26.8	4.2	5.4

Supplementary Table 101. Egger's regression tests for funnel plot asymmetry and small-study effects across MoCA, MMSE, TMT-B, DST, DRA, and MFA networks

Outcome	Prob > z
MoCA	0.6331
MMSE	0.6918
TMT-B	0.2091
DST	0.1617
DRA	0.3809
MFA	0.842

Supplementary Table 102. GRADE certainty of evidence for the MoCA outcome in older adults with mild cognitive impairment.

Comparison	Number of studies	Within-study bias	Reporting bias	Indirectness	Imprecision	Heterogeneity	Incoherence	Confidence rating	Reason (s) for downgrading
AC:AE	6	No concerns	Low risk	No concerns	No concerns	Some concerns	No concerns	Moderate	["Heterogeneity"]
AC:AE-CT	2	No concerns	Low risk	No concerns	No concerns	Some concerns	No concerns	Moderate	["Heterogeneity"]
AC:CT	4	Some concerns	Low risk	No concerns	No concerns	Some concerns	No concerns	Low	["Within-study bias","Heterogeneity"]
AC:MBE	4	No concerns	Low risk	No concerns	No concerns	No concerns	No concerns	High	[]
AC:MBE-CT	1	No concerns	Low risk	No concerns	Some concerns	Some concerns	No concerns	Low	["Imprecision","Heterogeneity"]
AC:ME	3	Some concerns	Low risk	No concerns	No concerns	Some concerns	Some concerns	Very low	["Within-study bias","Heterogeneity","Incoherence"]
AC:RT	1	Some concerns	Low risk	No concerns	No concerns	Major concerns	No concerns	Very low	["Within-study bias","Heterogeneity"]
AE:AE-CT	1	No concerns	Low risk	No concerns	No concerns	Major concerns	No concerns	Low	["Heterogeneity"]
AE:MBE	1	No concerns	Low risk	No concerns	No concerns	Some concerns	No concerns	Moderate	["Heterogeneity"]
AE:RT	1	Some concerns	Low risk	No concerns	No concerns	Some concerns	No concerns	Low	["Within-study bias","Heterogeneity"]
AE:WLC	5	Major concerns	Low risk	No concerns	No concerns	No concerns	No concerns	Low	["Within-study bias"]
AE-CT:WLC	1	No concerns	Low risk	No concerns	No concerns	No concerns	No concerns	High	[]
CT:D/VRE	1	Some concerns	Low risk	No concerns	No concerns	Major concerns	No concerns	Very low	["Within-study bias","Heterogeneity"]
CT:RT	1	Some concerns	Low risk	No concerns	No concerns	Major concerns	No concerns	Very low	["Within-study bias","Heterogeneity"]
CT:WLC	2	Some concerns	Low risk	No concerns	No concerns	Some concerns	No concerns	Low	["Within-study bias","Heterogeneity"]
D/VRE:ME	1	Some concerns	Low risk	No concerns	No concerns	Major concerns	Some concerns	Very low	["Within-study bias","Heterogeneity","Incoherence"]
D/VRE:ME-CT	1	Major concerns	Low risk	No concerns	Some concerns	Some concerns	Major concerns	Very low	["Within-study bias","Imprecision","Heterogeneity","Incoherence"]
D/VRE:WLC	1	Some concerns	Low risk	No concerns	No concerns	Some concerns	No concerns	Low	["Within-study bias","Heterogeneity"]

Comparison	Number of studies	Within-study bias	Reporting bias	Indirectness	Imprecision	Heterogeneity	Incoherence	Confidence rating	Reason (s) for downgrading
MBE:WLC	1	Some concerns	Low risk	No concerns	No concerns	No concerns	No concerns	Moderate	["Within-study bias"]
ME:WLC	1	Some concerns	Low risk	No concerns	No concerns	Some concerns	No concerns	Low	["Within-study bias","Heterogeneity"]
RT:WLC	3	Some concerns	Low risk	No concerns	No concerns	Some concerns	Some concerns	Very low	["Within-study bias","Heterogeneity","Incoherence"]
AC:D/VRE	0	Some concerns	Low risk	No concerns	No concerns	Some concerns	No concerns	Low	["Within-study bias","Heterogeneity"]
AC:ME-CT	0	Some concerns	Low risk	No concerns	Some concerns	No concerns	Some concerns	Low	["Within-study bias","Imprecision","Incoherence"]
AC:WLC	0	Some concerns	Low risk	No concerns	No concerns	Some concerns	Major concerns	Very low	["Within-study bias","Heterogeneity","Incoherence"]
AE:CT	0	Some concerns	Low risk	No concerns	No concerns	Some concerns	No concerns	Low	["Within-study bias","Heterogeneity"]
AE:D/VRE	0	Some concerns	Low risk	No concerns	No concerns	Some concerns	No concerns	Low	["Within-study bias","Heterogeneity"]
AE:MBE-CT	0	No concerns	Low risk	No concerns	No concerns	Some concerns	No concerns	Moderate	["Heterogeneity"]
AE:ME	0	Some concerns	Low risk	No concerns	No concerns	Some concerns	Some concerns	Very low	["Within-study bias","Heterogeneity","Incoherence"]
AE:ME-CT	0	Some concerns	Low risk	No concerns	Some concerns	No concerns	No concerns	Low	["Within-study bias","Imprecision"]
AE-CT:CT	0	No concerns	Low risk	No concerns	No concerns	Some concerns	Major concerns	Very low	["Heterogeneity","Incoherence"]
AE-CT:D/VRE	0	Some concerns	Low risk	No concerns	Some concerns	No concerns	No concerns	Low	["Within-study bias","Imprecision"]
AE-CT:MBE	0	No concerns	Low risk	No concerns	No concerns	Major concerns	Major concerns	Very low	["Heterogeneity","Incoherence"]
AE-CT:MBE-CT	0	No concerns	Low risk	No concerns	No concerns	Some concerns	Some concerns	Low	["Heterogeneity","Incoherence"]
AE-CT:ME	0	Some concerns	Low risk	No concerns	No concerns	Some concerns	No concerns	Low	["Within-study bias","Heterogeneity"]
AE-CT:ME-CT	0	Some concerns	Low risk	No concerns	No concerns	Some concerns	Some concerns	Very low	["Within-study bias","Heterogeneity","Incoherence"]
AE-CT:RT	0	Some concerns	Low risk	No concerns	No concerns	Some concerns	Major concerns	Very low	["Within-study bias","Heterogeneity","Incoherence"]
CT:MBE	0	No concerns	Low risk	No concerns	No concerns	Some concerns	No concerns	Moderate	["Heterogeneity"]
CT:MBE-CT	0	No concerns	Low risk	No concerns	No concerns	Some concerns	No concerns	Moderate	["Heterogeneity"]
CT:ME	0	Some concerns	Low risk	No concerns	No concerns	Major concerns	Some concerns	Very low	["Within-study bias","Heterogeneity","Incoherence"]
CT:ME-CT	0	Some concerns	Low risk	No concerns	Some concerns	Some concerns	No concerns	Very low	["Within-study bias","Imprecision","Heterogeneity"]
D/VRE:MBE	0	Some concerns	Low risk	No concerns	No concerns	Some concerns	Major concerns	Very low	["Within-study bias","Heterogeneity","Incoherence"]

Comparison	Number of studies	Within-study bias	Reporting bias	Indirectness	Imprecision	Heterogeneity	Incoherence	Confidence rating	Reason (s) for downgrading
D/VRE:MBE-CT	0	Some concerns	Low risk	No concerns	No concerns	Some concerns	No concerns	Low	["Within-study bias","Heterogeneity"]
D/VRE:RT	0	Some concerns	Low risk	No concerns	No concerns	Major concerns	Some concerns	Very low	["Within-study bias","Heterogeneity","Incoherence"]
MBE:MBE-CT	0	No concerns	Low risk	No concerns	No concerns	No concerns	No concerns	High	[]
MBE:ME	0	Some concerns	Low risk	No concerns	No concerns	Some concerns	No concerns	Low	["Within-study bias","Heterogeneity"]
MBE:ME-CT	0	Some concerns	Low risk	No concerns	No concerns	No concerns	No concerns	Moderate	["Within-study bias"]
MBE:RT	0	Some concerns	Low risk	No concerns	No concerns	Some concerns	Some concerns	Very low	["Within-study bias","Heterogeneity","Incoherence"]
MBE-CT:ME	0	Some concerns	Low risk	No concerns	No concerns	Major concerns	Some concerns	Very low	["Within-study bias","Heterogeneity","Incoherence"]
MBE-CT:ME-CT	0	Some concerns	Low risk	No concerns	Some concerns	Some concerns	Some concerns	Very low	["Within-study bias","Imprecision","Heterogeneity","Incoherence"]
MBE-CT:RT	0	Some concerns	Low risk	No concerns	No concerns	Major concerns	Major concerns	Very low	["Within-study bias","Heterogeneity","Incoherence"]
MBE-CT:WLC	0	No concerns	Low risk	No concerns	No concerns	Major concerns	Major concerns	Very low	["Heterogeneity","Incoherence"]
ME:ME-CT	0	Some concerns	Low risk	No concerns	Some concerns	No concerns	No concerns	Low	["Within-study bias","Imprecision"]
ME:RT	0	Some concerns	Low risk	No concerns	No concerns	Major concerns	No concerns	Very low	["Within-study bias","Heterogeneity"]
ME-CT:RT	0	Some concerns	Low risk	No concerns	No concerns	No concerns	No concerns	Moderate	["Within-study bias"]
ME-CT:WLC	0	Some concerns	Low risk	No concerns	Major concerns	No concerns	Some concerns	Very low	["Within-study bias","Imprecision","Incoherence"]

Supplementary Table 103. GRADE certainty of evidence for the MMSE outcome in older adults with mild cognitive impairment.

Comparison	Number of studies	Within-study bias	Reporting bias	Indirectness	Imprecision	Heterogeneity	Incoherence	Confidence rating	Reason (s) for downgrading
AC:AE	3	Some concerns	Low risk	No concerns	No concerns	Major concerns	Some concerns	Very low	["Within-study bias","Heterogeneity","Incoherence"]

Comparison	Number of studies	Within-study bias	Reporting bias	Indirectness	Imprecision	Heterogeneity	Incoherence	Confidence rating	Reason<br><br>(s) for downgrading
AC:CT	2	Major concerns	Low risk	No concerns	No concerns	Major concerns	No concerns	Very low	["Within-study bias","Heterogeneity"]
AC:MBE	2	Some concerns	Low risk	No concerns	No concerns	Some concerns	Major concerns	Very low	["Within-study bias","Heterogeneity","Incoherence"]
AC:MBE-CT	1	No concerns	Low risk	No concerns	No concerns	Major concerns	Major concerns	Very low	["Heterogeneity","Incoherence"]
AC:ME	3	No concerns	Low risk	No concerns	No concerns	No concerns	No concerns	High	[]
AC:ME-CT	1	No concerns	Low risk	No concerns	No concerns	Major concerns	No concerns	Low	["Heterogeneity"]
AE:WLC	5	Some concerns	Low risk	No concerns	No concerns	No concerns	No concerns	Moderate	["Within-study bias"]
AE-CT:WLC	1	Major concerns	Low risk	No concerns	No concerns	Major concerns	Major concerns	Very low	["Within-study bias","Heterogeneity","Incoherence"]
CT:WLC	2	Some concerns	Low risk	No concerns	No concerns	Some concerns	No concerns	Low	["Within-study bias","Heterogeneity"]
D/VRE:WLC	1	No concerns	Low risk	No concerns	No concerns	No concerns	No concerns	High	[]
ME:ME-CT	1	Some concerns	Low risk	No concerns	No concerns	Major concerns	No concerns	Very low	["Within-study bias","Heterogeneity"]
ME:RT	1	Some concerns	Low risk	No concerns	No concerns	Some concerns	No concerns	Low	["Within-study bias","Heterogeneity"]
ME:RT-CT	1	Some concerns	Low risk	No concerns	No concerns	Major concerns	Some concerns	Very low	["Within-study bias","Heterogeneity","Incoherence"]
ME:WLC	2	Some concerns	Low risk	No concerns	No concerns	No concerns	No concerns	Moderate	["Within-study bias"]
ME-CT:RT	1	Some concerns	Low risk	No concerns	No concerns	Major concerns	Some concerns	Very low	["Within-study bias","Heterogeneity","Incoherence"]
ME-CT:RT-CT	1	Some concerns	Low risk	No concerns	No concerns	Some concerns	Some concerns	Very low	["Within-study bias","Heterogeneity","Incoherence"]
RT:RT-CT	1	Some concerns	Low risk	No concerns	No concerns	Some concerns	Major concerns	Very low	["Within-study bias","Heterogeneity","Incoherence"]
AC:AE-CT	0	Major concerns	Low risk	No concerns	No concerns	Major concerns	Major concerns	Very low	["Within-study bias","Heterogeneity","Incoherence"]
AC:D/VRE	0	No concerns	Low risk	No concerns	No concerns	Some concerns	No concerns	Moderate	["Heterogeneity"]
AC:RT	0	Some concerns	Low risk	No concerns	No concerns	Some concerns	Some concerns	Very low	["Within-study bias","Heterogeneity","Incoherence"]
AC:RT-CT	0	Some concerns	Low risk	No concerns	No concerns	Major concerns	No concerns	Very low	["Within-study bias","Heterogeneity"]

Comparison	Number of studies	Within-study bias	Reporting bias	Indirectness	Imprecision	Heterogeneity	Incoherence	Confidence rating	Reason (s) for downgrading
AC:WLC	0	Some concerns	Low risk	No concerns	No concerns	Some concerns	Some concerns	Very low	["Within-study bias","Heterogeneity","Incoherence"]
AE:AE-CT	0	Major concerns	Low risk	No concerns	No concerns	Some concerns	No concerns	Very low	["Within-study bias","Heterogeneity"]
AE:CT	0	Major concerns	Low risk	No concerns	No concerns	Some concerns	Some concerns	Very low	["Within-study bias","Heterogeneity","Incoherence"]
AE:D/VRE	0	No concerns	Low risk	No concerns	No concerns	No concerns	Some concerns	Moderate	["Incoherence"]
AE:MBE	0	Some concerns	Low risk	No concerns	No concerns	Some concerns	No concerns	Low	["Within-study bias","Heterogeneity"]
AE:MBE-CT	0	No concerns	Low risk	No concerns	No concerns	Major concerns	No concerns	Low	["Heterogeneity"]
AE:ME	0	Some concerns	Low risk	No concerns	No concerns	Some concerns	No concerns	Low	["Within-study bias","Heterogeneity"]
AE:ME-CT	0	No concerns	Low risk	No concerns	No concerns	Some concerns	Major concerns	Very low	["Heterogeneity","Incoherence"]
AE:RT	0	Some concerns	Low risk	No concerns	No concerns	Major concerns	No concerns	Very low	["Within-study bias","Heterogeneity"]
AE:RT-CT	0	Some concerns	Low risk	No concerns	No concerns	Some concerns	No concerns	Low	["Within-study bias","Heterogeneity"]
AE-CT:CT	0	Major concerns	Low risk	No concerns	No concerns	Some concerns	Some concerns	Very low	["Within-study bias","Heterogeneity","Incoherence"]
AE-CT:D/VRE	0	No concerns	Low risk	No concerns	No concerns	Major concerns	No concerns	Low	["Heterogeneity"]
AE-CT:MBE	0	Some concerns	Low risk	No concerns	No concerns	Some concerns	No concerns	Low	["Within-study bias","Heterogeneity"]
AE-CT:MBE-CT	0	No concerns	Low risk	No concerns	No concerns	Major concerns	Some concerns	Very low	["Heterogeneity","Incoherence"]
AE-CT:ME	0	Major concerns	Low risk	No concerns	No concerns	Some concerns	Major concerns	Very low	["Within-study bias","Heterogeneity","Incoherence"]
AE-CT:ME-CT	0	Some concerns	Low risk	No concerns	No concerns	Some concerns	No concerns	Low	["Within-study bias","Heterogeneity"]
AE-CT:RT	0	Some concerns	Low risk	No concerns	No concerns	Major concerns	Some concerns	Very low	["Within-study bias","Heterogeneity","Incoherence"]
AE-CT:RT-CT	0	Some concerns	Low risk	No concerns	No concerns	Some concerns	No concerns	Low	["Within-study bias","Heterogeneity"]
CT:D/VRE	0	No concerns	Low risk	No concerns	Some concerns	Some concerns	Some concerns	Very low	["Imprecision","Heterogeneity","Incoherence"]

Comparison	Number of studies	Within-study bias	Reporting bias	Indirectness	Imprecision	Heterogeneity	Incoherence	Confidence rating	Reason (s) for downgrading
CT:MBE	0	Some concerns	Low risk	No concerns	No concerns	Major concerns	Major concerns	Very low	["Within-study bias","Heterogeneity","Incoherence"]
CT:MBE-CT	0	No concerns	Low risk	No concerns	No concerns	Major concerns	Some concerns	Very low	["Heterogeneity","Incoherence"]
CT:ME	0	Some concerns	Low risk	No concerns	No concerns	Some concerns	Major concerns	Very low	["Within-study bias","Heterogeneity","Incoherence"]
CT:ME-CT	0	No concerns	Low risk	No concerns	No concerns	Major concerns	No concerns	Low	["Heterogeneity"]
CT:RT	0	Some concerns	Low risk	No concerns	Some concerns	No concerns	No concerns	Low	["Within-study bias","Imprecision"]
CT:RT-CT	0	Some concerns	Low risk	No concerns	No concerns	Major concerns	Major concerns	Very low	["Within-study bias","Heterogeneity","Incoherence"]
D/VRE:MBE	0	Some concerns	Low risk	No concerns	No concerns	Some concerns	Some concerns	Low	["Within-study bias","Heterogeneity","Incoherence"]
D/VRE:MBE-CT	0	No concerns	Low risk	No concerns	No concerns	Major concerns	No concerns	Low	["Heterogeneity"]
D/VRE:ME	0	No concerns	Low risk	No concerns	No concerns	Major concerns	No concerns	Low	["Heterogeneity"]
D/VRE:ME-CT	0	No concerns	Low risk	No concerns	No concerns	Major concerns	Some concerns	Very low	["Heterogeneity","Incoherence"]
D/VRE:RT	0	Some concerns	Low risk	No concerns	Some concerns	No concerns	No concerns	Low	["Within-study bias","Imprecision"]
D/VRE:RT-CT	0	Some concerns	Low risk	No concerns	No concerns	Some concerns	No concerns	Low	["Within-study bias","Heterogeneity"]
MBE:MBE-CT	0	No concerns	Low risk	No concerns	No concerns	Major concerns	No concerns	Low	["Heterogeneity"]
MBE:ME	0	Some concerns	Low risk	No concerns	Some concerns	No concerns	No concerns	Low	["Within-study bias","Imprecision"]
MBE:ME-CT	0	Some concerns	Low risk	No concerns	Some concerns	Some concerns	No concerns	Very low	["Within-study bias","Imprecision","Heterogeneity"]
MBE:RT	0	Some concerns	Low risk	No concerns	No concerns	No concerns	Some concerns	Low	["Within-study bias","Incoherence"]
MBE:RT-CT	0	Some concerns	Low risk	No concerns	Some concerns	Some concerns	No concerns	Very low	["Within-study bias","Imprecision","Heterogeneity"]
MBE:WLC	0	Some concerns	Low risk	No concerns	No concerns	No concerns	No concerns	Moderate	["Within-study bias"]
MBE-CT:ME	0	No concerns	Low risk	No concerns	No concerns	Some concerns	Some concerns	Low	["Heterogeneity","Incoherence"]
MBE-CT:ME-CT	0	No concerns	Low risk	No concerns	No concerns	Major concerns	Major concerns	Very low	["Heterogeneity","Incoherence"]
MBE-CT:RT	0	No concerns	Low risk	No concerns	Some concerns	Some concerns	No concerns	Low	["Imprecision","Heterogeneity"]
MBE-CT:RT-CT	0	No concerns	Low risk	No concerns	No concerns	Some concerns	No concerns	Moderate	["Heterogeneity"]
MBE-CT:WLC	0	No concerns	Low risk	No concerns	No concerns	No concerns	No concerns	High	[]

Comparison	Number of studies	Within-study bias	Reporting bias	Indirectness	Imprecision	Heterogeneity	Incoherence	Confidence rating	Reason (s) for downgrading
ME-CT:WLC	0	Some concerns	Low risk	No concerns	No concerns	No concerns	No concerns	Moderate	["Within-study bias"]
RT:WLC	0	Some concerns	Low risk	No concerns	No concerns	Some concerns	Some concerns	Very low	["Within-study bias","Heterogeneity","Incoherence"]
RT-CT:WLC	0	Some concerns	Low risk	No concerns	No concerns	No concerns	No concerns	Moderate	["Within-study bias"]

Supplementary Table 104. GRADE certainty of evidence for the ADAS-Cog outcome in older adults with mild cognitive impairment.

Comparison	Number of studies	Within-study bias	Reporting bias	Indirectness	Imprecision	Heterogeneity	Incoherence	Confidence rating	Reason (s) for downgrading
AC:AE	1	Some concerns	Low risk	No concerns	Major concerns	No concerns	No concerns	Very low	["Within-study bias","Imprecision"]
AC:CT	2	No concerns	Low risk	No concerns	No concerns	No concerns	No concerns	High	[]
AC:MBE	1	Some concerns	Low risk	No concerns	No concerns	Some concerns	No concerns	Low	["Within-study bias","Heterogeneity"]
AC:ME	2	No concerns	Low risk	No concerns	No concerns	No concerns	No concerns	High	[]
AC:ME-CT	2	No concerns	Low risk	No concerns	No concerns	Some concerns	No concerns	Moderate	["Heterogeneity"]
AE:MBE	1	Some concerns	Low risk	No concerns	No concerns	Some concerns	No concerns	Low	["Within-study bias","Heterogeneity"]
AE-CT:WLC	1	Major concerns	Low risk	No concerns	No concerns	No concerns	No concerns	Low	["Within-study bias"]
CT:ME	1	No concerns	Low risk	No concerns	No concerns	No concerns	No concerns	High	[]
CT:ME-CT	1	No concerns	Low risk	No concerns	No concerns	No concerns	No concerns	High	[]
D/VRE:MBE	1	No concerns	Low risk	No concerns	Some concerns	No concerns	No concerns	Moderate	["Imprecision"]
D/VRE:WLC	1	No concerns	Low risk	No concerns	Some concerns	No concerns	No concerns	Moderate	["Imprecision"]
MBE:WLC	1	No concerns	Low risk	No concerns	Some concerns	Some concerns	No concerns	Low	["Imprecision","Heterogeneity"]
ME:ME-CT	1	No concerns	Low risk	No concerns	No concerns	No concerns	No concerns	High	[]

Comparison	Number of studies	Within-study bias	Reporting bias	Indirectness	Imprecision	Heterogeneity	Incoherence	Confidence rating	Reason<br><br>(s) for downgrading
RT:WLC	1	No concerns	Low risk	No concerns	No concerns	No concerns	No concerns	High	[]
AC:AE-CT	0	No concerns	Low risk	No concerns	Major concerns	No concerns	No concerns	Low	["Imprecision"]
AC:D/VRE	0	No concerns	Low risk	No concerns	No concerns	Some concerns	No concerns	Moderate	["Heterogeneity"]
AC:RT	0	No concerns	Low risk	No concerns	Some concerns	Some concerns	No concerns	Low	["Imprecision","Heterogeneity"]
AC:WLC	0	No concerns	Low risk	No concerns	No concerns	Some concerns	No concerns	Moderate	["Heterogeneity"]
AE:AE-CT	0	No concerns	Low risk	No concerns	Major concerns	No concerns	No concerns	Low	["Imprecision"]
AE:CT	0	Some concerns	Low risk	No concerns	Some concerns	Some concerns	No concerns	Very low	["Within-study bias","Imprecision","Heterogeneity"]
AE:D/VRE	0	No concerns	Low risk	No concerns	No concerns	Some concerns	No concerns	Moderate	["Heterogeneity"]
AE:ME	0	Some concerns	Low risk	No concerns	Major concerns	No concerns	No concerns	Very low	["Within-study bias","Imprecision"]
AE:ME-CT	0	Some concerns	Low risk	No concerns	Some concerns	Some concerns	No concerns	Very low	["Within-study bias","Imprecision","Heterogeneity"]
AE:RT	0	No concerns	Low risk	No concerns	Some concerns	Some concerns	No concerns	Low	["Imprecision","Heterogeneity"]
AE:WLC	0	No concerns	Low risk	No concerns	Some concerns	No concerns	No concerns	Moderate	["Imprecision"]
AE-CT:CT	0	No concerns	Low risk	No concerns	Major concerns	No concerns	No concerns	Low	["Imprecision"]
AE-CT:D/VRE	0	No concerns	Low risk	No concerns	No concerns	No concerns	No concerns	High	[]
AE-CT:MBE	0	No concerns	Low risk	No concerns	Some concerns	Some concerns	No concerns	Low	["Imprecision","Heterogeneity"]
AE-CT:ME	0	No concerns	Low risk	No concerns	Major concerns	No concerns	No concerns	Low	["Imprecision"]
AE-CT:ME-CT	0	No concerns	Low risk	No concerns	Some concerns	Some concerns	No concerns	Low	["Imprecision","Heterogeneity"]
AE-CT:RT	0	No concerns	Low risk	No concerns	No concerns	Some concerns	No concerns	Moderate	["Heterogeneity"]
CT:D/VRE	0	No concerns	Low risk	No concerns	No concerns	No concerns	No concerns	High	[]
CT:MBE	0	No concerns	Low risk	No concerns	No concerns	Some concerns	No concerns	Moderate	["Heterogeneity"]
CT:RT	0	No concerns	Low risk	No concerns	Some concerns	Some concerns	No concerns	Low	["Imprecision","Heterogeneity"]
CT:WLC	0	No concerns	Low risk	No concerns	No concerns	Some concerns	No concerns	Moderate	["Heterogeneity"]
D/VRE:ME	0	No concerns	Low risk	No concerns	No concerns	Some concerns	No concerns	Moderate	["Heterogeneity"]

Comparison	Number of studies	Within-study bias	Reporting bias	Indirectness	Imprecision	Heterogeneity	Incoherence	Confidence rating	Reason (s) for downgrading
D/VRE:ME-CT	0	No concerns	Low risk	No concerns	No concerns	No concerns	No concerns	High	[]
D/VRE:RT	0	No concerns	Low risk	No concerns	No concerns	No concerns	No concerns	High	[]
MBE:ME	0	No concerns	Low risk	No concerns	No concerns	Some concerns	No concerns	Moderate	["Heterogeneity"]
MBE:ME-CT	0	No concerns	Low risk	No concerns	No concerns	Some concerns	No concerns	Moderate	["Heterogeneity"]
MBE:RT	0	No concerns	Low risk	No concerns	No concerns	Some concerns	No concerns	Moderate	["Heterogeneity"]
ME:RT	0	No concerns	Low risk	No concerns	Some concerns	Some concerns	No concerns	Low	["Imprecision","Heterogeneity"]
ME:WLC	0	No concerns	Low risk	No concerns	Some concerns	No concerns	No concerns	Moderate	["Imprecision"]
ME-CT:RT	0	No concerns	Low risk	No concerns	Some concerns	Some concerns	No concerns	Low	["Imprecision","Heterogeneity"]
ME-CT:WLC	0	No concerns	Low risk	No concerns	No concerns	Some concerns	No concerns	Moderate	["Heterogeneity"]

Supplementary Table 105. GRADE certainty of evidence for the TMT-B outcome in older adults with mild cognitive impairment.

Comparison	Number of studies	Within-study bias	Reporting bias	Indirectness	Imprecision	Heterogeneity	Incoherence	Confidence rating	Reason (s) for downgrading
AC:AE	3	Some concerns	Low risk	No concerns	No concerns	Some concerns	No concerns	Low	["Within-study bias","Heterogeneity"]
AC:AE-CT	2	No concerns	Low risk	No concerns	Some concerns	Some concerns	No concerns	Low	["Imprecision","Heterogeneity"]
AC:CT	2	No concerns	Low risk	No concerns	No concerns	No concerns	No concerns	High	[]
AC:MBE	2	No concerns	Low risk	No concerns	No concerns	Some concerns	No concerns	Moderate	["Heterogeneity"]
AC:MBE-CT	1	No concerns	Low risk	No concerns	No concerns	No concerns	No concerns	High	[]
AC:ME	3	Some concerns	Low risk	No concerns	No concerns	Some concerns	No concerns	Low	["Within-study bias","Heterogeneity"]
AC:ME-CT	1	Some concerns	Low risk	No concerns	Some concerns	Some concerns	No concerns	Very low	["Within-study bias","Imprecision","Heterogeneity"]
AE:ME	1	Some concerns	Low risk	No concerns	No concerns	Some concerns	No concerns	Low	["Within-study bias","Heterogeneity"]

Comparison	Number of studies	Within-study bias	Reporting bias	Indirectness	Imprecision	Heterogeneity	Incoherence	Confidence rating	Reason (s) for downgrading
CT:MBE	1	No concerns	Low risk	No concerns	No concerns	Some concerns	No concerns	Moderate	["Heterogeneity"]
CT:WLC	2	No concerns	Low risk	No concerns	Some concerns	No concerns	Some concerns	Low	["Imprecision","Incoherence"]
D/VRE:MBE	1	No concerns	Low risk	No concerns	No concerns	Some concerns	Some concerns	Low	["Heterogeneity","Incoherence"]
D/VRE:WLC	1	No concerns	Low risk	No concerns	No concerns	No concerns	Some concerns	Moderate	["Incoherence"]
MBE:MBE-CT	1	No concerns	Low risk	No concerns	No concerns	Some concerns	No concerns	Moderate	["Heterogeneity"]
MBE:ME	1	No concerns	Low risk	No concerns	No concerns	No concerns	No concerns	High	[]
MBE:WLC	1	No concerns	Low risk	No concerns	Some concerns	Some concerns	Some concerns	Very low	["Imprecision","Heterogeneity","Incoherence"]
AC:D/VRE	0	No concerns	Low risk	No concerns	No concerns	No concerns	No concerns	High	[]
AC:WLC	0	No concerns	Low risk	No concerns	Major concerns	No concerns	Some concerns	Very low	["Imprecision","Incoherence"]
AE:AE-CT	0	Some concerns	Low risk	No concerns	Some concerns	Some concerns	No concerns	Very low	["Within-study bias","Imprecision","Heterogeneity"]
AE:CT	0	Some concerns	Low risk	No concerns	No concerns	Some concerns	No concerns	Low	["Within-study bias","Heterogeneity"]
AE:D/VRE	0	No concerns	Low risk	No concerns	No concerns	Some concerns	No concerns	Moderate	["Heterogeneity"]
AE:MBE	0	No concerns	Low risk	No concerns	No concerns	Some concerns	No concerns	Moderate	["Heterogeneity"]
AE:MBE-CT	0	No concerns	Low risk	No concerns	No concerns	Some concerns	No concerns	Moderate	["Heterogeneity"]
AE:ME-CT	0	Some concerns	Low risk	No concerns	Some concerns	No concerns	No concerns	Low	["Within-study bias","Imprecision"]
AE:WLC	0	No concerns	Low risk	No concerns	Major concerns	No concerns	No concerns	Low	["Imprecision"]
AE-CT:CT	0	No concerns	Low risk	No concerns	No concerns	Some concerns	No concerns	Moderate	["Heterogeneity"]
AE-CT:D/VRE	0	No concerns	Low risk	No concerns	No concerns	No concerns	No concerns	High	[]
AE-CT:MBE	0	No concerns	Low risk	No concerns	Some concerns	No concerns	No concerns	Moderate	["Imprecision"]
AE-CT:MBE-CT	0	No concerns	Low risk	No concerns	No concerns	Some concerns	No concerns	Moderate	["Heterogeneity"]
AE-CT:ME	0	No concerns	Low risk	No concerns	Some concerns	No concerns	No concerns	Moderate	["Imprecision"]
AE-CT:ME-CT	0	Some concerns	Low risk	No concerns	Major concerns	No concerns	No concerns	Very low	["Within-study bias","Imprecision"]
AE-CT:WLC	0	No concerns	Low risk	No concerns	Major concerns	No concerns	No concerns	Low	["Imprecision"]

Comparison	Number of studies	Within-study bias	Reporting bias	Indirectness	Imprecision	Heterogeneity	Incoherence	Confidence rating	Reason<br><br>(s) for downgrading
CT:D/VRE	0	No concerns	Low risk	No concerns	Some concerns	No concerns	No concerns	Moderate	["Imprecision"]
CT:MBE-CT	0	No concerns	Low risk	No concerns	No concerns	Some concerns	No concerns	Moderate	["Heterogeneity"]
CT:ME	0	No concerns	Low risk	No concerns	Some concerns	No concerns	No concerns	Moderate	["Imprecision"]
CT:ME-CT	0	Some concerns	Low risk	No concerns	No concerns	Some concerns	No concerns	Low	["Within-study bias","Heterogeneity"]
D/VRE:MBE-CT	0	No concerns	Low risk	No concerns	Some concerns	Some concerns	No concerns	Low	["Imprecision","Heterogeneity"]
D/VRE:ME	0	No concerns	Low risk	No concerns	No concerns	Some concerns	No concerns	Moderate	["Heterogeneity"]
D/VRE:ME-CT	0	No concerns	Low risk	No concerns	No concerns	No concerns	No concerns	High	[]
MBE:ME-CT	0	No concerns	Low risk	No concerns	Some concerns	No concerns	No concerns	Moderate	["Imprecision"]
MBE-CT:ME	0	No concerns	Low risk	No concerns	No concerns	Some concerns	No concerns	Moderate	["Heterogeneity"]
MBE-CT:ME-CT	0	No concerns	Low risk	No concerns	No concerns	Some concerns	No concerns	Moderate	["Heterogeneity"]
MBE-CT:WLC	0	No concerns	Low risk	No concerns	Some concerns	No concerns	No concerns	Moderate	["Imprecision"]
ME:ME-CT	0	Some concerns	Low risk	No concerns	Some concerns	No concerns	No concerns	Low	["Within-study bias","Imprecision"]
ME:WLC	0	No concerns	Low risk	No concerns	Some concerns	Some concerns	No concerns	Low	["Imprecision","Heterogeneity"]
ME-CT:WLC	0	No concerns	Low risk	No concerns	Major concerns	No concerns	No concerns	Low	["Imprecision"]

Supplementary Table 106. GRADE certainty of evidence for the DST outcome in older adults with mild cognitive impairment.

Comparison	Number of studies	Within-study bias	Reporting bias	Indirectness	Imprecision	Heterogeneity	Incoherence	Confidence rating	Reason<br><br>(s) for downgrading
AC:AE	3	Some concerns	Low risk	No concerns	Some concerns	Some concerns	No concerns	Very low	["Within-study bias","Imprecision","Heterogeneity"]
AC:AE-CT	2	No concerns	Low risk	No concerns	Some concerns	No concerns	No concerns	Moderate	["Imprecision"]
AC:CT	2	No concerns	Low risk	No concerns	No concerns	Some concerns	No concerns	Moderate	["Heterogeneity"]

Comparison	Number of studies	Within-study bias	Reporting bias	Indirectness	Imprecision	Heterogeneity	Incoherence	Confidence rating	Reason (s) for downgrading
AC:MBE	4	No concerns	Low risk	No concerns	No concerns	Some concerns	No concerns	Moderate	["Heterogeneity"]
AC:MBE-CT	2	No concerns	Low risk	No concerns	No concerns	Some concerns	No concerns	Moderate	["Heterogeneity"]
AC:ME	1	No concerns	Low risk	No concerns	No concerns	No concerns	Major concerns	Low	["Incoherence"]
AE-CT:CT	1	No concerns	Low risk	No concerns	Some concerns	Some concerns	No concerns	Low	["Imprecision","Heterogeneity"]
AE-CT:WLC	1	No concerns	Low risk	No concerns	Some concerns	No concerns	No concerns	Moderate	["Imprecision"]
CT:WLC	1	No concerns	Low risk	No concerns	No concerns	Some concerns	No concerns	Moderate	["Heterogeneity"]
MBE:MBE-CT	1	No concerns	Low risk	No concerns	Major concerns	No concerns	No concerns	Low	["Imprecision"]
MBE:ME	1	No concerns	Low risk	No concerns	Some concerns	No concerns	Major concerns	Very low	["Imprecision","Incoherence"]
RT:WLC	3	Some concerns	Low risk	No concerns	Some concerns	No concerns	No concerns	Low	["Within-study bias","Imprecision"]
AC:RT	0	No concerns	Low risk	No concerns	Major concerns	No concerns	No concerns	Low	["Imprecision"]
AC:WLC	0	No concerns	Low risk	No concerns	Major concerns	No concerns	No concerns	Low	["Imprecision"]
AE:AE-CT	0	No concerns	Low risk	No concerns	Some concerns	No concerns	No concerns	Moderate	["Imprecision"]
AE:CT	0	No concerns	Low risk	No concerns	No concerns	Some concerns	No concerns	Moderate	["Heterogeneity"]
AE:MBE	0	No concerns	Low risk	No concerns	Some concerns	No concerns	No concerns	Moderate	["Imprecision"]
AE:MBE-CT	0	No concerns	Low risk	No concerns	No concerns	Some concerns	No concerns	Moderate	["Heterogeneity"]
AE:ME	0	No concerns	Low risk	No concerns	No concerns	No concerns	No concerns	High	[]
AE:RT	0	No concerns	Low risk	No concerns	Major concerns	No concerns	No concerns	Low	["Imprecision"]
AE:WLC	0	No concerns	Low risk	No concerns	Major concerns	No concerns	No concerns	Low	["Imprecision"]
AE-CT:MBE	0	No concerns	Low risk	No concerns	Major concerns	No concerns	No concerns	Low	["Imprecision"]
AE-CT:MBE-CT	0	No concerns	Low risk	No concerns	Major concerns	No concerns	No concerns	Low	["Imprecision"]
AE-CT:ME	0	No concerns	Low risk	No concerns	Some concerns	No concerns	No concerns	Moderate	["Imprecision"]
AE-CT:RT	0	No concerns	Low risk	No concerns	Major concerns	No concerns	No concerns	Low	["Imprecision"]
CT:MBE	0	No concerns	Low risk	No concerns	Major concerns	No concerns	No concerns	Low	["Imprecision"]

Comparison	Number of studies	Within-study bias	Reporting bias	Indirectness	Imprecision	Heterogeneity	Incoherence	Confidence rating	Reason (s) for downgrading
CT:MBE-CT	0	No concerns	Low risk	No concerns	Major concerns	No concerns	No concerns	Low	["Imprecision"]
CT:ME	0	No concerns	Low risk	No concerns	Major concerns	No concerns	No concerns	Low	["Imprecision"]
CT:RT	0	No concerns	Low risk	No concerns	Some concerns	Some concerns	No concerns	Low	["Imprecision","Heterogeneity"]
MBE:RT	0	No concerns	Low risk	No concerns	Major concerns	No concerns	No concerns	Low	["Imprecision"]
MBE:WLC	0	No concerns	Low risk	No concerns	Some concerns	No concerns	No concerns	Moderate	["Imprecision"]
MBE-CT:ME	0	No concerns	Low risk	No concerns	Some concerns	Some concerns	No concerns	Low	["Imprecision","Heterogeneity"]
MBE-CT:RT	0	No concerns	Low risk	No concerns	Major concerns	No concerns	No concerns	Low	["Imprecision"]
MBE-CT:WLC	0	No concerns	Low risk	No concerns	Some concerns	No concerns	No concerns	Moderate	["Imprecision"]
ME:RT	0	No concerns	Low risk	No concerns	Some concerns	No concerns	No concerns	Moderate	["Imprecision"]
ME:WLC	0	No concerns	Low risk	No concerns	No concerns	No concerns	No concerns	High	[]

Supplementary Table 107. GRADE certainty of evidence for the SCWT outcome in older adults with mild cognitive impairment.

Comparison	Number of studies	Within-study bias	Reporting bias	Indirectness	Imprecision	Heterogeneity	Incoherence	Confidence rating	Reason (s) for downgrading
AE:AE-CT	1	No concerns	Low risk	No concerns	No concerns	Major concerns	No concerns	Low	["Heterogeneity"]
AE:CT	1	No concerns	Low risk	No concerns	No concerns	No concerns	No concerns	High	[]
AE:WLC	2	No concerns	Low risk	No concerns	No concerns	No concerns	No concerns	High	[]
AE-CT:CT	1	No concerns	Low risk	No concerns	No concerns	Major concerns	No concerns	Low	["Heterogeneity"]
AE-CT:WLC	1	No concerns	Low risk	No concerns	No concerns	Some concerns	No concerns	Moderate	["Heterogeneity"]

Comparison	Number of studies	Within-study bias	Reporting bias	Indirectness	Imprecision	Heterogeneity	Incoherence	Confidence rating	Reason (s) for downgrading
CT:MBE	1	No concerns	Low risk	No concerns	No concerns	Major concerns	No concerns	Low	["Heterogeneity"]
CT:WLC	1	No concerns	Low risk	No concerns	No concerns	Major concerns	No concerns	Low	["Heterogeneity"]
D/VRE:MBE	1	No concerns	Low risk	No concerns	No concerns	Some concerns	No concerns	Moderate	["Heterogeneity"]
D/VRE:WLC	1	No concerns	Low risk	No concerns	No concerns	Major concerns	No concerns	Low	["Heterogeneity"]
MBE:WLC	1	No concerns	Low risk	No concerns	No concerns	Major concerns	No concerns	Low	["Heterogeneity"]
RT:WLC	1	Some concerns	Low risk	No concerns	No concerns	Major concerns	No concerns	Very low	["Within-study bias","Heterogeneity"]
AE:D/VRE	0	No concerns	Low risk	No concerns	No concerns	Some concerns	No concerns	Moderate	["Heterogeneity"]
AE:MBE	0	No concerns	Low risk	No concerns	Some concerns	No concerns	No concerns	Moderate	["Imprecision"]
AE:RT	0	No concerns	Low risk	No concerns	No concerns	Major concerns	No concerns	Low	["Heterogeneity"]
AE-CT:D/VRE	0	No concerns	Low risk	No concerns	No concerns	Major concerns	No concerns	Low	["Heterogeneity"]
AE-CT:MBE	0	No concerns	Low risk	No concerns	No concerns	Some concerns	No concerns	Moderate	["Heterogeneity"]
AE-CT:RT	0	No concerns	Low risk	No concerns	No concerns	Some concerns	No concerns	Moderate	["Heterogeneity"]
CT:D/VRE	0	No concerns	Low risk	No concerns	No concerns	Major concerns	No concerns	Low	["Heterogeneity"]
CT:RT	0	No concerns	Low risk	No concerns	No concerns	Major concerns	No concerns	Low	["Heterogeneity"]

Comparison	Number of studies	Within-study bias	Reporting bias	Indirectness	Imprecision	Heterogeneity	Incoherence	Confidence rating	Reason (s) for downgrading
D/VRE: RT	0	No concerns	Low risk	No concerns	No concerns	Some concerns	No concerns	Moderate	["Heterogeneity"]
MBE: RT	0	No concerns	Low risk	No concerns	No concerns	Major concerns	No concerns	Low	["Heterogeneity"]

Supplementary Table 108. GRADE certainty of evidence for the DRA outcome in older adults with mild cognitive impairment.

Comparison	Number of studies	Within-study bias	Reporting bias	Indirectness	Imprecision	Heterogeneity	Incoherence	Confidence rating	Reason (s) for downgrading
AC:AE	5	Some concerns	Low risk	No concerns	No concerns	Some concerns	No concerns	Low	["Within-study bias","Heterogeneity"]
AC:AE-CT	1	No concerns	Low risk	No concerns	Some concerns	Some concerns	No concerns	Low	["Imprecision","Heterogeneity"]
AC:CT	3	Some concerns	Low risk	No concerns	Some concerns	Some concerns	No concerns	Very low	["Within-study bias","Imprecision","Heterogeneity"]
AC:MBE	2	Some concerns	Low risk	No concerns	Major concerns	No concerns	No concerns	Very low	["Within-study bias","Imprecision"]
AC:ME-CT	1	No concerns	Low risk	No concerns	No concerns	No concerns	No concerns	High	[]
AE:MBE	1	Some concerns	Low risk	No concerns	Some concerns	No concerns	No concerns	Low	["Within-study bias","Imprecision"]
AE:ME	1	Some concerns	Low risk	No concerns	Major concerns	No concerns	No concerns	Very low	["Within-study bias","Imprecision"]
AE:WLC	3	Major concerns	Low risk	No concerns	No concerns	No concerns	No concerns	Low	["Within-study bias"]
CT:D/VRE	1	Some concerns	Low risk	No concerns	Major concerns	No concerns	No concerns	Very low	["Within-study bias","Imprecision"]
CT:MBE	2	Some concerns	Low risk	No concerns	Major concerns	No concerns	No concerns	Very low	["Within-study bias","Imprecision"]
D/VRE:ME-CT	1	Major concerns	Low risk	No concerns	Major concerns	No concerns	No concerns	Very low	["Within-study bias","Imprecision"]
D/VRE:WLC	1	Some concerns	Low risk	No concerns	Some concerns	No concerns	No concerns	Low	["Within-study bias","Imprecision"]

Comparison	Number of studies	Within-study bias	Reporting bias	Indirectness	Imprecision	Heterogeneity	Incoherence	Confidence rating	Reason (s) for downgrading
ME:ME-CT	1	Some concerns	Low risk	No concerns	Some concerns	Some concerns	No concerns	Very low	["Within-study bias","Imprecision","Heterogeneity"]
ME:RT	1	Some concerns	Low risk	No concerns	No concerns	No concerns	No concerns	Moderate	["Within-study bias"]
ME:RT-CT	1	Some concerns	Low risk	No concerns	Major concerns	No concerns	No concerns	Very low	["Within-study bias","Imprecision"]
ME:WLC	1	Some concerns	Low risk	No concerns	No concerns	No concerns	No concerns	Moderate	["Within-study bias"]
ME-CT:RT	1	Some concerns	Low risk	No concerns	No concerns	No concerns	No concerns	Moderate	["Within-study bias"]
ME-CT:RT-CT	1	Some concerns	Low risk	No concerns	Major concerns	No concerns	No concerns	Very low	["Within-study bias","Imprecision"]
RT:RT-CT	1	Some concerns	Low risk	No concerns	No concerns	No concerns	No concerns	Moderate	["Within-study bias"]
AC:D/VRE	0	Some concerns	Low risk	No concerns	Major concerns	No concerns	No concerns	Very low	["Within-study bias","Imprecision"]
AC:ME	0	Some concerns	Low risk	No concerns	Some concerns	No concerns	No concerns	Low	["Within-study bias","Imprecision"]
AC:RT	0	Some concerns	Low risk	No concerns	No concerns	No concerns	No concerns	Moderate	["Within-study bias"]
AC:RT-CT	0	Some concerns	Low risk	No concerns	Major concerns	No concerns	No concerns	Very low	["Within-study bias","Imprecision"]
AC:WLC	0	Major concerns	Low risk	No concerns	Some concerns	No concerns	No concerns	Very low	["Within-study bias","Imprecision"]
AE:AE-CT	0	No concerns	Low risk	No concerns	Major concerns	No concerns	No concerns	Low	["Imprecision"]
AE:CT	0	Some concerns	Low risk	No concerns	Some concerns	No concerns	No concerns	Low	["Within-study bias","Imprecision"]
AE:D/VRE	0	Some concerns	Low risk	No concerns	Some concerns	Some concerns	No concerns	Very low	["Within-study bias","Imprecision","Heterogeneity"]
AE:ME-CT	0	No concerns	Low risk	No concerns	Some concerns	Some concerns	No concerns	Low	["Imprecision","Heterogeneity"]
AE:RT	0	Some concerns	Low risk	No concerns	No concerns	No concerns	No concerns	Moderate	["Within-study bias"]
AE:RT-CT	0	Some concerns	Low risk	No concerns	Major concerns	No concerns	No concerns	Very low	["Within-study bias","Imprecision"]
AE-CT:CT	0	No concerns	Low risk	No concerns	Major concerns	No concerns	No concerns	Low	["Imprecision"]
AE-CT:D/VRE	0	No concerns	Low risk	No concerns	Major concerns	No concerns	No concerns	Low	["Imprecision"]
AE-CT:MBE	0	No concerns	Low risk	No concerns	Major concerns	No concerns	No concerns	Low	["Imprecision"]
AE-CT:ME	0	Some concerns	Low risk	No concerns	Major concerns	No concerns	No concerns	Very low	["Within-study bias","Imprecision"]

Comparison	Number of studies	Within-study bias	Reporting bias	Indirectness	Imprecision	Heterogeneity	Incoherence	Confidence rating	Reason (s) for downgrading
AE-CT:ME-CT	0	No concerns	Low risk	No concerns	Major concerns	No concerns	No concerns	Low	["Imprecision"]
AE-CT:RT	0	Some concerns	Low risk	No concerns	No concerns	No concerns	No concerns	Moderate	["Within-study bias"]
AE-CT:RT-CT	0	Some concerns	Low risk	No concerns	Major concerns	No concerns	No concerns	Very low	["Within-study bias","Imprecision"]
AE-CT:WLC	0	No concerns	Low risk	No concerns	No concerns	No concerns	No concerns	High	[]
CT:ME	0	Some concerns	Low risk	No concerns	Some concerns	Some concerns	No concerns	Very low	["Within-study bias","Imprecision","Heterogeneity"]
CT:ME-CT	0	Some concerns	Low risk	No concerns	Major concerns	No concerns	No concerns	Very low	["Within-study bias","Imprecision"]
CT:RT	0	Some concerns	Low risk	No concerns	No concerns	No concerns	No concerns	Moderate	["Within-study bias"]
CT:RT-CT	0	Some concerns	Low risk	No concerns	Major concerns	No concerns	No concerns	Very low	["Within-study bias","Imprecision"]
CT:WLC	0	Some concerns	Low risk	No concerns	Some concerns	No concerns	No concerns	Low	["Within-study bias","Imprecision"]
D/VRE:MBE	0	Some concerns	Low risk	No concerns	Major concerns	No concerns	No concerns	Very low	["Within-study bias","Imprecision"]
D/VRE:ME	0	Some concerns	Low risk	No concerns	Major concerns	No concerns	No concerns	Very low	["Within-study bias","Imprecision"]
D/VRE:RT	0	Some concerns	Low risk	No concerns	No concerns	No concerns	No concerns	Moderate	["Within-study bias"]
D/VRE:RT-CT	0	Some concerns	Low risk	No concerns	Major concerns	No concerns	No concerns	Very low	["Within-study bias","Imprecision"]
MBE:ME	0	Some concerns	Low risk	No concerns	Some concerns	Some concerns	No concerns	Very low	["Within-study bias","Imprecision","Heterogeneity"]
MBE:ME-CT	0	No concerns	Low risk	No concerns	Major concerns	No concerns	No concerns	Low	["Imprecision"]
MBE:RT	0	Some concerns	Low risk	No concerns	No concerns	No concerns	No concerns	Moderate	["Within-study bias"]
MBE:RT-CT	0	Some concerns	Low risk	No concerns	Major concerns	No concerns	No concerns	Very low	["Within-study bias","Imprecision"]
MBE:WLC	0	Some concerns	Low risk	No concerns	Some concerns	Some concerns	No concerns	Very low	["Within-study bias","Imprecision","Heterogeneity"]
ME-CT:WLC	0	Some concerns	Low risk	No concerns	Some concerns	No concerns	No concerns	Low	["Within-study bias","Imprecision"]
RT:WLC	0	Some concerns	Low risk	No concerns	No concerns	No concerns	No concerns	Moderate	["Within-study bias"]
RT-CT:WLC	0	Some concerns	Low risk	No concerns	Major concerns	No concerns	No concerns	Very low	["Within-study bias","Imprecision"]

Supplementary Table 109. GRADE certainty of evidence for the MFA outcome in older adults with mild cognitive impairment.

Comparison	Number of studies	Within-study bias	Reporting bias	Indirectness	Imprecision	Heterogeneity	Incoherence	Confidence rating	Reason (s) for downgrading
AC:AE	8	Some concerns	Low risk	No concerns	No concerns	Some concerns	Major concerns	Very low	["Within-study bias","Heterogeneity","Incoherence"]
AC:CT	2	Some concerns	Low risk	No concerns	Some concerns	No concerns	Major concerns	Very low	["Within-study bias","Imprecision","Incoherence"]
AC:MBE-CT	1	Some concerns	Low risk	No concerns	No concerns	No concerns	Some concerns	Low	["Within-study bias","Incoherence"]
AC:ME-CT	1	Some concerns	Low risk	No concerns	Some concerns	No concerns	Some concerns	Very low	["Within-study bias","Imprecision","Incoherence"]
AE:ME	1	Some concerns	Low risk	No concerns	Some concerns	No concerns	No concerns	Low	["Within-study bias","Imprecision"]
AE:WLC	1	Major concerns	Low risk	No concerns	No concerns	No concerns	No concerns	Low	["Within-study bias"]
CT:MBE	1	No concerns	Low risk	No concerns	Some concerns	No concerns	No concerns	Moderate	["Imprecision"]
RT:WLC	1	Some concerns	Low risk	No concerns	Some concerns	No concerns	Major concerns	Very low	["Within-study bias","Imprecision","Incoherence"]
AC:MBE	0	No concerns	Low risk	No concerns	No concerns	No concerns	No concerns	High	[]
AC:ME	0	Some concerns	Low risk	No concerns	Some concerns	No concerns	No concerns	Low	["Within-study bias","Imprecision"]
AC:RT	0	Some concerns	Low risk	No concerns	No concerns	Some concerns	No concerns	Low	["Within-study bias","Heterogeneity"]
AC:WLC	0	Major concerns	Low risk	No concerns	No concerns	No concerns	No concerns	Low	["Within-study bias"]
AE:CT	0	Some concerns	Low risk	No concerns	Some concerns	No concerns	Major concerns	Very low	["Within-study bias","Imprecision","Incoherence"]
AE:MBE	0	Some concerns	Low risk	No concerns	No concerns	No concerns	No concerns	Moderate	["Within-study bias"]
AE:MBE-CT	0	Some concerns	Low risk	No concerns	Some concerns	No concerns	Some concerns	Very low	["Within-study bias","Imprecision","Incoherence"]
AE:ME-CT	0	Some concerns	Low risk	No concerns	Some concerns	No concerns	No concerns	Low	["Within-study bias","Imprecision"]
AE:RT	0	Some concerns	Low risk	No concerns	No concerns	No concerns	No concerns	Moderate	["Within-study bias"]
CT:MBE-CT	0	Some concerns	Low risk	No concerns	Some concerns	No concerns	No concerns	Low	["Within-study bias","Imprecision"]
CT:ME	0	Some concerns	Low risk	No concerns	No concerns	No concerns	Some concerns	Low	["Within-study bias","Incoherence"]

Comparison	Number of studies	Within-study bias	Reporting bias	Indirectness	Imprecision	Heterogeneity	Incoherence	Confidence rating	Reason (s) for downgrading
CT:ME-CT	0	Some concerns	Low risk	No concerns	Some concerns	No concerns	No concerns	Low	["Within-study bias","Imprecision"]
CT:RT	0	Some concerns	Low risk	No concerns	No concerns	No concerns	Some concerns	Low	["Within-study bias","Incoherence"]
CT:WLC	0	Some concerns	Low risk	No concerns	Some concerns	No concerns	No concerns	Low	["Within-study bias","Imprecision"]
MBE:MBE-CT	0	Some concerns	Low risk	No concerns	Some concerns	No concerns	Major concerns	Very low	["Within-study bias","Imprecision","Incoherence"]
MBE:ME	0	Some concerns	Low risk	No concerns	Some concerns	No concerns	Major concerns	Very low	["Within-study bias","Imprecision","Incoherence"]
MBE:ME-CT	0	Some concerns	Low risk	No concerns	No concerns	No concerns	No concerns	Moderate	["Within-study bias"]
MBE:RT	0	Some concerns	Low risk	No concerns	Some concerns	No concerns	Major concerns	Very low	["Within-study bias","Imprecision","Incoherence"]
MBE:WLC	0	Some concerns	Low risk	No concerns	No concerns	No concerns	Some concerns	Low	["Within-study bias","Incoherence"]
MBE-CT:ME	0	Some concerns	Low risk	No concerns	Some concerns	No concerns	Major concerns	Very low	["Within-study bias","Imprecision","Incoherence"]
MBE-CT:ME-CT	0	Some concerns	Low risk	No concerns	Some concerns	No concerns	No concerns	Low	["Within-study bias","Imprecision"]
MBE-CT:RT	0	Some concerns	Low risk	No concerns	Some concerns	No concerns	No concerns	Low	["Within-study bias","Imprecision"]
MBE-CT:WLC	0	Some concerns	Low risk	No concerns	No concerns	No concerns	Some concerns	Low	["Within-study bias","Incoherence"]
ME:ME-CT	0	Some concerns	Low risk	No concerns	Some concerns	No concerns	No concerns	Low	["Within-study bias","Imprecision"]
ME:RT	0	Some concerns	Low risk	No concerns	No concerns	Some concerns	No concerns	Low	["Within-study bias","Heterogeneity"]
ME:WLC	0	Some concerns	Low risk	No concerns	Some concerns	No concerns	Major concerns	Very low	["Within-study bias","Imprecision","Incoherence"]
ME-CT:RT	0	Some concerns	Low risk	No concerns	No concerns	No concerns	No concerns	Moderate	["Within-study bias"]
ME-CT:WLC	0	Some concerns	Low risk	No concerns	No concerns	No concerns	No concerns	Moderate	["Within-study bias"]

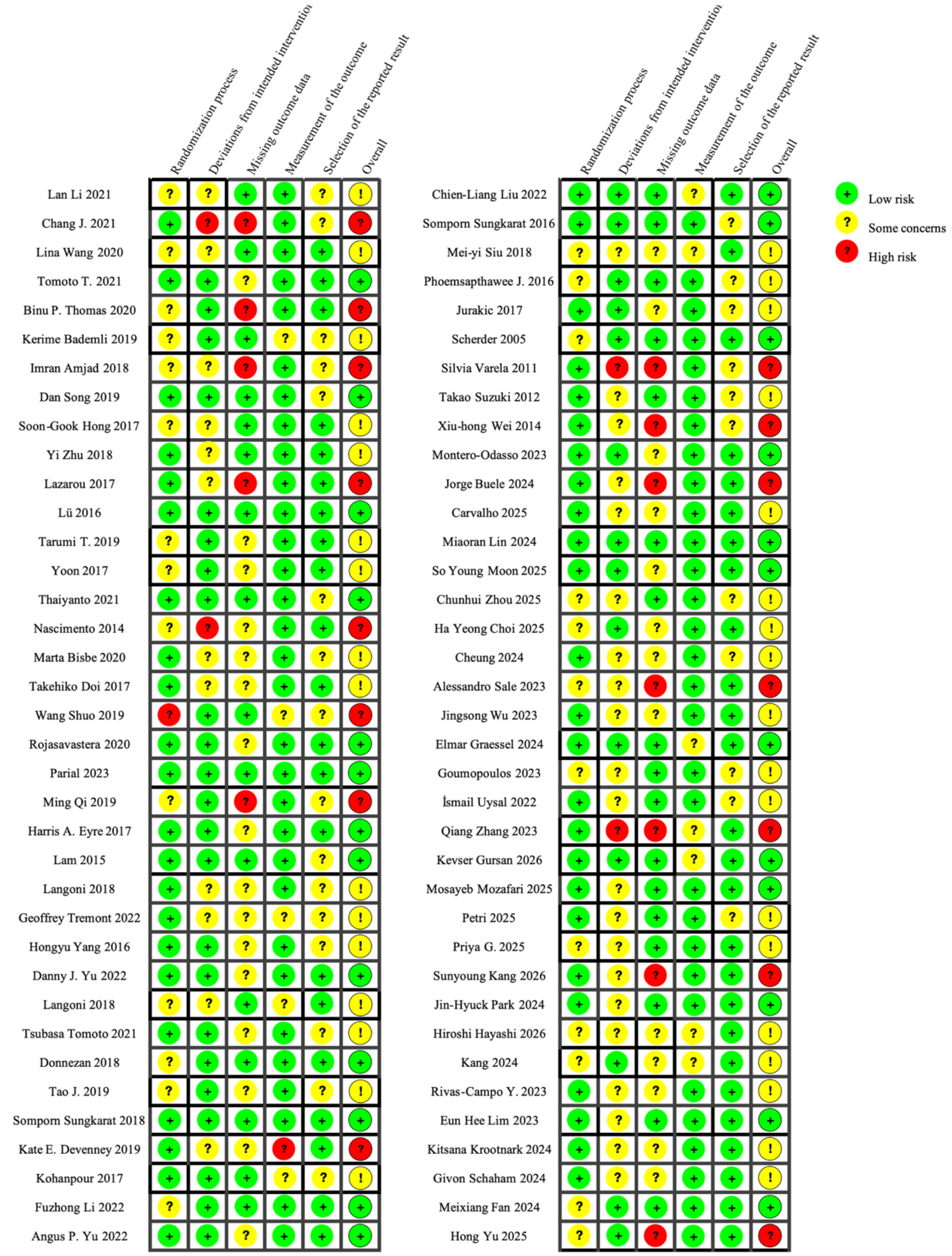

Supplementary Figure 1

RoB 2.0 quality assessment of studies

MMSE

MBE	0.29 (-2.33,2.91)	0.42 (-2.87,3.70)	0.47 (-2.43,3.38)	1.58 (-1.61,4.77)	1.62 (-1.96,5.19)	1.84 (-1.40,5.08)	2.04 (-0.29,4.37)	2.42 (-0.88,5.72)	2.65 (-0.10,5.39)	**4.23 (1.42,7.03)**
Rank 1st	ME	0.13 (-2.03,2.29)	0.18 (-1.56,1.92)	1.29 (-0.95,3.54)	1.32 (-1.35,4.00)	1.55 (-0.99,4.09)	1.75 (0.56,2.94)	2.13 (-0.06,4.31)	**2.36 (0.70,4.01)**	**3.94 (2.43,5.44)**
	Rank 2nd	RT-CT	0.05 (-2.11,2.21)	1.16 (-1.88,4.21)	1.20 (-2.19,4.59)	1.42 (-1.80,4.64)	1.62 (-0.69,3.93)	2.00 (-0.28,4.28)	2.23 (-0.39,4.85)	**3.81 (1.24,6.38)**
Rank 1st		Rank	ME-CT	1.11 (-1.57,3.79)	1.14 (-1.94,4.23)	1.37 (-1.46,4.20)	1.57 (-0.16,3.30)	1.95 (-0.24,4.14)	**2.18 (0.01,4.34)**	**3.76 (1.60,5.91)**
MBE	Rank 2nd		Rank 4th	CT	0.03 (-2.92,2.99)	0.26 (-2.87,3.38)	0.46 (-1.72,2.63)	0.84 (-2.23,3.90)	1.06 (-1.22,3.35)	**2.64 (0.68,4.60)**
0.71 (-1.62,3.03)	AE-CT	Rank		Rank 5th	D/VRE	0.22 (-3.29,3.74)	0.42 (-2.28,3.13)	0.80 (-2.60,4.21)	1.03 (-1.63,3.70)	**2.61 (0.40,4.82)**
0.95 (-0.79,2.68)	0.24 (-1.80,2.27)	AE	Rank 4th		Rank 6th	MBE-CT	0.20 (-2.04,2.44)	0.58 (-2.66,3.82)	0.81 (-1.86,3.47)	2.39 (-0.35,5.12)
**2.34 (0.51,4.18)**	1.64 (-0.58,3.85)	1.40 (-0.19,2.98)	CT	Rank 5th		Rank 7th	AC	0.38 (-1.96,2.72)	0.61 (-0.83,2.05)	**2.19 (0.63,3.75)**
**2.49 (0.58,4.40)**	1.78 (-0.51,4.07)	1.54 (-0.18,3.27)	0.15 (-1.56,1.85)	ME	Rank 6th		Rank 8th	RT	0.23 (-2.41,2.87)	1.81 (-0.79,4.40)
**2.52 (0.28,4.77)**	1.82 (-0.73,4.36)	1.58 (-0.45,3.61)	0.18 (-1.66,2.02)	0.03 (-1.85,1.92)	D/VRE	Rank 7th		Rank 9th	AE	**1.58 (0.09,3.07)**
**2.62 (0.48,4.75)**	1.91 (-0.53,4.34)	1.67 (-0.11,3.45)	0.27 (-1.55,2.10)	0.13 (-2.00,2.25)	0.09 (-2.19,2.38)	RT	Rank 8th		Rank 10th	WLC
**3.06 (1.62,4.49)**	**2.35 (0.42,4.29)**	**2.11 (0.89,3.34)**	0.72 (-0.56,1.99)	0.57 (-0.71,1.85)	0.54 (-1.29,2.36)	0.44 (-1.33,2.22)	AC	Rank 9th		Rank 11th
**4.06 (1.08,7.04)**	**3.35 (0.10,6.61)**	**3.11 (0.22,6.00)**	1.72 (-1.19,4.63)	1.57 (-1.34,4.48)	1.54 (-1.66,4.73)	1.44 (-1.72,4.60)	1.00 (-1.62,3.62)	MBE-CT	Rank 10th	
**4.35 (2.63,6.07)**	**3.64 (1.57,5.71)**	**3.40 (2.06,4.75)**	**2.01 (0.52,3.49)**	**1.86 (0.08,3.64)**	1.83 (-0.06,3.71)	1.73 (0.10,3.37)	1.29 (-0.08,2.66)	0.29 (-2.66,3.24)	WLC	

MoCA

Supplementary Figure 2

League table from the network meta-analysis comparing intervention nodes in older adults with mild cognitive impairment. Mean differences (MDs) with 95% confidence intervals (CIs) are presented for MoCA in the lower-left triangle and MMSE in the upper-right triangle. Symbols denote certainty of evidence according to the GRADE approach: * high; † moderate; ‡ low; § very low. AE, aerobic exercise; RT, resistance training; MBE, mind–body exercise; ME, multicomponent exercise; D/VRE, digital or virtual reality–based exercise; CT, cognitive training; AE-CT, aerobic exercise plus cognitive training; RT-CT, resistance training plus cognitive training; MBE-CT, mind–body exercise plus cognitive training; ME-CT, multicomponent exercise plus cognitive training.

ADAS-Cog

RT	1.72 (-1.89,5.34)	1.85 (-1.76,5.46)	2.42 (-1.22,6.06)	2.25 (-1.35,5.85)	2.28 (-1.24,5.81)	**4.62 (1.53,7.71)**	**5.02 (2.74,7.30)**	**6.42 (3.65,9.19)**	
Rank 1st	ME-CT	0.13 (-0.69,0.95)	0.70 (-1.64,3.03)	0.52 (-0.27,1.32)	0.56 (-0.25,1.36)	**2.90 (1.02,4.77)**	**3.30 (0.49,6.10)**	**4.70 (1.96,7.43)**	
	Rank 2nd	CT	0.57 (-1.75,2.89)	0.39 (-0.36,1.15)	0.43 (-0.34,1.20)	**2.77 (0.91,4.63)**	**3.17 (0.37,5.96)**	**4.57 (1.84,7.30)**	
Rank 1st		Rank 3rd	AE	-0.17 (-2.48,2.13)	-0.14 (-2.33,2.05)	**2.20 (0.28,4.12)**	2.60 (-0.23,5.43)	**4.00 (1.23,6.77)**	
D/VRE	Rank 2nd		Rank 5th	ME	0.03 (-0.69,0.76)	**2.37 (0.53,4.22)**	2.77 (-0.01,5.55)	**4.17 (1.46,6.89)**	
21.04 (-14.75,56.83)	MBE-CT	Rank 3rd		Rank 4th	AC	**2.34 (0.65,4.03)**	**2.74 (0.05,5.42)**	**4.14 (1.52,6.76)**	
26.72 (-9.61,63.06)	5.68 (-14.58,25.95)	CT	Rank 4th		Rank 6th	MBE	0.40 (-1.68,2.48)	1.80 (-0.20,3.80)	
33.05 (-0.19,66.30)	12.01 (-2.28,26.30)	6.33 (-12.43,25.08)	MBE	Rank 5th		Rank 7th	WLC	1.40 (-0.17,2.97)	
33.51 (-1.72,68.74)	12.47 (-4.54,29.48)	6.79 (-12.29,25.87)	0.46 (-12.42,13.34)	ME	Rank 6th		Rank 8th	D/VRE	
**41.42 (4.94,77.89)**	**20.38 (1.75,39.01)**	14.69 (-7.88,37.27)	8.37 (-7.59,24.32)	7.90 (-9.53,25.34)	AE	Rank 8th		Rank 9th	
**48.42 (19.97,76.87)**	27.38 (-7.00,61.76)	21.70 (-12.97,56.37)	15.37 (-16.46,47.19)	14.91 (-18.90,48.72)	7.00 (-27.81,41.82)	WLC	Rank 7th		
**47.08 (12.51,81.66)**	**26.05 (11.91,40.18)**	**20.36 (6.16,34.57)**	**14.03 (1.91,26.15)**	**13.57 (1.06,26.09)**	5.67 (-8.42,19.76)	-1.33 (-34.40,31.73)	AC	Rank 9th	
**53.97 (6.75,101.20)**	32.94 (-2.20,68.07)	27.25 (-7.91,62.42)	20.92 (-13.45,55.30)	20.46 (-14.05,54.98)	12.56 (-22.56,47.68)	5.56 (-40.58,51.69)	6.89 (-25.28,39.06)	AE-CT	Rank 10th
**58.08 (13.18,102.99)**	**37.05 (5.09,69.00)**	31.36 (-0.62,63.35)	25.03 (-6.08,56.15)	24.57 (-6.70,55.84)	16.67 (-15.27,48.60)	9.67 (-34.09,53.42)	11.00 (-17.66,39.66)	4.11 (-38.97,47.19)	ME-CT

TMT-B

Supplementary Figure 3

League table from the network meta-analysis comparing intervention nodes in older adults with mild cognitive impairment. Mean differences (MDs) with 95% confidence intervals (CIs) are presented for TMT-B in the lower-left triangle and ADAS-Cog in the upper-right triangle. Symbols denote certainty of evidence according to the GRADE approach: * high; † moderate; ‡ low; § very low. AE, aerobic exercise; RT, resistance training; MBE, mind–body exercise; ME, multicomponent exercise; D/VRE, digital or virtual reality–based exercise; CT, cognitive training; AE-CT, aerobic exercise plus cognitive training; RT-CT, resistance training plus cognitive training; MBE-CT, mind–body exercise plus cognitive training; ME-CT, multicomponent exercise plus cognitive training.

DST

**ME**	0.43 (-0.60,1.46)	0.54 (-0.44,1.52)	0.62 (-0.27,1.50)	0.71 (-0.35,1.76)	1.07 (-0.26,2.39)	**1.05 (0.14,1.95)**	**1.09 (0.04,2.13)**	**1.35 (0.11,2.60)**
Rank 1st	**CT**	0.11 (-0.54,0.76)	0.19 (-0.43,0.80)	0.28 (-0.30,0.86)	0.64 (-0.27,1.55)	**0.62 (0.13,1.11)**	0.66 (-0.06,1.37)	**0.93 (0.13,1.73)**
	Rank 2nd	**MBE-CT**	0.08 (-0.43,0.58)	0.17 (-0.52,0.86)	0.53 (-0.52,1.58)	**0.51 (0.08,0.93)**	0.55 (-0.12,1.22)	0.82 (-0.14,1.77)
Rank 1st		Rank 3rd	**MBE**	0.09 (-0.56,0.75)	0.45 (-0.58,1.48)	**0.43 (0.06,0.80)**	0.47 (-0.17,1.11)	0.74 (-0.19,1.67)
**AE**	Rank 2nd		Rank 4th	**AE-CT**	0.36 (-0.54,1.25)	0.34 (-0.21,0.88)	0.38 (-0.37,1.13)	0.65 (-0.13,1.43)
1.89 (-2.33,6.10)	**AE-CT**	Rank 3rd		Rank 5th	**RT**	-0.02 (-0.98,0.94)	0.02 (-1.07,1.11)	0.29 (-0.15,0.73)
2.89 (-4.60,10.38)	1.00 (-6.57,8.57)	**D/VRE**	Rank 4th		Rank 7th	**AC**	0.04 (-0.48,0.56)	0.31 (-0.54,1.16)
4.51 (-1.57,10.58)	2.62 (-3.55,8.79)	1.62 (-6.11,9.34)	**RT**	Rank 6th		Rank 6th	**AE**	0.27 (-0.73,1.27)
4.87 (-1.28,11.03)	2.98 (-3.26,9.23)	1.98 (-4.94,8.91)	0.37 (-6.56,7.30)	**MBE**	Rank 5th		Rank 8th	**WLC**
**4.81 (0.03,9.58)**	2.92 (-1.97,7.81)	1.92 (-5.35,9.19)	0.30 (-6.01,6.61)	-0.07 (-5.07,4.94)	**CT**	Rank 7th		Rank 9th
**4.96 (0.61,9.30)**	3.07 (-1.41,7.54)	2.07 (-4.38,8.52)	0.45 (-3.80,4.70)	0.08 (-5.39,5.56)	0.15 (-4.51,4.82)	**WLC**		

SCWT

Supplementary Figure 4

League table from the network meta-analysis comparing intervention nodes in older adults with mild cognitive impairment. Mean differences (MDs) with 95% confidence intervals (CIs) are presented for SCWT in the lower-left triangle, and standardized mean differences (SMDs) with 95% CIs are presented for DST in the upper-right triangle. Symbols denote certainty of evidence according to the GRADE approach: * high; † moderate; ‡ low; § very low. AE, aerobic exercise; RT, resistance training; MBE, mind–body exercise; ME, multicomponent exercise; D/VRE, digital or virtual reality–based exercise; CT, cognitive training; AE-CT, aerobic exercise plus cognitive training; RT-CT, resistance training plus cognitive training; MBE-CT, mind–body exercise plus cognitive training; ME-CT, multicomponent exercise plus cognitive training.

DRA

Rank											
Rank 1st	ME	0.22 (-0.28,0.72)	0.19 (-0.52,0.90)	0.28 (-0.23,0.79)	0.35 (-0.39,1.09)	0.44 (-0.18,1.06)	0.49 (-0.09,1.06)	**0.52 (0.02,1.02)**	**0.49 (0.00,0.99)**	0.57 (-0.07,1.20)	**1.69 (0.88,2.50)**
Rank 2nd		AE	-0.03 (-0.59,0.52)	0.05 (-0.26,0.37)	0.13 (-0.64,0.89)	0.22 (-0.37,0.81)	0.26 (-0.18,0.70)	0.29 (-0.32,0.91)	**0.27 (0.05,0.49)**	0.34 (-0.15,0.84)	**1.47 (0.63,2.31)**
Rank 3rd			AE-CT	0.09 (-0.47,0.65)	0.16 (-0.75,1.07)	0.25 (-0.51,1.01)	0.30 (-0.34,0.94)	0.33 (-0.46,1.12)	0.30 (-0.21,0.81)	0.38 (-0.30,1.06)	**1.50 (0.53,2.47)**
Rank 4th				ME-CT	0.07 (-0.67,0.81)	0.17 (-0.43,0.76)	0.21 (-0.24,0.66)	0.24 (-0.39,0.87)	0.22 (-0.02,0.45)	0.29 (-0.21,0.79)	**1.42 (0.61,2.22)**
Rank 5th					RT-CT	0.09 (-0.79,0.98)	0.14 (-0.69,0.96)	0.17 (-0.69,1.03)	0.14 (-0.61,0.90)	0.22 (-0.65,1.08)	**1.34 (0.49,2.19)**
Rank 6th						D/VRE	0.04 (-0.44,0.52)	0.08 (-0.49,0.64)	0.05 (-0.51,0.61)	0.12 (-0.47,0.72)	**1.25 (0.30,2.20)**
Rank 7th							CT	0.03 (-0.59,0.65)	0.01 (-0.38,0.39)	0.08 (-0.30,0.46)	**1.21 (0.32,2.09)**
Rank 8th								WLC	-0.03 (-0.63,0.58)	0.05 (-0.64,0.74)	**1.17 (0.25,2.10)**
Rank 9th									AC	0.07 (-0.38,0.52)	**1.20 (0.38,2.02)**
Rank 10th										MBE	**1.13 (0.20,2.05)**
Rank 11th											RT

MFA

Rank							
Rank 1st	ME-CT						
Rank 2nd	0.26 (-0.63,1.15)	AE					
Rank 3rd	0.49 (-0.31,1.29)	0.23 (-0.16,0.63)	AC				
Rank 4th	0.57 (-0.44,1.58)	0.31 (-0.42,1.05)	0.08 (-0.54,0.70)	CT			
Rank 5th	0.66 (-0.61,1.92)	0.40 (-0.50,1.30)	0.16 (-0.82,1.15)	0.09 (-1.08,1.25)	ME		
Rank 6th	0.68 (-0.58,1.94)	0.42 (-0.62,1.47)	0.19 (-0.78,1.16)	0.11 (-1.04,1.27)	0.02 (-1.36,1.41)	MBE-CT	
Rank 7th	0.80 (-0.46,2.06)	0.54 (-0.50,1.59)	0.31 (-0.66,1.28)	0.23 (-0.51,0.98)	0.15 (-1.24,1.53)	0.12 (-1.25,1.50)	MBE

Supplementary Figure 5

League table from the network meta-analysis comparing intervention nodes in older adults with mild cognitive impairment. Standardized mean differences (SMDs) with 95% confidence intervals (CIs) are presented for MFA in the lower-left triangle and for DRA in the upper-right triangle. Symbols denote certainty of evidence according to the GRADE approach: * high; † moderate; ‡ low; § very low. AE, aerobic exercise; RT, resistance training; MBE, mind–body exercise; ME, multicomponent exercise; D/VRE, digital or virtual reality–based exercise; CT, cognitive training; AE-CT, aerobic exercise plus cognitive training; RT-CT, resistance training plus cognitive training; MBE-CT, mind–body exercise plus cognitive training; ME-CT, multicomponent exercise plus cognitive training.

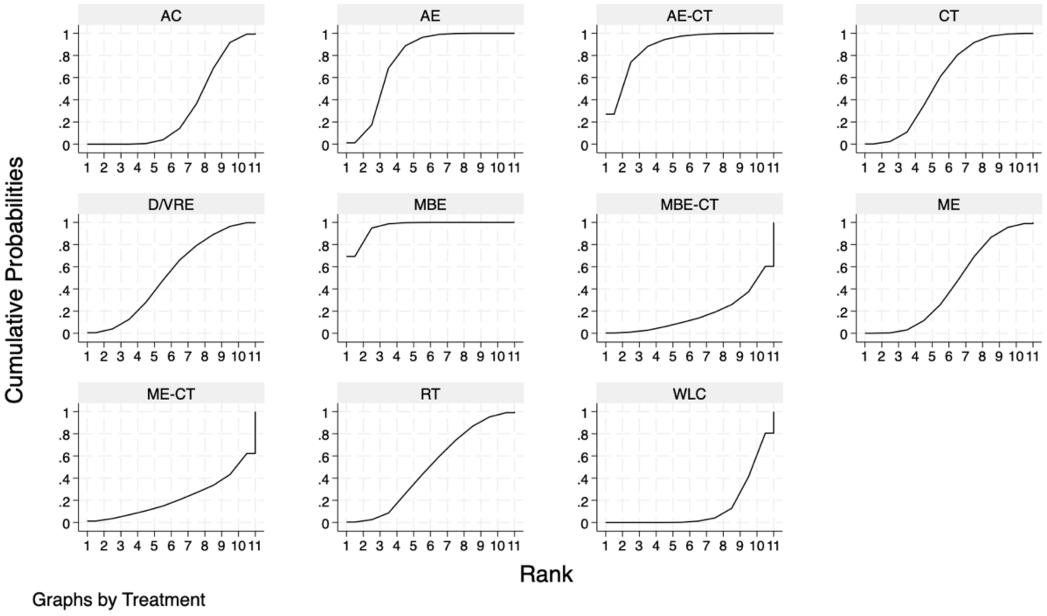

Supplementary Figure 6. Cumulative ranking probability curves of different interventions for MoCA in older adults with mild cognitive impairment.

The figure presents the cumulative probabilities of each intervention being ranked from best to worst for Montreal Cognitive Assessment (MoCA). A steeper increase at lower ranks suggests a greater likelihood of better performance. AC, active control; AE, aerobic exercise; AE-CT, aerobic exercise plus cognitive training; CT, cognitive training; D/VRE, digital or virtual reality–based exercise; MBE, mind–body exercise; MBE-CT, mind-body exercise plus cognitive training; ME, multicomponent exercise; ME-CT, multicomponent exercise plus cognitive training; RT, resistance training; WLC, wait-list or usual care control.

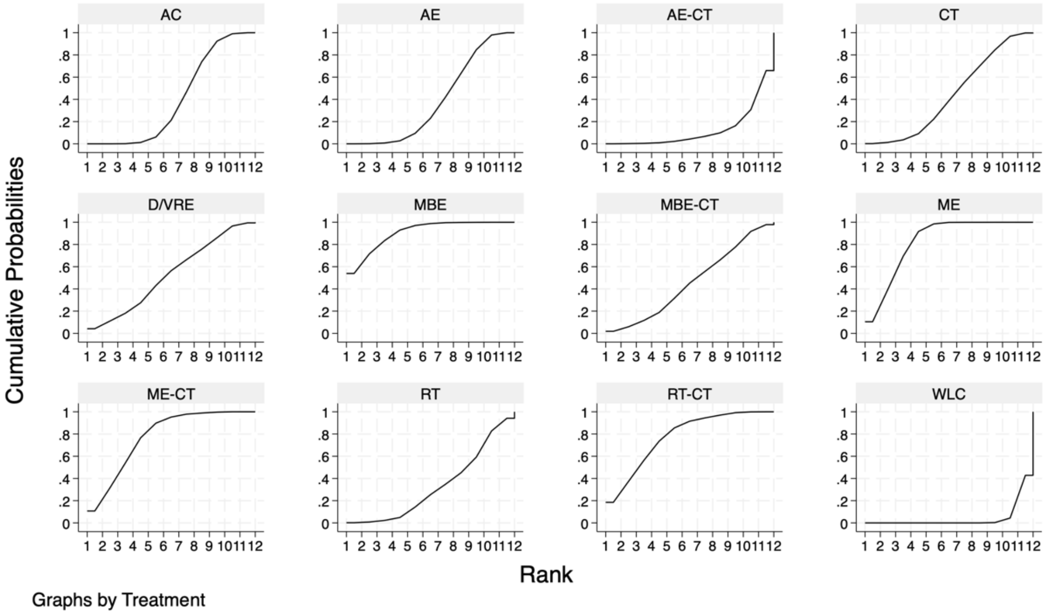

Supplementary Figure 7. Cumulative ranking probability curves of different interventions for MMSE in older adults with mild cognitive impairment.

The figure presents the cumulative probabilities of each intervention being ranked from best to worst for the Mini-Mental State Examination (MMSE). A steeper increase at lower ranks suggests a greater likelihood of better performance. AC, active control; AE, aerobic exercise; AE-CT, aerobic exercise plus cognitive training; CT, cognitive training; D/VRE, digital or virtual reality–based exercise; MBE, mind-body exercise; MBE-CT, mind-body exercise plus cognitive training; ME, multicomponent exercise; ME-CT, multicomponent exercise plus cognitive training; RT, resistance training; RT-CT, resistance training plus cognitive training; WLC, wait-list or usual care control.

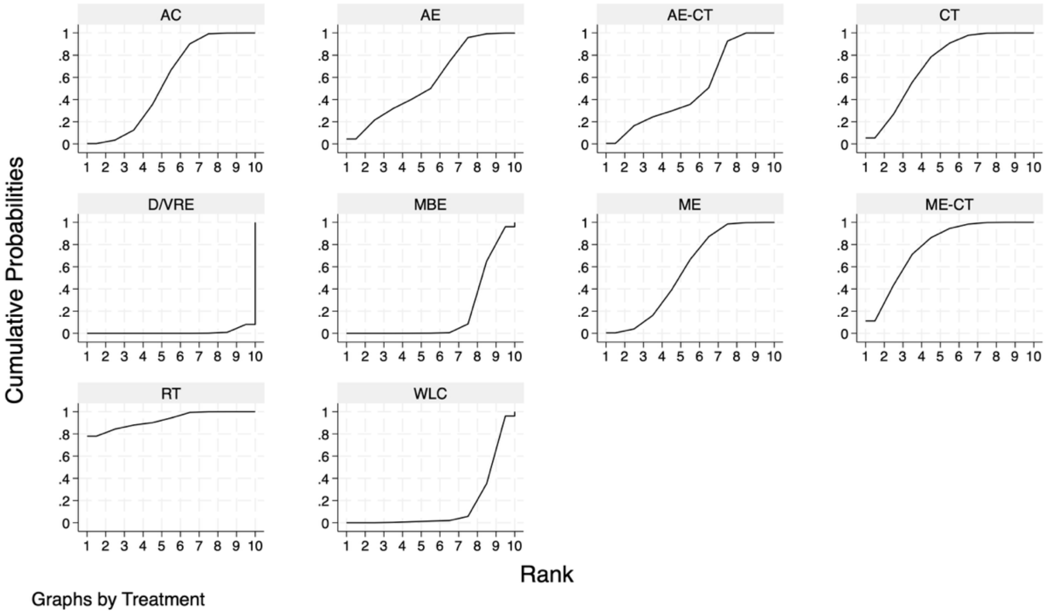

Supplementary Figure 8. Cumulative ranking probability curves of different interventions for ADAS-Cog in older adults with mild cognitive impairment.

The figure presents the cumulative probabilities of each intervention being ranked from best to worst for the Alzheimer's Disease Assessment Scale–Cognitive Subscale (ADAS-Cog). A steeper increase at lower ranks suggests a greater likelihood of better performance. AC, active control; AE, aerobic exercise; AE-CT, aerobic exercise plus cognitive training; CT, cognitive training; D/VRE, digital or virtual reality–based exercise; MBE, mind-body exercise; ME, multicomponent exercise; ME-CT, multicomponent exercise plus cognitive training; RT, resistance training; WLC, wait-list or usual care control.

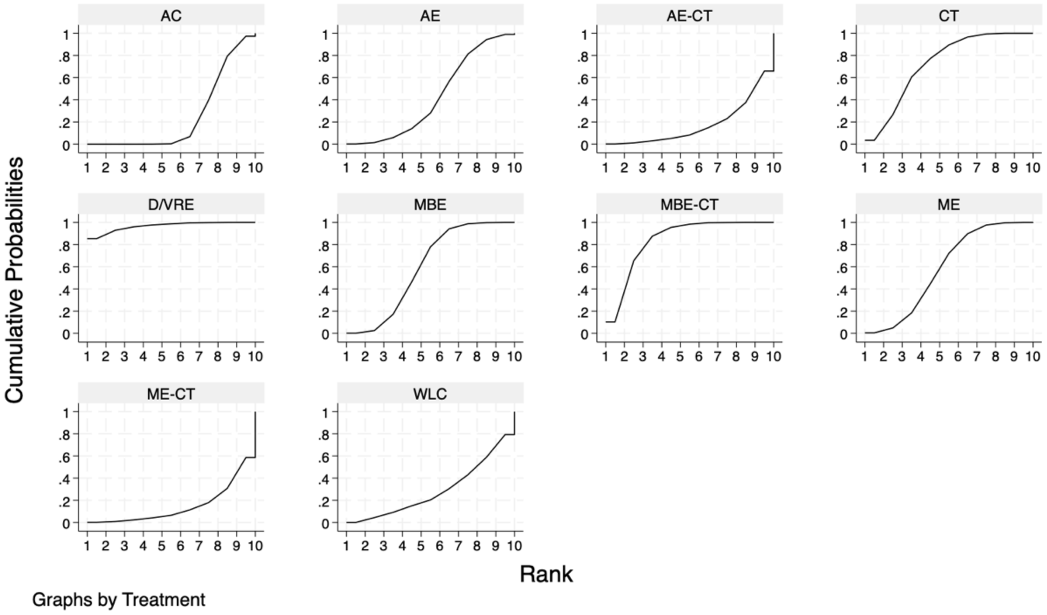

Supplementary Figure 9. Cumulative ranking probability curves of different interventions for TMT-B in older adults with mild cognitive impairment.

The figure presents the cumulative probabilities of each intervention being ranked from best to worst for the Trail Making Test Part B (TMT-B). A steeper increase at lower ranks suggests a greater likelihood of better performance. AC, active control; AE, aerobic exercise; AE-CT, aerobic exercise plus cognitive training; CT, cognitive training; D/VRE, digital or virtual reality–based exercise; MBE, mind-body exercise; MBE-CT, mind-body exercise plus cognitive training; ME, multicomponent exercise; ME-CT, multicomponent exercise plus cognitive training; WLC, wait-list or usual care control.

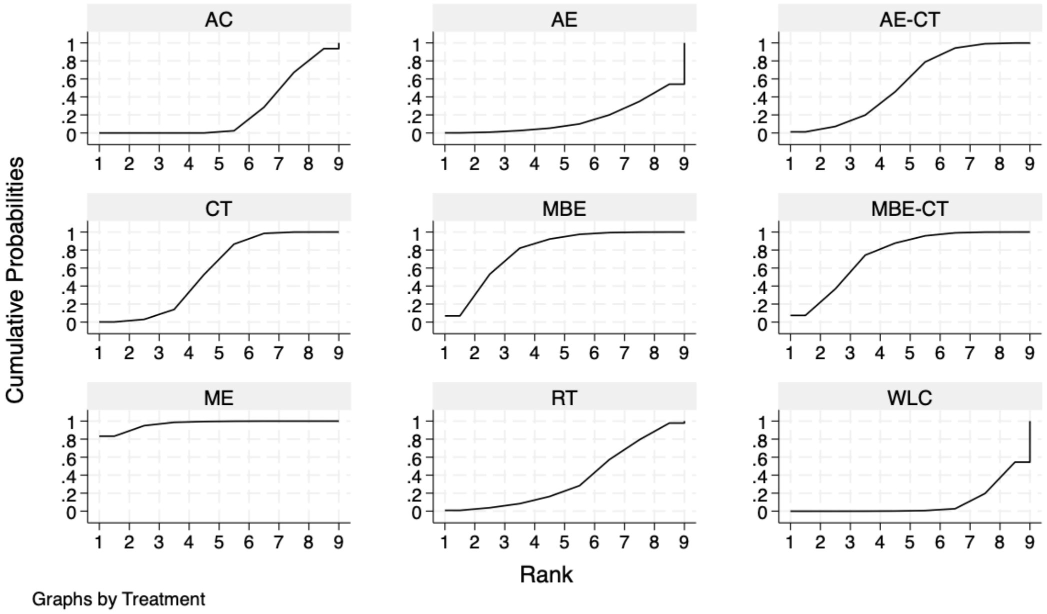

Supplementary Figure 10. Cumulative ranking probability curves of different interventions for DST in older adults with mild cognitive impairment.

The figure presents the cumulative probabilities of each intervention being ranked from best to worst for the Digit Span Test (DST). A steeper increase at lower ranks suggests a greater likelihood of better performance. AC, active control; AE, aerobic exercise; AE-CT, aerobic exercise plus cognitive training; CT, cognitive training; MBE, mind-body exercise; MBE-CT, mind-body exercise plus cognitive training; ME, multicomponent exercise; RT, resistance training; WLC, wait-list or usual care control.

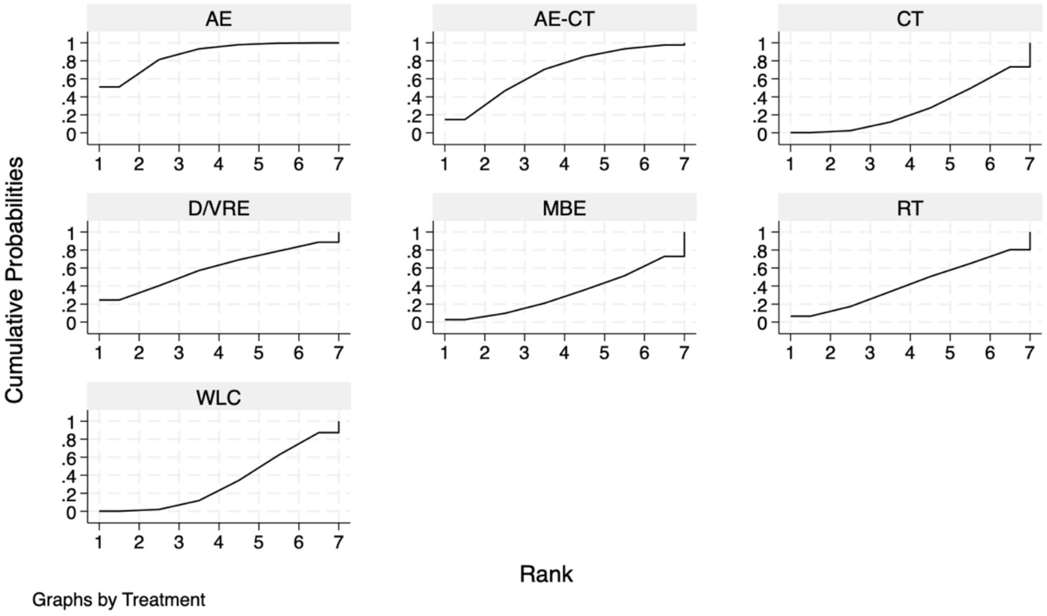

Supplementary Figure 11. Cumulative ranking probability curves of different interventions for SCWT in older adults with mild cognitive impairment.

The figure presents the cumulative probabilities of each intervention being ranked from best to worst for the Stroop Color–Word Test (SCWT). A steeper increase at lower ranks suggests a greater likelihood of better performance. AE, aerobic exercise; AE-CT, aerobic exercise plus cognitive training; CT, cognitive training; D/VRE, digital or virtual reality–based exercise; MBE, mind-body exercise; RT, resistance training; WLC, wait-list or usual care control.

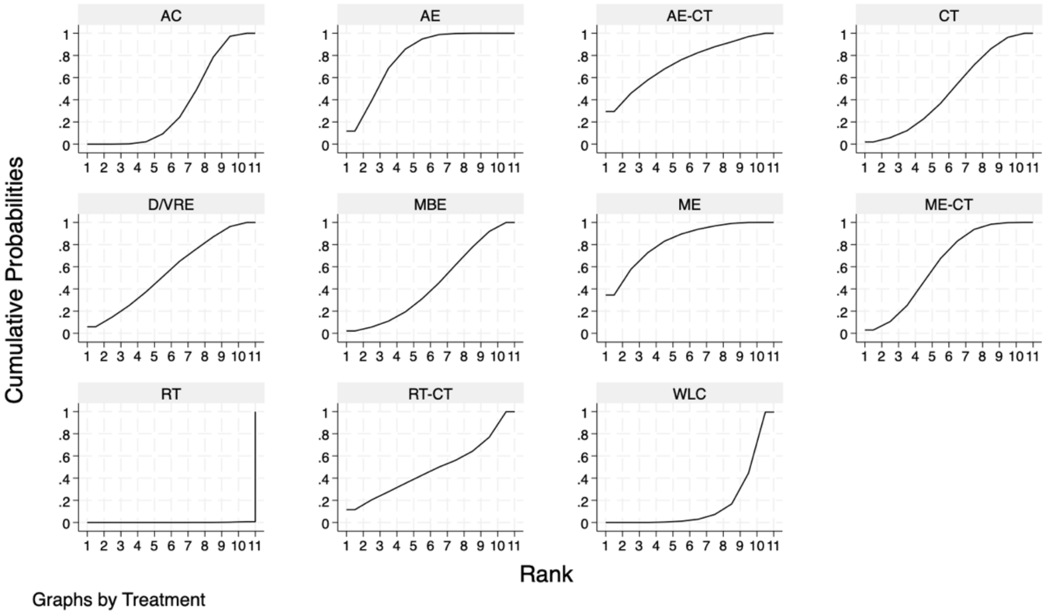

Supplementary Figure 12. Cumulative ranking probability curves of different interventions for DRA in older adults with mild cognitive impairment.

The figure presents the cumulative probabilities of each intervention being ranked from best to worst for DRA. A steeper increase at lower ranks suggests a greater likelihood of better performance. AC, active control; AE, aerobic exercise; AE-CT, aerobic exercise plus cognitive training; CT, cognitive training; D/VRE, digital or virtual reality–based exercise; MBE, mind-body exercise; ME, multicomponent exercise; ME-CT, multicomponent exercise plus cognitive training; RT, resistance training; RT-CT, resistance training plus cognitive training; WLC, wait-list or usual care control.

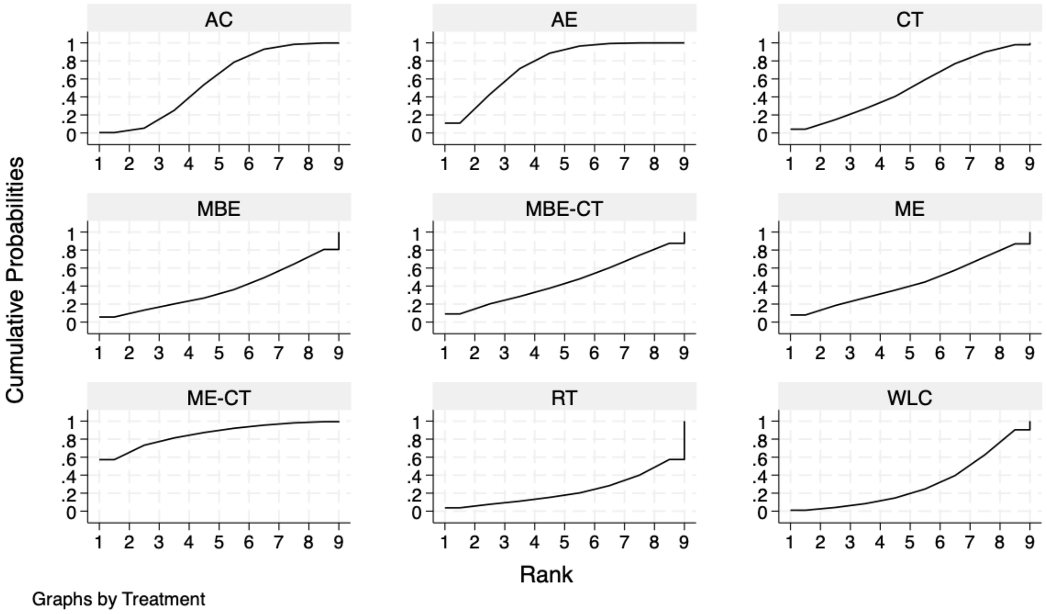

Supplementary Figure 13. Cumulative ranking probability curves of different interventions for MFA in older adults with mild cognitive impairment.

The figure presents the cumulative probabilities of each intervention being ranked from best to worst for MFA. A steeper increase at lower ranks suggests a greater likelihood of better performance. AC, active control; AE, aerobic exercise; CT, cognitive training; MBE, mind-body exercise; MBE-CT, mind-body exercise plus cognitive training; ME, multicomponent exercise; ME-CT, multicomponent exercise plus cognitive training; RT, resistance training; WLC, wait-list or usual care control.

Supplementary Figure 14. Comparison-adjusted funnel plot for the MoCA outcome in older adults with mild cognitive impairment.

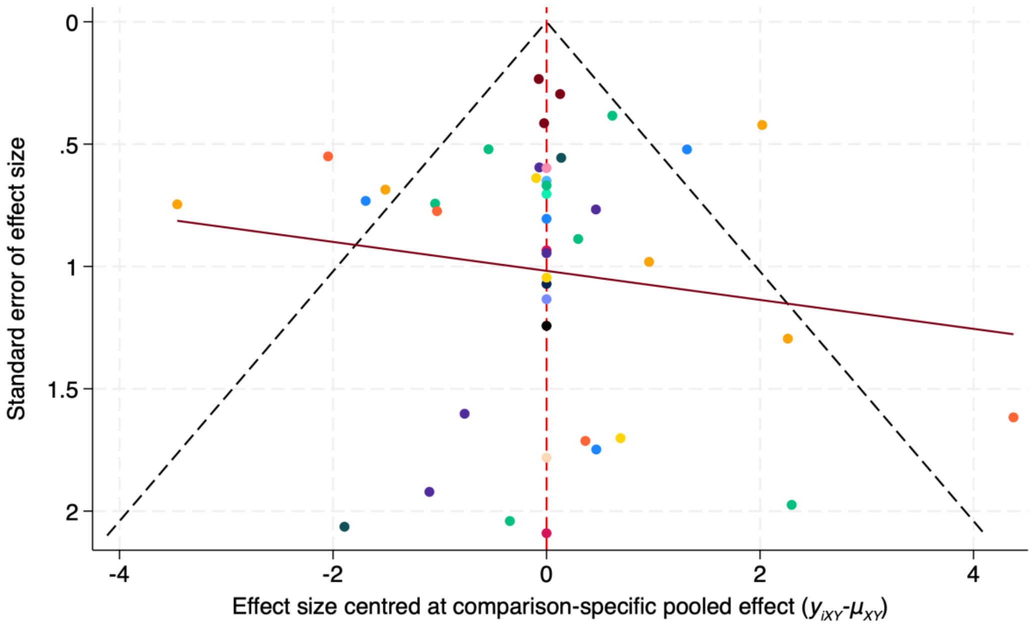

Supplementary Figure 15. Comparison-adjusted funnel plot for the MMSE outcome in older adults with mild cognitive impairment.

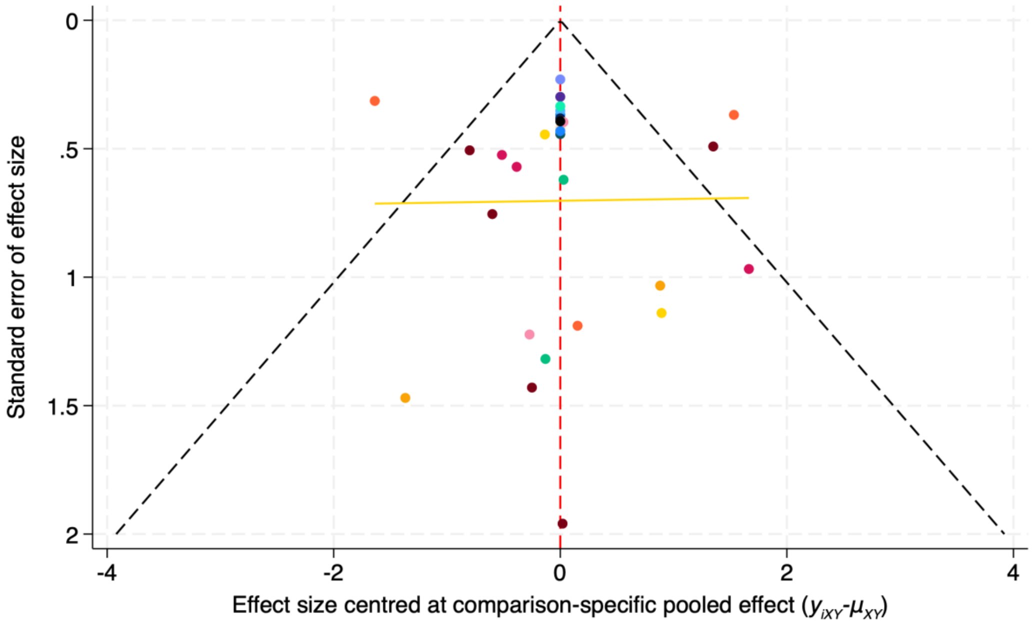

Supplementary Figure 16. Comparison-adjusted funnel plot for the TMT-B outcome in older adults with mild cognitive impairment.

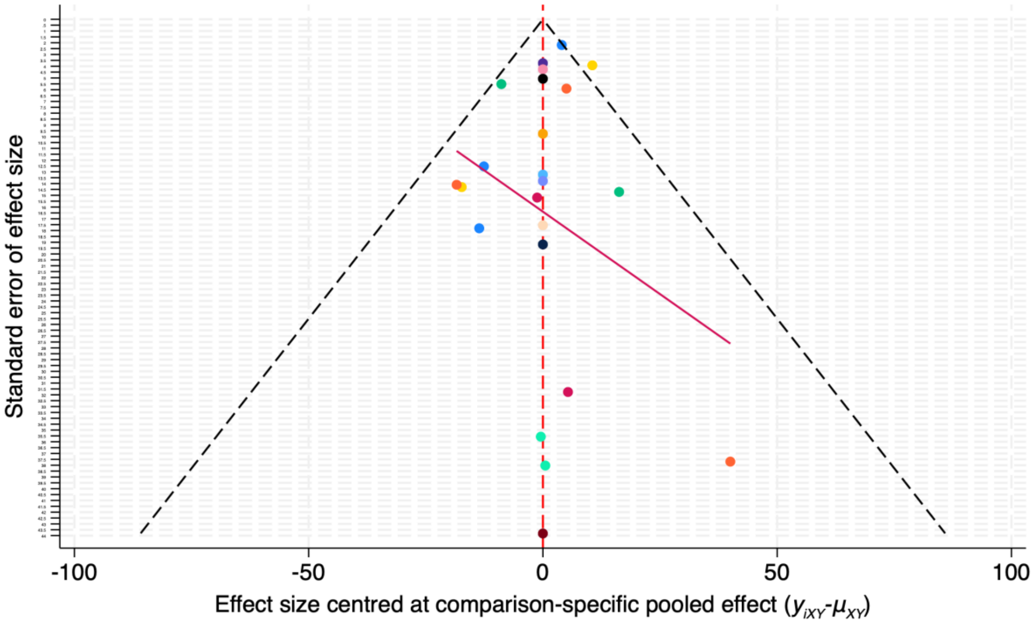

Supplementary Figure 17. Comparison-adjusted funnel plot for the DST outcome in older adults with mild cognitive impairment.

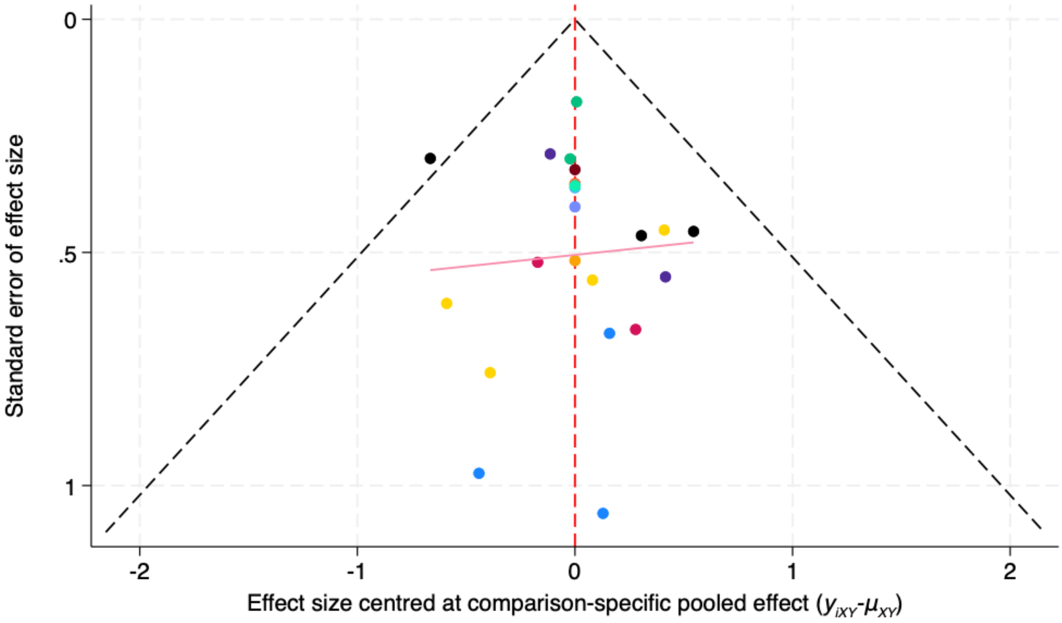

Supplementary Figure 18. Comparison-adjusted funnel plot for the DRA outcome in older adults with mild cognitive impairment.

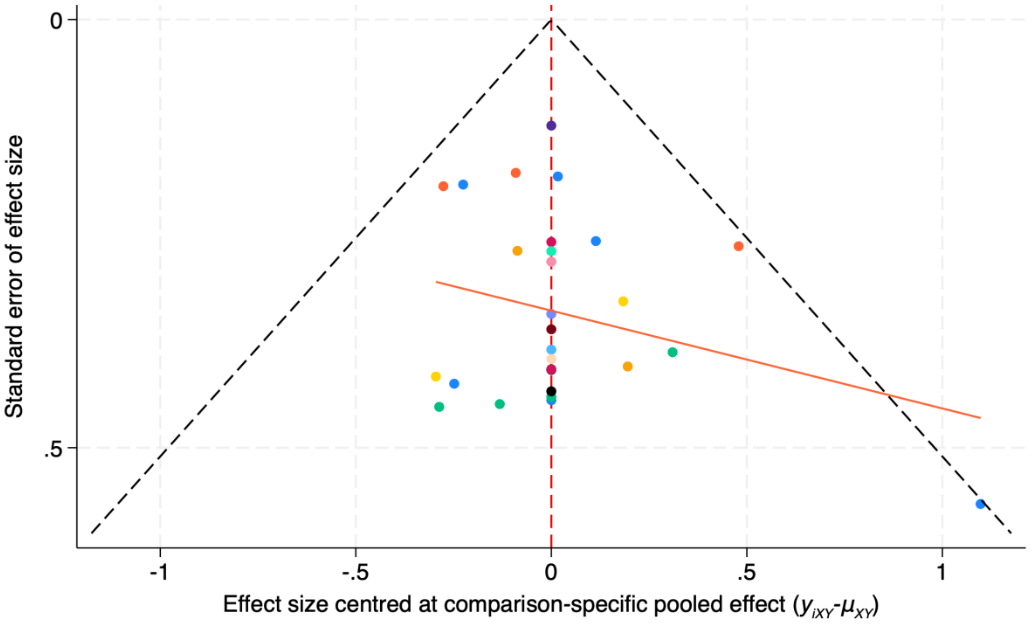

Supplementary Figure 19. Comparison-adjusted funnel plot for the MFA outcome in older adults with mild cognitive impairment.

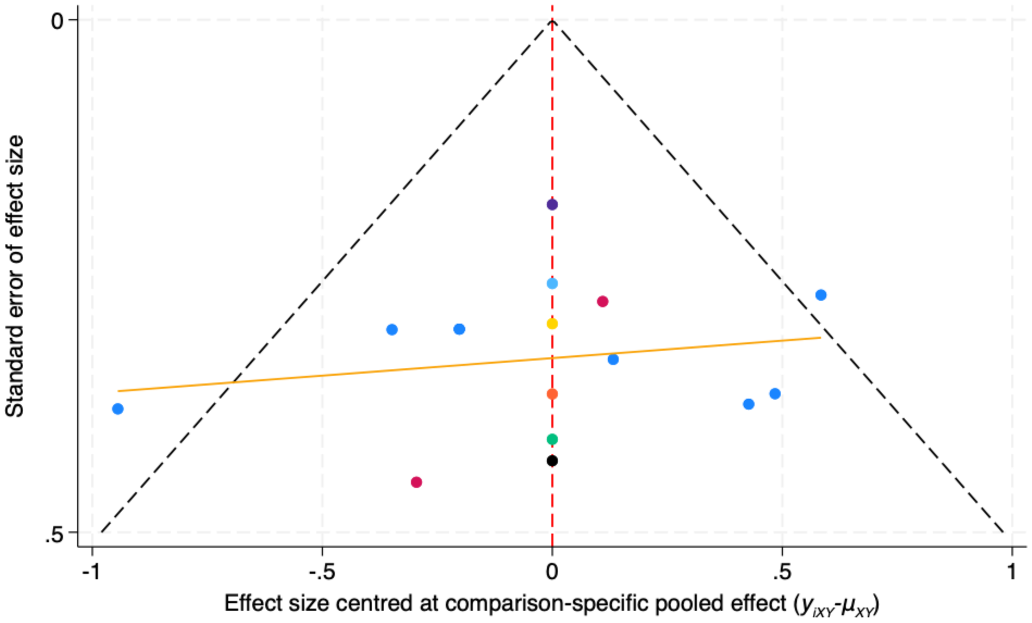

Supplement: Supplementary file 1 [file Data_Sheet_1.pdf]
